# Effect of an 18-Month Meditation Training on Regional Brain Volume and Perfusion in Older Adults: The Age-Well Randomized Clinical Trial

**DOI:** 10.1001/jamaneurol.2022.3185

**Published:** 2022-10-10

**Authors:** Gael Chételat, Antoine Lutz, Olga Klimecki, Eric Frison, Julien Asselineau, Marco Schlosser, Eider M. Arenaza-Urquijo, Florence Mézenge, Elizabeth Kuhn, Inès Moulinet, Edelweiss Touron, Sophie Dautricourt, Claire André, Cassandre Palix, Valentin Ourry, Francesca Felisatti, Julie Gonneaud, Brigitte Landeau, Géraldine Rauchs, Anne Chocat, Anne Quillard, Eglantine Ferrand Devouge, Patrik Vuilleumier, Vincent de La Sayette, Denis Vivien, Fabienne Collette, Géraldine Poisnel, Natalie L. Marchant

**Affiliations:** 1 Normandie Univ, UNICAEN, INSERM, U1237, Physiopathology and Imaging of Neurological Disorders (PhIND), Institut Blood and Brain @ Caen-Normandie, Cyceron, France; 2 Lyon Neuroscience Research Center INSERM U1028, CNRS UMR5292, Lyon 1 University, Lyon, France; 3 Swiss Center for Affective Sciences, Department of Neuroscience, University of Geneva Medical School, Geneva, Switzerland; 4 EUCLID/F-CRIN Clinical Trials Platform, INSERM, CHU Bordeaux, University of Bordeaux, CIC1401-EC, Bordeaux, France; 5 Division of Psychiatry, University College London, London, United Kingdom; 6 Department of Psychology, Faculty of Psychology and Educational Sciences, University of Geneva, Geneva, Switzerland; 7 Barcelonabeta Brain Research Center, Fundación Pasqual Maragall, Barcelona, Spain; 8 Normandie Univ, UNIROUEN, Department of General Practice, Rouen, France; 9 Rouen University Hospital, CIC-CRB 1404, F 76000, Rouen, France; 10 CHU Caen-Normandie, Department of Neurology, Caen, France; 11 CHU Caen-Normandie, Department of Clinical Research, Caen, France; 12 GIGA-CRC, In Vivo Imaging, Université de Liège and Belgian National Fund for Scientific Research, Liège, Belgium

## Abstract

**Importance:**

No lifestyle-based randomized clinical trial directly targets psychoaffective risk factors of dementia. Meditation practices recently emerged as a promising mental training exercise to foster brain health and reduce dementia risk.

**Objective:**

To investigate the effects of meditation training on brain integrity in older adults.

**Design, Setting, and Participants:**

Age-Well was a randomized, controlled superiority trial with blinded end point assessment. Community-dwelling cognitively unimpaired adults 65 years and older were enrolled between November 24, 2016, and March 5, 2018, in France. Participants were randomly assigned (1:1:1) to (1) an 18-month meditation-based training, (2) a structurally matched non-native language (English) training, or (3) no intervention arm. Analysis took place between December 2020 and October 2021.

**Interventions:**

Meditation and non-native language training included 2-hour weekly group sessions, practice of 20 minutes or longer daily at home, and 1-day intensive practices.

**Main Outcomes and Measures:**

Primary outcomes included volume and perfusion of anterior cingulate cortex (ACC) and insula. Main secondary outcomes included a global composite score capturing metacognitive, prosocial, and self-regulatory capacities and constituent subscores.

**Results:**

Among 137 participants (mean [SD] age, 69.4 [3.8] years; 83 [60.6%] female; 54 [39.4%] male) assigned to the meditation (n = 45), non-native language training (n = 46), or no intervention (n = 46) groups, all but 1 completed the trial. There were no differences in volume changes of ACC (0.01 [98.75% CI, −0.02 to 0.05]; *P* = .36) or insula (0.01 [98.75% CI, −0.02 to 0.03]; *P* = .58) between meditation and no intervention or non-native language training groups, respectively. Differences in perfusion changes did not reach statistical significance for meditation compared with no intervention in ACC (0.02 [98.75% CI, −0.01 to 0.05]; *P* = .06) or compared with non-native language training in insula (0.02 [98.75% CI, −0.01 to 0.05]; *P* = .09). Meditation was superior to non-native language training on 18-month changes in a global composite score capturing attention regulation, socioemotional, and self-knowledge capacities (Cohen *d*, 0.52 [95% CI, 0.19-0.85]; *P* = .002).

**Conclusions and Relevance:**

The study findings confirm the feasibility of meditation and non-native language training in elderly individuals, with high adherence and very low attrition. Findings also show positive behavioral effects of meditation that were not reflected on volume, and not significantly on perfusion, of target brain areas.

**Trial Registration:**

ClinicalTrials.gov Identifier: NCT02977819

## Key Points

**Question:**

Could meditation, a mental training approach toward attention and emotion regulation, preserve brain structure and function in cognitively unimpaired older adults?

**Findings:**

In this randomized clinical trial that included 137 cognitively unimpaired community-dwelling older individuals, the 18-month meditation-based intervention did not significantly modify the volume of the anterior cingulate cortex and insula compared with a passive or active control, respectively.

**Meaning:**

Future analyses on secondary outcomes will determine the measures most sensitive to meditation training and the factors associated with responsiveness to the intervention.

## Teaser

This randomized clinical trial investigates the effects of meditation training on brain integrity in older adults.

## Introduction

Strategies to prevent dementia are urgently needed. In later life, the main risk factors for dementia include smoking, depression, social isolation, physical inactivity, air pollution, and diabetes.^1^ There is also evidence for other potentially modifiable risk factors, including hearing loss, poor diet, anxiety, neuroticism, repetitive negative thinking, and sleep disorders.^1,2,3^ Recent and ongoing lifestyle-based multidomain interventions thus include cognitive stimulation, physical activity, diet, and cardiovascular recommendations.^4,5,6^ However, psychoaffective risk factors, including depression, stress, and anxiety have not been directly targeted, to our knowledge.

Mental training that targets stress and attention regulation has the potential to improve both cognitive and emotional aspects of aging.^7,8^ Previous studies have shown that mindfulness meditation improves cognition, specifically in older adults across multiple domains including attention, executive functions, and self-awareness or meta-cognition.^9,10,11,12^ Mindfulness meditation can also reduce stress, anxiety, and depression,^13,14,15^ including in older adults.^16^ Moreover, meditation in young adults has been associated with brain structural and functional changes mainly in frontal and limbic networks,^10,17^ with the insula and anterior cingulate cortex (ACC) being the most sensitive regions to meditation training according to a recent meta-analysis.^18^ These interconnected brain regions form the salience network and are particularly involved in self-awareness,^19,20^ attentional, emotional and empathic processing,^15^ and self-regulation of attention and emotion.^20,21^ They are consistently reported in task-related functional magnetic resonance imaging (MRI) studies on meditation,^10,17^ showing increased activity during mindfulness and/or compassion meditation.^7^ Interestingly, these brain areas are also particularly sensitive to aging.^22,23,24^ Studies on meditation in elderly populations are sparse, with high risk of bias, and there are no randomized clinical trials (RCTs) with large samples. The 3 RCTs in older adults with neuroimaging end points used 8-week training and reported conflicting findings.^25,26,27^ Two cross-sectional studies assessing elderly expert meditators showed more age-preserved gray matter volume and/or glucose metabolism in various brain regions including the ACC and insula compared with nonmeditators.^22,28^

Thus, meditation appears to be a promising approach to preserve brain structure and function as well as cognition and thus to reduce dementia risk by directly targeting psychoaffective factors. Faced with methodological limitations in previous or ongoing studies, the Age-Well RCT of the Medit-Aging European project was designed to investigate the impact of an 18-month meditation intervention on the volume and perfusion of the ACC and insula (coprimary outcomes) compared with active (non-native language training) and passive (no intervention) control groups, respectively, and on a self-report–based global composite score capturing attention regulation, socioemotional, and self-knowledge capacities and its constituent subscores (main secondary outcomes) compared with the active control group to test for meditation-specific effects.

## Methods

### Study Design and Participants

The design and method of Age-Well have been described previously.^29^ The trial protocol and statistical analysis plan are available in Supplement 1. Briefly, our study was an 18-month monocentric, randomized, observer-blind controlled superiority clinical trial with 3 parallel groups: 1 group with a meditation-based training (intervention group), 1 group with structurally matched non-native language training (English learning = active control group), and 1 group with no intervention (passive control group). Participants fulfilling eligibility criteria were invited to the baseline preintervention visit and then randomized. Participants were enrolled between November 24, 2016, and March 5, 2018. The 18-month intervention period started just after randomization, and participants had a midintervention visit at 9 months and a postintervention visit at the end of the intervention. Participants were 65 years or older, community-dwelling, native French speakers, retired for at least 1 year, had 7 years or more of education, and performed within the normal range for age and educational level on standardized cognitive tests (see Tables 1 and 2 in Poisnel et al^29^ for details). They had no evidence of major neurological or psychiatric disorders, no history of cerebrovascular disease, chronic disease or acute unstable illness, and no current medication that could interfere with cognitive functioning. Full eligibility criteria are listed in eAppendix 1 in Supplement 2. All participants gave their written informed consent to participate in the study. The Age-Well RCT, sponsored by Institut National de la Santé et de la Recherche Médicale (INSERM), was approved by the ethics committee (CPP Nord-Ouest III, Caen).

Detailed biological, behavioral, neuroimaging, and sleep measures were collected in Caen, France, at the preintervention and postintervention visits (the full list is available from Poisnel et al^29^ and eAppendix 2 in Supplement 2), including the structural T1-weighted MRI and early ^18^F-florbetapir (Amyvid; Lily Diagnostics) positron emission tomography (PET) scan, used for the primary outcomes, and the self-reported measures used to compute the global composite score and subscores used as the main secondary outcomes.^30^

### Randomization and Masking

Eligible participants from each of the 3 waves were randomized after their baseline assessment. They were randomly assigned (1:1:1) to the meditation, non-native language training (active control), or no intervention (passive control) arm according to a randomization list with permuted blocks of varying size (6 and 9), which was generated centrally by a biostatistician at the EUCLID clinical trials platform. All study personnel, including the investigators and outcome assessors, were masked to treatment allocation. Only the meditation and non-native language teachers and the trial-independent statisticians and data monitoring infrastructure staff were unmasked or partially unmasked. See further details in eAppendix 3 in Supplement 2.

### Interventions

The meditation and non-native language training interventions were structurally equivalent in overall course length, class time, and home activities, as well as level of expertise and number of teachers per class. Thus, they had 2-hour weekly group sessions, daily home practice (minimum 20 minutes), and 1-day intensive practice (5 hours) during the intervention. During the study, participants were strongly encouraged not to practice the activity proposed in the other group(s).

For each intervention, a manual describing the detailed procedure was written before the study started. Both interventions have been described in detail previously.^29^ Briefly, the meditation intervention consisted of a secular program of meditation training labeled the Silver Santé Study Meditation Programme especially designed for this study, based on preexisting interventions, and composed of mindfulness and loving kindness and compassion meditations. The non-native language training program consisted of English language exercises designed to reinforce each participant’s abilities in comprehension, writing, and speaking. Participants in the passive control group were requested not to change their habits, ie, continue living as they used to before entering the study.

### Outcomes Measures

The coprimary outcomes were (1) for the comparison between meditation and no intervention groups, the 18-month changes in ACC volume and perfusion (from pre- to postintervention) and (2) for the comparison between meditation and non-native language training groups, the 18-month changes in insula volume and perfusion. These brain regions were selected as they are known to be particularly sensitive both to aging and to meditation practice (see Introduction and Lutz et al^7^). The ACC was expected to be modified by both interventions (compared with no intervention), as this structure is considered a brain signature of cognitive reserve and brain maintenance in general,^31^ and to be more resistant to aging with bilingual experience in particular.^32^ In contrast, the insula was expected to be modified specifically in the meditation compared with the non-native language training group, given its role in emotional and empathic processing.^33^

The ACC and insula volume and perfusion measures were obtained from T1-weighted MRI and early ^18^F-florbetapir PET scans, respectively. Note that while early-phase florbetapir has been shown to be highly correlated with fludeoxyglucose-, arterial spin‐labeled–, and H_2_O-PET, it remains a surrogate marker of perfusion and glucose metabolism. The procedure is detailed in eAppendix 3 in Supplement 2 and described elsewhere.^34^ Briefly, T1-weighted images were segmented, normalized, and corrected for nonlinear warping so that values were corrected for brain size. Early ^18^F-florbetapir-PET images were normalized and scaled by the white matter to obtain standardized uptake values ratio. Averaged gray matter volume and perfusion values were extracted from the resulting images in our 2 regions of interest.

The main secondary outcomes have been defined after trial commencement so as to select, among the huge amount of secondary outcomes (eAppendix 2 in Supplement 2), the most relevant according to the goal and hypotheses of the Age-Well RCT. They consisted of the comparison between meditation and non-native language training on changes from baseline to 18 months in a self-report–based global composite score and its constituent subscores capturing (1) attention regulation (ie*,* attentional subscore), (2) socioemotional capacities (ie, constructive subscore), and (3) self-knowledge capacities to understand one’s own psychological processes (ie, deconstructive subscore). This global score and its subscores, recently operationalized,^30,35^ draw on self-report measures used in Age-Well (eAppendix 4 in Supplement 2).^29^ Each composite subscore was computed by averaging the z scores of the scales that were assigned to the respective composite, while the global composite score corresponded to the mean of the 3 composite subscores.

Adverse events and serious adverse events were recorded throughout the study when reported by the participants and systematically at each study visit during a consultation with a physician.

### Sample Size

The trial was powered to detect a relevant effect size of 0.75 for the meditation intervention, as suggested by a meta-analysis of meditation effects on morphometric neuroimaging markers.^17^ To detect an effect size of 0.75 for each of the 4 comparisons, with 80% power and a 2-sided type I error of 1.25% (Bonferroni correction for test multiplicity), 42 participants per arm (126 in total) were required. To account for analyses on secondary outcomes, a total sample size of 150 participants was fixed. A statistical analysis plan was developed and validated by the trial steering committee before database lock and analyses.

### Statistical Analyses

The primary outcome analysis was performed according to an intention-to-treat principle, including all randomized participants, as planned in the statistical analysis plan. Missing data on neuroimaging markers were handled using a missing = failure strategy, where missing values were replaced by the most detrimental value of change observed in all groups combined for a given outcome. Outcomes were compared between groups using a linear regression model adjusted for baseline prognostic factors (sex, median-centered age, level of education, and Mini-Mental State Examination) and baseline outcome value. Comparisons were made with a 1.25 type I error rate. Additional analyses were performed, including a sensitivity analysis to missing data, a “minimum intervention” analysis, and post hoc analyses for additional adjustment, stratification, and subgroup analyses (eAppendix 3 in Supplement 2).

For the secondary outcomes, the statistical analysis plan focused on between-group differences in mean changes in the global composite score and subscores in meditation vs non-native language training so as to highlight the specific effects of meditation compared with its active control. For each composite score and subscore, we built 1 mixed-effect linear regression model incorporating data from pre- and postintervention with an interaction term between visit and group, controlling for baseline scores of the outcome. In all mixed-effects regression models, missing data were not replaced and assumed to be missing at random.

Analyses were performed using SAS statistical software version 9.4 (SAS Institute) for the coprimary outcomes and R version 4.0.2 (R Foundation) for the secondary outcomes. Two-sided *P* values were statistically significant at less than .0125 for each coprimary outcome (Bonferroni correction for test multiplicity) and less than .05 for secondary outcomes. Analysis took place between December 2020 and October 2021 (and April 2022 for the supplementary analyses during the revision process).

## Results

**Figure 1.**
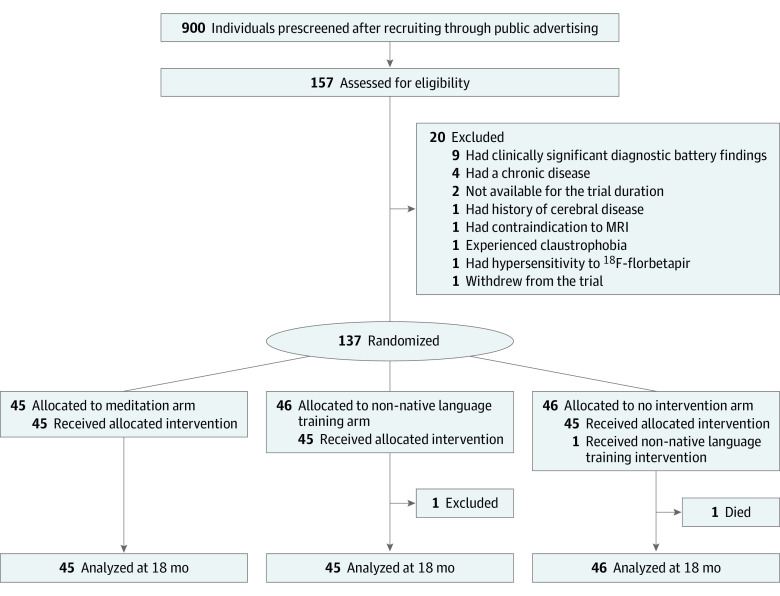
Trial Profile Of 137 randomized participants, 1 was excluded from all analyses due to major eligibility criteria not met (not included in the analyses), 1 died during follow-up, and 1 revealed not to have followed his allocated arm (randomized to no intervention but attended non-native language training); those 2 later participants were retained in the analyses and treated by the intention-to-treat principle, as specified in the statistical analysis plan. MRI indicates magnetic resonance imaging.

Of 157 participants assessed for eligibility, 137 were randomized (mean [SD] age, 69.4 [3.8] years; 83 [60.6%] female; 54 [39.4%] male), among whom 1 participant was excluded from all analyses because of major eligibility criteria not met (decision by the trial steering committee blinded to group allocation for head trauma with loss of consciousness >1 hour; Figure 1). Among 136 participants included in the analyses, 45 were randomized to meditation, 45 to non-native language training, and 46 to the no intervention groups. One participant died during follow-up, while another participant was revealed to have not followed his allocated arm; both were retained in the analyses and included using the intention-to-treat principle, as specified in the statistical analysis plan.

Baseline characteristics of the 136 participants included in the intention-to-treat analysis are detailed in Table 1. There were no major clinical differences in any demographic or clinical characteristics between groups. The median (IQR) follow-up time between pre- and postintervention visits was 21.1 (20.7-21.7) months. The mean (SD) class attendance for meditation and non-native language groups was 62.0 (8.9) and 57.9 (12.2) of 72 classes, respectively. Of 45 participants, 44 in the meditation/non-native language groups attended at least 20% of their intervention classes.

**Table 1.** Baseline Characteristics

Characteristic	Mean (SD)		
	Meditation (n = 45)	Non-native language training (n = 45)	No intervention (n = 46)
Age, median (IQR), y	68 (67-72)	69 (67-73)	68 (66-70)
Female, No. (%)	31 (69)	24 (53)	28 (61)
Male, No. (%)	14 (31)	21 (47)	18 (39)
Years of education	13 (3)	12 (3)	14 (2)
Years in retirement	7 (5-13)	9 (5-13)	6 (4-10)
Handedness laterality	92 (83-100)	91 (83-100)	92 (83-100)
BP level, mm Hg			
Systolic	143.6 (20.1)	145.3 (16.4)	136.7 (20.5)
Diastolic	86.1 (10.5)	86.6 (10.0)	84.8 (11.7)
BMI	26.1 (4.6)	26.5 (4.3)	25.9 (4.0)
Mini-Mental State Examination score	28.9 (1.2)	29.0 (1.0)	29.2 (0.9)
Montgomery–Åsberg Depression Rating Scale score	1.2 (1.2)	1.2 (1.5)	0.7 (1.0)
*APOE* ε4 carriers (≥1 allele), No. (%)	13 (29)	13 (29)	11 (24)
Positive for brain amyloid,^a^ No. (%)	11 (24)	11 (24)	6 (13)
Amyloid SUVR	1.26 (0.16)	1.27 (0.19)	1.21 (0.10)
Familial history of dementia, No. (%)	14 (31)	11 (24)	14 (30)
Presence of ≥1 Alzheimer disease risk factors,^b^ No. (%)	28 (62)	23 (51)	23 (51)

Abbreviations: APOE, apolipoprotein E; BMI, body mass index (calculated as weight in kilograms divided by height in meters squared); BP, blood pressure; SUVR, standardized uptake values ratio.

^a^ Methods detailed in eAppendix 3 in Supplement 2.

^b^ Alzheimer disease risk factors include *APOE* ε4 (≥1 allele), brain amyloid positivity, and familial history of dementia.

For the coprimary outcomes, data were missing on baseline early ^18^F-florbetapir-PET scans due to extravasation and technical problems for 2 participants (meditation, n = 1 and no intervention, n = 1), and data were missing on 18-month MRI and early ^18^F-florbetapir-PET scans for 1 participant in the no intervention group who died during follow-up.

**Figure 2.**
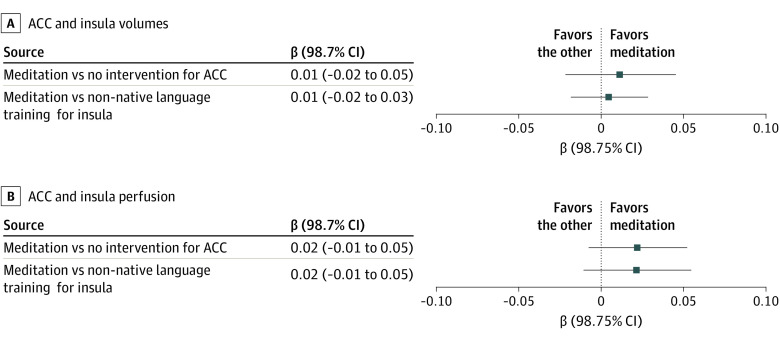
Forest Plots for Anterior Cingulate Cortex (ACC) and Insula Volume and Perfusion Results of the intention-to-treat analyses with missing = failure strategy are shown.

Primary end points at baseline and 18 months and their mean changes are reported in the eTable in Supplement 2, and intervention effects on the coprimary outcome measures in the intention-to-treat analyses are presented in Figure 2. The differences in the mean volume changes over 18 months between meditation and no intervention in the ACC (0.01 [98.75% CI, −0.02 to 0.05]) or the non-native language group in the insula (0.01 [98.75% CI, −0.02 to 0.03]) were not statistically significant (*P* = .36 and *P* = .58, respectively). As for perfusion, differences in the mean changes over 18 months, in favor of meditation compared with no intervention in the ACC (0.02 [98.75% CI, −0.01 to 0.05]) and compared with non-native language training in the insula (0.02 [98.75% CI, −0.01 to 0.05]) did not reach statistical significance (*P* = .06 and *P* = .09, respectively).

**Figure 3.**
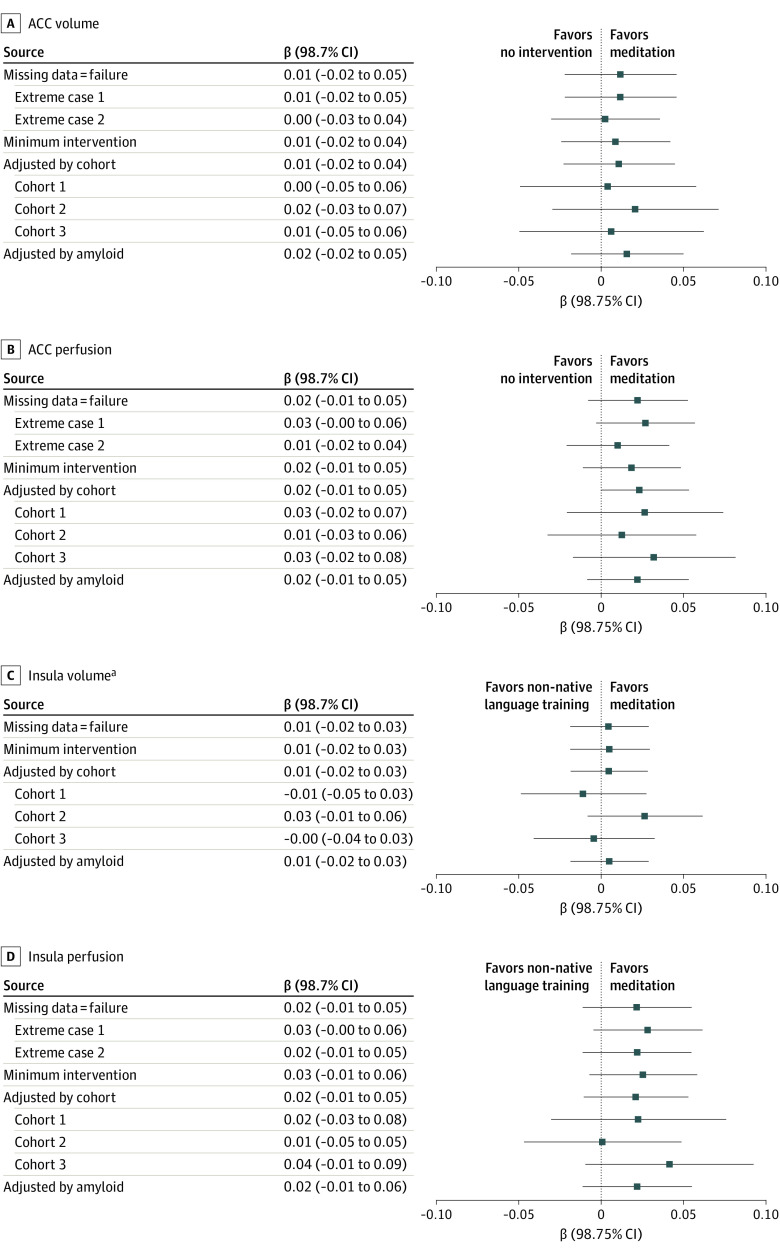
Sensitivity Analyses for Anterior Cingulate Cortex (ACC) and Insula Volume and Perfusion ^a^No missing outcome values for participants included in the comparison.

Results of the sensitivity and post hoc analyses are presented in Figure 3 and in eAppendix 5 in Supplement 2. Briefly, results were very similar to those of the main analyses, showing no between-group differences in the volume changes of the ACC or insula. Changes in the perfusion of the ACC or insula always favored meditation (compared with no intervention or non-native language training, respectively), but the between-group differences never reached statistical significance. Finally, the subgroup analyses showed that neither the recruitment wave nor the presence of risk factor(s) for Alzheimer disease (apolipoprotein E ε4 genotype, brain amyloid positivity, familial history of dementia, or presence of at least 1 of these risk factors) affected the results.

Regarding secondary outcomes, the global composite score and attention regulation subscore were not computed for 1 participant because of missing data at baseline on a subscale assigned to the attention regulation subscore. Secondary end points at baseline and 18 months and their standardized mean changes are reported in Table 2 and in eAppendix 6 and 7 in Supplement 2. The differences in the mean changes over 18 months between meditation and non-native language training in the global composite score (0.52 [95% CI, 0.19-0.85]; *P* = .002), attention regulation (0.38 [95% CI, 0.10-0.67]; *P* = .009), and socioemotional (0.31 [95% CI, 0.06-0.57]; *P* = .01) subscores were all statistically significant, while it was not statistically significant for the self-knowledge subscore (0.28 [95% CI, −0.01 to 0.58]; *P* = .06).

**Table 2.** Results From Mixed-Effects Models Assessing Change From Baseline to 18 Months in Meditation and Non-Native Language Training in Composite Scores^a^

Outcome	Standardized estimated change (95% CI)		Between-group difference in change meditation vs non-native language training	
	Meditation	Non-native language training	Mean (95% CI)	*P* value
Global	0.43 (0.20 to 0.67)	−0.09 (−0.12 to 0.30)	0.52 (0.19 to 0.85)	.002
Attention regulation capacities	0.48 (0.28 to 0.69)	0.10 (−0.10 to 0.30)	0.38 (0.10 to 0.67)	.009
Socioemotional capacities	0.04 (−0.15 to 0.23)	−0.27 (−0.46 to −0.08)	0.31 (0.06 to 0.57)	.02
Self-knowledge capacities	0.27 (0.07 to 0.48)	−0.01 (−0.22 to 0.20)	0.28 (−0.01 to 0.58)	.06

^a^ All analyses were adjusted for baseline scores of the outcome. Positive (negative) estimated mean between-group differences reflect increases (decreases) in composite scores in the meditation intervention.

### Adverse Events

One death was reported during study follow-up for a participant in the no intervention group (myocardial infarction, not related to the study). A total of 170 adverse events were recorded, 41 of which were considered serious (meditation, 13; non-native language training, 15; no intervention, 13). Among these, 7 adverse events (meditation, 3; non-native language training, 3; no intervention, 1) were judged to be related to study procedures (procedural complication, 4 [scans had to be redone]; extravasation, 2; asthenia, 1). No serious adverse event was related to the intervention.

## Discussion

Results indicate that the 18-month meditation-based intervention did not significantly modify the volume of the ACC and insula in older adults compared with a passive or active control, respectively; the between-group differences did not reach statistical significance for perfusion either. Regarding the main secondary outcomes, there were significant effects of meditation compared with non-native language training on the global composite score reflecting attention regulation, socioemotional, and self-knowledge capacities and two-thirds of its constituent subscores.

The fact that no effects were found on anterior cingulate and insula volumes in our study, despite being identified as structures most sensitive to meditation,^36^ might indicate that 18 months of meditation training is not sufficient to alter the effects of age on their volume. Meditation might alter volume in younger and more plastic brains but not halt the age- and disease-related brain volume decreases at older ages. While larger brain volumes were observed in cross-sectional studies in older expert meditators vs nonmeditators, especially in the ACC and insula,^22^ these differences might reflect meditation effects in younger ages, intense meditation practice accumulated throughout the adult lifespan, and/or between-group differences on other variables, such as lifestyle. Note that the lack of meditation effect on brain structure is consistent with the very recent publication from a large and rigorously controlled study.^37^

Regarding perfusion, both the differences in the anterior cingulate between the meditation and no intervention groups, and in the insula between the meditation and non-native language training, were not statistically significant but close to the threshold. Mean differences between groups, in favor of meditation, were rather low (0.023 and 0.022, respectively) but could represent a substantial gain compared with the mean 18-month change observed in the no intervention group (0.031 for both the ACC and insula). Assuming a linear loss over 18 months, the 74% and 71% reduction in rate of loss could translate into about 13 months less loss over the course of the intervention, in favor of the meditation group. One could hypothesize that a larger sample size or longer follow-up time would have yielded a significant effect of the intervention on perfusion.

The lack of significant effects on the coprimary outcomes could also be related to the design of the study, eg, the use of an RCT when the intervention, by its nature and duration, strongly relies on the motivation, preferences, and adherence of the participants. Alternative trial designs taking into account patient preference might be particularly relevant in the context of such nonpharmacological interventions.^38^ Moreover, as in most preventive trials, our population resulted in being, through self-selection, enriched with healthy participants with high education and reserve, and low probability of cognitive decline, which left limited room for lifestyle changes and intervention-related improvements.^39^ Finally, the selected coprimary outcomes were very specific neuroimaging measures that show variability and significant but protracted age-related changes from which it might be difficult to show a deviation in a short period of time in terms of aging effects.

Regarding secondary outcomes, meditation was superior to non-native language training on changing a global composite score and 2 of its subscores reflecting attention regulation and socioemotional capacities. As this composite score and its subscores are thought to measure core dimensions of well-being,^40^ these findings suggest meditation training–related effects on mental health and human flourishing. The attention regulation subscore increased after meditation only; in the context of meditation practices, this capacity allows a heightened awareness and monitoring of the contents of experience without becoming absorbed by them. Socioemotional capacities decreased substantially after non-native language training but not meditation training, suggesting that the difference observed may be due to maintenance of skills by meditation training.

### Limitations and Strengths

Our study has several limitations. The sample size was defined based on an expected effect size of 0.75 on neuroimaging measures, which was likely overestimated as it was defined from a meta-analysis including both preintervention-postintervention studies and cross-sectional studies in long-term meditation experts but also due to publication bias, considerable methodological caveats in the existing literature at that time, and general biases in estimating effect sizes in neuroimaging studies.^17,41^ We were not able to observe this effect size in the present study and may have been underpowered to observe a smaller but still clinically significant effect. Moreover, our sample was not representative of the global aging population as it included very healthy individuals (see above). Finally, early-phase florbetapir is an indirect, surrogate marker, of perfusion or fludeoxyglucose-PET, and the optimal processing method (scaling and timeframe) is still unclear.

Several strengths must also be noted. The primary end points were measured blinded to allocated intervention, the intervention was particularly long, we used both a passive and a carefully matched active control condition, and the exceptionally high adherence and low attrition (1 of 137 participants only) demonstrates the appropriateness of the interventions and feasibility of this approach in healthy and motivated elderly. The Age-Well clinical trial includes many complementary biological and behavioral measures of mental health and well-being. Future analyses on secondary outcomes, including whole-brain voxelwise analyses of gray matter volume and perfusion, but also other brain and behavior modalities, will allow us to determine the measures most sensitive to meditation practice, and investigate the mechanisms of these effects.

## Conclusions

Future secondary analyses from this trial will allow assessment of the impact of meditation on volume and perfusion throughout the whole brain and on other measures, and will examine factors (participants’ characteristics, additional outcomes, intervention doses) associated with responsiveness to the intervention.

## Notes

Supplement 1. Trial protocol and statistical analysis plan

Supplement 2. eAppendix 1. Inclusion and exclusion criteria for the Age-Well clinical trial eAppendix 2. List of assessments and corresponding outcomes eAppendix 3. Method details eAppendix 4. Details on the 15 subscales included in the global composite score of attention regulation, socio-emotional and self-knowledge capacities eAppendix 5. Results of the moderator analyses eAppendix 6. Baseline values of the composite scores by group eAppendix 7. Longitudinal changes from baseline (Pre) to 18 months (Post) in the composite scores by group eTable. Primary outcome descriptive results eReferences

Supplement 3. The Medit-Ageing Research Group nonauthor collaborators

Supplement 4. Data sharing statement.
