# Supplementary material for: Effect of an 18-Month Meditation Training on Regional Brain Volume and Perfusion in Older Adults: The Age-Well Randomized Clinical Trial
Source: JAMA Neurol. 2022 Oct 10;79(11):1165–74. doi: 10.1001/jamaneurol.2022.3185 (PMC9552046; doi:10.1001/jamaneurol.2022.3185)
Supplement: Supplement 1. — Trial protocol and statistical analysis plan [file jamaneurol-e223185-s001.pdf]

BIOMEDICAL RESEARCH PROTOCOL

Full title
INTERVENTIONAL TRIAL IN COGNITIVELY INTACT SENIORS TO EVALUATE THE EFFECTS OF MEDITATION AND FOREIGN LANGUAGE LEARNING ON BEHAVIOURAL, BIOLOGICAL AND IMAGING MEASUREMENTS

Short title: AGE-WELL

Sponsor No.	EudraCT or RCB ID[1] No.	CPP No.
C16-38	2016-002441-36 (IDRCB No.: 2016-A01767-44)	2016-33

VERSION NO. 12.0 OF 20/01/2022

CONFIDENTIAL

Sponsor:
Inserm – Address: ITMO Santé Publique - Pôle Recherche Clinique (PRC)
Biopark, Bâtiment A, 8 rue de la Croix Jarry, 75013 Paris, France

Contact:
Gabrielle DEROCLE, Inserm Project Leader
gabrielle.derocle@inserm.fr
Tel: +33 1 44 23 60 41
Fax: +33 1 44 23 61 26

Coordinating investigator (statutory):	Scientific leader[1] (statutory):
**Dr Vincent De La Sayette**<br><br>**Function: Neurologue**<br>**Inserm affiliation unit: U1077**<br><br>**Address: Département de Neurologie, CHU de Caen 14033 Caen Cedex, France**<br>**Tel: +33 2 31 06 54 90**<br>**Fax: +33 2 31 06 51 16**<br>**Email: delasayette-v@chu-caen.fr**	**Gaël, Chételat (PhD)**<br><br>**Function: Research director**<br>**Inserm affiliation unit: U1237**<br><br>**Address: GIP Cyceron Boulevard Henri Becquerel BP5229 14074 Caen cedex 5, France**<br>**Tel: +33 2 31 47 01 73**<br>**Fax: +33 2 31 47 01 06**<br>**Email: chetelat@cyceron.fr**

☒ **Monocentric study**
☐ **French multicentric study**
☐ **European multicentric study**
☐ **International multicentric study**

[1]In addition to registration with the EMA, this study can also be registered on other international registries: ClinicalTrials.gov, International Clinical Trials Registry Platform (ICTROP) of the WHO.
[2] If different from the coordinating investigator

HISTORY OF PROTOCOL VERSIONS

Part reserved to the sponsor

Short title: AGE-WELL

Version No.	Type of modification	Date
V1.0	Initial submission	08/06/2016
V2.0	Modification following CPP DIC<br>-<br>Version approved by authorities for the start of the study	28/07/2016
V3.0	Protocol amendment no.1	14/11/2016
V4.0	Protocol amendment no.1 - following ANSM requalification	14/12/2016
V5.0	Protocol amendment no.2	24/04/2017
V6.0	Protocol amendment no.3	26/06/2018
V7.0	Protocol amendment no.4	11/02/2020
V8.0	Protocol amendment no.5	08/04/2020
V9.0	Protocol amendment no.6	08/07/2020
V10.0	Protocol amendment no.7	10/12/2020
V11.0	Protocol amendment no.8 – addition of questionnaires to the substudy AGE-WELL – Lockdown	23/03/2021
V12.0	Protocol amendment no.9	07/01/2022

LIST OF ABBREVATION

Aβ	Amyloid
AD	Alzheimer's disease
AE	Adverse Event
Ag/AgCl	Silver/Silver chloride
aMRI	Anatomical Magnetic Resonance Imaging
ANSM	Agence Nationale de Sécurité du médicament et des produits de santé (National Agency for the Safety of Medicines and Health Products)
ApoE	Apolipoprotein E
ASL	Arterial Spin Labeling
ASN	Autorité de Sûreté Nucléaire (Nuclear Safety Authority)
^{18}F-AV45	Amyvid®; Radio tracer of amyloid pathology
AX-CPT	AX Continuous Performance Task
BDNF	Brain-derived neurotrophic factor
BNP	Brain Natriuretic Protein
CRB	Biological Ressources Center
CBC	Cell Blood Count
CCTP	Cahier des Clauses Techniques Particulières (Special Technical Specifications)
cDNA	Complementary deoxyribonucleid acid
CFT	Compassion-focused Therapy
CIRS	Cumulative Illness Rating Scale
CPP	Comité de Protection des Personnes (Committee for the Protection of Persons, french Ethical Committee)
CRA	Clinical research Assistant
CPS	Cellule de présélection (Preselection Unit)
CRC	Clinical Research Center
CRF	Case Report Form
CRP ultra-sensitive	ultra-sensitive C Reactive-Protein
CRU	Clinical Research Unit
DKI	Diffusional Kurtosis Imaging
DMN	Default Mode Network
DSIC	Department of Scientific Information and Communication
DTI	Diffusion Tensor Imaging
EDF	Experimental Drug File
eCRF	Electronic Case Report Form
EDTA	Ethylenediaminetetraacetic acid
EEG	Electroencephalography
Eudra CT	EudraVigilance Clinical Trial Module
Experts Med	Expert meditators
FDA	Food and Drug Administration
^{18}F-FDG	^{18}F-FluoroDeoxyGlucose
fMRI	Functional Magnetic Resonance Imaging
GCP	Good Clinical Practices
GGT	Gamma Glutamyl Transpeptidase
GWAS	Genome-Wide Association Study
Hpc	Hippocampus
HDL	High Density Lipoprotein
ICA	Independent Component Analysis
IGF-1	Insulin-like Growth Factor
ITMO	Institut Thématique Multi-Organisme (Multi-Organization Thematic Institute)
LDL	Low Density Lipoprotein
MBSR	Meditation-Based Stress Reduction
MCI	Mild Cognitive Impairment
MRI	Magnetic Resonance Imaging
mRNA	Messenger ribonuclic acid

MMN	Mismatch Negativity
NE Seniors	Non-expert seniors
NFD	Neurofibrillary Degeneration
NFL	Plasma Neurofilament Light
PAI-1	Plasminogen Activator Inhibitor-1
PBMC	Peripheral Blood Mononuclear Cells
PET	Positron Emission Tomography
PFRS	Pôle Formation en Recherche et en Santé (Research and Health Training Pole)
PI	Participant Identification
QSM	Quantitative Susceptibility Mapping
Rest-SoVT	Rest-Socioaffective Video Task
REST	Neuron-restrictive Silencer Factor
SAE	Serious Adverse Event
SCD	Subjective Cognitive Decline
SCIH	Service Commun Investigation Humaine (Common Human Investigation Service)
SDHEA	Dehydroepiandrosterone-Sulfate
SHBG	Sex Hormone Binding Globulin
SPC	Summary of Product Characteristics
SPM	Statistical Parametric Mapping
STAI-A	State-Trait Anxiety Inventory - A
SUAE	Serious Unexpected Adverse Effect
SUVR	Standardized Uptake Value Ratio
TGO	Glutamyl Pyruvate Transaminase
TGP	Glutamyl Oxaloacetate Transferase
TSH	Thyroid Stimulating Hormone
tPA	Tissue Plasminogen Activator
WP	Workpackage

LIST OF THE TABLES AND FIGURES

Table 1: Study groups
Table 2: Study populations
Table 3: Flow Chart of the Age-Well trial
Table 4: Diagnostic battery – Neuropsychological tests performed at the inclusion visit
Table 5: Neuropsychological tests proposed for the neuropsychological and behavioural assessment and performed during the different on-site testing sessions
Table 6: Self-questionnaires proposed as part of the neuropsychological and behavioural assessment and to be completed by the participants outside the on-site testing sessions
Table 7: Overall logistics of the Age-Well trial
Table 8: Details of biological markers and conditions for sample collection and analysis
Table 9: AE grading scale (severe and non-severe)

Figure 1: Increase in volume and metabolism on FDG-PET in 6 senior expert meditators (over 60 years of age) versus 67 age-matched controls who never meditated. The results of this pilot study show an increase in the cingulate cortex and insula, which is particularly important for FDG metabolism (Chételat and al., submitted)
Figure 2: Experimental design of the Age-Well trial
Figure 3: Provisional schedule of the Age-Well trial
Figure 4: Recruitment procedure
Figure 5: Practical procedure of the Age-Well trial detailing all the examinations
Figure 6: Represantation of the different conditions in the rest-SoVT task
Figure 7: MRI1 examination procedure
Figure 8: Representation of the different conditions of the AX-CPT task

Figure 9: MRI2 examination procedure
Figure 10: MRI3 examination procedure
Figure 11: PET-Amyvid® examination procedure
Figure 12: FDG-PET examination procedure

Summary of the research

**SPONSOR**	Inserm
**COORDINATING INVESTIGATOR**	**Dr Vincent De La Sayette**<br>Neurology department, CHU de Caen 14033 Caen Cedex, France
**ACRONYM AND TITLE**	**AGE-WELL**<br>**Interventional study in cognitively intact seniors to evaluate the effects of meditation and foreign language learning on behavioural, biological and imaging measurements**
**RATIONALE / BACKGROUND**	In view of the aging population, it is crucial to develop approaches that allow seniors to maintain their quality of life, preserve their physical and mental conditions and prevent cognitive decline, dementia, sleep disorders and depression, the occurrence of which increases with age.<br>This project is based on the premise that a mental training in the regulation of stress and emotions through meditation would reduce these effects and thus improve the well-being and mental health of seniors and delay Alzheimer's disease or reduce the risk of developing this disease.
**OBJECTIVES**	**Main objectives:**<br>- To estimate the effect of an 18-month meditation intervention on the volume and perfusion of the anterior cingulate cortex in comparison to an absence of intervention (passive control);<br>- To estimate the effect of an 18-month meditation intervention on the volume and perfusion of the insula compared to a 18-month language learning intervention.<br><br>**Secondary objectives:**<br>o Related to interventions:<br>- To compare the effects of the two interventions (meditation and learning a foreign language, for 18 months) with each other and with a passive control in non-meditating seniors, on behavioural and biological measurements (cognitive, psycho-affective, blood, sleep, and imaging), including measurements to assess potential deleterious effects as well as gender-specific effects (male/female);<br>- To estimate the maintenance of the interventions' effects (meditation and learning a foreign language in the long term, 35 months after the end of the intervention) on cognition, psycho-emotional factors, sleep and lifestyle (but also imaging and blood markers if budget allows).<br>- To compare the long-term effects (35 months after the end of the intervention) of the 2 interventions (meditation and learning a foreign language) on cognition, psycho-emotional factors,

	sleep and lifestyle (but also imaging and blood markers if budget allows).<br>- To estimate the effect of the interventions (meditation and learning a foreign language for 18 months) on the participant's partner (i.e., their perception of the participant's changes, their interactions with the participant, and their willingness to help the participant), compared to a passive control.<br>- To compare the long-term effects of the interventions (meditation and foreign language learning) on the participant's partner (i.e., their perception of the participant's changes, their interaction with the participant, and their willingness to help the participant), 35 months after the end of the intervention.<br>- To estimate the effect of the interventions (meditation and foreign language learning, for 18 months) (i.e. their acceptance of the intervention, their perception of changes...).<br>- To evaluate the consistency between participants' perceptions of the interventions and their effects with those of the meditation and English teachers (after the end of the interventions).<br>o Not related to the interventions:<br>- To estimate the associations between meditation expertise and behavioural and biological measurements (cognitive, psycho-emotional, life factors, blood, sleep, and imaging), and to compare expert meditators and non-meditating seniors;<br>- To identify, in senior meditators, the neural signatures of two meditative practices (mindfulness meditation and compassion-based meditation), i.e. the specific neural activity associated with each practice;<br>- To explore associations between lifestyle factors (cognitive activity, diet, and physical activity), psycho-emotional measurements, and behavioural and biological measurements (cognitive, psycho-emotional, life factors, blood, sleep, and imaging), as well as gender-specific associations (male/female);<br>- To explore the pathophysiological mechanisms of AD by studying the links between the different measurements and the influence of structural and functional connectivity on the spread of lesions, as well as the gender-specific effects (male/female) (association with data collected in the IMAP+ study);<br>- To compare the early FDG-PET perfusion measurement to the early AMYVID®-PET perfusion measurement to better understand the relationships (and potential changes in coupling) between metabolism and perfusion and compare the sensitivity of the two measurements to evaluate the effects of interventions.<br>- To identify determinants of well-being and mental health in aging in participants and their partners from cognitive data, psycho-emotional, sleep, and lifestyle measurements (as well as imaging and blood markers if budget allows) in passive control participants over the course of the trial.
**RESEARCH DESIGN**	- Monocentric, randomized, controlled trial with blinded assessment of achieved measurements, with three parallel arms comparing an 18-month meditation intervention, an 18-month foreign language learning intervention, and a passive control group (no intervention), in volunteer participants over 65 years of age with no meditative practice experience.<br>- Cross-sectional study evaluating the association between meditation practice and markers of health status, including expert meditators consecutively to compare their characteristics with those of non-expert seniors at inclusion in the randomized trial.

**INCLUSION CRITERIA**	For all participants:<br>- Age ≥ 65 years old;<br>- Be independant (assessed by the following question asked by the physician during an interview with the participant, and their partner when present: would you be able to live on your own, on a purely "functional" and not emotional level?);<br>- Live at home;<br>- Have a level of education ≥ 7 years (starting from 1rst Grade included);<br>- Be a member or beneficiary of a social security regime;<br>- Be motivated to participate effectively in the project and sign the informed consent of the protocol in agreement with the Comité de Protection des Personnes;<br>- Present normal neuropsychological performances (based on age, gender, and education level) on the diagnostic battery tests (Table 4, Section 6.4).<br>Specific criteria for non-expert seniors<br><br>Specific criteria for non-meditation experts:<br>- Native french speaker;<br>- Be available for at least the next 24 months (this criterion will be verified during the individual interviews or the question of availability for the next 24 months may be explored;<br>- Retired from occupation for at least 1 year;<br>- Have no strong preference or objection to any of the three intervention groups (assessed by a question to the participant);<br>- Not have practiced meditation or comparable practices (yoga, Qi Gong, Alexander Technique) regularly or intensively EITHER:<br>• > 1 time per week for 6 or more months in a row in the past 10 years,<br>• intensively (internship or retreat > 5 consecutive days) within the last 10 years,<br>• > 25 days of retirement accumulated before the last 10 years;<br>- Not fluent in English (assessed by asking the following question to the participant: "Are you fluent in English, can you hold a conversation with ease? fluent in English, can you hold a conversation with ease?"; only those who answered no to this question could be included in the trial).<br><br>Specific criteria for meditation experts:<br>- Have practiced at least 10,000 hours of formal meditation practice in a lifetime, including at least 6 months in retreat;<br>- Have a daily meditation practice (at least 6 days/week, at least 45 minutes per day on average);<br>- Practice mindfulness meditation (i.e. mindfulness, Samatha/ Vipassana, Zazen (Zen), Shikantaza (Zen), focused attention, Mahamudra/ Dzogchen), and compassionate/ loving-kindness meditation (i.e. tonglen practice, "four immeasurable qualities" (metta/ karuna) practices, Bodhichitta meditation).<br>These criteria will be assessed in a semi-directed interview including a detailed assessment of the type, duration and frequency of meditation during the lifetime.

	o Presence of contraindications to MRI examination (claustrophobia, ferromagnetic object in the body); or to Amyvid® and Glucotep® PET scan;<br>For safety precautions in the use of radiopharmaceuticals, a blood sample to check renal and hepatic functions but also blood sugar will be taken at the V1 visit, at the 18-month follow-up visit (V3) and at the long-term follow-up visit (V4) prior to PET imaging exams. The glomerular filtration rate will also be calculated. In case of any renal failure, hepatic failure or other biological anomaly of grade 3 or higher detected during these analyses or if the blood sugar measured is higher than 1.6g/L, the volunteer will not be able to perform the PET scans.<br>o Known hypersensitivity to Amyvid® or Glucotep®;<br>o History and presence of a known major neurological or psychiatric condition (including addiction to alcohol or psychoactive substances capable of causing strong psychic and/or physical dependence);<br>o History of brain disease (vascular, degenerative, malformative, tumor or head trauma with loss of consciousness for more than one hour);<br>o Presence of an unstabilized chronic or acute disease (respiratory, cardiovascular, digestive, renal, metabolic, hematological, endocrine or infectious);<br>o Current or recent use of medications that may interfere with cognitive or imaging measurements (psychotropic drugs, anti-histamines with anti-cholinergic action, anti-parkinsonian drugs, benzodiazepines including muscle relaxants, long-term steroidal anti-inflammatory drug, anti-epileptics, central analgesics);<br>o Be under guardianship or curatorship;<br>o Inclusion in another biomedical research protocol at the time of entry if the study includes imaging studies using radiomarkers;<br>o Physical or behavioural inabillity to comply with follow-up.<br><br>Volunteers meeting only one of the non-inclusion criteria may not be eligible to participate in the research.
**NON-INCLUSION CRITERIA**	(continued above)
**RESEARCH INTERVENTIONS**	For the two types of interventions, volunteers will have:<br>- 2-hours group lessons, once a week,<br>- Exercises to realize every day for at least 20 min,<br>- Days and/or half-days of more intense practice.<br>In each weekly group session, a time will be devoted to the presentation, another for discussion, and another one for practice. The participants will be strongly encouraged to participate in all of these activities over the course of the intervention (18 months).<br><br>**Meditation group:**<br>The intervention in the meditation group will be delivered by expert meditation teachers and will take place at the Pôle de Formations et de Recherche en Santé (PFRS), in Caen, France. This intervention will consist of an original secular meditation training program offered from a perspective of personal development and successful aging. This 18-month program is intended for healthy retired people living at home. The goal of this program is to develop mindfulness and compassion as additional psychological resources for the physical, cognitive and psychological challenges of aging.<br><br>**Foreign language learning group:**<br>The intervention in the foreign language learning group (English) will be carried out by teachers from the Carré International, a department at the

	University of Caen (France) dedicated to language learning and experienced with the older adults. This intervention will consist of English exercises aimed at reinforcing each participant's skills in terms of written and oral comprehension and expression.<br>Starting with the second wave, the foreign language learning group will be divided into two subgroups in order to respect the logistical constraints on group size. The allocation in each of the two English groups will be drawn at random, and adapted by the English teachers if necessary from the level evaluated during the English test carried out during the diagnostic battery, to limit the heterogeneity of level within the same group.<br>**Passive control group:**<br>No intervention will be offered to this group.
**JUDGING CRITERIA**	**Primary Endpoint:**<br>o <u>For the comparison between meditation group and passive control group:</u><br>- Volume difference in the anterior cingulate cortex measured on V1-weighted MRI at inclusion and at 18 months;<br>- Perfusion difference in the anterior cingulate cortex measured by PET (early measurements of the radiopharmaceutical Amyvid®) at inclusion and at 18 months.<br>o <u>For the comparison between meditation group and foreign language learning group:</u><br>- Volume difference in the insula measured on T1-weighted MRI at inclusion and at 18 months;<br>- Perfusion difference in the insula measured by PET (early measurements of the radiopharmaceutical Amyvid®) at inclusion and at 18 months.<br><br>**Secondary Endpoint:**<br>o <u>Behavioural measurements:</u><br>- A composite score by function assessed by neuropsychological tests and questionnaires.<br>o <u>Imaging measurements:</u><br>- Volume of gray matter measured in each voxel of the brain;<br>- Volume of the hippocampus and hippocampal subfields;<br>- Cerebral perfusion measured in each voxel of the brain;<br>- Fractional anisotropy and mean diffusivity in each brain voxel;<br>- Number, size and location of white matter lesions;<br>- Magnetic susceptibility measured in each voxel of the brain;<br>- Functional brain connectivity in a non-meditative state;<br>- Beta-amyloid load (of the radiopharmaceutical Amyvid®) measured in each brain voxel and global average in the grey matter;<br>- Brain glucose consumption at rest (relative to the average consumption measured in the cerebellum);<br>- Brain activity measured in fMRI specifically associated with emotional processing (comparing emotional and neutral items) from the Rest-SoVT task .<br>- Brain activity associated with attentional processes of alertness, inhibition and sustained attention processes measured in fMRI during the AX-CPT task.<br>o <u>Sleep measurements:</u><br>- Subjective sleep measurements collected by sleep questionnaires (tables 6 and 7 below);<br>- Actimetry parameters (average sleep duration, fragmentation index of activity and rest periods, regularity of activity/rest cycle);<br>- Data collected through polysomnographic recordings;<br>- Data collected with the Somno-Art device.

	o Anthropometric measurements: body fat, fat mass, lean mass, hydration mass, lean mass, hydration rate, muscle mass, bone mass, impedance mass, bone mass, impedance, basal metabolic rate, visceral fat and metabolic age, as well as Sarcopenic Index (SMI) and Skeletal Muscle Mass (SMM)<br>o Blood measurements:<br>- Cholesterol, triglycerides, HDL, LDL, urea, creatinine, GGT, TGO and TGP, blood sugar, CBC, APOE4, Peroxyredoxin, BNP, insulin, estradiol, SDHEA, cortisol, ultra-sensitive CRP, SHBG, bioavailable testosterone, TSH, IGF1, serotonin, tPA, PAI-1, cytokines, BDNF, Tau & PhosphoTau, Aβ-40 & 42, , lymphocyte immunophenotyping, telomers & telomerase, cDNA/mRNA (REST), GWAS, NeurofilamentLight (NFL), fibrinogen, GFAP.<br>o CIRS Score<br>o Impact on the participants' surroundings:<br>- Scores on questionnaires offered to a person close to the participant as "hetero-questionnaires" (see tables 5 and 6 below for list of questionnaires)<br>o Perception of the intervention and consistency between participants' and teachers' perceptions:<br>- Qualitative data from interviews with participants and teachers<br>o Neural signatures of meditative practices:<br>- Measurement of neuronal activity in each brain voxel in fMRI at rest, and during the rest-SOVT task, in a state of mindfulness meditation versus a non-meditative state;<br>- Measurement of neuronal activity in each brain voxel in fMRI at rest, and during the rest-SOVT task, in a compassionate meditative state versus a non-meditative state.<br>o Measurement of the tolerance of interventions:<br>- Occurrence of adverse events, measurements of anxiety, depression, satisfaction and well-being<br>o Assessment of the intensity of meditation and foreign language practice over the long term (i.e. up to 35 months after the end of the intervention).
**STUDY SIZE**	**Participants in the randomized clinical trial:**<br>The comparison between the meditation group and the no-intervention group is going to be on the difference in 1) volume and 2) perfusion of the anterior cingulate cortex 18 months after the start of the intervention compared to the value at inclusion, with an expected effect size of 0.75. The same effect size of 0.75 is expected for the comparison between the meditation group and the English language learning group on the difference in 1) volume and 2) perfusion of the insula 18 months after the start of the intervention compared to the inclusion value.<br><br>To show an effect size of 0.75 for each comparison, with 80% power and a two-sided first-species risk of 1.25% (Bonferroni correction), 42 participants per group must be recruited for a total of 126 participants. In order to increase the statistical power of the secondary analysis on the FDG-PET scan performed from the second wave of inclusion, 24 additional participants will be recruited, i.e. 8 additional participants per group (meditation, English, passive control) for a total of 150 participants.<br><br>**Expert meditators participants:**<br>For reasons of recruitment capacity, it was agreed to recruit 30 expert meditators into the trial.<br>The inclusion of 30 expert meditators will allow a power of 95% for comparison with the 126 non-expert participants, with a first order risk

	of 5%, to show an effect size of 0.74 in the comparison of brain imaging data.
**ANTICIPATED NUMBER OF CENTERS**	Monocentered trial
**DURATION OF THE RESEARCH**	7 years (6 years for the data collection and 1 for its analysis)
**STATISTICAL ANALYSIS OF DATA**	The main analysis will be performed on an intention-to-treat basis, comparing the primary endpoints between groups by Student's t test or Wilcoxon test if the normality of the criterion is not verified.
**EXPECTED IMPACTS**	Scientific publications; better understanding of the determinants of mental health, and the effects of both interventions on biological and psychological parameters as well as the mechanisms underlying these effects.

In the attachment to this protocol, the Age-Well - Lockdown sub-study entitled "Impact of Lockdown in the Age-Well Cohort" is described.

TABLE OF CONTENTS

1. RATIONALE FOR THE TRIAL – WORKING HYPOTHESES 15

1.1. Cognitive functions, psycho-affective measurements and lifestyle 16
1.1.1. Effects of aging and Alzheimer's disease 16
1.1.2. Effects of meditation 17
1.1.3. Lifestyle 17
1.2. Brain imaging 18
1.2.1. Effects of aging and Alzheimer's disease 18
1.2.2. Effects of meditation 19
1.2.3. PET imaging 19
1.2.4. Functional MRI (fMRI) at rest 21
1.2.5. Functional MRI (fMRI) of activation 22
1.2.6. Sleep 23
1.2.7. Blood biomarkers 24
1.2.8. Effect of gender 25
1.2.9. Electrophysiological markers of activation and rest by electroencephalography (EEG) 25

2. OBJECTIVES 26

2.1. Main objectives 27
2.2. Secondary objectives 27
2.2.1. Secondary objectives in relation to the study interventions 27
2.2.2. More exploratory secondary objectives (not related to the interventions) 28

3. TRIAL TYPOLOGY AND EXPERIMENTAL DESIGN 29

3.1. Trial type 29
3.2. Statement of the experimental design 29
3.3. Provisional schedule of the trial (Flow-Chart) 30

4. SELECTION OF PARTICIPANTS 32

4.1. Study populations 32
4.2. Eligibility criteria 32
4.2.1. Inclusion criteria 32
4.2.2. Non-inclusion criteria 33
4.3. Recruitment method 34

5. EVALUATION CRITERIA 37

5.1. Main evaluation criteria 37
5.2. Secondary evaluation criteria 37
5.3. Methods and timelines for measuring, collecting, analyzing, and reporting on the outcomes of the trial (efficiency or other criteria) 38
5.4. Compliance monitoring criteria 38

6. PRACTICAL REALIZATION OF THE PROTOCOL 40

6.1. Investigating center 40
6.2. Follow-up schedule of the participants 41
6.3. Recruitement and screening 42
6.4. Visit no.0 – Screening visit 42
6.5. Participant Identification (PI) in the protocol 44
6.6. Visit no.1 – Inclusion visit 44
6.7. Randomization (Non-expert seniors only) 61
6.8. 18-month intervention 62
6.8.1. « Meditation » intervention 63
6.8.2. « English learning » intervention 64
6.9. Visit no.2 - Intermediate evaluation visit at 9 months 65
6.10. Visit no.3 – Follow-up visit 65
6.11. Visit no.4 – Follow-up visit 29±6 months post-intervention 66
6.12. Significant health outcomes (information, follow-up, treatment) 68
6.13. Termination of participation in the trial 68
6.14. Premature termination of the trial 68

6.15. Deviations from the protocol 69
6.15.1. Premature and permanent cessation of research treatment 69
6.15.2. Lost to follow-up participant 69
6.15.3. Incorrectly included participant 69
6.16. Rules for the discontinuation of the trial 70
6.16.1. Description of the rules for permanent or temporary cessation of part or all of the trial 70
6.16.2. Defining the end of trial on individuals 70

7. DESCRIPTION OF THE LOGISTIC ORGANIZATION OF THE TRIAL 70

7.1. General trial logistics 70
7.2. Experimental products 72
7.2.1. Name and description of the product / medical device 72
7.2.2. Terms of use 73
7.2.3. Treatments authorized during the trial 73
7.2.4. Unauthorized treatments during the trial 73
7.2.5. Logistic circuit 73
7.3. Collection of blood samples of human origin 76
7.3.1. Nature of the samples 76
7.3.2. Sampling conditions 76
7.3.3. Coding and labelling procedures 77
7.3.4. Sample processing procedures 77
7.3.5. Transport conditions 80
7.3.6. Storage conditions 80
7.3.7. Description of secure storage conditions 81
7.3.8. Sample management procedures 81
7.3.9. Computerized management of sample data 81
7.3.10. Conditions of traceability 81
7.3.11. Description of the collection's quality system 82

8. VIGILANCE OF THE TRIAL 83

8.1. Definitions 83
8.2. Adverse Event (AE) 83
8.2.1. Adverse event 83
8.2.2. Serious adverse event (SAE) 83
8.2.3. Serious Unexpected Adverse Effect (SUAE) 84
8.2.4. New safety event 84
8.3. Investigator's responsibilities 84
8.3.1. Notification of AEs to the sponsor 84
8.3.2. Notification of the SAEs to the sponsor 84
8.3.3. What should be declared? 84
8.3.4. Evaluation of AEs 86
8.3.5. Reporting of incidents or accidents in the field of radiation protection 87
8.3.6. Potentiel risks of the intervention and trial, and what to do in case of an adverse event 87
8.4. Responsibilities of the sponsor 87
8.4.1. Recording and evaluation of SAEs 88
8.4.2. Declaration of safety data to the competent national authorities and the ethics committee 88

9. TRIAL MONITORING 89

9.1. Scientific Council 89
9.2. Independant Supervisory Committee 90

10. DATA COLLECTION AND PROCESSING 90

10.1. Description of the collected data 90
10.1.2. Data collected during the inclusion visit (V1) 91
10.1.3. Data collected during the intervention 93
10.1.4. Data collected at the 9-month follow-up visit (V2) 94
10.1.5. Data Collected at the 18-Month End-of-Intervention Visit (V3) 94
10.2. Data collected at the 29±6 (35) month post-procedure follow-up visit (V4) 94
10.3. Definition of source data 95
10.4. Data circuit before computer entry 95
10.5. Computerized data entry 96
10.6. Description of data processing, verification and validation methods (data management) 98
10.6.1. Processing of eCRF data 98
10.6.2. Preprocessing of the imaging data 99

10.6.3. Verification of imaging data ... *102*
10.7. Management and archiving conditions of the trial documents ... 102

11. STATISTICAL ANALYSIS OF DATA ... 103

11.1. Responsibility for statistical analysis ... 103
11.2. Calculation of the study size ... 103
11.3. Description of the statistical analysis plan ... 104
11.3.1. Analysis strategy ... *104*
11.3.2. Descriptive statistical methods ... *105*
11.3.3. Participants included in the analysis ... *105*
11.3.4. Characteristics of the participants ... *105*
11.3.5. Analysis of the main criteria ... *106*
11.3.6. Analysis of the secondary criteria ... *106*
11.3.7. Other analysis ... *107*

12. COMMUNICATION ... 108

12.1. Terms and conditions for the publication of results ... 108
12.2. Procedures for writing the final report ... 108
12.3. Procedures for informing trial participants about the overall results of the research ... 109
12.4. Procedures for informing individuals about health data during and after research ... 109
12.5. Terms and conditions relating to press communication ... 109

13. CONFIDENTIALITY ... 109

13.1. Terms of confidentiality with respect to the individuals ... 109
13.2. Terms of confidentiality with respect to the trial ... 109

14. PROTECTION OF PERSONS ... 110

14.1. Ethical rationale of the protocol ... 110
14.2. Ethical and regulatory provisions ... 111
14.3. Comité de Protection des Personnes (CPP) ... 112
14.4. Insurance and funding ... 112
14.5. Procedures for obtaining consent from individuals ... 112

15. QUALITY ... 114

15.1. Description ... 114
15.2. Monitoring (quality control of the trial) ... 114

16. SUBSTANTIAL AMENDMENTS TO THE PROTOCOL ... 114

16.1. Sponsor's procedure for substantial changes ... 114

17. BIBLIOGRAPHY ... 115

ANNEXES ... 129

17.1. Protocol synopsis ... **Error! Bookmark not defined.**
17.2. Information and consent forms ... **Error! Bookmark not defined.**
17.3. Authorization of location ... **Error! Bookmark not defined.**
17.4. ASN authorization to hold and use radionuclides in nuclear medicine ... **Error! Bookmark not defined.**
17.5. List of investigators and research site ... **Error! Bookmark not defined.**
17.6. Copy of insurance certificate ... **Error! Bookmark not defined.**
17.7. Copy of the CPP's favorable opinion ... **Error! Bookmark not defined.**
17.8. Copy of the ANSM authorization ... **Error! Bookmark not defined.**
17.9. Protocol annex: Age-Well Lockdown substudy entitle "Impact of Lockdown in the Age-Well Cohort" ... 129

1. RATIONALE FOR THE TRIAL – WORKING HYPOTHESES

The number of people over the age of 65 (referred to as seniors in the following) is steadily increasing in Europe and is expected to rise from 86 million (18% of the population) to about 120 million (24%) in 2040 (Jané-Llopis and Gabilondo, 2008). As the incidence of health problems increases with age, this growth in the number of older people represents a major social and economic challenge for European societies. For example, more than 50% of older adults have sleep problems (Ohayon and Vecchierini, 2005), 10 to 15% suffer from depression (Barua et al., 2011), 7 to 10% develop dementia (Jané-Llopis and Gabilondo, 2008). These health problems are often chronic, require long-term care, and have a significant impact on the quality of life of the individuals, those around them, and on the society in which they live. In addition, these problems are increased by stress and negative emotions, interact with each other, and increase the risk of Alzheimer's disease (AD) (Norton et al., 2014). Numerous studies have shown the deleterious effect of stress on the hippocampus (Ricci et al., 2012), a brain structure important for memory and vulnerable during aging and implicated in AD mechanisms. Sleep disturbances could also accelerate the pathological process of AD (Ju et al., 2013) and each depressive symptom increases the risk of dementia by approximately 20% (Wilson et al., 2002).

This project is based on the premise **that mental training in stress and emotion regulation through meditation could reduce these effects and thus improve the well-being and mental health of seniors and delay AD or reduce the risk of AD**. Although research in this field is still recent, studies have shown encouraging effects in seniors. Notably, meditation is reported to be associated with improved cognitive abilities (mainly attention but also memory, which are the two most age-sensitive functions and the most frequently impaired in AD) (Gard et al., 2014; Marciniak et al., 2014; Newberg et al., 2014). Other studies have shown that meditation is also thought to be associated with reduced stress, anxiety, depression, insomnia, feelings of loneliness and social exclusion (Chen et al., 2012; Innes and Selfe, 2014), and cardiovascular risk (Schneider et al., 2012). Furthermore, related to the notion of neuroplasticity, neuroimaging studies have shown that meditation is not only associated with a change in brain activity pattern during practice but may also produce long-term structural and functional brain changes, particularly in frontal and limbic areas (Newberg et al., 2014; Slagter et al., 2011) (Fox et al., 2014; Marciniak et al., 2014; Tang et al., 2015) -known to be susceptible to aging and AD. Finally, recent studies have shown that meditation may increase telomerase activity that impacts aging and AD (Schutte and Malouff, 2014). However, these results need to be confirmed, strengthened, and refined; indeed, the populations studied are very heterogeneous, the size of the studies remains small, and very few studies include a comparison to another type of active intervention. In this trial, therefore, we propose to include an active intervention and to use a randomized allocation to control for potential selection and confounding biases in order to preserve the validity of the comparisons. We chose to use an active foreign language learning intervention (English) because 1) like meditation, this learning corresponds to mental training; 2) its organization can structurally resemble the meditation intervention (group sessions, daily practice with audio support, etc.); 3) a positive effect can be expected because foreign language learning is known to have a positive impact on cognitive functions and brain

structures sensitive to aging and AD (Mårtensson, J. et al., 2012). Finally, in addition to being useful as an active intervention for demonstrating specific effects, this intervention has its own interest because foreign language learning in older adults has never been studied within a randomized trial in older adults. Unlike the meditation intervention, foreign language learning is not known to have an impact on emotion regulation. The outcome measurements will be chosen to compare the relative impact of the two interventions based on a priori hypotheses (some related to these emotional factors, others not).

Understanding of the neurocognitive mechanisms of meditation is still limited. Meditation can be conceptualized as "a set of complex emotional and attentional regulation strategies developed for various purposes including the development of well-being and emotional balance" (Lutz et al., 2008). **Affective (emotional) and cognitive (attentional) control** are therefore the most likely mechanisms by which meditation could impact aging and AD. Specifically, meditation could enhance the control role of the midbrain structures and the executive network on structures involved in memory, emotion and immune system regulation. This would lead to better emotional and cognitive control, which in turn would be associated with improved mental and physical health.

The overall aim of the present trial is to assess the effects of a mindfulness-compassion meditation practice on the well-being and mental health of older adults and to investigate the attentional and emotional mechanisms involved in these effects. To address these objectives, we will use two complementary approaches: 1) we will study the effects of a meditation intervention (versus no intervention and versus another intervention) over 18 months in non-meditating seniors and 2) we will compare data from expert meditating seniors to data from non-meditating seniors before the intervention.

To measure well-being and mental health, we will perform a series of behavioural, psycho-emotional, biological and neuroimaging measurements in non-meditating seniors before and after the intervention as well as in long-time senior meditators (so-called experts). In these same expert meditators, the brain activity associated with specific meditative practices (mindfulness versus compassion) will be measured in order to obtain the "neural signature" of these two meditative practices. These neural signatures will allow us to better understand the mechanisms underlying the effects measured in the 18-month intervention. Both groups (non-expert seniors and expert meditators) will be included in the same study because of the close relationship between the analyses performed on one and the other group.

1.1. *Cognitive functions, psycho-affective measurements and lifestyle*

1.1.1. Effects of aging and Alzheimer's disease

Cognitive decline is one of the biggest concerns for the older adults. Cognitive abilities such as memory, attention and executive functions are affected early in the aging process (Craik & Salthouse, 2008; Deary et al., 2009). A decline in these domains can create difficulties in daily life and is a risk factor for developing AD (Jessen et al., 2014; Landau et al., 2010). AD is characterized by greater

cognitive impairments than those associated with so-called "normal" aging, affecting a variety of cognitive functions. Episodic memory deficits are the first to be identified by these patients, and are most often the focus of their complaint and the concern that leads them to consult. Contrary to normal aging where memory deficits are generally compensated by indexing, indicating that they reflect more a decline in retrieval (probably related to the decline in executive functions), AD is characterized by so-called "authentic" disorders that affect the encoding of information in memory (Eustache et al., 2006). In AD, delayed recall is particularly sensitive, as is autobiographical memory (Eustache et al., 2006). In addition to episodic memory tests, verbal fluency tasks appear to be particularly effective in detecting AD in the earliest stages. Thus, in patients with mild cognitive deficits (called MCI for Mild Cognitive Impairment), performance in verbal fluency and autobiographical memory can predict progression to AD (Chételat et al., 2005). Executive functions (flexibility, inhibition) are also affected early in AD (Salmon and Bondi, 2009).

1.1.2. Effects of meditation

The study of the potential long-term effects of meditation on cognition is an emerging field. Although recent, initial results have shown significant changes with attention, executive functions and intelligence in meditating experts compared to non-meditators (Gard, Taquet, et al., 2014; Prakash et al., 2010; Prakash et al., 2012; Wenk-Sormaz, 2005). The effect of mindfulness meditation on cognitive performance in older adults remains relatively unexplored. Preliminary research reports that meditation improves cognitive abilities in older adults. For example, a recent meta-analysis shows that studies in older adults (of which only 6 are randomized clinical trials) report an improvement in memory performance, attention and executive functions (Gard, Holzel, & Lazar, 2014; Marciniak et al., 2014). In addition, a study with a mindfulness meditation in older adults with subjective cognitive decline (SCD - Subjective Cognitive Decline) also shows an improvement in memory and executive function, as well as a reduction in worry (Lenze et al., 2014). Several works have also shown a positive effect of meditation on other psycho-emotional factors such as stress, anxiety, depression, and feelings of loneliness and social exclusion (Chen et al., 2012; Innes and Selfe, 2014). Despite promising results, a number of limitations exist: the number of studies remains limited, the study populations are highly heterogeneous, the study sizes remain small, and very few studies include an appropriate comparison design to another intervention. Moreover, all these studies use relatively short meditation programs (typically the standardized MBSR program for Mindfulness-based stress reduction lasting 8 weeks). Longer programs, in the context of randomized clinical trials, are therefore necessary.

The present trial proposes to evaluate the effects of meditation in the longer term (through an 18-month program offered to non-expert seniors, and the study of expert senior meditators) on cognitive and psycho-emotional functions, with a particular focus on those functions that are most sensitive to aging, AD and meditation, such as emotional, memory, attentional and executive functions.

1.1.3. Lifestyle

Various lifestyle characteristics have been widely demonstrated in the literature to have significant and long-term effects on "brain health". Notably, epidemiological studies have shown that

lifestyle has a major effect on cognition in aging and on AD risk (Norton et al., 2014). For example, these studies show that factors, such as physical activity, cognitively stimulating activities, diet, and education level are related to cognitive preservation or decline in older adults, and also to the risk of developing dementia, and specifically AD. **In order to better understand the impact of lifestyle on markers of aging and AD, and to assess whether the effects of meditation could be related to lifestyle changes, different lifestyle measurements will be performed in this study.**

1.2. *Brain imaging*

In this study, we chose the neuroimaging techniques most suitable for our purposes. This trial aims to assess the effects of long-term meditation on the well-being and mental health of older adults and to investigate the underlying mechanisms. We therefore chose imaging measurements that are particularly sensitive to aging, Alzheimer's disease and/or meditation, as well as techniques that allow us to study the mechanisms of action of meditation and more specifically the attentional and emotional components.

1.2.1. Effects of aging and Alzheimer’s disease

Aging is characterized by a decrease in brain volume and metabolism/perfusion, especially in the frontal, anterior cingulate, parietal, frontotemporal and insula regions (Kalpouzos et al., 2009). During aging, white matter lesions are also observed on FLAIR and T2 sequences and may play a role in AD (Barkhof and Scheltens, 2002; Hentschel et al., 2007). Diffusion tensor imaging (DTI) also measures microscopic white matter and gray matter lesions, and is particularly sensitive to normal and pathological aging, in a manner complementary to other evoked Magnetic Resonance Imaging (MRI) measurements (Chételat et al., 2013).

AD is also accompanied by characteristic brain alterations, described in a relatively consensual manner in the literature, especially recent reviews on imaging markers (Teipel et al., 2015; Villemagne and Chételat, 2016). In relation to memory impairment, the hippocampal region is the earliest affected by atrophy measured in vivo using Anatomical Magnetic Resonance Imaging (AMRI). The atrophy also affects the temporal neocortex. At the functional level, the damage, measured by Positron Emission Tomography coupled to ^{18}fluoro-2-deoxy-D-glucose (FDG-PET), single photon emission tomography, or functional Magnetic Resonance Imaging (fMRI), is the earliest and most marked in the posterior cingulate and temporo-parietal regions, then in the frontal cortex (especially the medial and anterior cingulate cortex). At the molecular level, AD is characterized by neurofibrillary degeneration (NFD) composed of abnormally phosphorylated tau proteins and by the accumulation of β-amyloid protein in the form of amyloid plaques or senile plaques. These lesions are now observable in vivo thanks to molecular imaging techniques combining PET and radiopharmaceuticals such as Amyvid® (^{18}F-AV45; for amyloid deposits). All these changes (atrophy, hypometabolism/hypoperfusion, amyloid deposits and DNF) occur very early in the course of the disease and can be visualized even before the onset of symptoms, at a so-called asymptomatic stage (in older adults without cognitive deficits).

1.2.2. Effects of meditation

Consistent with the principle of neuroplasticity that the brain, more than any other organ in the human body, can be subject to lasting changes in response to experience and environment, the intentional deployment of mental strategies can induce lasting plastic changes in the brain, which could have a beneficial impact on health.

For example, research has shown that meditation is associated with brain changes that persist beyond the period of meditative practice. Neuroimaging studies suggest not only that specific brain regions are activated during meditation, but also that some changes may persist over the longer term and be observed in tasks that are not directly related to meditation. During meditation, changes are generally observed in regions involved in attention such as the anterior cingulate and dorsal prefrontal cortex, as well as in regions involved in emotion control and memory functions such as the insula and the limbic system including the hippocampus and amygdala (Brefczynski-Lewis et al., 2007; Newberg et al., 2014). Changes in the fronto-parietal cortex and thalamus are also observed during meditation. Long-term effects are reported in similar regions, and involve cortical thickness, volume, gray matter density in the prefrontal, anterior cingulate, insula, and amygdala cortex (Hölzel et al., 2008a, 2010; Lazar et al., 2005), but also in some studies the inferior temporal, posterior cingulate, and temporo-parietal cortex (Hölzel et al., 2008b). The hippocampus is also sometimes reported as one of the altered structures in meditators (Hölzel et al., 2008b, 2011; Luders et al., 2013). Very few studies have been conducted in functional imaging but the results point in the same direction. For example, one study showed increased brain perfusion in the anterior cingulate and prefrontal cortex and subcortical ganglia (Newberg et al., 2010).

Given these results, it seems interesting to evaluate the effects of meditation on multiple brain regions for different reasons (regions sensitive to aging, AD, meditation, and/or attentional and/or emotional mechanisms). These issues will therefore be evaluated as secondary objectives via whole-brain analyses. However, to address our primary objective (to assess the effects of meditation in brain aging), a more focused analysis based on a priori hypothesis is crucial. The anterior cingulate cortex seems to be the most appropriate region to assess the effects of meditation in aging as it is a region that is both i) impaired in normal and pathological aging (Baron et al., 2001; Kalpouzos et al., 2009; La Joie et al., 2012); ii) known to be involved in maintaining cognitive abilities in older adults (Gefen et al., 2015; Rosano et al., 2012), and iii) one of the most sensitive to the effects of meditation (Fox et al., 2014; Tang et al., 2015). We decided to measure both volume and metabolism to combine: i) a measure on which the effects of meditation are well documented (volume) and ii) a measure considered to be more sensitive to the effects of age and AD but which has never been used in meditation studies (perfusion).

1.2.3. PET imaging

1.2.3.1. PET imaging with Amyvid® to measure amyloid deposits

Over the last decade, several radioligands more or less specific to amyloid plaques and labeled with radioactive isotopes have been used in human PET scans in AD. Studies have shown: 1) a higher average binding of these ligands in AD patients compared to healthy older adults, in particular in the

regions known to be the most affected by amyloid deposits in AD (Villemagne et al, 2008; Villemagne and Chételat, 2016); 2) an increase that would occur progressively from the asymptomatic stage, since 20 to 30% of older adults without cognitive deficits would have a level of amyloid deposits equivalent to that found in AD patients (Jansen et al., 2015; Villemagne et al., 2011). Among the different radioligands developed, fluorine-18 (^{18}F) tracers seem to be more suitable because they have a half-life of 110 min, longer than carbonaceous tracers whose half-life of 20 min considerably reduces accessibility and clinical use. Among the different fluorinated tracers developed (Herholz and Ebmeier, 2011), Amyvid® (^{18}F-AV45 or Florbetapir) has a high affinity to amyloid plaques (Choi et al., 2009), and its pharmacokinetic properties (Lin et al., 2010) make it a promising tracer. Clinical studies have confirmed the feasibility and interest of this marker by showing the good tolerance of the product by the participants and the capacity of the measurement of Amyvid® binding to discriminate AD patients from controls (Wong et al., 2010). A multicenter phase III study combining ante mortem PET-Amyvid® and neuropathological examination confirmed the interest and validity of this marker (Clark et al., 2011). Finally, our experience with this marker in the laboratory will be an asset for this study.

1.2.3.2. PET imaging with ^{18}F-FDG at rest

FDG-PET is highly sensitive for measuring changes related to normal and pathological aging. Thus, during aging, glucose metabolism progressively decreases, predominantly in the prefrontal, anterior cingulate cortex and insula (Chételat et al., 2013; Kalpouzos et al., 2009). During Alzheimer's disease, glucose depletion mainly involves the temporo-parietal and posterior cingulate cortex, and these changes appear as early as the pre-dementia stage in healthy subjects without cognitive deficits but with a genetic risk factor or a memory complaint (Scheef et al., 2012;Reiman et al., 1996). Temporo-parietal and posterior cingulate hypometabolism are among the three neuroimaging biomarkers considered in the new criteria for pre-dementia research (Sperling et al., 2011; Jack et al., 2011; Albert et al., 2011). To date, there is no PET study in the context of meditation. However, we conducted a pilot study in 6 meditating experts over 60 years of age that we compared to a database of 67 age-matched controls who had never meditated. Interestingly, we show that expert meditators show less loss of grey matter volume and metabolism in FDG-PET, in regions such as the anterior cingulate cortex and the insula (see Figure 1 below). These results are particularly interesting in the context of the present study as they concern regions i) involved in attentional and emotional processes (Menon et al., 2010; Brassen et al., 2011); ii) activated during meditative states (Tang et al., 2015); and particularly sensitive to the effects of age (Chételat et al., 2013; Kalpouzos et al., 2009). Furthermore, this study shows that the effects are even stronger for metabolism than for volume, suggesting that FDG-PET is particularly sensitive to the effects of meditation. This pilot study has just been submitted for publication.

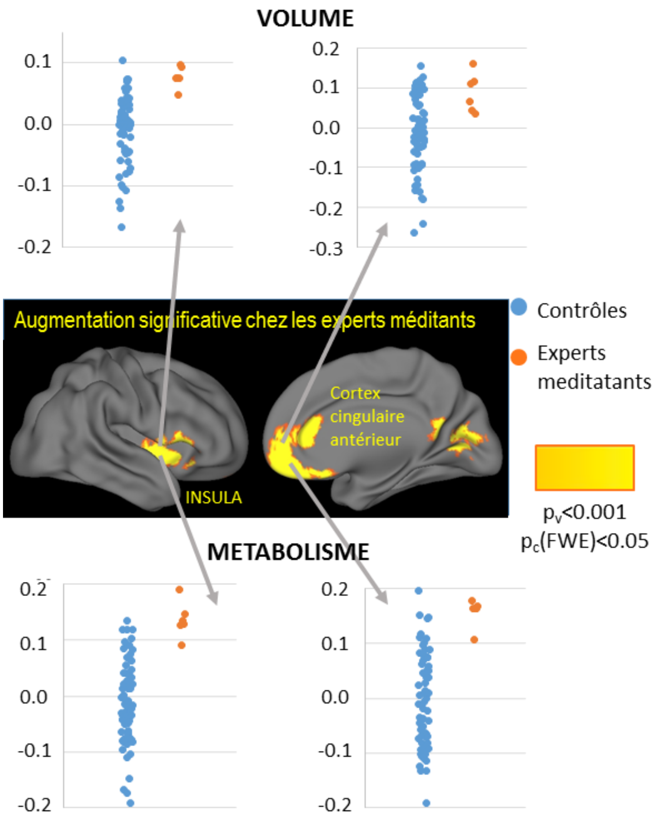

Figure 1: Increase in volume and metabolism on FDG-PET in 6 senior expert meditators (over 60 years of age) versus 67 age-matched controls who never meditated. The results of this pilot study show an increase in the cingulate cortex and insula, particularly important for FDG metabolism (Chételat et al., submitted).

1.2.3.3. Early PET-Amyvid® imaging to measure perfusion

In addition to the acquisition of PET images 50 to 70 min post-injection of Amyvid® to measure amyloid load, it is possible to acquire early images (0-10 min post-injection) to measure cerebral perfusion. Indeed, the cerebral distribution of Amyvid® within 10 min of injection has recently been shown to reflect cerebral perfusion, which in turn is coupled to cerebral metabolism (Hsiao et al., 2012; Lin et al., 2016). The considerable advantage of this measurement is that with a single injection of the radiopharmaceutical, we can access both brain perfusion (early acquisition) and amyloid plaque labeling (late acquisition). The early perfusion measurement represents our main evaluation criterion, and can be compared to the FDG-PET measurement to better understand the links (and possible changes in coupling) between metabolism and perfusion and to compare the sensitivity of the two measurements to assess the effects of interventions.

1.2.4. Functional MRI (fMRI) at rest

Resting fMRI is a particularly promising investigation method adapted to our problem. Indeed, the measurement of brain activity at rest in fMRI in young participants without brain damage has shown the existence of a "default mode network" (DMN) which would underlie auto-generated activities related to memory processes (especially autobiographical memory) and self-perception. Interestingly, this network is particularly close to the pattern of hypometabolism highlighted in AD, involving notably the posterior cingulate, anterior ventral, medial frontal, and inferior parietal cortex (Damoiseaux et al., 2006;

Greicius et al., 2004; Lustig et al., 2003), as well as the medial and lateral temporal structures in some studies (Fox et al., 2015; Greicius et al., 2004). In addition, altered DMN has been demonstrated in AD, particularly in regions altered on FDG-PET and/or anatomical MRI, such as the posterior cingulate cortex, lateral parietal cortex, medial frontal cortex, and hippocampus (Celone et al., 2006; Greicius et al., 2004; Lustig et al., 2003). DMN changes have also been reported at a pre-dementia stage of AD, in participants carrying the ε4 allele of apolipoprotein E (APOE4) or in MCI patients (Celone et al., 2006; Mevel et al., 2010). Furthermore, as discussed above, several studies have shown changes in DMN with meditation. For example, resting-state fMRI studies suggest increased connectivity between the anterior cingulate, posterior cingulate, and dorsolateral prefrontal cortices involved in cognitive and self-control in expert meditators (Brewer et al., 2011).

Resting-state fMRI is therefore relevant not only for assessing the effects of meditation in normal and pathological aging but also for trying to better understand the mechanisms underlying these effects.

1.2.5. Functional MRI (fMRI) of activation

Affective (emotional) and cognitive (attentional) control are the most likely mechanisms by which meditation could have an impact on aging and AD. The fMRI tasks used in this study aim to highlight these mechanisms and better understand their respective roles.

The evidence for the positive effects of meditation on attentional functioning has been previously shown, at the behavioural level, by an improvement in attentional abilities (see for example, Chiesa et al., 2011). Indirectly, this positive effect has also been shown through structural changes within a large fronto-parietal network (including the anterior cingulate cortex) that is known to be involved in attentional functioning (for a review, Newberg et al., 2014). However, few studies to date have directly assessed whether attentional task performance is associated with changes in activity in this network following meditative practice (Teper & Inzlicht, 2013; Dickenson, Berkman, Arch, & Lieberman, 2013; Hasenkamp, Wilson-Mendenhall, Duncan, & Barsalou, 2012). While the results of these studies are broadly consistent with this hypothesis, one criticism that can generally be leveled at them is that they use very global measurements of attentional functioning. Therefore, the objective of our fMRI activation protocol is to determine precisely the changes in brain activity for different attentional processes following 18 months of meditative practice and also in expert meditators. To this end, the AX-CPT (AX Continuous Performance Task) test initially developed by Braver et al. (2001) will be adapted to an fMRI administration. The interest of this task is that it allows us to evaluate, during the same session, the attentional capacities of phasic alertness, inhibition and sustained attention (for this last more exploratory measure, by comparing the brain activity for the different types of items between the first and second part of the test). The use of this task will also allow us to have an estimate of the compensation capacities of our participants, by comparing the performance and brain activity for "easy" and "difficult" blocks within the task (absence vs. presence of distractors). In addition to its characteristics allowing us to relate different aspects of attentional functioning to changes in brain activity, the interest of this task is that it is sensitive to the cognitive effects of normal aging (Braver et al., 2001) and AD (Braver et al., 2005). Furthermore, a comparison of brain activity of healthy young and older participants during the performance of this task (Paxton et al., 2008) highlights a fronto-parietal network, some

regions of which have been previously described as associated with meditative practice. **The use of this task thus appears to be a judicious choice in order to explore the attentional modifications that could occur, both at the cognitive and cerebral levels, following a meditative practice in an older adults' population, and the contribution of these modifications to the effects of meditation on behavioural and biological measurements of well-being and mental health in seniors.**

In addition, coping with difficult life events and situations, such as being confronted with the suffering of others, can be particularly challenging and requires special resources to overcome these trials. Research in young adults with the Socio-affective Video Task (SoVT) showed very encouraging results suggesting that adaptive emotional responses to stressful events were enhanced by compassionate meditation training (Klimecki, Leiberg, Lamm, & Singer, 2013). These changes were accompanied by increased activity in brain areas related to feelings of social inclusion and positive emotions, specifically the medial orbitofrontal cortex and striatum (Klimecki, Leiberg, Lamm, & Singer, 2013; Klimecki, Leiberg, Lamm, & Singer, 2014). Furthermore, meditation training would reduce negative feelings and increase positive feelings in response to SoVT (Klimecki, Leiberg, Lamm, & Singer, 2014). Although these studies highlight the potential of meditation training to alter emotional response and their neural underpinnings in young adults, it remains to be determined whether such effects could be found in older adults. Beyond an effect on the regulation of emotional response, meditation could also allow for a more rapid and efficient restoration of homeostatic levels after exposure to a stressful situation. Using recent results from fMRI recordings during a rest period following a stressful event (Eryilmaz, Van De Ville, Schwartz, & Vuilleumier, 2014), it has been shown that **rapid recovery from an intense emotional episode is important for well-being. In this trial, we therefore plan to use SoVT combined with fMRI recording during, and after, emotionally charged videos to better understand the contribution of compassion training and the role of emotion regulation in the effects of meditation on health and well-being in older adults.**

1.2.6. Sleep

Sleep disorders represent an important public health problem and are age-related since they affect about 20% of seniors (13% of men and 26% of women) and increase significantly with age (from about 12% between 50 and 59 years of age to 23% between 80 and 89 years of age (April 2005 report from Health, Aging and Retirement in Europe)). Epidemiological studies even report that sleep disorders could affect up to 50% of seniors (Ohayon 2005; Sleep). Sleep disorders are also common in AD patients. They are described very early in the disease and can affect up to 60% of MCI patients (Beaulieu-Bonneau and Hudon, 2009). They are also more marked in APOE4 carriers (Hita-Yañez et al., 2013). Sleep disorders have a deleterious impact on the cognitive functioning of patients (Bonanni et al., 2005; Rauchs et al., 2008; Hot et al., 2011; Westerberg et al., 2012), but in recent years, there have been several indications that these disorders may represent a risk factor for AD and exacerbate the formation of amyloid deposits (Kang et al., 2009). In particular, it has been shown in humans that sleep deprivation prevents the reduction in Aβ levels classically observed during the night (Ooms et al., 2014), highlighting the potential deleterious effects of chronic sleep deprivation over long periods. In a recent study, Spira et al. (2013) linked subjective measurements of sleep, obtained via a questionnaire,

to a quantification of amyloid load performed in vivo using PET. The authors found greater amyloid deposition in healthy older adults who slept less than 6 hours per night over the past month compared to those who slept more than 7 hours. Poor sleep quality may therefore have a deleterious effect on cognitive functioning and promote the formation of amyloid deposits. However, further studies using both subjective and objective measurements of sleep are needed to better understand the links between sleep disturbance and amyloid pathology in AD.

Sleep is particularly sensitive to psycho-affective factors such as stress, anxiety and depression. Studies have shown that stress and anxiety can cause or contribute to sleep complaints. In this respect, meditation could help preserve good quality sleep by allowing better management of stress and emotions. Initial studies suggest an improvement in sleep quality and/or duration after an 8-week meditation intervention (Winbush 2007). However, conflicting results have also been reported and there are no studies that have evaluated the effects of longer training on sleep. Furthermore, this issue is particularly relevant in an older population where sleep disturbances are more prevalent (see above). Sleep is considered to be a favorable period for the consolidation (or storage) of memories in long-term memory (Rauchs et al., 2005). This mechanism is thought to operate mainly during deep slow wave sleep phases, and involves a dialogue between the hippocampus and different neocortical areas, including the ventromedial prefrontal cortex (Rasch & Born, 2013). The various sleep changes observed during aging, associated with anatomo-functional changes, are likely to disrupt this mechanism (Harand et al., 2012). We will therefore explore the consolidation of visuo-spatial learning during sleep.

In this trial, we will measure sleep quality using complementary objective and subjective measurements not only to better understand the role of sleep in normal versus pathological aging, but also to assess the effects of meditation on sleep (as important factors in well-being and mental health in aging).

1.2.7. Blood biomarkers

Beyond neuropsychological and neuroimaging markers, blood-based biomarkers are the subject of intense research because of the accessibility advantages of a blood test. The study of meditators via biochemical and hormonal assays has revealed a number of neurochemical, hormonal and epigenetic changes. Changes in plasma serotonin and cortisol, correlated with neurophysiological changes, have been observed and are thought to follow the frontal activation related to the attentional mobilization required by meditation techniques (Newberg & Iversen, 2003; Yu et al., 2011). Hormonal changes are also numerous: modification of the corticotropic axis, decrease in TSH... (Elias & Wilson, 1995). The effects of meditation on the immune system have highlighted its down-regulatory role via the hormonal pathway mainly (release of CRF, decrease in cortisol...) (Downing & Miyan, 2000) but also via the brain effects of cytokines (Pace et al., 2009; Rosenkranz et al. 2016). Finally a meditative practice would contribute to increase the activity of telomerases, responsible for telomere length which is a predictor of immune cell longevity (Jacobs et al., 2011; Kaliman et al., 2014). This still recent field of inquiry has paid very little attention to the influence of meditative expertise on a large number of biological markers that we wish to study in this protocol. Measurements of i) general health such as blood count, urea, creatinine, gamma-glutamyl transferase, aspartame aminotransferase and alanine

aminotransferase, glycemia, insulin and fibrinogen, triglyceride-cholesterol, HDL and LDL, bioavailable testosterone, estradiol and Sex Hormone Binding Globulin (SHBG), Dehydroepiandrosterone-Sulfate (SDHEA), Thyroid Stimulating Hormone (TSH) and Brain Natriuretic Protein (BNP), ii) stress and inflammation such as ultra-sensitive C Reactive Protein (CRP), cytokines, cortisol, insulin growth factor 1 (IGF-1), peroxyredoxin, iii) aging and dementia (AD) such as telomere length, telomerase activity, lymphocyte senescence (exploration of cellular immunity via B, T, and NK cells), Brain-Derived Neurotrophic Factor (BDNF), GWAS genotyping (specifically APOE), Aβ 1-40 and 1-42, Tau/phoshoTau proteins, tPA and PAI-1, expression of proteins of interest (REST) (Lu et al. , 2014), neurofilament light (NFL) (Zhou et al., 2017) and glial fibrillary acidic protein (GFAP) and iv) mood such as serotonin will allow us on the one hand **to characterize the health status of the participants and, on the other hand to determine the specific biological signatures of an 18-month meditative practice (intervention with non-meditating seniors) and of a long-term meditation practice (with the meditating expert population). A sample will be taken at the beginning of the trial, another at the end of the trial and another one after 35 months after the end of the intervention. We will thus be able to identify whether these biological markers can be modulated by this type of mental training and to trial these parameters in relation to cognitive changes occuring during normal aging.**

1.2.8. Effect of gender

Dementia in general and AD in particular are more common in women than in men because women may have a greater susceptibility to develop the disease (Alzheimer's Association 2014). Beyond AD, studies suggest that the processes involved in so-called "normal" aging differ between men and women (Gur and Gur 2002; Chêne et al. 2015). Recent reviews have emphasized the importance of investigating these specificities in more detail to better understand the mechanisms and develop the most appropriate treatments (Rocca et al., 2014). The origin of these differences is poorly understood. They could reflect intrinsic sex differences (genetically and/or hormonally related) (Gillies and McArthur, 2010), and/or lifestyle differences related to the socio-cultural context - inducing different exposure to dementia risk factors such as smoking (Ford et al., 2004), alcohol (French et al., 2014), diet, (Larrieu et al., 2004) depression (Djernes, 2006), or even educational level (Jack et al., 2015) or occupation (Valenzuela et al., 2013). On the other hand, recent evidence suggests that meditation-based interventions may benefit women more than men (de Vibe et al., 2013). In this trial, we will therefore focus on the effect of gender on AD risk factors, the mechanisms involved in aging and Alzheimer's disease, and the impact of meditation.

1.2.9. Electrophysiological markers of activation and rest by electroencephalography (EEG)

An evoked potential refers to the change in electrical potential produced by the nervous system in response to an external stimulation, particularly sensory (i.e. sound) recorded using techniques such as electroencephalography (EEG). EEG is a non-invasive brain imaging technique that does not involve

the injection of contrast media. No risk has been identified for this method. Evoked potentials are used in human neurophysiology to understand the functional organization of the nervous system. The MMN (mismatch negativity) is a component of the evoked potential generated mainly by the auditory cortex obtained by an oddball paradigm. The oddball paradigm consists of presenting a deviant or rare stimulus during the presentation of series of repeated stimuli with the same physical characteristics. The MMN is obtained by subtracting the EEG waves corresponding to the repeated stimuli from those of the deviant stimuli. The MMN is generated by any discriminable sound change in a repetitive stimulation environment. The amplitude of the MMN is lower and its latency longer in older adults indicating a deterioration and slowing down of the automatic detection of changes (NDDIN ZOTERO_ITEM CSL). MMN may also be used as a marker of cognitive decline and Alzheimer's disease (Ruzzoli et al. 2016). MMN occurs largely automatically, regardless of the subject's attentional focus, although recent studies have shown that it is modulable by attentional states such as meditation (Biedermann et al. 2016). **The use of this evoked potential thus appears to be a judicious electrophysiological marker to measure functional modifications related to aging and to explore the effect of aging, both at the cognitive and cerebral levels, following a sustained meditation practice in an older adults population.**

Before measuring MMN, we would like to record spontaneous EEG activity during a resting state for ten minutes. Resting state EEG allows the identification of rhythmic brain electrical activities that are classified according to their frequency. Analysis of the spectral power of these rhythmic activities, and of the functional connectivity of the oscillatory activity to these rhythms, allows us to characterize dynamic markers of psychological states such as meditative states or pathological states, like Alzheimer's disease. For example, we have recently shown that one-day meditation training increases the activity of theta (3-8Hz), alpha (8-13Hz) and beta (13-25Hz) rhythms over frontal and parietal electrodes. In contrast, many studies have linked Alzheimer's disease to a decrease in these rhythms and an increase in slow waves in the slow frequencies (delta 1-4Hz) (Babiloni et al. 2016). Long-range connectivity between fronto-parietal, or frontotemporal EEG electrodes are also abnormal in subjects with Alzheimer's (Babiloni et al. 2016). **The study of the resting EEG neurodynamic profile will be relevant not only to assess the effects of meditation in normal and pathological aging but also to try to better understand the mechanisms underlying these effects.**

2. OBJECTIVES

This research aims to **determine <u>the effects</u> of an 18-month meditation practice on imaging, biological, and behavioural markers** particularly associated with aging and AD and **to better understand the cognitive and emotional <u>mechanisms</u>** underlying these effects. All the objectives and corresponding analyses are detailed below.

We hypothesize that an 18-month meditation intervention will result in an increase in volume and/or perfusion of the anterior cingulate cortex. This intervention will be compared to a passive control (participants receiving no intervention in the trial). We also hypothesize that an 18-month meditation intervention will result in increased volume and/or perfusion of the insula compared to a foreign language

learning intervention, designed to have a similar structure to the meditation intervention. The non-specific effect of meditation on the anterior cingulate cortex will be estimated by comparing it to the passive control group. The specific effect of meditation on the insula will be estimated by comparing it to the foreign language learning group.

2.1. *Main objectives*

The **main objectives** of the trial are:

- To estimate the effect of an 18-month meditation intervention in non-meditating senior participants on volume and perfusion of the anterior cingulate cortex compared to a passive control (**OBJECTIVE 1**);
- To estimate the effect of an 18-month meditation intervention in non-meditating senior participants on the volume and perfusion of the insula compared to an 18-month foreign language learning intervention (**OBJECTIVE 1bis**).

2.2. *Secondary objectives*

2.2.1. Secondary objectives in relation to the interventions

OBJECTIVE 2: To compare the effects of the two interventions (meditation and foreign language learning, for 18 months) with each other and with a passive control in non-meditating seniors, on behavioural and biological measurements (cognitive, psycho-affective, blood, sleep, and imaging), including measurements to assess potential deleterious effects as well as gender-specific effects (men/women);

OBJECTIVE 2 bis: To estimate the maintenance of the effect of the interventions (meditation and foreign language learning) in the long term (29 months after the end of the intervention) on cognition, psycho-emotional factors, sleep, and lifestyle (and imaging and blood markers if the budget allows).

OBJECTIVE 2 ter: To compare the long-term effects (29 months after the end of the intervention) of the 2 interventions (meditation and foreign language learning) with each other on cognition, psycho-emotional factors, sleep, and lifestyle (and imaging and blood markers if the budget allows).

OBJECTIVE 3: To estimate the effect of the interventions (meditation and foreign language learning, for 18 months) on the volunteers' entourage (i.e., their perception of the volunteers' changes, their interaction with the volunteer, and their willingness to help the volunteer), compared to a passive control.

OBJECTIVE 3 bis: To compare the long-term effects of the interventions (meditation and foreign language learning) on the volunteers' entourage (i.e., their perception of the volunteers'

changes, their interaction with the volunteer, and their willingness to help the volunteer) 29 months after the intervention ended.

OBJECTIVE 3 ter: To estimate the effect of the interventions (meditation and foreign language learning, for 18 months) (i.e. their acceptance of the intervention, their perception of the changes...).

OBJECTIVE 3 quater: To assess the consistency between participants' perceptions of the interventions and their effects and those of the meditation or English teachers (after the interventions have ended).

2.2.2. More exploratory secondary objectives (not related to the interventions)

OBJECTIVE 4: To estimate the association between meditation expertise and behavioural and biological measurements (cognitive, psycho-affective, life factors, blood, sleep, and imaging), comparing expert meditators and non-meditating seniors;

OBJECTIVE 5: To identify, in senior meditation experts, the neural signatures of two meditative practices (mindfulness meditation and compassionate meditation), i.e. the specific neural activity associated with each practice;

OBJECTIVE 6: Explore associations between lifestyle factors (cognitive activity, diet, and physical activity), psycho-emotional measurements, and behavioural and biological measurements (cognitive, psycho-emotional, lifestyle factors, blood, sleep, and imaging), as well as gender-specific associations (male/female);

OBJECTIVE 7: To explore the pathophysiological mechanisms of AD by studying the links between different measurements as well as the influence of structural and functional connectivity on the spread of lesions, as well as gender-specific effects (male/female);

OBJECTIVE 8: To compare the early FDG-PET perfusion measure to the early Amyvid®-PET perfusion measure to better understand the relationships (and potential changes in coupling) between metabolism and perfusion and compare the sensitivity of the two measurements to assess the effects of interventions;

OBJECTIVE 9: To identify determinants of well-being and mental health in aging in volunteers and their designated partners from cognitive data, psycho-emotional, sleep, and lifestyle measurements (as well as imaging and blood markers if budget allows) in passive control participants.

The list of exploratory analyses may be updated according to the progress of knowledge in the field or the results of the primary or secondary objectives. The scientific rationale will be presented to the Scientific Council.

3. TRIAL TYPOLOGY AND EXPERIMENTAL DESIGN

3.1. *Trial type*

Biomedical research with health product, monocentric, studying two participant populations (expert meditators and non-expert seniors):

1) Monocentered, randomized, controlled trial with blinded assessment of achieved measurements, with three parallel arms comparing an 18-month meditation intervention, an 18-month foreign language learning intervention, and a no-intervention group, in volunteer participants over 65 years of age with no meditative practice experience (non-meditative seniors).

2) Cross-sectional study evaluating the association between meditation practice and health status markers, including consecutively meditating experts in order to compare their characteristics with those of non-meditating seniors at inclusion in the randomized trial.

This trial is classified as non-health product despite the use of Amyvid® and/or FDG in PET imaging scans performed at inclusion (Visit 1 and for meditating experts), 18-month follow-up (Visit 3) and long-term follow-up (35 months post-intervention) of all participants.

3.2. *Statement of the experimental design*

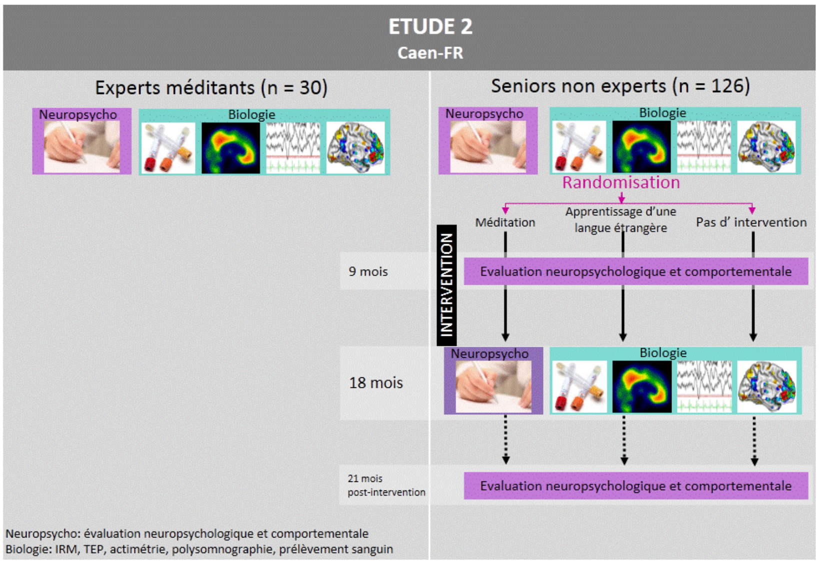

Figure 2: Experimental design of the Age-Well trial

Four groups will be made:

Expert Meditators	Non-expert seniors		
	Meditation Group	Foreign Language Learning Group	Passive Control Group
30 expert meditators	50 seniors with 18-month meditation	50 seniors with 18-month english lessons	50 seniors with no intervention

Tableau 1: Study groups

Methods for randomization:

The randomization list will be established by the biostatistician of the Centre de méthodologie et de gestion (EUCLID, CHU Bordeaux, France and Inserm, F-CRIN platform) before the beginning of the research. The allocation of the intervention will be done according to a 1:1:1 ratio.

- Group 1: Meditation
- Group 2: Learning a foreign language (English)
- Group 3: Passive control (follow-up without intervention)

A document describing the randomization procedure will be kept confidential by the EUCLID Statistician. The randomization of the participants will be done before the beginning of the intervention by the investigator in a centralized way via the Ennov Clinical software. This centralization allows the investigator and possibly other correspondents to be informed of the randomization of each participant. From the second wave onwards, the foreign language learning group will be divided into 2 subgroups in order to respect the logistic constraints on group size. The allocation in each of the two English groups will be drawn at random, and adapted by the English teachers if necessary from the level assessed during the English test carried out during the diagnostic battery, to limit the heterogeneity of level within the same group.

Assignment of the non-medical senior intervention is unbeknownst to the individuals responsible for data analysis for all visits, including visit 4.

The investigating team (medical investigators, neuropsychologists, radiological manipulators, nuclear physicians) knowing the identity of the participants is not the one who analyses the data.

Interventions:

The content of the different interventions is described in section 6.9.

3.3. *Provisional schedule of the trial (Flow-Chart)*

The projected schedule for the trial is illustrated in Figure 3 below:

Duration of recruitment:

- Non-expert seniors: recruitment will take place 3 months before the beginning of each inclusion wave, i.e. 9 months of recruitment.
- Expert meditators: recruitment will be done on an ongoing basis and over a total period of 73 months (until February 2022; due to an interruption in recruitment in 2020 and part of 2021 linked to COVID-19 and the resulting health measures).

Maximum duration of participation for each participant:

- Expert meditators: 6 months (Screening visit V0 + Inclusion visit V1)

And in the Age-Well substudy - Lockdown: 24 months (April 2020-April 2022)

- Non-expert seniors: 53±6 months (3 months for V0 + V1 + 18 months for intervention + 3 months for Follow-up Visit 3 + 29 months without intervention which is the long-term post-intervention visit (V4)) (± 6 months depending on health status changes and a possible new period of confinement).
Total duration of data collection: 6 years (1st participant included in December 2016 and last follow-up of the last participant: in June 2022 ± 6 months depending on the evolution of the health situation and a possible new containment period).
And in the Age-Well substudy - Lockdown: 24 months (April 2020 - April 2022)
Total duration of the research: 7 years (6 years for data collection + 1 year for data analysis) as shown below:

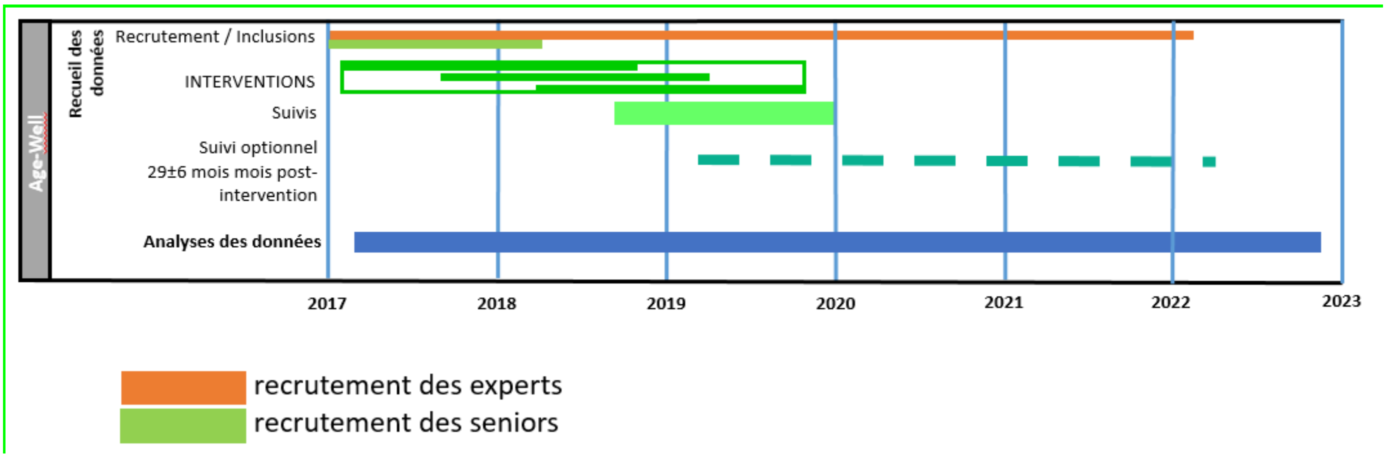

Figure 3: Provisional schedule of the Age-Well trial (data collection and analysis).

4. SELECTION OF PARTICIPANTS

4.1. *Study populations*

Group	Description of the participants	Number to be included	Duration of participation in the trial
**Expert meditators**	Independent and willing people over 65 years old with a regular practice of mindfulness and compassionate meditation	30	6 months
**Non-expert seniors**	Independent and willing people over 65 with no previous meditation experience	150 (included in 3 waves)	53 ± 6 months

Tableau 2: Study Populations

4.2. *Eligibility criteria*

Eligibility criteria will be verified during the recruitment phase, which includes an inclusion visit with the investigating physician and psychologist. The investigator will be responsible for providing all the necessary information and collecting the signed consent of the participant before any procedure is performed. After this interview, the inclusion of the participants will continue with a psychologist who will conduct an interview and tests.

Monitoring of eligibility criteria will take place over a maximum period of 3 months after consent is signed. It is assumed that the set of measurements performed within this time frame reflects a steady state characterizing the participant's condition at inclusion.

4.2.1. Inclusion criteria

For all the participants:

- Age ≥ 65 years old;
- Be independant (assessed by the following question asked by the physician during an interview with the participant, and their partner when present: would you be able to live on your own, on a purely "functional" and not emotional level?);
- Living at home;
- Have a level of education ≥ 7 years (from the 1rst grade included);
- Be a member or beneficiary of a social security regime;
- Be motivated to participate effectively in the project and sign the informed consent of the protocol in agreement with the Comité de Protection des Personnes;

- Present normal neuropsychological performance (based on age, gender, and education level) on the diagnostic battery tests (Table 4, Section 6.4).

Specific criteria for non-experts seniors:

- Be a native French speaker;
- Be available for at least the next 24 months (this criterion will be verified during the individual interviews or the question of availability for the next 24 months may be explored);
- Retired from the profession for at least 1 year;
- Have no strong preference nor objection to any of the three intervention groups (assessed by a question to the participant);
- Not have practiced meditation or comparable practices (yoga, Qi Gong, Alexander Technique) regularly or intensively EITHER:
    - > 1 time per week for 6 or more months in a row in the past 10 years,
    - intensively (internship or retreat > 5 consecutive days) within the last 10 years,
    - > 25 days of retirement accumulated before the last 10 years;
- Not fluent in English (assessed by asking the participant, "Are you fluent in English, can you hold a conversation with ease?"; only those answering no to this question will be included in the trial).

Specific criteria for the expert meditators:

- At least 10,000 hours of formal meditation practice in a lifetime, including at least 6 months in retreat;
- Have a daily meditation practice (at least 6 days/week, at least 45 minutes per day on average; Practice mindfulness meditation (i.e. mindfulness, Samatha/ Vipassana, Zazen (Zen), Shikantaza (Zen), focused attention, Mahamudra/ Dzogchen), and compassionate/ loving-kindness meditation (i.e. tonglen practice, "four immeasurable qualities" (metta/ karuna) practices, Bodhichitta meditation).

These criteria will be assessed in a semi-structured interview including a detailed assessment of the type, duration and frequency of meditation during the lifetime.

4.2.2. Non-inclusion criteria

- Presence of contraindications to MRI examination (claustrophobia, ferromagnetic object in the body) or to Amyvid®; or Glucotep® PET scan
  The difference in perfusion in specific brain structures measured by PET (early measurement of the radiopharmaceutical Amyvid®) at inclusion and at 18 months is one of the primary evaluation criterion of the trial. For safety precautions in the use of Amyvid® and Glucotep®, a

blood sample to check renal and hepatic functions will be taken at the V1 visit, at the 18-month follow-up visit V3, and at the long-term follow-up visit 29 ± 6 months post-intervention V4 prior to this imaging examination. The glomerular filtration rate will be calculated from the results obtained.

In case of renal insufficiency, hepatic insufficiency, unbalanced diabetes, blood sugar > 1.6g/L or any other biological abnormality of grade 3 or higher detected during these analyses, the volunteer will not be able to perform the PET scans. In this case, the test results will be sent to the physician indicated by the participant;

- Known hypersensitivity to Amyvid® or Glucotep®;
- Presence of a known major neurological or psychiatric condition (including addiction to alcohol or psychoactive substances capable of causing strong psychic and/or physical dependence);
- HIstory of brain disease (vascular, degenerative, malformative, tumor, or head trauma with loss of consciousness over one hour);
- Presence of an unstabilized chronic or acute disease (respiratory, cardiovascular, digestive, renal, metabolic, hematological, endocrine or infectious);
- Current or recent use of medications that may interfere with cognitive or imaging measurements (psychotropic drugs, anti-histamines with anticholinergic action, anti-parkinsonian drugs, benzodiazepines including muscle relaxants, long-term steroidal anti-inflammatory drugs, anti-epileptics, central analgesics);
- Be under guardianship or curatorship;
- Inclusion in another biomedical research protocol at the time of trial entry if the study includes imaging studies using radiomarkers;
- Physical or behavioural inability to comply with trial follow-up.

Participants meeting only one of the non-inclusion criteria may not be eligible to participate in the research .

4.3. *Recruitment method*

The meditating experts will be recruited in France and abroad under the responsibility of Antoine Lutz (Research Fellow at the Research Center in Neurosciences, Lyon, France), who has experience in several neuroscience researches on meditating experts, with the help of Mr. Matthieu Ricard (Centre d'Etudes de Chanteloube, France), Expert-Associate. This recruitment will be done in meditation centers and monasteries of the Buddhist tradition. Participants will have to follow the criteria for inclusion in the trial as detailed below, and will have to be recommended by the meditation teachers of their center. These centers will be required to offer teachings and practices in mindfulness meditation techniques and compassionate and loving-kindness meditation. The recruitment process will be as follows. With the support of Mr. Matthieu Ricard, who is an emblematic figure of Buddhism in France, we will contact teachers in charge of Buddhist meditation centers in France and in Europe to present our trial and our needs for the trial. These teachers will contact meditation practitioners.

The following centers and their affiliates will be contacted:

1) Centre de méditation de la tradition du Bouddhiste Tibétain dans la lignée Kagyu, la lignée Nyingmapa et la lignée Gelugpa.

- Monastère et centre de meditation Dhagpo Kundreul Ling: Le Bost 63640 Biollet (http://www.dhagpo-kundreul.org/index.php/fr/)
- Centre d'étude et de pratique du bouddhisme tibétain de Montchardon 8900 route de Montchardon 38160 IZERON, France (http://www.montchardon.org/indexspip.php)
- Association du Centre d'Etudes de Chanteloube: La Bicanderie 24290 Saint-Léon-sur-Vézère, France (http://chanteloube.pagesperso-orange.fr/chanteloube.htm)
- Centre Rigpa de Lérab Ling L'Engayresque, 34650 Roqueredonde, France (https://lerabling.org/lang-fr/)

2) Centres de méditation du Bouddhiste Théravada:

- L'Association de Méditation Bouddhique – Centre Vimalakirti, Rue Henri-Frédéric Amiel 10, 1203 Genève (http://www.vimalakirti.org)
- Centre Bouddhique International - Pagode du Bourget, 7, Rue Cité Firmin Bourgeois, 93350 Le Bourget (http://centrebouddhique.fr)

3) Centres de Bouddhisme Zen Coréen, Vietnamien, ou Japonais:

- Village des Pruniers (Thich Nhât Hanh), Le Pey 24240, Thenac, France (http://villagedespruniers.net)

The procedure for recruiting the non-expert seniors is shown in Figure 4. The non-expert seniors will be recruited in the Caen area through a communication campaign established by two communication companies: Minerva, located in London and specialized in the communication of European projects, and Mediapilote, located in Caen and responsible for its local implementation. This communication campaign will be aimed at the general public and at target institutions having a privileged link with the population of interest in this trial, i.e. people over 65 years old (the CLIC - Centres Locaux d'Information et de Coordination gérontologique, l'Espace 14, the Calvados Department, the Basse-Normandie Region, the Caen La Mer Community of Municipalities, etc.) The recruitment will be done through posters, and via various media. A conference and an online questionnaire (secure site) will allow to explain the trial to the participants and to verify their motivation as well as to check that the participants who will be invited to sign the consent form correspond to the target population (e.g. over 65 years old, retired for more than one year, etc). One of the investigating physicians will be present during the conferences explaining the protocol to potential future participants in order to answer any questions. The signature of the consent form will only be done at a second stage during the selection visit (V0 visit, see below) which will take place at least one week after the conference in order to give the participants time to reflect..

RECRUTEMENT		VISITE DE SELECTION - V0		
Campagne d'information	Conférence et présélection	Signature du Consentement	Entretien médical	Examens psychologiques (entretien et batterie de tests diagnostiques)

Figure 4: Recruitment procedure

5. Evaluation criteria

The evaluation criteria are listed below with reference to the objectives they will address.

5.1. *Main evaluation criteria*

- For the comparison between meditation group and passive control group (**OBJECTIVE 1**):
  - Volume difference in the anterior cingulate cortex measured on T1-weighted MRI at inclusion and at 18 months;
  - Perfusion difference in the anterior cingulate cortex measured by PET (early frames of the radiopharmaceutical Amyvid®) at inclusion and at 18 months.

- For the comparison between meditation group and foreign language learning group (**OBJECTIVE 1bis**):
  - Volume difference in the insula measured on T1-weighted MRI at inclusion and at 18 months;
  - Perfusion difference in the insula measured by PET (early frames of the radiopharmaceutical Amyvid®) at inclusion and at 18 months .

5.2. *Secondary evaluaion criteria*

- Behavioural measurements (**OBJECTIVES 2, 4, 6, 7, 9**) :
  a composite score per function assessed by the neuropsychological tests and questionnaires (listed in Tables 5 and 6 below, see section 6.7). The composite scores will be an average of z-scores calculated from all scores collected for the corresponding function that have a normal distribution and do not show a floor/ceiling effect. (In addition, exploratory analyses will be conducted on all individual behavioural measurements collected).

- Imaging measurements (**OBJECTIVES 2, 4, 6, 7,8, 9**):
  - Volume of gray matter in each brain voxel;
  - Volume of the hippocampus and hippocampal subfields;
  - Brain perfusion measure in each brain voxel;
  - Fractional anisotropy and mean diffusivity in each brain voxel;
  - Number, size and location of white matter lesions;
  - Magnetic susceptibility measured in each brain voxel;
  - Functional brain connectivity in a non-meditative state;
  - Beta-amyloid load (from the radiopharmaceutical Amyvid®) measured in each brain voxel and global average in the gray matter;
  - Cerebral glucose consumption at rest (relative to the average consumption measured in the cerebellum
  - Brain activity measured in fMRI specifically associated with emotional processing (comparing emotional and neutral items) from the Rest-SoVT task.
  - Brain activity associated with attentional processus of alertness, inhibition and sustained attention measured in fMRI during the AX-CPT task.
  - Spontaneous oscillatory activity (EEG) in a resting state.
  - EEG brain activity specifically associated with the processing of auditory stimuli (MMN protocol).

- Sleep measurements (**OBJECTIVES 2, 4, 6, 7, 9**):
  - Subjective sleep measurements collected by sleep questionnaires (Tables 6 and 7 below)
  - Actimetry parameters (average sleep duration, fragmentation index of activity and rest periods, regularity of activity/rest cycle).
  - Data collected through polysomnographic recordings
  - Data collected with the Somno-Art device.

- Biological measurements (**OBJECTIVES 2, 4, 6, 7, 9**):
  - Blood measurements as listed in Table 8 below, see section 7.3. 4 (such as total cholesterol, BNP, bioavailable testosterone, serotonin, estradiol, SHBG, SDHEA, cytokines, cortisol,

telomere length, telomerase activity, Aβ 1-42, Tau/phosphoTau proteins, tPA and BDNF, fibrinogen and GFAP)
- Anthropometric measurements (weight (kg), fat (%), fat mass (kg), lean mass (kg), hydration rate (kg), muscle mass (kg), bone mass (kg), impedance (Ohm), basal metabolic rate (kJ; kcal), visceral fat (level) and metabolic age (year), as well as BMI, Sarcopenic Index (SMI; kg/m²) and Skeletal Muscle Mass (SMM; kg and %).
- Charlson and CIRS scores

- <u>Impact on the participants' entourage (**OBJECTIVE 3**):</u>
  - Scores from the questionnaires given to the participants' partners as listed in Tables 5 and 6 below.
  - Qualitative data from interviews with participants and teachers.

- <u>Neural signature of meditative practices (**OBJECTIVE 5**):</u>
  - Measurement of neural activity in each brain voxel in fMRI at rest, and during the rest-SOVT task, in a mindfulness meditation state versus a non-meditative state ;
  - Measurement of neural activity in each brain voxel in fMRI at rest, and during the rest-SOVT task, in a compassionate meditative state versus a non-meditative state.

- <u>Measurement of the tolerance to the intervention (**OBJECTIVE 2**):</u>
  - Occurrence of adverse events;
  - Anxiety measured by the State-Trait anxiety Inventory (STAI);
  - Depression as measured by the Geriatric Depression Scale (GDS);
  - Positive and negative emotions measured by the questionnaire PANAS-NOW;
  - Satisfaction and well-being measured by self-questionnaires.

5.3. *Methods and timelines for measuring, collecting, analyzing, and reporting on the outcomes of the trial (efficiency or other criteria)*

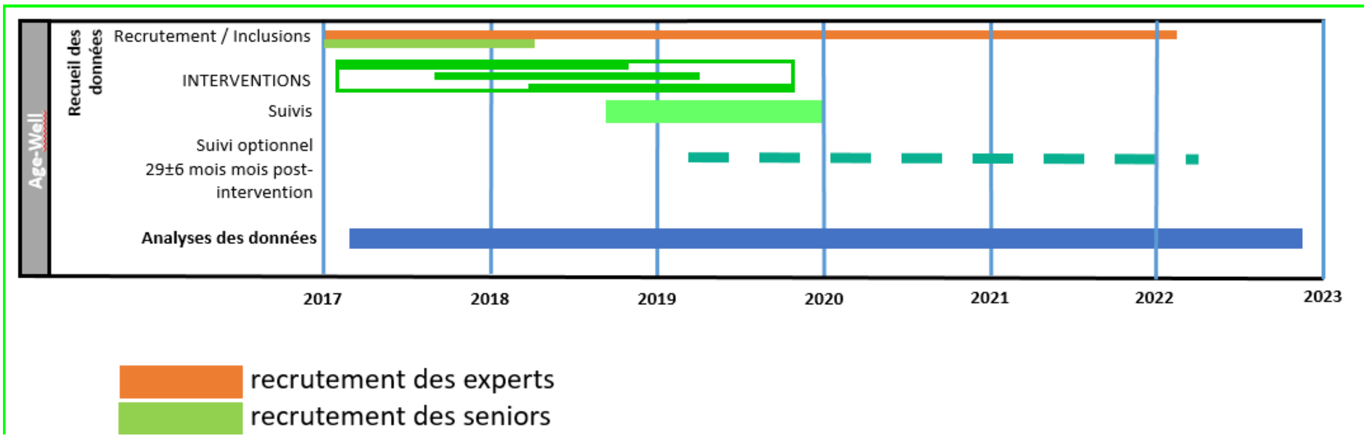

Figure 5: Provisional schedule of the Age-Well trial (data collection and analysis).

5.4. *Compliance monitoring criteria*

Adherence will be measured throughout the intervention via a very brief questionnaire to be filled out each week, allowing in particular to measure the number and duration of weekly exercises. The questionnaire consists of 6 daily questions to be filled in once a day in the form of an application for an Ipad 2 tablet, developed internally by Antoine Lutz's team at the Research Center in Neurosciences,

Lyon, Inserm. The 6 questions intended for the subjects of the English and meditation groups are composed of 4 multiple choice questions and 2 open questions. The first question concerns the well-being of the person (the choice of answers is presented in the form of a table in which the rows represent different intensities (very big, big, small, very small) and the columns different weather images (sun, sun and small cloud, sun and big cloud, cloud, rain, flash cloud, double flash). The second question asks about formal practice time for the day (0 min, less than 15 min, between 15 and 30 min, between 30 and 45 min, between 45 and 60 min, between 60 and 90 min, between 90 and 120 min, over 120 min). Formal practice is defined as practice related to the instructions, exercises, or materials (books and applications) of the course for the English intervention. For the meditation group, formal practice is defined as practicing meditation exclusively without doing another activity at the same time. The third question asks about the duration of their informal practice (0 min, less than 15 min, between 15 and 30 min, between 30 and 45 min, between 45 and 60 min, between 60 and 90 min, between 90 and 120 min, more than 120 min). Finally, the fourth question asks about motivation to practice during the day (very low, low, medium, high, very high). The fifth question asks about the geographic area where the subject is located during the day (free text). The last question gives subjects the opportunity to leave comments on the intervention in general.

To increase the participants' retention during the trial, participants' motivation will be assessed through an interview with the psychologist prior to the start of the trial (see inclusion visit below). Participants will be strongly encouraged/motivated by the trainers to participate in all activities throughout the duration of the intervention.

6. Practical realization of the protocol

6.1. *Investigating center*

All visits will be made in Caen, France.

The screening, inclusion and follow-up visits will be conducted at:

- the Cyceron center, (Boulevard Henri Becquerel in Caen). This is an imaging platform with a 3T MRI (Phillips Achieva), a PET camera (Discovery RX VCT 64, GE Healthcare) and test rooms where all the brain imaging examinations and neuropsychological and behavioural assessments will be performed (see below).
- the Clinical Research Center (CRC) of the University Hospital of Caen (CHU), Avenue de la Côte de Nacre, where the blood samples included in the protocol will be taken.
- the Pôle Formation en Recherche et en Santé (PFRS; 2 rue des Rochambelles, Caen), where the electrodes and various sensors for the polysomnographies are installed and removed.

Thus all participants will perform the examinations under similar conditions and on the same scanners.

The intervention (non-expert seniors only) will take place at:

- the Pôle Formation en Recherche et en Santé (PFRS; 2 rue des Rochambelles, Caen) for meditation courses.
- the Carré International, at the University of Caen (Esplanade de la Paix, Caen) for foreign language courses.

6.2. *Follow-up schedule of the participants*

In general, the trial will be organized as follows:

	SENIORS group			EXPERTS group
	**Meditation group**	**English Learning group**	**Passive Control group**	
General information	x	x	x	x
Recruitment in three waves via an information campaign	x	x	x	
Recruitment by Expert-Associate				x
Information for the participant by phone	x	x	x	x
Assignment of a referent for the participant	x	x	x	x
Organization of the 1st visit by phone	x	x	x	x
V0: Consent signature and screening visit	x	x	x	x
Signature of consent	x	x	x	x
Medical interview	x	x	x	x
Neuropsychological consultation	x	x	x	x
V1: Inclusion visit (up to three months)	x	x	x	x
Blood sample V1	x	x	x	x
MRI session 1	x	x	x	x
MRI session 2	x	x	x	x
MRI session 3				x
EEG session	x	x	x	x
Amyvid® PET scan	x	x	x	x
^{18}F-FDG PET SCAN	x	x	x	x
Neuropsychological and behavioural assessment V1 (2 sessions + additional questionnaires)	x	x	x	x
Sleep assessment	x	x	x	x
Randomization	x	x	x	
18-month intervention	x	x		
Weekly group meditation session	x			
Daily meditation exercise at home	x			
Days and half days of intensive meditation practice	x			
Weekly group language courses		x		
Daily language training at home		x		
Days and half days of intensive language practice		x		
Completion of diary/questionnaire for intervention follow-up	x	x		
V2: Intermediate evaluation visit at 9 months	x	x	x	
Neuropsychological and behavioural assessment V2 (1 session)	x	x	x	
V3: End of intervention visit	~~x~~	~~x~~	~~x~~	
Medical interview	x	x	x	
Series of examinations identical to V1	x	x	x	
Qualitative interview about the intervention experience	x	x	-	
V4: Visit 29 months post-intervention				
Medical interview	x	x	x	
Identical series of examinations at V1 and V3 (except PET-Amyvid and polysomnography)	x	x	x	

Tableau 3: Age-Well trial Flow-Chart

The details of the visits are as follows:

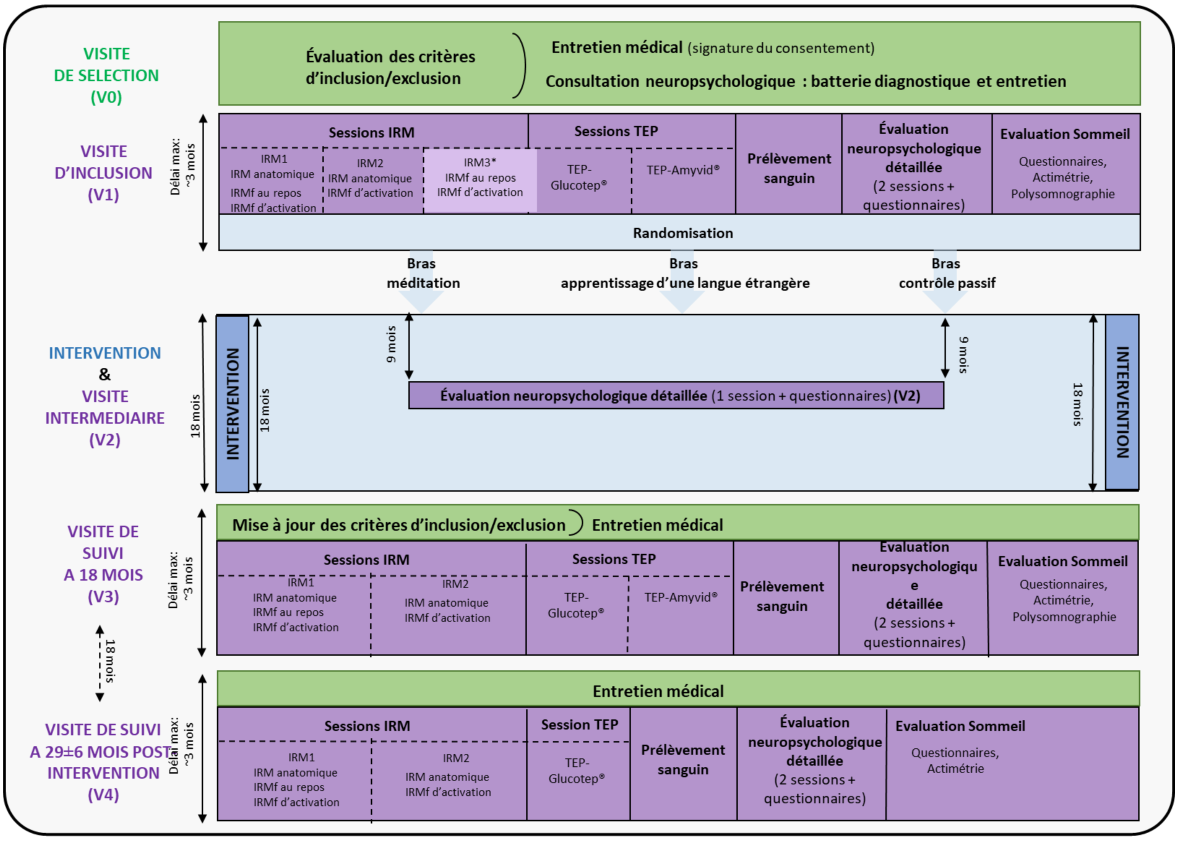

Figure 6: Practical procedure of the Age-Well trial detailing all the examinations.

** examination offered only to expert meditators.*
*** This visit will include a neuropsychological and behavioural assessment and will be supplemented by other measures such as imaging, blood biomarkers, and sleep measurements).*

6.3. *Recruitement and screening*

For how participants were recruited, see section 4.3.

6.4. *Visit no.0 – Screening visit*

The screening visit concerns all participants without exception and takes place in the recruitment center in Caen. This selection visit is mandatory and includes two steps during which the inclusion and non-inclusion criteria will be verified. Each person pre-selected for the protocol will have to complete:

1) An interview with an investigating physician.

The investigating physician will explain to the volunteer the whole trial and the protocol; he/she will give the volunteer the information documents and the consent form (see Annex 2), specific to his/her inclusion group (meditating expert or non-expert senior). Once all of the volunteer's questions have been answered, the investigating physician will be responsible for obtaining free and informed consent. The participants will have been clearly informed during the recruitment phase (information campaign, conference and screening, see 4.3), thus giving the participant sufficient time to reflect before the

medical interview. Without consent, participants will not continue with the screening examinations and cannot be included.

During this interview, the physician will also verify the absence of contraindications to the MRI examination (claustrophobia, ferromagnetic material in the body). In addition, the medical history and drug treatments of the participants will be listed in order to verify the corresponding non-inclusion criteria. From the medical data collected during this interview, a comorbidity index will be calculated (Charlson et al., 2008), and the CIRS score (see detailed description in visit 4).

2) *Consultation with a neuropsychologist*.

During this consultation, the tests of the diagnostic battery (see Table 4) will be proposed to the participants by an investigating neuropsychologist. These tests will ensure the absence of cognitive disorders (see table below). No results will be communicated to the participant. The presence of pathological abnormality(ies) will be communicated to the participant and to his/her regular physician (who will then ensure the follow-up), and will lead to the termination of the participation.

In addition, during this consultation, a selection interview will take place with the participant in order to evaluate, among other things, his eligibility for the protocol and his constraints, his motivation, etc., in order to increase the retention of participants during the trial.

	**Diagnostic battery**			
**Evaluated functions**	**Tests**	**Score(s) collected**	**References**	**Expected performance**
Manual laterality	*Edinburgh Questionnaire*	Unique	*Oldfield, 1971*	*Not applicable*
Global cognitive functioning	*MMSE*	Unique	*Folstein et al, 1975*	*Age, gender and education standards (see ref.)*
Depressive state	*Montgomery and Asberg Depression Rating Scale*	Unique	*Montgomery & Asberg, 1979*	*Score < 19*
Executive functions	*Wisconsin Card Sorting Test*	Multiple	*Godefroy& Grefex, 2009*	*z-score > -1.65 compared to norms (calculated according to age, gender and education level (see ref.))*
Verbal episodic memory	*RL-RI16*	Multiple	*Van der Linden et al., 2004*	*z-score > -1.65 compared to norms (calculated according to age, gender and education level (see ref.))*
English test	*Test of written and oral comprehension*	Unique	*Original Test*	*Score < 16/18*

Table 4: Diagnostic Battery - Neuropsychological tests performed during the selection visit - Battery (~60 min)

6.5. *Participant Identification (PI) in the protocol*

Once the screening visit is complete, consent has been signed, and it has been verified that the participant can be included, a code will be assigned. The identification of participants is anonymous and will correspond to the combination of their inclusion number in order of arrival (letter code + number). The correspondence list containing the identity of the participants and their anonymization code will be kept in a secure place at the Cyceron Center accessible only to the research personnel of this trial. In addition, each participant will, at this stage, be integrated into the national file of persons engaged in medical research, managed by the Ministry of Health.

6.6. *Visit no.1 – Inclusion visit*

The inclusion visit will take place in the investigating center in Caen, shortly after the participant's selection visit (less than 1 month delay). It includes different MRI sessions (anatomical and functional), two PET scans (Amyvid® and ^{18}F-FDG), a blood sample, a detailed neuropsychological evaluation and a polysomnographic examination on awakening and during a night of sleep (see Figure 5 above).

The inclusion of expert meditating participants will be done "as it happens". On the other hand, the inclusion of non-expert seniors will be carried out in three waves (42 participants for wave 1 and 54 participants for waves 2 and 3). This organization in waves is linked to i) the logistical organization of the different appointments of the inclusion visit and ii) the logistics of the intervention periods (e.g. courses in groups, of reasonable size).

For each of these appointments, the participant will be taken care of by one of the investigators. As far as possible, this will be the same person for all the examinations. This person, called the "referent", will be responsible for organizing the various appointments for this visit and for following up with the participant on the site (welcoming and accompanying the participant on each visit). The neuropsychological tests can be done by the referent or by an investigating neuropsychologist. All the examinations will be carried out according to a standardized procedure (pre-established procedure in the course of the imaging and biological examinations and standardization of the instructions of the various tests proposed in functional MRI or during the neuropsychological evaluation).

The examinations of this inclusion visit will be organized in several appointments and spread over a maximum duration of 3 months (6 months for the experts); time constrained by the feasibility of the various examinations, mainly for those of imaging (availability of the apparatuses, availability of the personnel supervising these examinations, constraints of delivery of the PET tracers according to the capacities of production and their duration of half-life). The different appointments will be determined according to the availability of the participants and the availability of the brain imaging tools, with the constraint of carrying out the first MRI session ("MRI1 session") first, as this will allow us to ensure the absence of brain abnormality. The presence of pathological abnormality(ies) will be communicated by the investigating physician to the participant and to their regular physician (who will then ensure the participant's follow-up), and will lead to the participant's termination of participation. In addition, the

different MRI sessions must always be performed in the predetermined order (see below). At each visit, it will be verified that the participant still meets the eligibility criteria.

Blood sample on V1

The blood sample will be used to measure the various biological parameters detailed in section 7.3, and in particular the parameters concerning the hepatic and renal functions of the participant.

Blood sampling for liver, kidney function and blood sugar must be done after the consent is signed and before the first PET scan is performed. The results must be known to the investigating physician before these examinations are performed. The interpretation of the results (in particular via the calculation of the glomerular filtration rate) concerning liver, kidney function and blood sugar will be the responsibility of the investigating physician and will condition the performance of the PET imaging examinations during the V1 visit. A biological abnormality of grade 3 or higher constitutes a criterion of ineligibility for the trial.

At this time, the sample required for GWAS and APOE genotyping will also be collected, which will be specified in the consent.

The sample will be taken in the morning at the CRC of the University Hospital of Caen. Due to the constraints related to the determination of some of the blood biomarkers, it will be necessary that the sample is collected in the morning between 8 and 10 am, on an empty stomach, and after one day of diet excluding foods rich in serotonin (tomatoes, citrus fruits, dried fruits, avocados, pineapples, plums, chocolate and bananas). The participant will be reminded of the instructions concerning the diet prior to this sampling.

On the day of the sampling, each participant will join their referent at the CRC of the CHU of Caen. Once the sample has been taken, the participant will be invited to have a snack if they needs to continue with another examination.

All the tubes collected (maximum total volume of 68 mL) will then be transmitted directly by the CRC of the University Hospital of Caen to the various laboratories responsible for the analyses (see section 7.3).

Brain imaging examinations in MRI

Two MRI sessions will be offered to all participants (meditating experts and non-expert seniors). A third session will be offered only to the group of meditating experts. All MRI sessions will take place at the Cyceron center in Caen and the three sessions will always be conducted successively, as indicated below (MRI session 1 will always precede MRI session 2, which will precede MRI session 3). Prior to each MRI session, the participant will be reminded (by phone and/or during the previous visit)

that they must limit their consumption of coffee before the examination, present theirselves without make-up, hair spray or gel, and that all jewelry or metallic objects must be removed before entering the MRI.

MRI1 session

The MRI1 session will always be performed at the beginning of the protocol, after the consent signature (performed at the beginning of the inclusion visit) and before any other examination at the inclusion visit. The sequence of this MRI session is shown in Figure 7. Upon arrival at the Cyceron center, the participant will be greeted by their referent, who will remind them of the upcoming examination. The participant will then be asked to complete a self-assessment questionnaire (STAI-A, Spielberger et al., 1983). This questionnaire makes it possible to evaluate "state anxiety", i.e. at the very moment when the participant fills it out. As the anxiety-state may change from one appointment to another, it will be proposed several times. The participant will then be asked to put on pyjamas, leave clothing and any jewelry, glasses or dental/auditory appliances in a secure changing room, thus ensuring that no metallic material is present at the MRI. Blood pressure, weight, height, abdominal circumference and hip circumference will be measured during this step (measurements used as indices in the measurement of cardiovascular risk). Before entering the MRI, the participant will be provided with disposable earplugs to reduce noise from the machine. The participant will then be placed in the MRI by the radiology manipulators.

The rest-SoVT task (proposed in fMRI at the end of this first MRI session) will then be explained to the participant. The instructions will be given to the participant, who will then be invited to perform a short training session on the computer, in order to make sure that the instructions are well understood. In total, this training will take about 10 minutes.

The details of the MRI sequences for the first session are shown in Figure 7 below. After a baseline scan, the first part of the examination will be devoted to anatomical acquisitions, for a duration of approximately 35 minutes. These anatomical acquisitions will include a T1-weighted sequence, a T2-weighted hippocampal targeted sequence (T2 HCP), a T2-weighted sequence and a FLAIR sequence.

A 10-minute resting fMRI sequence will be proposed. During the fMRI examination, brain function will be assessed at rest (as opposed to during an activation task). No task is proposed and the participant will be asked to "keep eyes closed, don't sleep and let thoughts flow freely". However, the instruction given to the meditating experts will invite them to perform this sequence in a meditative state. For half of the experts, the sequence will be performed in a state of mindfulness meditation, for the other half it will be performed in a state of compassionate meditation. The experts who performed the sequence in a state of mindfulness meditation during this session will perform it in a state of compassionate meditation during the MRI3 session (see below), and vice versa for the other half of the experts. This functional sequence will be preceded by a T2*-weighted sequence, necessary for the pre-processing of the fMRI data.

The MRI examination will be followed by an activation fMRI acquisition during which the rest-SoVT task will be proposed. During the rest-SoVT task, participants will be asked to watch short videos (less than 20 seconds), either neutral or emotional (people expressing a suffering emotion). A total of

24 videos, divided into 8 series, will be presented. For each series of videos, a period of exposure, corresponding to the successive presentation of 3 videos expressing the same emotion, will be followed by a 90-second rest period (fixation cross). The participant will be instructed to press a button before each series of three videos and then to watch the videos that follow (see Figure 6). For expert meditators, this rest-SoVT session will be performed in a neutral state (compared to the same task performed in different meditative states, as proposed in Session MRI3, see below). The series of neutral and emotional videos will be presented alternately and the order of presentation will be randomized across participants. In total, the test will last 17 minutes.

The total duration of the examination (anatomical acquisitions + fMRI) will be approximately 60 minutes.

At the end of the acquisition, the participant will be asked to leave the MRI and take back their personal belongings. Finally, each participant will have to watch again, this time outside the MRI, the 24 videos presented during the rest-SoVT task and judge for each of them how much empathy, positive emotions and negative emotions they felt.

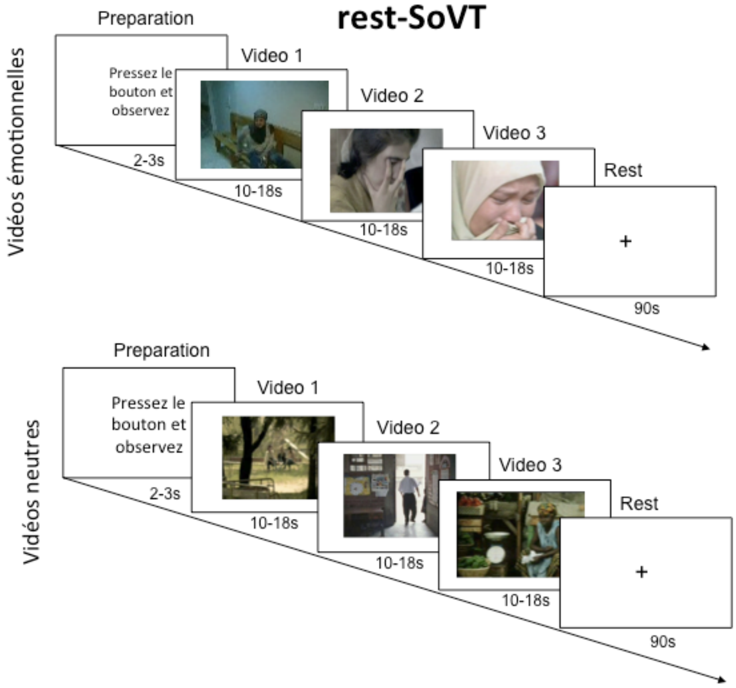

Figure 6: Representation of the different conditions of the rest-SoVT task

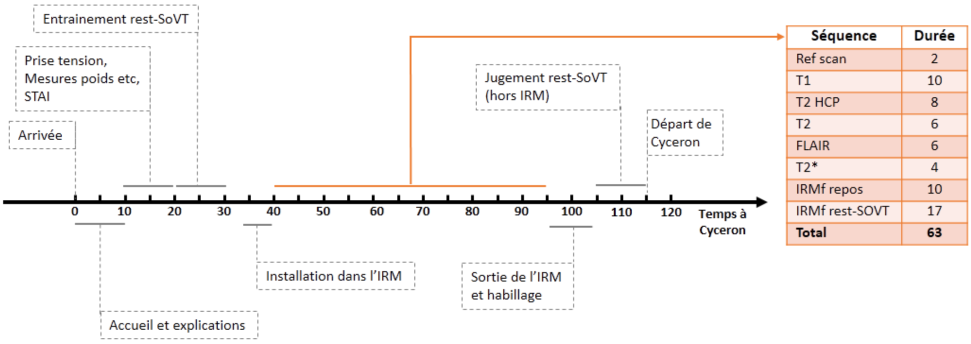

Figure 8: MRI 1 examination procedure (the time spent in the MRI is shown in orange with a total duration at the Cyceron center ≈ 120 min)

MRI2 session

The procedure for the second MRI session is shown in Figure 9. Upon arrival at the Cyceron center, the participant will be greeted by their referent, who will remind them of the upcoming examination. As with MRI1, the participant will be asked to complete a self-anxiety questionnaire (STAI-A) and a blood pressure measurement will be performed. Participants will then be familiarized with the AX-CPT task, proposed in fMRI during this second MRI session. A short training session on the computer will be proposed to ensure that the instructions are well understood. Then, the participant will be invited to put on pyjamas (leaving clothes and possible jewels, glasses or dental/auditory devices in a secured locker room) and to put earplugs (to attenuate the noise generated by the MRI) before being installed in the MRI by the radiology manipulators.

The details of the MRI sequences are shown in Figure 10 below. The session will start, after a baseline scan and a T2*-weighted sequence (necessary for fMRI preprocessing), with a functional acquisition with the AX-CPT task. During this attentional test, letters written in black or grey scroll on the screen. The participant is instructed to press a particular key when a black "X" (target) appears after a black "A" (cue). For all other letter combinations, he must press another key. Two conditions will be proposed: an easy condition and a difficult condition. In the easy condition, the attentional load is said to be low because no distraction appears between the target and the clue. In the difficult condition, the attentional resources are strongly engaged by the addition of distracting letters, written in grey, between the cue and the target. In 25% of the cases, the trials will be valid (letter "X" preceded by an "A" - "AX"), reflecting phasic alertness abilities. The remaining 75% of trials will be invalid. In 1/3 of the cases, these will be control trials where the letter "Y" has been preceded by a letter "B" ("BY" = invalid index and target), 1/3 will evaluate the inhibition abilities of the item where the letter "X" is preceded by a "B" ("BX" = invalid cue but valid target) and the last third of the trials will evaluate the inhibition abilities of the context where a "Y" will appear after a letter "A" ("AY" = valid cue but invalid target) A total of 200 trials will be offered (100 in the easy condition and 100 in the difficult condition), and for each condition the different types of trials will be presented randomly. Overall, the AX-CPT task will last approximately 23 minutes.

The session will continue with an anatomical acquisition of approximately 21 minutes including a diffusion sequence (DKI for Diffusion Kurtosis Imaging), a magnetic susceptibility sequence (QSM for Quantitative Suceptibility Mapping) and a perfusion sequence (ASL for Arterial Spin Labeling).

In total, the acquisition time of the MRI2 session should be about 60 minutes.

At the end of the acquisition, the participant will be invited to leave the MRI, to collect his personal belongings. They will then be able to leave.

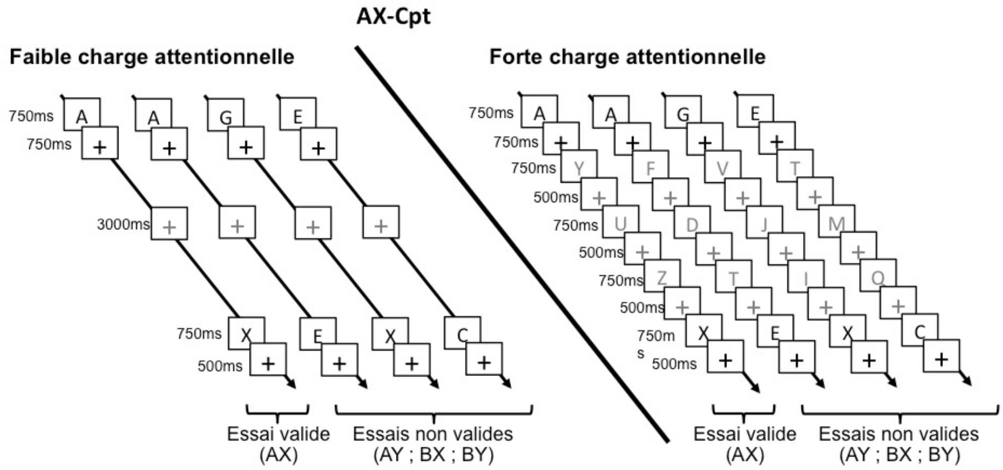

Figure 9: Representation of the different conditions of the AX-CPT task

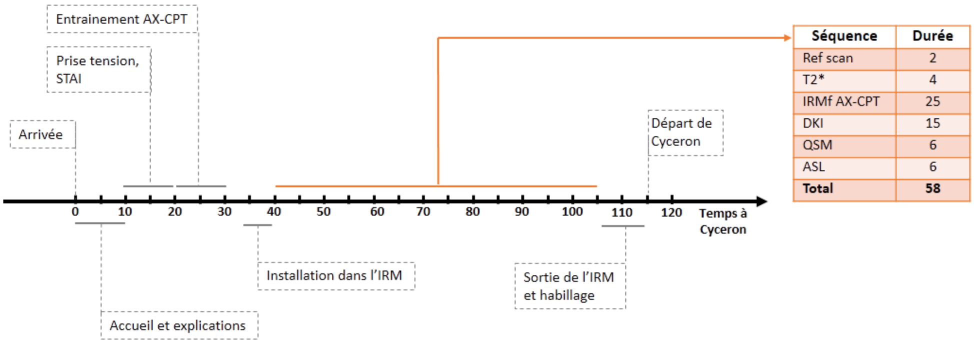

Figure 10: MRI2 examination procedure (time spent in the MRI is shown in orange with a total duration at the Cyceron center ≈ 120 min)

MRI3 session *(Experts only)*

This 3rd MRI session is only offered to meditative experts. Upon arrival at the Cyceron center, the participant will be greeted by their referent, who will remind them of the upcoming examination. As in other sessions, the participant will be asked to fill out a self-anxiety questionnaire (STAI-A) and a blood pressure measurement will be taken. During this MRI session, the participant will be familiarized with the rest-SoVT task, and will then be asked to put on pajamas (leaving clothing and any jewelry, glasses or dental/auditory appliances in a secure locker room) and put in earplugs (to attenuate the noise generated by the MRI) before being installed in the MRI by the radiology manipulators.

The details of the MRI sequences are shown in figure 11 below. During this session, fMRI sequences will be acquired. The session will start with a first fMRI sequence at rest, for which no specific instructions will be given. A second fMRI sequence at rest will then be proposed and the participant will be asked to perform it in a state of mindfulness meditation if the same sequence was performed in a state of compassionate meditation during the MRI2, and vice versa for the second half of the participants. Subsequently, the participants will be asked to perform the rest-SoVT task again, (see MRI1 session) twice, in a different meditation state. Half of the participants will first perform the task in a mindfulness meditation state and then in a compassionate meditation state. The other half of the participants will perform these tasks in the other order (compassionate meditation then mindfulness). Each resting fMRI sequence will last 10 minutes and each activation fMRI sequence (rest-SoVT) 17 minutes.

Overall, the acquisition time for this last session will be approximately 60 minutes.

At the end of the acquisition, the participant will be invited to leave the MRI and take back his personal belongings. Finally, as for the MRI1 session, each participant will have to watch again, this time outside the MRI, the 48 videos presented during the two rest-SoVT tasks and judge for each of them how much they experienced empathy, positive emotions and negative emotions.

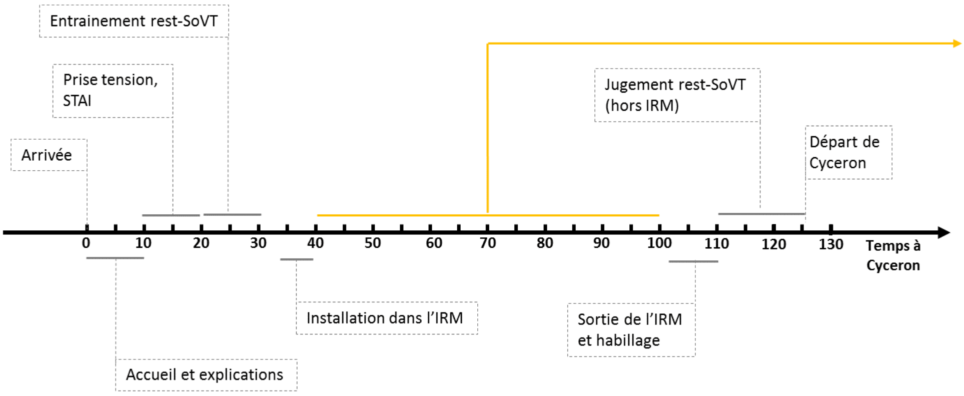

Séquence	Durée
Ref scan	2
T2*	4
IRMf repos	10
IRMf repos (med)	10
IRMf rest-SOVT (med)	17
IRMf rest-SOVT (med)	17
**Total**	**60**

Figure 11: MRI3 examination procedure (time spent in the MRI is shown in yellow with a total duration at the Cyceron center ≈ 130 min)

PET brain imaging examinations

Amyvid®-PET examination

The Amyvid®-PET examination will allow to evaluate both cerebral perfusion and the presence of beta-amyloid deposits. The delivery of the Amyvid® tracer will be ensured by the Lilly laboratory and the examination will be carried out under the responsibility of the nuclear physician, present at the Cyceron center. This examination does not imply any particular constraint (no need to fast, no

contraindication related to metallic material, make-up, etc.). The only constraints are the obligation to carry out the examination in the afternoon (linked to product delivery constraints) and recommendations for the period following the examination, namely the need to hydrate well (facilitates the elimination of the radiopharmaceutical) and to avoid contact with children or pregnant women in the 24 hours following the administration of the Amyvid®.

The investigating physician will use the information from the screening visit and the biological sample to identify the participants who will be able to perform the Amyvid®-PET examination.

Upon arrival at the Cyceron Center, the participant will be greeted by their referrer who will briefly remind the participant of the procedure (see Figure 12). The participant will then be taken care of by the radiology manipulators, who will insert a peripheral venous catheter for the injection of the radiopharmaceutical before installing the participant in supine position on the PET camera bed.

After the acquisition of an attenuation scan, the intravenous injection of 4 MBq/kg of Amyvid® will be performed as a bolus, followed by a rinse with 9 mg/mL (0.9%) sodium chloride injection; the proper acquisition of the PET images will begin at the time of the injection and will last 10 minutes, in 10 one-minute frames. Following this early frame acquisition, the participant will exit the PET camera and be allowed to rest, while remaining in the PET examination area, for approximately 30 minutes. After these 30 minutes, the radiology manipulators will reinstall the participant under the PET camera to proceed with a second attenuation scan followed by a new 10-minute PET image acquisition, starting at 50 minutes post-injection ("late frame").

When leaving the camera, the participant will be reminded to hydrate well (facilitates the elimination of the radiopharmaceutical) and to avoid contact with children or pregnant women in the 24 hours following the administration of Amyvid® (information present in the information document and recalled orally).

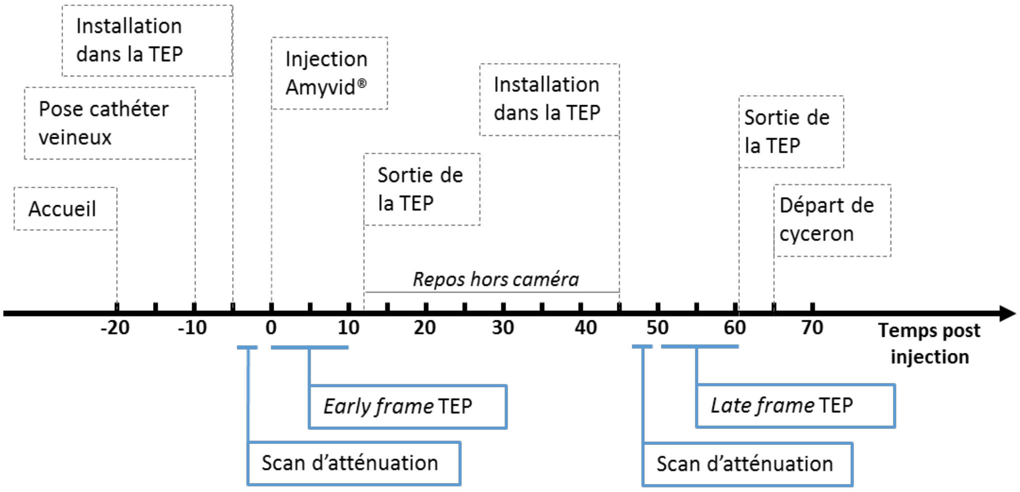

Figure 12: Amyvid®-PET examination procedure (time spent in the PET camera is shown in blue (total time at the Cyceron center ≈ 90 min)

18F-FDG-PET examination

The ^{18}F-FDG-PET scan will allow the quantification of the cerebral glucose consumption and therefore the estimation of the resting metabolism. The only constraints of this examination are the obligation to perform the examination on an empty stomach for at least 4 hours and recommendations for the period before and after the examination, namely the need to hydrate well (facilitates the elimination of the radiopharmaceutical), to empty the bladder and to avoid contact with children or pregnant women in the 24 hours following the administration of the radiopharmaceutical.

The investigating physician will use the information from the screening visit to identify the participants who will be able to perform the ^{18}F-FDG-PET scan.

The order and delivery of the radiotracer Glucotep® or ^{18}F-FDG will be done with the Cyclopharma laboratories under the responsibility of the Cyceron center and will be delivered by a transport company authorized for this type of radioelements. The examination will be carried out under the responsibility of the nuclear physician, present at the Cyceron center.

Upon arrival at the Cyceron Center, the participant will be greeted by their referent who will remind them of the procedure (see Figure 13 below). The participant will then be taken care of by the radiology manipulators, who will insert a venous catheter before injecting 200 MBq of ^{18}F-FDG in bolus. The participant will remain at rest. Approximately 40 min after the injection, the radiology manipulators will place the participant in the supine position on the PET-CT camera bed and perform an attenuation scan. 50 min after tracer injection, PET data will be acquired for a duration of 10 minutes.
When leaving the camera, the participant will be reminded to hydrate well (facilitates the elimination of the radiopharmaceutical drug) and to avoid contact with children or pregnant women in the 24 hours following the administration of the radiopharmaceutical drug (information present in the information document and recalled verbally).

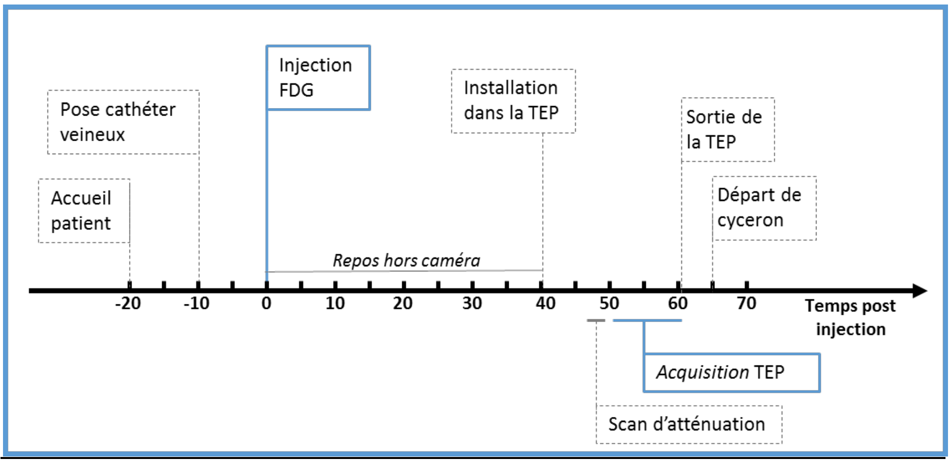

Figure 13: Sequence of the ^{18}F-FDG-PET scan (time in the PET camera is shown in blue (total time at the Cyceron center ≈ 80 min).

Neuropsychological and behavioural assessment on V1

The neuropsychological and behavioural assessment includes a set of paper-and-pencil and computerized tests, as well as a set of questionnaires (paper-and-pencil, computerized), listed in the tables below. This will allow for a detailed cognitive assessment as well as an assessment of psycho-emotional, personality, compassionate, etc. characteristics and lifestyle (social, cognitive and physical

activity and diet) through the questionnaires. This evaluation will be carried out at the Cyceron center (Caen) during two test sessions. Self-questionnaires will also be completed by each participant outside of these testing sessions.

Test sessions 1 and 2

Each participant will be welcomed at the Cyceron center by their referent or an investigating psychologist in order to perform a set of cognitive tests, detailed in table 5. For the inclusion visit (see table 5), the set of tests and some questionnaires will be spread over 2 sessions of about 2 hours, during which breaks are planned.

		Neuropsychological Evaluation (session 1 & 2 on site)								
**Evaluated functions**	**Test**	**Organi-zation at Cyceron**	**Score(s) collected**	**Reference**	**Expert meditators**	**Non-expert seniors**				**Conditions for completing the questionnaires**
		**Session**			**V1 Inclusion**	**V1 Inclusion**	**V2 9 months**	**V3 18 months**	**V4 29 months post-intervention**	
**Global cognitive functioning**	*Mattis Dementia Rating Scale*	S1	Multiple	*Mattis, 1976*	x	x		x	X	*-- With a neuropsychologist - At the center - on paper*
**IQ**	*Matrices (Matrix Reasoning)*	S1	Multiple	*WAIS*	x			x	X	*- With a neuropsychologist - At the center - on paper*
	Vocabulary	S1	Multiple	*WAIS*	x			x	X	*- With a neuropsychologist - At the center - on paper*
**Attention / Executive functions**	*Flanker task with spatial cueing*	S1	Multiple	*Trautwein, 2016*	x	x	x	x	X	*- With a neuropsychologist - At the center - on paper*
	Stroop	S1	Multiple	*GREFEX 2008*	x	x	x	x	X	*- With a neuropsychologist - At the center - on paper*
	Codes - Digit Symbol Substitution	S1	Multiple	*WAIS*	x	x	x	x	X	*- With a neuropsychologist - At the center - on paper*
	Trail Making Test A & B	S1	Multiple	*GREFEX 2008*	x	x	x	x	X	*- With a neuropsychologist - At the center - on paper*
	D2R - Selective attention	S2	Multiple	*Brickenkamp, Liepman, & Schmidt, 2015*	x	x		x	X	*- With a neuropsychologist - At the center - on paper*
	Empan endroit - Digit Span Forward	S2	Multiple	*WAIS*	x	x		x	X	*- With a neuropsychologist - At the center - on paper*
	Empan envers - Digit Span Backward	S2	Multiple	*WAIS*	x	x		x	X	*- With a neuropsychologist - At the center - on paper*
**Verbal episodic memory**	*California Verbal Learning*	S1	Multiple	*Delis et al, 2000*	x	x		x	X	*- With a neuropsychologist - At the center*

	Test-Iversion A&B-5 learning trials, short-term recall									- on paper
	California Verbal Learning Test-II version A&B-5 learning trials, long-term recall	S1	Multiple	Delis et al, 2000	x	x		x	X	- With a neuropsychologist<br>- At the center<br>- on paper
	Mémoire logique paragraphe 1 -Short-term recall	S2	Multiple	MEM	x	x		x	X	- With a neuropsychologist<br>- At the center<br>- on paper
	Mémoire logique paragraphe 1 - Long-term recall	S2	Multiple	MEM	x	x		x	X	- With a neuropsychologist<br>- At the center<br>- on paper
**Visual episodic memory**	Visual Object Separation Task	S1	Multiple	Yassa et al., 2010	x	x	x	x	X	- With a neuropsychologist<br>- At the center<br>- On computer
**Autobiographical memory**	Fluences autobiographiques - Autobiographical Fluency (Episodic & Semantic)	S2	Multiple	Piolino et al., 2002	x	x		x	X	- With a neuropsychologist<br>- At the center<br>- on paper
**Language**	Orthographic verbal fluency: P	S1	Multiple	GREFEX 2008	x	x	x	x	X	- With a neuropsychologist<br>- At the center<br>- on paper
	Categorical verbal fluency: animals	S1	Multiple	GREFEX 2008	x	x	x	x	X	- With a neuropsychologist<br>- At the center<br>- on paper
**Emotion**	State-Trait anxiety Inventory (STAI)** et	S1	Unique	Spielberger al, 1983	x	x	x	x	X	- With a neuropsychologist<br>- At the center, by phone<br>- On the tablet
	Geriatric Depression Scale (GDS) - 15 item**	S1	Unique	Sheikh & Yesavage, 1986	x	x	x	x	X	- Autoquestionnaire<br>- At the center<br>- on paper
	Interpersonal Reactivity Index, IRI	S2	Multiple	Davis, 1983	x	x	x	x	X	- Autoquestionnaire<br>- At the center<br>- on paper
	Positive and negative affect, PANAS-NOW	fMRI	Multiple	Watson, Clark, & Tellegen, 1988	x	x	x	x	X	- Autoquestionnaire<br>- At the center<br>- on paper
	Cyberball task (Pre-Cyberball and post-Cyberball)*	S2	Multiple	Williams, Cheung & Choi, 2000	x			x		- With a neuropsychologist<br>- At the center<br>- On computer
**Mental Imaging**	2D - Mental Rotation Test	S2	Multiple	Adapté de Shepard & Metzler, 1971	x	x		x	X	- With a neuropsychologist<br>- At the center<br>- On computer
	Visual Mental Imaging Battery	S2	Multiple	Adapté de Bourlon et al., 2009	x	x		x	X	- With a neuropsychologist<br>- At the center<br>- On computer

**Altruistic behavior**	*Empathic Dictator**	S2	Multiple	*Klimecki et al., (en révision)*	x			x		*- With a neuropsychologist<br>- At the center<br>- On computer*
**Personality**	*The Big Five Inventory*	S1	Multiple	*John, 1991*	x	x	x	x	X	*- Autoquestionnaire<br>- At the center<br>- on paper*
**Lifestyle**	*Questionnaire sur les expériences passées - The Lifetime of Experiences Questionnaire, LEQ*	S1	Multiple	*Valenzuela, Sachdev, Psychol.Medi, 2007*	x	x				*-Autoquestionnaire<br>- At the center<br>- on paper*
	Modifiable Activities Questionnaire	S1	Unique	*Kriska, et al., 1990*	x				x	*- With a neuropsychologist<br>-At the center<br>-On computer or paper**
	Questionnaire about marital status	S2	Multiple		x				x	*- With a neuropsychologist<br>-At the center<br>-On computer or paper**
	Questionnaires about lockdown periods	S2	Multiple		x				x	*- With a neuropsychologist<br>-At the center<br>-On computer or paper**
**Emotion**	*PTQ (Perseverative Thinking Questionnaire)*	S1	Multiple						x	*- With a neuropsychologist<br>-At the center<br>-On computer or paper**
**Intervention**	*English test*	S2	Simple						X	*- With a neuropsychologist<br>-At the center<br>-On computer or paper**
**Emotion**	*ICG: Inventory of Complicated Grief*	S2	Multiple						X	*- With a neuropsychologist<br>-At the center<br>-On computer or paper**

Table 5: Neuropsychological tests offered as part of the neuropsychological and behavioural assessment and conducted during the various on-site testing sessions
** the Cyberball and Empathic Dictator tasks will be proposed at inclusion (V1) for expert meditators only; they will be performed by the other participants (non-experts) only at the end of the intervention (V3) and at 29 months post-intervention (V4)*
*** these questionnaires will also be offered to the participant's designated partner as "hetero-questionnaires"*

Autoquestionnaires

The neuropsychological and behavioural evaluation will also include a set of questionnaires (available on paper and electronic versions), not requiring the presence of a referent or an investigating psychologist, which will be carried out by the participant outside of the test sessions; either before or after certain examinations (paper or computerized version, as desired), or at home (paper version in this case). The set of questionnaires proposed at the inclusion visit is presented in Tables 5 and 6. The partners of the participants will also have questionnaires to fill in (also shown in tables 5 and 6), which they will complete in paper form only.

					Complementary neuropsychological evaluation (site questionnaires)					
**Domains evaluated**	**Questionnaire**	**Organization**	**Score(s) collected**	**Reference**	**Expert meditators**	**Non-expert seniors**				**Conditions for completing the questionnaires**
					**V1 Inclusion**	**V1 Inclusion**	**V2 9 months**	**V3 18 months**	**V4 29 months post-intervention**	
**Medical history**	*Drug list and cardiovascular factors*	AHQ	Multiple	*Adaptated from Roulet et al., 2012; Recommendations from National Insitute of Alcohol Abuse and Alcoholism*	x	x		x	X	*- Autoquestionnaire<br>- At home<br>- On computer or paper **
**Quality of life & well-being**	*McNair Cognitive Difficulties Scale*	AHQ	Multiple	*McNair and Kahn, (1983) Self-assessment of cognitive deficits*<br>*Cognitive Difficulties Scale*	x	x		x	X	*- Autoquestionnaire<br>- At home<br>- On computer or paper **
	Well-being (7-items version)	AHQ	Multiple	*Ryff & Keyes, 1995*	x	x	x	x	X	*- Autoquestionnaire<br>- At home<br>- On computer or paper **
	Three-Item Loneliness Scale	AHQ	Unique	*Adaptated from Revised UCLA Loneliness Scale, Hughes et al., 2004*	x	x	x	x	X	*- Autoquestionnaire<br>- At home<br>- On computer or paper **
	Questionnaire de Qualité de vie – Quality of Life, WHOQOL	AHQ	Multiple	*World Health Organisation, 1996*	x	x		x	X	*- Autoquestionnaire<br>- At home<br>- On computer or paper **
**Compassion, support, meditation**	*Trait Mindfulness: Five-Facet Mindfulness Questionnaire (FFMQ-15)***	AHQ	Multiple	*Baer et al. 2012*	x	x	x	x	X	*- Autoquestionnaire<br>- At home<br>- On computer or paper **
	Drexel Defusion scale	AHQ	Unique	*Forman, 2012*	x	x	x	x	X	*- Autoquestionnaire<br>- At home<br>- On computer or paper **
	Multidimensional Assessment of Interoceptive Awareness (MAIA)	AHQ	Multiple	*Mehling et al. (2012)*	x	x	x	x	X	*- Autoquestionnaire<br>- At home<br>- On computer or paper **
	Depression Death Scale Revised (21 items)	AOS	Multiple	Templer et al., 2012	x	x		x	X	*- Autoquestionnaire<br>- At the center<br>- On computer or paper **
	Self-compassion-short version	AHQ	Unique	*Raes et al., 2011, adapté de Neff, 2003*	x	x	x	x	X	*- Autoquestionnaire<br>- At home<br>- On computer or paper **
	*Other Compassion ***	AHQ	Unique	*Pommier, 2011*	x	x	x	x	X	*- Autoquestionnaire<br>- At home<br>- On computer or paper **

	*The COPE index***	AHQ	Multiple	*McKee et al. 2003*	x	x	x	x	X	*- Autoquestionnaire<br>- At home<br>- On computer or paper **
	*MOS Social Support Survey***	AHQ	Multiple	*Sherbourne & Steward 1991*	x	x	x	x	X	*- Autoquestionnaire<br>- At home<br>- On computer or paper **
	*Prosocialness scale ***	AOS	Unique	*Caprara et al., 2005*	x	x	x	x	X	*- Autoquestionnaire<br>- At the center<br>- On computer or paper **
**Emotion**	*Penn State Worry Questionnaire - short*	AHQ	Unique	*Meyer et al 1990*	x	x	x	x	X	*- Autoquestionnaire<br>- At home<br>- On computer or paper **
	Emotion regulation abilities	AHQ	Multiple	*Gross & John, 2003*	x	x	x	x	X	*- Autoquestionnaire<br>- At home<br>- On computer or paper **
	Rumination Response Scale	AHQ	Multiple Unique	*Treynor, Gonzalez, & Nolen-Hoeksema, 2003*	x	x	x	x	X	*- Autoquestionnaire<br>- At home<br>- On computer or paper **
	PSS-14 (Perceived Stress scale)	AHQ	Unique	*Cohen et al., 1983*	x				x	*- Autoquestionnaire<br>- At home<br>- On computer or paper **
	Satisfaction with life	AOS	Unique	*Diener, Emmons, Larsen, & Griffin, 1985*	x	x	x	x	X	*- Autoquestionnaire<br>- At the center<br>- On computer or paper **
	UCLA	AOS	Unique	*Russel et al., 1980*	x				x	*- Autoquestionnaire<br>- At the center<br>- On computer or paper **
	Survey on mood, emotions, stress	AOS	Multiple	*Adaptated from A Giersch lockdown questionnaire*	x				x	*- Autoquestionnaire<br>- At the center<br>- On computer or paper **
	Attentional Style Questionnaire	Pre-PET	Multiple	*Van Calster, D'Argembeau & ajerus, 2016*	x	x		x	X	*- Autoquestionnaire<br>- At the center<br>- On computer or paper **
**Lifestyle**	*AUDIT = Alcohol use disorders test*	AHQ	Multiple	*Schwarzinger et al., 2018*				x	x	*- Autoquestionnaire<br>- At home<br>- On computer or paper **
	Adapted CAQ and LEQ	AOS	Multiple	*Adaptated from Valenzuela, Sachdev, Psychol.Medi, 2007 and Wilson et al., 2003*			x	x	X	*- Autoquestionnaire<br>- At the center<br>- On computer or paper **
	Social Network Index	AOS	Multiple	*Adaptated from A. Giersch lockdown questionnaire*	x				x	*- Autoquestionnaire<br>- At the center*

										- On computer or paper *
	Place of Living Questionnaire	AOS	Multiple		x				x	- *Autoquestionnaire*<br>- *At the center*<br>- *On computer or paper **
	Questionnaire d'activité physique - Modifiable Activity Questionnaire, MAQ	AHQ	Unique	*Kriska et al., 1997*<br>*Vuillemin et al., 2000*	x	x			X	- *Autoquestionnaire*<br>- *At home*<br>- *On computer or paper **
	Adherence to the Mediterranean diet	AHQ	Unique	*Schröder et al., 2011*	x	x		x	X	- *Autoquestionnaire*<br>- *At home*<br>- *On computer or paper **
	The Physical Activity Scale for the Elderly	Pre-PET	Multiple	*Washburn et al., 1992*		x	x	x	X	- *Autoquestionnaire*<br>- *At home*<br>- *On computer or paper **
	Questionnaire des activités cognitives - Cognitive activities questionnaire)	Pre-PET	Multiple	*Wilson et al., 2003*	x	x			X	- *Autoquestionnaire*<br>- *At the center*<br>- *On computer or paper **
	Activity Control Questionnaire	AOS	Multiple	-			x			- *Autoquestionnaire*<br>- *At the center*<br>- *On computer or paper **
	Stressful Events questionnaire (SEQ)	AHQ	Multiple	*Adaptated from Smith et al., 2011*				x		- *Autoquestionnaire*<br>- *At home*<br>- *On computer or paper **
	Childhood Trauma Questionnaire (CTQ)	AHQ	Multiple	*Paquette et al., 2004*				x		- *Autoquestionnaire*<br>- *At home*<br>- *On computer or paper **
Sleep	*Leeds Sleep evaluation questionnaire*	AOS	Multiple	*Parrott et al., 1980*				x	X	- *Autoquestionnaire*<br>- *At the center*<br>- *On computer or paper **
	Pittsburg Sleep Quality Index<br>*Mois dernier*	AHQ	Multiple	*Buysse et al., 1981*	x	x		x	X	- *Autoquestionnaire*<br>- *At home*<br>- *On computer or paper **
	St Mary's hospital questionnaire	A&P	Multiple	*Ellis et al., 1989*	x	x		x	X	- *Autoquestionnaire*<br>- *At home*<br>- *On computer or paper **
	Insomnia severity index	A&P	Unique	*Bastien et al., 2001*	x	x		x	x	- *Autoquestionnaire*<br>- *At home*<br>- *On computer or paper **
	Sleep quality questionnaire (last 5 years)	A&P	Multiple	*Adaptated from Buysse et al., 1989*	x	x		x	X	- *Autoquestionnaire*<br>- *At home*<br>- *On computer or paper **
	Echelle de Somnolence d'Epworth - Epworth Sleepiness Scale	A&P	Multiple<br>Unique	*Johns, 1991*	x	x		x	X	- *Autoquestionnaire*<br>- *At home*<br>- *On computer or paper **
	STOP-BANG Sleep Apnea Questionnaire	A&P	Unique	*Chung et al., 2008*	x	x		x	X	- *Autoquestionnaire*<br>- *At home*<br>- *On computer or paper **

	Retrospective sleep questionnaire	A&P	Multiple	-	x					*- Autoquestionnaire<br>- At home<br>- On computer or paper **
Evaluation of practice (foreign language and meditation)	*Long-term post-intervention practice questionnaire*	AOS	Multiple	-					X	*- Autoquestionnaire<br>- At the center<br>- On computer or paper **
	Interview about practice experience	OS	Multiple	-				x		*- With evaluator<br>- At the center<br>- On paper<br>- For meditation and English teachers as well*

Table 6: Self-questionnaires offered as part of the neuropsychological and behavioural assessment and to be completed by the participant outside of the testing sessions conducted on site (≈ 180 min divided into several rounds of questionnaires)

*** these questionnaires will also be offered to the participant's designated partner as "hetero-questionnaires"*

Sleep assessment

An assessment of sleep will be performed through both subjective measures (questionnaires, consolidation task) and objective measures (actimetry, Somno-Art and polysomnography).

Sleep questionnaires

Subjective measures of sleep will be collected through a set of questionnaires to be completed by the participant, listed in Tables 5 and 6 (see above). Specifically, these questionnaires include the Pittsburgh Sleep Quality Index (Buysse et al., 1981), which assesses sleep over the previous month in its original version and a version adapted for assessment over the past five years and for the previous week; the Saint Mary's Hospital Questionnaire (Ellis et al, 1981), which assesses overnight sleep and will be offered in conjunction with polysomnography (see below), Epworth Sleepiness Scale, Leeds Sleep Assessment Questionnaire, and the Insomnia Severity Index (ISI, Bastien et al., 2001).

Consolidation task

We will explore the consolidation of visual-spatial learning during sleep. To this end, we will propose a memory task consisting of memorizing pairs of cards (task based on the principle of the Memory game; Rasch et al., 2007; Cherdieu et al., 2013). Learning will be performed in the evening, and will be followed by immediate and delayed recall, after the night of sleep.

We will collect the number of correctly retrieved pairs of cards after learning and after the night of sleep, in order to compute a rate of improvement or forgetting during the night.

Actimetry

Participants will wear an actimeter, a type of watch worn on the wrist of the non-dominant hand, for a period of one week. This device has a piezoelectric sensor that detects movement accelerations. It thus allows a long-term recording of the activity-rest cycle. Different parameters can be extracted from

this recording and analyzed: average sleep duration, fragmentation index of activity and rest periods, regularity of the activity/rest cycle…

The actimeter will be given to the participant during the inclusion visit by his referent. The participant will have to wear it during his daily activities during one week, at the end of which he will have to give the actimeter back to the referent.

Polysomnography

Two home sleep recordings will be performed for participants willing to perform this examination, using a portable device (Siesta, Compumedics, Australia). This examination consists of a continuous recording of an EEG, an electro-oculogram and an electromyogram performed on the chin muscles, and an electrocardiogram. EEG activity will be recorded at prefrontal, central, parietal, temporal and occipital leads according to the international 10-20 system, using Ag/AgCl electrodes with a ground at the vertex and a bi-mastoid reference. We will also record the ventilatory parameters in order to detect a possible sleep apnea syndrome and to quantify the apnea-hypopnea index. The realization of a polysomnography will allow the detection of a possible sleep apnea syndrome.

The interpretation of the respiratory recording collected during the polysomnography examinations is done by an expert, by visual scoring. The initial scoring of apneas and hypopneas was based on the criteria used in clinical practice and proposed by the Société de Pneumologie de Langue Française in 2010. However, the international criteria used in research in scientific publications are those of the American Academy of Sleep Medicine (AASM), revised in 2017. Consequently, we will calculate this scoring according to these new rules.

Given the already known cardiovascular risks and the potential impact on amyloid pathology and cognitive functions, it is important to be able to detect and treat this syndrome as early as possible in the older populations. Moreover, an effective treatment of this pathology is available (treatment by continuous positive pressure). In this respect, the trial brings a direct individual benefit to the participants.

The sleep recording takes place at the participant's home. The installation of the device requires the participant to come to the PFRS before and after the night of recording. The electrodes will be placed on the scalp of the participant at the end of the day. At this time, the resting activity of the seated participant will be recorded for 1 minute with the eyes open, then for 8 minutes with the eyes closed, then for 1 minute with the eyes open. After that sounds at different frequencies will be presented for about 10 minutes (oddball paradigm, or discordant stimulus, to measure the potential for discordance negativity or MMN, see 1.2.9 and Ruzzoli et al. 2016). The participant will simply have to remain at rest and not fall asleep. A vigilance scale (Stanford Vigilance Scale, Dement et al. 1972) will be given to the participant at the beginning and end of the data collection. The participant can then go home. The next morning, the participant will return to the PFRS to remove the electrodes and retrieve the recording. The participant will be asked to come accompanied to the appointments for the placement and removal of the electrodes.

The fact of being equipped for polysomnography can induce stress and disturb the sleep of the participants (phenomenon known as the "first night" effect). We will therefore perform a first night of

habituation which will allow the volunteers to adapt to the presence of the different sensors and electrodes and to be able to sleep as usual during the second night. In practice, the night of habituation and the night of recording will be carried out at approximately one week intervals, according to the availability of the volunteers. The installation of the electrodes and sensors will be done in the same way as described for the recording night. The subjects will have to come accompanied to the PFRS and will not be able to leave alone.

Acquisition of objective data using the Somno-Art® device

We will complete the sleep explorations already performed by adding 5 nights of recordings using the Somno-Art® device (PPRS; https://www.somno-art.com/#home). This examination does not replace the actimetric recording. The Somno-Art® device is based on the measurement of two physiological parameters, heart rate and motor activity, to discriminate between sleep and wakefulness, and to distinguish the different stages of sleep and their transitions. Scoring algorithms are then applied to the data in order to establish the hypnogram (i.e. the course of the night, divided into 30-second periods). The results obtained with this device show very good agreement with the visual interpretation of sleep tracings by an expert physician in subjects aged 18 to 40 years (Muzet et al., 2016). The advantage of Somno-Art is that it allows for recordings on multiple consecutive nights, as sleep variability from night to night is potentially predictive of cognitive decline (Westerberg et al., 2010). Moreover, the use of the device is very simple and much less cumbersome than the realization of a polysomnography.

In practice, the subjects will have to wear the Somno-Art device for 5 consecutive nights. The device takes the form of a large bracelet, resembling a blood pressure monitor. It must be placed on the upper forearm. The device must be placed at bedtime and removed when waking up. Subjects will need to recharge it after each use. In order to verify the good concordance between the acquired data and the polysomnography data, the recording period (i.e., 5 nights) will include the second night of polysomnography. The data will be collected anonymously and sent to the PPRS company with the corresponding polysomnography for analysis.

At the end of the inclusion visit, and once it has been verified that none of the results of the various examinations constitute a criterion for non-inclusion, the non-expert seniors will be randomized between the different intervention groups (meditation, English, no intervention) while, for the meditating experts, the end of this inclusion visit will correspond to the end of the trial.

6.7. *Randomization* (Non-expert seniors only)

Once all inclusion visit examinations have been completed for the entire wave (i.e., 42 participants for Wave 1 and 54 participants for Waves 2 and 3), participants will be contacted by telephone by one of the protocol investigators to confirm the planned start date of the intervention (Visit 2) within the next 2 weeks and to ensure that the participant still wants to participate in the trial prior to randomization. If the participant confirms their attendance, then the investigator can proceed to the randomization of the participant. To do so, they log into the e-CRF, complete the "randomization" page with the eligibility criteria and validate the content. The site immediately displays the group allocated to this participant.

If unable to connect to the e-CRF, the investigator will contact the Euclid platform.

The result of this randomization will be communicated to all participants in the same session at the same time shortly before the start of the intervention in order to limit the risk of participants dropping out if they were not assigned to the group they wanted (although this risk should be limited as early as the participant selection phase). Information will be communicated both by telephone and by mail (including important information about the first sessions of the intervention programs for participants assigned to the meditation and foreign language learning groups).

6.8. *18-month intervention*

As mentioned above (6.7. Visit 1 - Inclusion Visit), the inclusion of the non-expert senior participants will be conducted in three waves of participants (divided into three arms). The waves will be spaced approximately 6 months apart. Interventions will therefore also begin in three waves spaced approximately 6 months apart, and all intervention groups will consist of 14 participants.

Participants will be asked not to engage in the proposed activity in the arms to which they have not been assigned for the duration of the intervention. Participants assigned to the meditation group and the foreign language group will have a standardized intervention program following a well-defined plan described in a detailed manual (for the meditation intervention) or following a specific program (for the foreign language intervention) lasting 18 months. Both interventions will be delivered by experienced and motivated teachers. The formats of the interventions will be identical in terms of the volume and frequency of sessions, home practice, and group exchange periods. The number of teachers per class and their level of expertise in delivering these programs will be identical between the interventions.

Thus, for both types of intervention, participants will have:

- Group classes of 2 hours, once a week,
- Exercises to be done every day for at least 20 minutes,
- More intensive days and/or half-days of practice.

In each weekly group session, there will be a time for presentation, a time for sharing, and a time for practice; the first two sessions of each month will include an equal amount of these three aspects (3x40min), session 3 will include more sharing (30/60/30min), and session 4 more practice (30/30/60). For each intervention, participants will be provided with supports (manual and audio) for their practice. The alternation of supports (texts, images, audio, video) and activities (alone, in pairs, in groups) will contribute to maintain interest and motivation.

Participants will be strongly encouraged to participate in all of these activities over the course of the intervention (18 months). In addition, they will be given a questionnaire at the beginning of the intervention to assess their expectations of the intervention (Credibility/Expectancy questionnaire, Devilly et al., 2000).

Monthly meetings will be organized between the scientific investigators and the teachers of the two interventions in order to ensure the best possible follow-up. In case of specific problems, the teachers in charge of the intervention will immediately contact the scientific investigators in order to keep them informed and to ensure optimal care and follow-up of the participants.

In addition, participants will be asked to fill in a "diary" throughout the intervention to indicate the exercises performed and to assess whether they are difficult or pleasant (see also 5.3). Indeed, in order to quantify the daily practice of the participants throughout the trial and to allow us to analyze in a more detailed way the impact of the inter-individual differences of daily practice on the primary and secondary variables of the trial on the one hand, and to allow the participants to use the pedagogical material (audios) used in the interventions on the other hand, an Ipad tablet is lent to the participants during their entire participation in the trial. The functionalities of the Ipad which are not required for the trial have been deactivated (Internet...). The latter contains an application for an Ipad 2 tablet, containing a questionnaire of 6 daily questions and developed internally by Antoine Lutz's team at the Centre de Recherches en Neurosciences de Lyon, Inserm. The 6 questions intended for the subjects of the English and meditation groups are composed of 4 multiple choice questions and 2 open questions. The first question concerns the well-being of the person (the choice of answers is presented in the form of a table in which the rows represent different intensities (very big, big, small, very small) and the columns different weather images (sun, sun and small cloud, sun and big cloud, cloud, rain, flash cloud, double flash). The second question asks about formal practice time for the day (0 min, less than 15 min, between 15 and 30 min, between 30 and 45 min, between 45 and 60 min, between 60 and 90 min, between 90 and 120 min, over 120 min). Formal practice is defined as practice related to the instructions, exercises, or materials (books and applications) of the course for the English intervention. For the meditation group, formal practice is defined as practicing meditation exclusively without doing another activity at the same time. The third question asks about the duration of their informal practice (0 min, less than 15 min, between 15 and 30 min, between 30 and 45 min, between 45 and 60 min, between 60 and 90 min, between 90 and 120 min, more than 120 min). Finally, the fourth question asks about motivation to practice during the day (very low, low, medium, high, very high). The fifth question asks about the geographic area where the subject is located during the day (free text). The last question gives subjects the opportunity to leave comments on the intervention in general.

Participants assigned to the "passive control" group will not have an intervention during the 18 months. For these participants, the date of randomization will be considered as the date of the beginning of their follow-up.

6.8.1. « Meditation » intervention

The intervention in the meditation group will be delivered by meditation experts teachers and will take place at the Pôle de Formations et de Recherche en Santé (PFRS), in Caen, France. This intervention will consist of an original secular meditation training program offered from a perspective of personal development and successful aging. This eighteen-month program is intended for healthy retired people living at home. The goal of this program is to develop mindfulness and compassion as additional psychological resources for the physical, cognitive and psychological challenges of aging.

Mindfulness is the cultivation of a vigilant awareness of one's own thoughts, actions, emotions and motivations. The participant learns to intentionally pay attention to their internal or external experiences in the present moment, without making value judgments. Happy (mental calm, compassion) or unhappy (ruminations, destructive emotions) mental states are observed without the participant identifying with or being absorbed by these experiences. The present moment is thus experienced in a more open and flexible way and is less dominated by mental conditionings that are sources of suffering. Mindfulness-based psychotherapies are particularly effective for stress management and relapse prevention in depression. The mindfulness portion of the Medit-Ageing program will be directly adapted from an 8-month intervention developed and validated on a group of French-speaking older adults (Zellner Keller, Singh, and Winton 2014).

The practice of loving-kindness and compassion improves our relationship with ourselves and the world by addressing emotions such as shame, self-blame, or anger from a more positive perspective, and by developing gratitude and appreciation for positive experiences such as loving-kindness or compassion. The part of Medit-Ageing on compassion will be directly adapted from the developed Compassion-Focused Therapy (CFT), an integrated and multimodal psychotherapeutic approach developed in England by Prof. Paul Gilbert (Gilbert 2014), and adapted in France by Prof. Pascal Delamillieure, and Francis Gheysen at the University Hospital of Caen, France.

The pedagogical content of the meditation intervention will be structured in 9 months dedicated to teaching mindfulness meditation followed by 9 months dedicated to teaching caring and compassion meditation. A new teaching theme will be introduced each month in the first session and will then be explored, practiced, and discussed in the remaining sessions of the month. Each session contains moments of group meditation, sitting or walking, moments of sharing and moments of teaching. Annex 15 details the content of the first meditation session and its agenda, as an example. The content and agenda of the following sessions will be based on the same model. However, the teachers will adapt their teachings according to the feedback from the participants in group discussions and individual interviews.

The days and half-days will include moments of meditation and group exchange and will take place at a frequency of one every 3 to 4 months. They will last approximately 5 hours for the day and 3 hours for the half-day. They will be followed by a snack or a buffet.

6.8.2. « English learning » intervention

The intervention in the foreign language learning group (English) will be carried out by teachers from the Carré International, a department at the University of Caen dedicated to language learning and experience with the older people. This intervention will consist of English exercises aimed at reinforcing the skills of each participant in terms of written and oral comprehension and expression. A placement test will be offered during the first session to allow a precise evaluation of the initial level of each participant. The first session will also be devoted to the creation of a group spirit (introduction of each participant, commitment to participate in the sessions over the 18 months, rules of confidentiality, etc.).

Then the basics of English will be worked on (name, age, nationality, place of living, lifestyle, tastes, etc).
The other sessions will be a mix of activities that will allow the student to work on oral comprehension and expression, the acquisition of new vocabulary and new grammatical structures. A large part of the course will be devoted to reviewing the concepts covered in the previous courses.
Existing manuals will be used; these are methods that have been published and used in numerous training courses, particularly at the Carré International for several years.
Participants' progress will be evaluated using methods routinely used at the Carré International, including a training follow-up document and personalized or group assistance as needed.
The days and half-days will include similar exercises as well as games and artistic workshops where the emphasis will be on exchanges in English. A day on the Channel Island of Jersey will be organized where participants will have a mission to accomplish with informations to obtain on different places, objects to obtain (leaflets, ...) and objects to buy. The corresponding language needs will have been worked on beforehand.
Starting with the second wave, the foreign language learning group will be divided into 2 subgroups in order to respect the logistical constraints on group size. The allocation in each of the two English groups will be drawn at random via the eCRF randomization module and adapted by the English teachers if necessary from the level assessed during the English test carried out during the diagnostic battery, to limit the heterogeneity of level within the same group.

6.9. *Visit no.2 - Intermediate evaluation visit at 9 months*

Nine months after the start of the intervention, each participant (including the passive control group) will be seen for a first intermediate visit. For this visit, only a short neuropsychological and behavioural assessment will be performed.

Neuropsychological and behavioural assessment on V2

Each participant will be welcomed at the Cyceron center by their referent or an investigating psychologist to perform a set of cognitive tests. The battery of tests proposed will be shorter than the one proposed during the inclusion visit (see Table 5 above). All the tests and questionnaires will be carried out during the intermediate evaluation visit (V2) in a single session of about 2 hours, during which breaks will be scheduled.
The evaluators will not know the participants' group of assignment, who will be instructed not to communicate it.

6.10. *Visit no.3 – Follow-up visit*

At the end of the 18-month intervention, the participants will be welcomed back at the Cyceron center for a preliminary medical interview during which the physician will verify the absence of contraindication to the MRI examination (claustrophobia, ferromagnetic material in the body) and to the PET scan (known hypersensitivity to Amyvid® and Glucotep®), will update the participants' medical

history and drug treatments, and will declare any adverse events that may have occurred during this period. From the medical data collected during this interview, the comorbidity index will be calculated again (Charlson et al., 2008) as well as the CIRS score (see detailed description in V4). Then, the participants will be asked to perform again all the examinations of the inclusion visit (V1), namely:

- 2 MRI sessions,
- 2 PET Scans,
- a blood sample (3), conditioning the realization of the PET imaging to the same criteria as in V1 (cf 6.6),
- a detailed neuropsychological and behavioural assessment (V3),
- an examination of wakefulness (spontaneous EEG and MMN) and sleep.

Because some blood biomarkers and cognitive tests are not sensitive to longitudinal changes, they will not be repeated at this 18-month follow-up visit. Moreover, two behavioural tests (Cyberball (Williams et al., 2000) and Empathic Distractor (Klimecki et al., submitted); respectively evaluating social exclusion and empathy mechanisms), based on a surprise effect, can only be performed once and will be offered to non-expert seniors during this visit. A qualitative interview will be conducted with an evaluator experienced in conducting this type of interview (other than the neuropsychologists and members of the investigating team, this experimenter will be familiar with the intervention group). The open-ended semi-structured interview will last between 30 and 60 min and will be designed to collect data on the experience of learning English and meditation during their participation in the Age-Well trial. The V3 blood sample and the V3 neuropsychological and behavioural assessment are detailed in Tables 8 and 5 respectively.

The evaluators of the various examinations will not be aware of the participants' group assignment, and they will be instructed not to share it, except for the qualitative interview.

A qualitative interview of the meditation and English teachers will be conducted with an evaluator experienced in conducting this type of interview (other than the neuropsychologists and members of the investigative team). The open-ended semi-structured interview will last between 30 and 60 min and will be designed to collect data on the Age-Well participants' perceptions of English learning and meditation. The consistency between the participant's perception and the teacher's perception will then be assessed.

6.11. *Visit no.4 – Follow-up visit 29±6 months post-intervention*

29±6 months (35 months) after the end of the procedure, each participant will be seen for a long-term follow-up visit. This visit will include the same examinations as those performed in V1 and V3 except polysomnography and PET-Amyvid® examination.

The Cumulative Illness Rating Scale (CIRS) score will also be calculated, combining medical, biological, neuropsychological, and polysomnographic data collected at visits 1, 3, and 4.

Polysomnography is the reference method in this field, but it is extremely time-consuming and requires expertise both for the installation of electrodes and sensors and for the analysis of the tracings. During

the long-term follow-up of the participants, we will use a simpler and faster tool than polysomnography. The Dreem headband is a new EEG measurement tool that appears to be as accurate as polysomnography. It comes in the form of a headband with 5 dry electrodes (i.e. not requiring the application of a conductive paste) allowing the measurement of brain electrical activity, cardiac activity, respiratory rate and movements. It is easily placed on the participant's head, without any prior preparation. It takes less than three minutes to fit the headband to the participant's head and to move the hair away from the contact points of the electrodes. This headband will make it easier to collect sleep data, and will be proposed several consecutive nights at the participant's home in order to evaluate the impact of sleep variability, which is practically impossible with polysomnography.

In MRI2 session, the QSM sequence will not be performed because the signal-to-noise ratio does not allow to obtain usable scans. (Many QSM sequences in V1 and V3 are not valid.) It will be replaced by a 10-minute high-resolution DTI Locus sequence (HR-DTI-Locus), complementary to the HPC-T2 scan to understand the precise links between hippocampal substructures (HPC-T2), their connectivity and locus coeruleus substructures (HR-DTI-Locus) (Betts et al., 2019 Brain; Treit et al., 2018 Neuroimage).

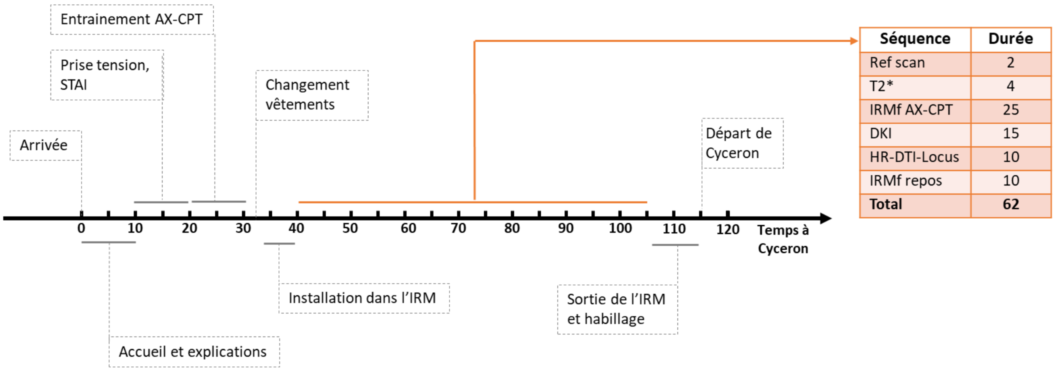

Figure 8: Course of the MRI2 examination in V4 (the time spent in the MRI is indicated in orange with a total duration at the Cyceron center of approximately 120 min).

Neuropsychological and behavioural assessment on V4

Each participant will be welcomed at the Cyceron center by their referent or an investigating psychologist to perform a set of cognitive tests. The battery of tests proposed will be identical to that proposed during the inclusion visit (see Table 5 above). A questionnaire on the practice of English or meditation following the intervention program will be proposed in order to evaluate the impact of the intervention in the long term on cognition, psycho-emotional factors, sleep and lifestyle. All the tests and questionnaires will be carried out during the post-intervention evaluation visit (V4) in 2 sessions of approximately 2 hours during which breaks will be scheduled .

Calculation of the CIRS score (Cumulative Illness Rating Scale): The CIRS provides an overall numerical measure of an individual's pathologies (Groot et al., 2003) and appears to be more subtle and

useful than the CCI (Charlson Comorbidity Index) for assessing the overall health status of participants. It is also more adapted to a population of older adults without serious pathologies. The CIRS will provide a more detailed assessment of the overall health status of the participants in the trial.
The CIRS score is calculated from medical (history, treatments), biological (Body Mass Index (BMI), Blood Pressure (BP), Blood Count (CBC), Exploration of Lipid Abnormality (EAL), Fasting Blood Glucose), neuropsychological (Mini-Mental State (MMS)), and the Apnea-hypopnea index (AHI) score from polysomnography, collected during visits 1, 3 and 4 collected in the eCRF.

6.12. *Significant health outcomes (information, follow-up, treatment)*

In case of detection of pathological abnormalities during the imaging or biological tests, the investigator will inform the participant and their regular physician. The results will be transmitted to the regular physician who will take charge of the participant's medical follow-up. These persons will benefit from a classical clinical follow-up by their regular physician(s).

6.13. *Termination of participation in the trial*

At the end of participation in the trial, participants will receive standard clinical follow-up by their regular physician.

6.14. *Premature termination of the trial*

In the event of premature discontinuation of the trial (withdrawal or withdrawal of consent), participants will receive standard clinical follow-up by their regular physician.

A participant is considered lost to follow-up when they stop the protocol follow-up without a reason known to the investigator, so that data collection cannot be conducted as planned. Participants lost to follow-up should be actively sought by the investigator.

A participant who wishes to withdraw their consent to participate in the research (as they is entitled to do at any time), is no longer followed within the framework of the protocol, but must receive the best possible care given their state of health and the state of knowledge at the time.

A withdrawal of consent is a decision, by an included participant to exercise their right to discontinue participation in a research study at any time during the course of follow-up, without incurring any prejudice as a result, and without having to provide a reason.

The investigator should identify the cause of the withdrawal of consent and assess whether it is possible to collect, for the participants included in the randomized clinical trial, the primary outcome variable at the time of withdrawal of consent.

Withdrawals of consent should be promptly notified to the sponsor. The reasons for and date of withdrawal of consent should be documented in the participant's case report and medical record.

No data will be collected after withdrawal of consent but previously collected data will be retained unless the participant objects in writing.

6.15. *Deviations from the protocol*

Deviations may concern all aspects of a research protocol: inclusion process, follow-up, measurement of judgment criteria, treatments. All deviations must be documented by the investigator and discussed at the Scientific Council.
Only dropouts lead to a termination of the follow-up. Even in the case of a deviation from the protocol, the participant's follow-up must be carried out until the end of the protocol.

6.15.1. Premature and permanent cessation of research treatment

A participant is considered to be in treatment discontinuation when they stop treatment before the protocol date. Participants in early discontinuation continue to be followed as planned in the protocol. In no case should the planned follow-up be modified.
The participant who discontinues treatment must receive the best possible care given their state of health and the state of knowledge at the time.

6.15.2. Lost to follow-up participant

A participant is considered lost to follow-up when they stop the protocol follow-up for no reason known to the investigator, so that data collection cannot be completed as planned.
Lost to follow-up participants should be actively sought by the investigator.

6.15.3. Incorrectly included participant

A participant is considered to be wrongly included when they were actually included in the research even though they did not meet all the eligibility criteria. Participants who are wrongly included must be discussed by the Scientific Council. They must continue to be followed as per the protocol until a decision has been made by the Scientific Council.

6.16. *Rules for the discontinuation of the trial*

6.16.1. Description of the rules for permanent or temporary cessation of part or all of the trial

Discontinuation of the trial by the investigator:

In the event of an adverse event judged to be severe by the investigator and which could jeopardize the health of the participants, the coordinating investigator may stop the trial in agreement with the sponsor.

If a hypersensitivity or anaphylactic reaction occurs, the radiopharmaceutical administration should be discontinued immediately.

Discontinuation of the trial by the sponsor:

The sponsor may stop the trial at any time for the following reasons:

- Failure of the investigator to include participants on schedule;
- Major violations of protocol;
- Incomplete or incorrect data;
- Occurrence of a new security event.

If the trial is stopped definitively, Inserm will inform all the investigators, the CPP and the ANSM in writing of the reasons for this definitive interruption.

6.16.2. Defining the end of trial on individuals

Once all the examinations have been completed, i.e. the V1 visit for expert meditators and the V1 to V4 visits for non-expert seniors, the trial will be completed for the participants (end of study for the participant).

The end of the trial will be defined as the end of the analyses, approximately 12 months after the last visit of the last included participant.

7. Description of the logistic organization of the trial

7.1. *General trial logistics*

Table 7 (below) lists the various examinations performed by the participants, specifying for each one the person in charge of the examination and the place where it was performed.

Group	Place	Supervisor

	Non-expert seniors			Expert meditators		
	Mediatiton group	Foreign language learning group	Passive control group			
Recruitement	x	x	x		Caen and region	Minerva and Mediapilote
				x	Europe	Expert-Associate
V0: Screening visit and medical interview	x	x	x	x	Cyceron center	Physician and investigator neurpsychologist
V1: Inclusion visit	x	x	x	x	-	-
MRI1 session	x	x	x	x	Cyceron center	Referent and radiology manipulator
MRI2 session	x	x	x	x	Cyceron center	Referent and radiology manipulator
MRI3 session				x	Cyceron center	Referent and radiology manipulator
PET-Amyvid®	x	x	x	x	Cyceron center	Referent and radiology manipulator
PET-^{18}F-FDG	x	x	x	x	Cyceron center	Referent and radiology manipulator
Neuropsychological and behavioural assessment on V1	x	x	x	x	Cyceron center + At home	Referent or investigator neuropsychologist
Blood sample on V1	x	x	x	x	CRC – CHU of Caen	Referent and CRC
Assessment of wakefulness and sleep	x	x	x	x	PFRS and at home	Referent
Randomization	x	x	x			EUCLID
18-month intervention	x	x			-	-
Weekly meditation group session	x				PFRS – Caen	Meditation teacher
Daily meditation exercise	x				At home	Participant alone
Day/half-day of intensive meditation practice	x				PFRS – Caen	Meditation teacher
Weekly English group learning session					Carré International – UNICAEN	English teacher
Daily English exercise					At home	Participant alone
Day/half-day of intensive English practice					Jersey	English teacher
Agenda/questionnaire for the intervention's follow-up	x	x			At home	Participant alone
V2: Intermediate evaluation visit at 9 months	x	x	x		-	-
Neuropsychological and behavioural assessment on V2 (1 session)	x	x	x		Cyceron center	Referent or investigator neuropsychologist
V3: End of intervention visit	x	x	x		Same as V1	Same as V1

Medical interview<br>Same examinations as V1						
Qualitive interview about intervention's experience	x	x	-	-	Cycéron center	Independent experimenter (blind knowledge)
V4:29 months post-intervention visit	X	X	X		Same as V1 and V3 except for polysomnography and PET-Amyvid	Same as V1 and V3 except for polysomnography and PET-Amyvid

Tableau 7: Global logistics of the Age-Well trial

All data will be collected directly on site and transmitted to the persons responsible for their analysis and processing (see sections 9.3 to 9.6).

7.2. *Experimental products*

^{18}F-Florbetapir is a radiopharmaceutical drug, it is a speciality (Amyvid®) that has obtained a marketing authorization. We have the summary of product characteristics for this product.

The Amyvid® is indicated for PET imaging to measure the density of senile plaques in the brains of cognitively impaired adult patients being evaluated for Alzheimer's disease and other causes of cognitive impairment. As part of this biomedical research protocol, Amyvid® will be administered to healthy volunteers. This use outside of the AD indication requires that Amyvid® is considered as an investigational drug.

^{18}F-FDG is a radiopharmaceutical drug, it is a speciality (Glucotep®) that has obtained a marketing authorization. We have the summary of the product characteristics for this product.

7.2.1. Name and description of the product / medical device

Amyvid® (florbetapir (^{18}F)) is a fluorine-18 labelled radiopharmaceutical drug in the form of a clear, colorless aqueous solution. It is a ready-to-use speciality packaged in 10 or 15 mL multi-dose glass vials containing 800 to 12,000 MBq at the date and time of calibration (Toc).

Amyvid® Specialty contains as excipient ethanol (75 mg/mL), sodium chloride (37 mg maximum per vial), sodium ascorbate, and water for injection.

Each vial is supplied enclosed in a leaded container with an appropriate thickness to limit exposure to γ radiation of 511 keV .

The summary of product characteristics is provided in annex 4a .

Glucotep® is a fluorine-18 labelled radiopharmaceutical drug in the form of a clear, colorless, aqueous solution. It is a ready-to-use speciality packaged in colourless or brown type I glass vials that comply with the European Pharmacopoeia. They are closed by an elastomeric stopper (chlorobutyl) sealed by an aluminum capsule. Each vial contains between 0.1 and 10 mL of solution corresponding to an activity between 15 and 1500 MBq at calibration date and time (Toc).

Each milliliter of Glucotep® solution contains 5.2 mg of sodium as chloride and citrate .

Each vial is delivered enclosed in a leaded container with an appropriate thickness to limit exposure to γ radiation of 511 keV .

The summary of product characteristics is provided in annex 4b.

7.2.2. Terms of use

7.2.2.1. Dose and method of administration of the product / Method of operation and use of the device

The dose of Amyvid® is 370 MBq for a 70 kg patient as described in the SPC. This dose will be optimized for the AGE-WELL trial. Indeed, in order to limit the exposure of subjects to ionizing radiation, the dose used will be 4 MBq/kg. This adaptation is made possible thanks to the sensitivity of the hybrid Positron Emission Tomography (PET) and Whole Body CT (WBCT) camera of GIP CYCERON (Discovery RX VCT 64 General Electric Healthcare).

The dose of Glucotep® is between 100 and 400 MBq depending on the patient's body weight, the type of camera used and the acquisition mode. For the evaluation of cerebral glucose consumption, a single dose of 200 MBq, independent of weight, is usually used.

The administration of these radiopharmaceuticals must be performed intravenously with prior calibration using an activimeter. The injection is performed as a bolus via a venous catheter followed by rinsing with a 9 mg/mL (0.9%) sodium chloride injection solution to ensure complete administration of the dose. A differential measurement of the full and empty syringe also allows for an accurate estimation of the dose injected to the subject.

In order to limit the phenomenon of adsorption of florbetapir (^{18}F) on the venous catheter, its length must be limited (maximum 4 cm). Before administration of the radiopharmaceutical drug, it will be necessary to ensure the correct positioning of the catheter in order to avoid extravasations which lead to significant local irradiation and imaging artifacts.

7.2.2.2. Randomization

PET scans with Amyvid® and Glucotep® radiopharmaceuticals will be performed regardless of the randomization group of participants in the trial .

7.2.2.3. Compliance control

NA

7.2.3. Treatments authorized during the trial

NA

7.2.4. Unauthorized treatments during the trial

Amyvid® and Glucotep® do not appear to have any particular interaction with other drug classes. However, some treatments are not allowed in the trial (see previous paragraph (1.1.3.)).

7.2.5. Logistic circuit

7.2.5.1. Description of product presentation

^{18}F-Florbetapir:

Amyvid® is packaged in multi-dose vials of 10 or 15 mL. The vial is made of type I colorless borosilicate glass. It is closed with a chlorobutyl rubber stopper protected by a FluroTEC protective film and sealed with an aluminum cap.

The bottle has a primary label with the following information:
1 – name of the drug: Amyvid with volume activity (800 MBq/mL), route of administration and the international nonproprietary name (INN) florbetapir (^{18}F);
2 – active substance composition: specifies the active substance activity by volume at Toc;
3 – list of excipients: ethanol, sodium ascorbate, sodium chloride and water for injection;
4 – pharmaceutical form and content: injectable solution, volume and activity at Toc;
5 – mode and route of administration;
6 – special warning that the drug should be kept out of the reach and sight of children;
7 – other special warning: radioactive clover and coordinates of the production site;
8 – the expiration date;
9 – special storage precautions;
10 – special precautions for disposal of unused drugs or waste from these drugs if applicable;
11 – name and address of the marketing authorization holder: Eli Lilly Nederland B.V., Papendorpseweg 83, 3528 BJ Utrecht, Netherlands;
12 – marketing authorization number: EU/1/12/805/001 (10 mL) and EU/1/12/805/002 (15 mL);
13 – the lot number and the bottle number;
14 – conditions of prescription and delivery: medicine subject to medical prescription;
15 – indications for use.

^{18}F-FDG:

Glucotep® is packaged in multi-dose vials of 15 mL.
The bottle has a primary label with the following information:
1 – drug name: Glucotep® with volume activity (150 MBq/mL at Toc), route of administration and the international non-proprietary name (INN) fludeoxyglucose-(^{18}F);
2 – active substance composition: specifies the active substance activity by volume at Toc;
3 – pharmaceutical form and content: injectable solution, volume and activity at Toc;
4 – mode and route of administration;
5 – special warning that the drug should be kept out of the reach and sight of children;
6 – other special warning: radioactive clover and coordinates of the production site;
7 – the expiration date;
8 – name of the laboratory: Cyclopharma and address of the production site;
9 – marketing authorization number: N° 575 243 – 7;
10 – the lot number and the bottle number;
11 – conditions of prescription and delivery: medicine subject to medical prescription.

7.2.5.2. Procurement

For Amyvid®, orders are placed with the pharmaceutical laboratory Lilly with a 72-hour delay between the order and the desired delivery date. The order is placed by computer entry on a dedicated order form and sent by fax and e-mail.
For Glucotep®, orders are placed with the pharmaceutical laboratory Cyclopharma with a 24-hour delay between the order and the desired delivery date. The order is entered on a computerized order form and sent by fax or e-mail.

The main elements contained on the order forms are the pharmaceutical company's contact information, the name of the principal investigating physician, the delivery address and the name of the radiopharmacist in charge of the order. They also include the desired delivery date and time, the patient inclusion codes for the Amyvid® specialty, the time and calibration of the MRP doses and the signature of the radiopharmacist. An acknowledgement of receipt is sent by the supplier 24 hours after the order and an order confirmation 24 hours before delivery. The reception of MRP is done in accordance with the loading/unloading procedures of radioactive substances established between the CYCERON center and the pharmaceutical laboratory. A return of the transport packages of MRP is organized for each reception of dose. This operation is carried out in accordance with the regulations in force and the procedures for the return of drums from suppliers.

7.2.5.3. Storage

The storage of radiopharmaceuticals is located within the GIP CYCERON. A primary area is dedicated to the reception of sources and allows a temporary storage before transfer to the nuclear medicine sector. The nuclear medicine department has a laboratory dedicated to handling which is the secondary storage area for sources before preparation and dispensing. Receipt of the sources involves checking that their transport complies with ADR regulations (verification of the transport index, measurement of dose rates, compliance with the signage and conformity of the activities delivered). A pharmaceutical control is essential at this stage, it will concern the adequacy between the delivered activities and the ordered activities (available volumetric activity, MRP volumes), the integrity of the packaging(s) and the conformity of the primary and secondary labeling. The presence of the quality control certificate is essential before any dispensing and administration to patients.

7.2.5.4. Dispensing

Before any dispensing of radiopharmaceutical drug marked with fluorine 18, a control of the activimeter is realized (daily control). In order to proceed with the dispensing, an estimation of the volume activity to be shipped is made in order to determine the volumes to be taken for the preparation of the syringes. For the dispensing operations, the vial is transferred to a shielded cell under vacuum inside which the preparation of the dose takes place (syringing). During this dispensing, a label is generated to ensure the traceability of the pharmaceutical act. This label includes the following information: the name of the

MRP, the dose syringed, the lot/vial number, the expiration date, the patient's name, the inclusion code and weight, the name of the protocol, the EUDRACT number of the trial and the name of the principal investigator. The following legal information is also included:
« For biomedical research only. Sterile injectable solution for administration
by strict intravenous route in a single injection. Use under strict medical supervision
(article R.5121 CSP). Store at room temperature in a sealed container. »

Traceability: At all stages of the drug circuit, there are physical and computerized traceability elements such as the order summary file, order files, the source reception and control register, the Institut de Radioprotection et de Sureté Nucléaire (IRSN) register of sources held and the examination progress sheets. A medical prescription and informed consent form are required for each examination. A prescription number is assigned to each order.

7.2.5.5. Return of treatment units / medical devices

NA

7.2.5.6. Destruction

After use, the unused radiopharmaceutical residues are kept in radioactive decay within the shielded distribution cell. After 10 physical periods (about 20 hours), the vial is disposed of according to the procedures in force within the GIP CYCERON.

7.3. *Collection of biological samples of human origin*

All procedures related to biological sample collections are included in the laboratory manual.

7.3.1. Nature of the samples

Venous blood samples.

7.3.2. Sampling conditions

An appointment date and time will be set for each participant at the Clinical Research Center (CRC) of the University Hospital of Caen, France.

Blood samples will be taken by frank venipuncture at the elbow, using a winged sampling unit. They will be taken at the CRC of the CHU of CAEN (unit 13-30). The sampling methods will be in accordance with the pre-analytical requirements of the prescribed analyses as well as with the legislation in force (Guide des Bonnes Exécution des Analyses de biologie médicale) under the responsibility of the sampling establishment, the CHU of Caen.

All medical tests to be performed will be recorded on the prescription written, dated and signed by the investigating physician.

Participants will arrive on an empty stomach (between 8:00 and 10:00 a.m.) and will be greeted by their referent. Participants will have been previously informed not to consume tomatoes, citrus fruits,

dried fruits, avocados, pineapples, plums, chocolate and bananas one day before the blood test. Once the blood test is done, they will be invited to have a snack if they need to continue with another test.

7.3.3. Coding and labelling procedures

The schedule of the participants' appointments at the CRC will be transmitted in advance by the referent so that the CRC can prepare the sampling "kits" with the appropriate number of tubes. A computer file will be created for each participant on the day of the sampling, in the computer system of the CHU, and a file number will be assigned to them as well as a bar code.

The blood sample tubes will be identified at the CRC at the time of collection by a label with the surname, first name, date and place of birth of the participant, the date of the day as well as the number and bar code of the patient file for the analyses that will be done in the CHU laboratories and will be pseudo-anonymized via the participant code of the trial thanks to the documents provided by the referent for all the tubes destined to be analyzed by the external laboratories.

7.3.4. Sample processing procedures

The CRC will be responsible for the reception of the participants, the sampling, the preparation of the samples, the good execution of the various pre-analytical stages, as well as the provision of the samples towards the dedicated structures, according to the instructions and procedures communicated beforehand, and in respect of the legislation in force and the Guide des Bonnes Exécutions des Analyses de Biologie Médicale (Guide to the Good Execution of Medical Biology Analyses).

For each non-expert senior participant, 18 tubes (70 mL blood volume) will be collected at visit V1, 16 tubes (64 mL blood volume) at V3, 16 tubes (70 mL blood volume) at visit 4.

The oxidative stress marker initially selected, superoxide dismutase (SOD), was not measurable on our frozen samples stored at the CRB, so we opted for another marker that could be measured in a small volume of previously stored plasma sample, peroxyredoxin, in order to have a longitudinal measurement of oxidative stress.

The collaboration with Life Length (telomere assay and telomerase activity) as well as U976 Saint Louis Hospital (lymphocyte immunosenescence) who were also extracting PBMC (Peripheral Blood Mononuclear Cells) has ended. This led us to contract with the CRB of the CHU of Caen, already involved in the management and conservation of our biological samples, the extraction of PBMC. The modification of the blood volume in V4 has therefore been updated.

For each meditating expert participant, 17 tubes will be collected (i.e. blood volume of 64 mL) during the V1 visit.

Blood biomarkers	Number and type of tubes collected	Samples' destination	Specific conditions					
				Experts (V1)	Non-expert seniors			
					Inclusion (V1)		18 months (V3)	35 months post-intervention (V4)
Cholesterol<br>Triglycerides<br>HDL<br>LDL<br>Urea<br>Creatinine<br>GGT<br>TGP<br>TGO	1 Dry<br>(Blood vol. 4 mL)	CHU Caen Biochemistry	on empty stomach	x<br>x<br>x<br>x<br>x<br>x<br>x<br>x<br>X<br>x	x<br>x<br>x<br>x<br>x<br>x<br>x<br>x<br>x		x<br>x<br>x<br>x<br>X<br>X<br>X<br>X<br>x	x<br>x<br>x<br>x<br>X<br>X<br>X<br>X<br>x
Glycemia	1 Fluorinated<br>(Blood vol. 2 mL)	CHU Caen Biochemistry	on empty stomach	x	x		x	x
Blood Cell Count	1 EDTA<br>(Blood vol. 2 mL)	CHU Caen Hematology		x	x			
APOE4	1 EDTA<br>(Blood vol. 4mL)	CHU Caen Biochemistry		x	x			
cDNA/mRNA<br>GWAS	1 EDTA<br>(Blood vol. 4mL)	CRB		x<br>x	x<br>x		x<br>x	x<br>x
Biobank	1EDTA<br>(Blood vol. 4mL)	CRB / Cyceron U1237		x<br>x	x<br>x		x<br>x	x<br>x
BNP<br>Total Insulin	1 EDTA<br>(Blood vol. 4 ml)	CRB Multipurpose medical analysis laboratory		x	x		x	x
Estradiol<br>SDHEA<br>Cortisol<br>CRP ultra-sensitive<br>SHBG<br>Bioavailable testosterone<br>TSH<br>IGF-1	3 SECS<br>(Blood vol 12 mL)	Multipurpose medical analysis laboratory<br>CRB	In the morning – between 8:00 AM and 10:00 AM	x<br>x<br>x<br>x<br>x<br>x<br>x<br>x	x<br>x<br>x<br>x<br>x<br>x<br>x<br>x		x<br>x<br>x<br>x<br>x<br>x<br>x	x<br>x<br>x<br>x<br>x<br>x<br>x
Serotonin<br>Biobank	1 HEPARIN<br>(Blood vol 4 mL)	Multipurpose medical analysis laboratory<br>CRB	Food restriction 1 day before (tomatoes, citrus fruits, dried fruits, avocados, pineapples, plums,	x<br>x	x<br>x		x<br>x	x<br>x

			chocolate and bananas)					
tPA<br>PAI-1<br>Fibrinogen	1 CITRATE<br>(Blood vol 4 mL)	CRB / Cyceron U1237		x<br>x	x<br>x		x<br>x	x<br>x
Cytokines<br>BDNF<br>Tau & PhosphoTau<br>Aβ-40 & 42<br>Neurofilament Light<br>Peroxyredoxin<br>GFAP	2 EDTA<br>(Blood vol 8 mL)	CRB / Cyceron U1237		x<br>x<br>x<br>x<br>x	x<br>x<br>x<br>x<br>x		x<br>x<br>x<br>x<br>x	x<br>x<br>x<br>x<br>x
Lymphocyte immunophenotyping	3 heparins<br>(Blood vol 12 mL)	U976 Hôpital saint Louis	Transfer on the day of collection	x	x		x	
Telomeres & Telomerase	1 EDTA<br>(Blood vol 6 mL)	Life Length	Transfer on the day of collection		x		x	
Extraction of PBMC for storage and subsequent epigenetic analysis	4 EDTA<br>(Blood vol 24 mL)	CRB						x
	18 tubes<br>(Blood vol 68 mL)			17 tubes (Blood vol 64 ml)	18 tubes (Blood vol 70 mL)		16 tubes (Blood vol 64 mL)	16 tubes (Blood vol 70 mL)

Table 8 - Details of blood-based biological markers, sample collection and analysis conditions.

For a description of the blood sample flow, techniques and pretreatments, see the laboratory manual.

7.3.5. Transport conditions

The transport conditions are organized and implemented in collaboration with the other collaborating laboratories which will take charge of the transport of the samples from the CRC or the CRB of the University Hospital of Caen.

In a suitable container, insulated at -20°C, the samples destined for the multipurpose medical analysis laboratory (Cerba laboratory) will be directly transported by a qualified carrier who will pick them up at the CRC or the CRB in V4 according to the terms specified by the investigator. The transport conditions will be under the responsibility of the multipurpose medical analysis laboratory.

The analyses that will be carried out on the samples kept at the CRB and destined for Inserm unit U1237, will be done from samples that an authorized investigator from the Inserm U1237 team will come directly to pick up, after having made a request by fax to the CRB to make the samples available. These samples will be transported in a triple-walled transport device, containing dry ice and directly transported, in less than one hour, to the unit's laboratory (the transport conditions will be in accordance with the pre-analytical requirements of the analyses to be carried out). The samples will be analyzed on site at the Unit's laboratory.

Three 5 ml heparinized tubes taken from each participant will be given by the CRC sampler to the transporter sent by the Inserm U976 unit of the Saint-Louis Hospital. These samples will be sent directly to them the same day. They will be given information about the collection (date, time of collection) beforehand. They are intended for the study of immunophenotyping of standard circulating lymphocytes and focused on lymphocyte aging with analysis of cells of the innate response and cells of the adaptive response.

In visit 4, an extraction of " Peripheral Blood Mononuclear Cells " (PBMC) (previously performed by the Life length laboratory on tubes intended for telomeres and telomerases assays) will be performed by the CRB. Then these PBMC will be stored at the CRB.

7.3.6. Storage conditions

The storage conditions of the samples collected in our trial, entrusted to the CRC and then kept at the Biological Resource Center (CRB) will be:

- in accordance with the pre-analytical requirements of the prescribed analyses,
- comply with the legislation in force concerning human tissue biobanks/collections, respecting the HAS recommendations for cryopreservation,
- comply with the legislation in force (Guide des Bonnes Exécution des Analyses de biologie médicale),
- under the responsibility of the sampling institution, the University Hospital of Caen.

Samples destined for the multipurpose medical analysis laboratory will be kept refrigerated between 4 and 8°C for 24 hours or at - 80°C (if more than 24 hours) at the CRC or the CRB in V4 until the carrier passes through.

7.3.7. Description of secure storage conditions

The samples stored at the CRB of the Caen University Hospital will be secured in accordance with the legislation in force concerning biobanks/human tissue collections. The methods of securing the storage of samples will be in accordance with the legislation in force (Guide des Bonnes Exécution des Analyses de biologie médicale) and under the responsibility of the CRB of the Caen University Hospital.

7.3.8. Sample management procedures

Sample management will be in accordance with the CRB procedures specific to each project in progress.

7.3.9. Computerized management of sample data

The computerized management of the data relating to the samples will be carried out according to the CRB procedures specific to each project in progress.

Once the results of the different analyses have been received at the CRC, the data will be sent as quickly as possible, pseudo-anonymized with the trial participant code, to a secure e-mail box for the Cyceron center's investigating physicians to check the hepatic and renal parameters, calculate the glomerular filtration rate and the blood sugar, and then to enter them into the eCRF. Afterwards, the validated results in paper version will be sent by mail to the investigating physician.

All tubes stored at the CRB will have been pseudo-anonymized with the participant code of the trial at the CRC before their transfer. A correspondence table will be available in the CRB computer files in case of a possible need to lift anonymity.

7.3.10. Conditions of traceability

The traceability of samples destined for the biochemistry and hematology laboratories of the University Hospital, the multi-purpose medical analysis laboratory, the Saint Louis laboratory and the Life Length laboratory is the responsibility of the CRC and the CRB in V4 of the University Hospital.

The request to send a sample from the CRB to a collaborating laboratory will be made by e-mail or fax. The laboratory will acknowledge receipt of the samples by e-mail or fax to the CRB.

The traceability of the samples will be in accordance with the CRB procedures specific to each project in progress.

7.3.11. Description of the collection's quality system

The CRB procedures specific to our project are attached to this document.

8. Vigilance of the trial

8.1. *Definitions*

The Inserm Clinical Research Unit is responsible for the vigilance of this clinical trial as well as the reporting of adverse events to the competent authorities throughout this trial.

Investigators are responsible for collecting AEs and SAEs occurring in participants (even if the risks generated by their participation in the trial are limited) and for reporting them to the sponsor.

8.2. *Adverse Event (AE)*

Any adverse event in a patient or biomedical research participant, whether or not related to the radiopharmaceuticals Amyvid® and Glucotep® or to the trial.

8.2.1. Adverse event

Any harmful and unwanted reaction that may be related to Amyvid® and Glucotep® radiopharmaceuticals or to the trial (e.g., procedures, methods, or acts performed for the purpose of the trial).

8.2.2. Serious adverse event (SAE)

Any adverse event/effect meets the definition of "serious" if it:

- leads to death;
- endangers the life of the participant (corresponds to an event in which the participiant is at risk of death at the time of the event; it does not correspond to an event that hypothetically could have caused the death if it had been more severe);
- requires hospitalization or prolongation of hospitalization;
- causes a significant or lasting disability or incapacity;
- results in a congenital anomaly or malformation;
- is a Grade 4 clinical or laboratory adverse event;
- is a “significant medical event“ (an event that is considered by the investigator to be medically important and may endanger the participant or require intervention, medical or surgical, to prevent any of the above characteristics/consequences. Examples: intensive treatment in a hospital emergency room or at the participant’s home for allergic bronchospasm, seizure or bleeding disorders).

8.2.3. Serious Unexpected Adverse Effect (SUAE)

Any adverse reaction whose nature, severity or course is not consistent with the Safety Reference Information (SRI) for Amyvid®, Glucotep® and the procedures and methods used in the trial.

8.2.4. New safety event

A new safety event is defined as any new safety data that may significantly alter the assessment of the benefit/risk ratio of the trial, that may affect the safety of participants, or that may be sufficient to consider changes in the interventions tested, the conduct of the research, or the research materials.

8.3. *Investigator's responsibilities*

8.3.1. Notification of AEs to the sponsor

The investigator should report in the electronic case report form all biological and clinical AEs, except those detected before the V1 visit.

If a clinical or biological AE is present at inclusion, only its worsening should be reported.

8.3.2. Notification of the SAEs to the sponsor

8.3.3. *What should be declared?*

The investigator should report to the sponsor all serious adverse events except those identified in the protocol or investigator's brochure as not requiring immediate reporting.

AEs not requiring immediate notification to the sponsor:

- Some hospitalizations:
  - Admission to a day hospital; the trial's participant is admitted for less than 24 hours for a non-serious medical reason (see severity criteria above);
  - Hospitalization for medical or surgical treatment scheduled before the trial;
  - Hospitalization for administrative or social reasons;
  - Hospitalization predefined by the protocol.
- Pre-existing condition or condition detected prior to the V1 visit that is not worsening.

The investigator also sends to the sponsor all relevant documents related to the SAE (e.g. copies of laboratory results, reports of examinations or hospitalizations providing information on the SAE), without omitting to make the documents anonymous and to enter the participant's identification number in the trial.

The investigator should follow participants who have experienced an AE until resolution of the adverse event, stabilization at a level acceptable to the investigator, or return to baseline, even if the participant has exited the trial.

8.3.3.1. When to report ?

Reportable SAEs and any relevant documents related to these SAEs must be sent by the investigator to the sponsor, without delay from the day they becomes aware of them.

The investigator ensures that relevant follow-up information is provided to the sponsor within a further 7 days.

Any serious adverse event must be reported, if it occurs for a research participant, for the entire duration of the research, i.e. from the date of signing the consent form and until the end of the participant's follow-up.

Serious adverse events experienced by a participant after the end of follow-up (e.g., serious events that may occur long after exposure to the drug, such as cancers or birth defects) should be reported to the sponsor if the investigator is aware of them. The investigator is not required to actively monitor participants for adverse events after the trial has ended, unless otherwise specified in the protocol.

8.3.3.2. How to report ?

The initial notification is reported in writing and in detail using the "Initial Serious Adverse Event Report" form (located in the SAE section of the eCRF).
The initial notification must contain, at a minimum, all of the following elements: an identifiable participant code, an identifiable notifier, the radiotracer (Amyvid® or Glucotep®), the type of procedure performed, an adverse event.

The initial notification is followed by additional detailed written reports using the "Additional Serious Adverse Event Report" form (located in the eCRF section) to monitor the progress of the case or to complete the information.

When the form is completed, dated and signed by the investigator, an email is immediately and automatically sent to the Inserm « Mission Réglementation et Qualité et Recherche Clinique (RQRC) (Regulation and Quality and Clinical Research Mission).

If the eCRF is not available, the investigator completes and faxes the paper report form to:

Institut Thématique Santé Publique - Recherche Clinique et Thérapeutique (ITSP – RCT)
Mission Réglementation et Qualité et recherche clinique (RQRC)

Fax: +33 1 44 23 67 10
E-mail: rqrc.siege@inserm.fr

All SAEs reported through the paper circuit must be re-entered in the "Serious Adverse Event Report" section of the eCRF.

The investigator sends all relevant pseudo-anonymized documents related to the SAE (e.g. laboratory results, test reports, hospitalization reports, etc.) by fax to:

Institut Thématique Santé Publique - Recherche Clinique et Thérapeutique (ITSP – RCT)
Mission Réglementation et Qualité et recherche clinique (RQRC)
Fax: +33 1 44 23 67 10
E-mail: rqrc.siege@inserm.fr

8.3.4. Evaluation of AEs

8.3.4.1. Adverse event

The investigator should document the adverse event to the best of their ability and, if possible, provide a medical diagnosis.
The date of occurrence of the adverse event must be before (or the same as) the date of occurrence of the severity.
In the event of a medical or surgical procedure (e.g., surgery, endoscopy, dental extraction, transfusion, etc.), the investigator should note the event that led to such a procedure.

8.3.4.2. Intensity

The intensity of all AEs (serious and non-serious) should be assessed by the investigator using the Common Terminology Criteria for Adverse Events (CTCAE v4.0) grading scale and reported by the investigator in the case report form.

8.3.4.3. Severity

The decision to classify an event as serious or non-serious is generally made by the reporting investigator (see severity criteria).
Death of a research participant from a known cause should be reported as the progression of an adverse event and not as an adverse event. If the cause of death is not known, the death should be reported as "death of unknown cause”.

8.3.4.4. Causal link

The investigator should assess the causal relationship between the occurrence of the adverse event and the administration of the radiotracers Amyvid® or Glucotep®, the type of intervention, the research (i.e., with the acts and procedures implemented for the trial) and any associated treatments taken by the research participant.

8.3.4.5. Expected or unexpected character

The assessment of expected/unexpected is generally performed by the sponsor.
The expected/unexpected nature of a serious adverse event is assessed in light of the Safety Reference Information (SRI) mentioned in the Summary of Product Characteristics (SPC) of Amyvid®, in the SPC of Glucotep®, or described in the protocol, relating in particular to the acts and methods practiced during the research.

8.3.4.6. Evolution of the adverse event

The evolution of the event at the time of notification must be indicated on the initial SAE report form.
Any change in the evolution of the event (resolution, return to the previous state, aggravation, etc.) must be reported on a supplementary SAE declaration form.

8.3.5. Reporting of incidents or accidents in the field of radiation protection

Incidents or accidents likely to affect the health of individuals through exposure to ionising radiation are referred to as "significant events". The investigator responsible for the nuclear activity is required to declare these events without delay to the Autorité de Sureté Nucléaire and to inform the sponsor.
If the incident corresponds to the contamination of a third party by the investigational product, the investigator must report it to the sponsor as an adverse reaction.

8.3.6. Potentiel risks of the intervention and trial, and what to do in case of an adverse event

The expected risks in this trial are described in chapter 14.1.

In the event of an adverse event, the course of action is the usual medical management .

In order to limit the appearance of undesirable effects in the participants during the meditation training, a preliminary interview will be organized with one of the meditation teachers. If these effects appear, a follow-up will be set up with a psychotherapist. Finally, if these side effects become too severe, a termination of participation in the meditation intervention will be proposed.

8.4. *Responsibilities of the sponsor*

8.4.1. Recording and evaluation of SAEs

The sponsor maintains detailed records of all serious adverse events reported to it by the investigator(s).

The sponsor assesses the causal relationship between the occurrence of the serious adverse event and the research (i.e. with the trial procedures) and any associated treatments taken by the research participant (in case of drug interactions with radiopharmaceuticals).

In the absence of information on causality from the investigator, the sponsor should consult with the investigator and encourage the investigator to provide an opinion on causality.

The causal link established by the investigator should not be minimized by the sponsor. Where the sponsor's and investigator's assessments of causality differ, both shall be reported in the case report to the relevant national competent authority and ethics committee.

All serious adverse events for which the investigator or sponsor considers a causal relationship reasonably possible are considered serious adverse events.

The evaluation of the expected or unexpected nature of the serious adverse reaction is generally carried out by the sponsor, in light of the Safety Reference Information described in the protocol, relating in particular to the acts and methods practised during the research.
If information on the expected or unexpected character has been made available by the reporting investigator, it should be taken into account by the sponsor.

8.4.2. Declaration of safety data to the competent national authorities and the ethics committee

8.4.2.1. Reporting of Serious and Unexpected Adverse Events (SUAEs)

All SUAEs must be notified by the sponsor to the ANSM.

The deadlines for the notification of SUAEs are as follows:
- The sponsor must report any life-threatening or fatal SUAEs as soon as possible and within a maximum of 7 calendar days from the day on which they become known. Additional relevant information must be sought and transmitted within a further 8 days.
- Other SUAEs should be reported by the sponsor as soon as possible and within a maximum of 15 calendar days from the date of knowledge.

If the sponsor receives new relevant additional information on a previously reported SUAE, this new information will be reported within 15 days, starting from the day the sponsor becomes aware of it.

8.4.2.2. Reporting of safety developments

When there is a development in the research that may affect the safety of research participants, the sponsor and investigator shall implement urgent safety measures to protect participants from immediate harm.
The sponsor must declare to the ANSM and to the CPP concerned, any new event and the measures taken, if any, without delay and at the latest within 15 calendar days from the day on which it becomes aware of them.

8.4.2.3. Submission of the annual safety report

The sponsor must prepare an annual safety report once a year, for the duration of the research. This report will be written by the sponsor, with the help of the Inserm project leader, and validated by the coordinating investigator of the research.
It contains:

- A list of all expected and unexpected serious adverse events that occurred in the relevant research during the reporting period;
- Summary table of all serious adverse events;
- The list of deaths;
- A concise and critical analysis of the safety of research participants.

This report is sent by the sponsor to the ANSM and to the CPP within 60 days following the anniversary date of the first authorization of the trial.

9. TRIAL MONITORING

9.1. *Scientific Council*

The Scientific Council is responsible for ensuring that the protocol runs smoothly, that it is monitored and that Good Clinical Practices are followed. It is composed of members of the team directly involved in the clinical research: the Principal Investigator, the Scientific Manager, a representative of the sponsor (CRU), a representative of EUCLID (methodology and statistics), a representative of ECRIN, a representative of WP5 "Cognition and well-being", another one of WP6 "Biomarkers" and external experts. This committee will meet at least twice a year in order to follow the good progress of this trial.

Moreover, the governance of this project as well as the validation of strategic choices are the responsibility of the coordinator and the WP7 "management and coordination", the general assembly of

the European consortium "Medit-Ageing" and its executive committee (ExCom). All the rules of governance and the description of the decision-making bodies can be found in the document entitled "Medit-Ageing Management Guide V1- March 2016"

9.2. *Independant Supervisory Committee*

The Independent Supervisory Committee (ISC) is established by the sponsor, meets at the request of the sponsor, and is independent of the sponsor.

The mission of the ISC is to:

1. ensure the safety of participants throughout the trial,
2. maintain the scientific and ethical integrity of the trial.

The ISC is an advisory committee that provides advice and recommendations to the sponsor on the conduct of the trial.

The ISC may assist the trial sponsor and investigators in making decisions during the course of the trial for which independent judgment is desirable. In particular, the ISC may:

- Recommend additional analyses to interpret the results of an intermiadary review of the data;
- recommend minor or major protocol amendments that become necessary due to trial enrolment or follow-up or to reflect new scientific information;
- Recommend changes to statistical analysis criteria;
- recommend premature termination of the trial if, in its view, there is evidence (publications) or new events (SAEs), that would attest that the safety of participants is no longer assured.

The ISC is composed of the following independent members:

- Dr Agnès Caille – Methodologist, Public Health Physician – CHRU Bretonneau, Tours, France;
- Pr F Semah - PU-PH Nuclear Medicine – Clinique de Médecine Nucléaire et d'Imagerie Fonctionnelle, Hôpital Salengro CHU Lille, France;
- Pr P. Maquet – Professor of Neuropsychiatry – University of Liège, Belgium.

10. Data collection and processing

The data management plan will be the subject of a complete and specific document separate from the protocol. The main elements are included in this chapter.

10.1. *Description of the collected data*

The collection of information is done on an observation notebook.

10.1.1.1. Data collected at the screening visit (V0)

During this visit, we will collect various demographic information (age, gender, level of education, occupation) as well as general health parameters such as medical history, medication treatments or cardiovascular risk factors (blood pressure, weight, height, abdominal and hip circumference).

10.1.2. Data collected during the inclusion visit (V1)

10.1.2.1.1. MRI

Anatomical MRI:

Six types of anatomical images of different weights will be acquired from all participants during the first MRI session, all in electronic format (images):

- A high spatial resolution T1 acquisition
- A targeted acquisition on the hippocampus
- A T2 acquisition
- A FLAIR acquisition
- A DKI acquisition
- A QSM acquisiton
- An ASL acquisition (for participants in the 3rd wave of recruitment)

Functional MRI (resting and activation):

Dynamic functional acquisitions will be acquired both during activation fMRI sessions (AX-CPT and rest-SoVT) and during resting fMRI sessions. For each participant, as many functional acquisitions as there are fMRI sequences will be collected in electronic format, namely:

- *For non-expert seniors:* a functional acquisition of activation during the rest-SoVT task, a functional acquisition of activation during the AX-CPT task and a functional acquisition at rest.
- *For the expert meditators:* three functional acquisitions of activation during the rest-SoVT task (one for each meditation condition), one functional acquisition of activation during the AX-CPT task and three functional acquisitions at rest SoVT (one for each meditation condition).

These acquisitions will allow the study of brain function during the different tasks (attention and emotions) as well as functional connectivity. A brain volume of each participant will be acquired approximately every 2 seconds during all the fMRI sessions, thus allowing to measure the evolution of the cerebral neuronal activity in the same voxel over time. Indeed, the measurement of cerebral neuronal activity in fMRI is done through the BOLD signal which corresponds to an increase in the local blood concentration of deoxyhemoglobin (Logotheis et al., 2004). These concentration changes locally perturb the magnetic field and these perturbations are collected through the T2* weighted Echo Planar Imaging sequence every 2 seconds or so.

These functional acquisitions will be accompanied by a T2* acquisition (one for each different MRI session). This acquisition is necessary for the preprocessing (registration) of the functional imaging data and will also be collected in electronic format. Consequently, the following data will be collected:

- *For non-expert seniors:* two acquisitions in T2*.
- *For the experts:* three acquisitions in T2*.

In addition, the following will be collected during activation fMRI examinations:

- Behavioural performance on the AX-CPT task (accuracy measure and reaction time)
- The results of the post-scan debriefings of the rest-SoVT task (video judgment scores)

All these acquisitions will be collected on the MRI of the Cyceron Center.

10.1.2.2. PET scan

The PET data collected will include:

- An early acquisition in PET-Amyvid®, allowing a measurement of cerebral perfusion;
- A late acquisition in PET-Amyvid®, allowing a measurement of the binding of the marker to amyloid deposits;
- A PET-^{18}F-FDG acquisition, allowing a measurement of the cerebral metabolism.

These acquisitions will all be collected on a high spatial resolution camera. An attenuation correction will be made from an estimate of the attenuation map by X-ray scanner acquired with a low dose protocol. The resulting images will be collected in electronic format to be pre-processed and processed with brain imaging software.

10.1.2.3. Blood sample on V1

Numerical results of the various marker assays from the V1 blood sample (see Section 7.3.4, Table 8) will be collected from all participants.

GWAS and APOE genotyping will be collected for all participants who have signed the specific consent; these results will be communicated to the investigators via the neurologist in charge of the project.

10.1.2.4. Neuropsychological and behavioural assessment on V1

The neuropsychological data collected during the V1 neuropsychological and behavioural assessment correspond to the various neuropsychological test scores (see section 6.7, table 5). They correspond to scores, most of the time numerical, single or multiple depending on the tests with:

- responses times
- and/or the number of correct answers
- and/or the number of errors
- with total scores and sub-scores

10.1.2.5. Assessment of wakefulness and sleep

The assessment of wakefulness and sleep will include the collection of

- Scores on various wakefulness and sleep questionnaires;
- Actimetry measurements: this is a continuous recording of the activity-rest rhythm. This recording makes it possible to calculate a fragmentation index of the activity-rest rhythm;
- Polysomnographic recordings: this is an EEG recording during wakefulness and two nights of sleep. It allows us to collect different information on the reactivity of the brain during the presentation of auditory stimuli, the spectral profile and the functional connectivity during the awake state. For the sleep recordings, we will collect in particular the sleep latency, the sleep efficiency (time spent sleeping / time spent in bed), the sleep duration, the number of nocturnal awakenings, the percentage and the time spent in each sleep stage;
- With the Somno-Art® device: total sleep time, sleep efficiency, sleep latency, WASO (wake after sleep onset), quantities of different sleep stages, number of cycles, number of awakenings, number of transitions between stages and sleep instability index;
- With the consolidation task: the rate of improvement or forgetting during the night.

10.1.3. Data collected during the intervention

During the intervention, information will be collected on the participants' diary, including the duration of the daily exercises, the evaluation of their difficulty, etc. (numerical and qualitative data), the therapeutic alliance between the participant and the teacher, and the quality of the social ties forged in the group and between the participant and their partner during the intervention.

When the subject fills in his daily questionnaire on the iPad, the data are retrieved in coded and pseudo-anonymized text format on his tablet. The data is never transmitted via the Internet. The functionalities of the iPad which are not used for the trial are deactivated (Internet, etc.). Restrictions are put in place for access to the Internet browser and the "App store" from Apple tablets. To do this, the following procedure will be applied: General/restriction/activate restrictions/enter a code. The access codes to change these settings will not be known to the participants. They will therefore have no way of downloading new applications or surfing the Internet via these tablets and consequently the data collected will not be transmitted via the Internet. To connect to their tablet, participants must enter a code that is at least 7 characters long, including at least one special character or letter. The validity period is less than 12 months. The number of unsuccessful attempts is 3 before the account is blocked. Participants must then bring their tablet to the IT staff in charge so that they can restore access. This application has been developed by a certified staff: Pierre-Emmanuel AGUERA is a study engineer (IE2) at INSERM in the Centre de Recherche en Neurosciences de Lyon, in the DYCOG team, INSERM U1028 - CNRS UMR5292 (pe.aguera@inserm.fr). Mr. Aguera is also a certified developer at Apple. Once a month, the English and meditation intervention subjects bring their tablets to class to be processed by a computer scientist from the English and meditation workpackage team, respectively.

The data is then downloaded to a computer exclusively for this purpose and the data is deleted from the tablets. Throughout this process, the data remains pseudo-anonymized. During the transfer of the data, the new teaching material used in the interventions is downloaded to the participant's tablet. The data, once decoded by a computer expert, will then be transmitted to the data management structure via a secure file transfer platform and attached to the global database in order to analyze them in relation to the other modalities.

10.1.4. Data collected at the 9-month follow-up visit (V2)

The neuropsychological data collected during the V2 neuropsychological and behavioural assessment correspond to the various neuropsychological test scores (see section 6.7, table 5). They correspond to scores, most of the time numerical, single or multiple depending on the tests with:

- response times
- and/or the number of correct answers
- and/or the number of errors
- with total scores and sub-scores

10.1.5. Data Collected at the 18-Month End-of-Intervention Visit (V3)

The data collected at the 18-month follow-up visit (V3) will be similar to that collected at the inclusion visit (V1). This visit will include, in addition to these examinations, a semi-structured interview about the experience of English learning and meditation during their participation in the Age-Well trial. The qualitative data from this dictaphone-recorded interview will then be transcribed in full. A classical content analysis will then be performed (manual processing and/or use of software). This qualitative interview will only concern participants who have attended an intervention.

The qualitative data from the interview with the meditation and English teachers after the end of the interventions will be recorded with a dictaphone and then transcribed in full. A classical content analysis will be performed.

10.2. *Data collected at the 29±6 month post-procedure follow-up visit (V4)*

The neuropsychological data collected during the V4 neuropsychological and behavioural assessment correspond to the various neuropsychological test scores (see section 6.7, table 5). They correspond to scores, most of the time numerical, single or multiple depending on the tests with:

- response times
- and/or the number of correct answers
- and/or the number of errors
- with total scores and sub-scores

<u>This visit will include the same examinations as in the V1 inclusion visit and in the V3 post-intervention follow-up visit except for the PET-Amyvid examination and the overnight polysomnography.</u>

Anthropometric data

In order to improve the quality of anthropometric measurements (height, weight, BMI, abdominal girth and hip circumference), the acquisition of a body composition scale (Tanita MC-580) was made to allow accurate measurement of: Weight (kg), fat (%), body fat (kg), lean body mass (kg), hydration rate (kg), muscle mass (kg), bone mass (kg), impedance (Ohm), basal metabolic rate (kJ; kcal), visceral fat (level) and metabolic age (year), as well as BMI, Sarcopenic Index (SMI; kg/m²) and Skeletal Muscle Mass (SMM; kg and %).

The CIRS score (Cumulative Illness Rating Scale)

The CIRS score is calculated from medical (history, treatments), biological (Body Mass Index (BMI), Blood Pressure (BP), Blood Count (CBC), Lipid Abnormality Investigation (LAI), Fasting Blood Glucose), neuropsychological (Mini-Mental State (MMS)), and the Apnea-hypopnea index (AHI) score from polysomnography, collected at visits 1, 3, and 4 entered in the eCRF. It provides a global numerical measure of all the pathologies of an individual.

10.3. *Definition of source data*

Source data are all the information contained in original documents, or in authenticated copies of these documents, which relate to clinical examinations, observations or other activities carried out in the framework of biomedical research and which are necessary for the reconstruction and evaluation of the research. The documents in which the source data are recorded are called source documents, regardless of the medium used (paper, electronic...).

10.4. *Data circuit before computer entry*

Regarding the consistency check of the collected data, they are retrieved once a month for each participant in the English group and the meditation group. On this occasion, we can verify that the tablet is working as intended. In addition, we record the participants' data via the Kallysta case and the Dapi software installed on a MacBook computer that is not connected to the internet or any intranet and is password protected. We then check that the participants fill in the questionnaire according to our expectations (once a day). This is the first method that allows us to know that the use of the tablets by the participants is consistent with our expectations. The second method we use goes into a little more detail about the data recorded. In particular, we track practice time by verifying that, on the day of the weekly course, participation is around 2 hours on average for the participants. We can then confirm that participants are filling out their tablets as we explained to them. If this is not the case for a participant, we can re-explain the instructions for correctly filling out the questionnaire on the tablet.

As for the maintenance of the equipment, tablets, computers and briefcases, this is carried out by the computer specialists of the center where the equipment is located: the computer specialists of the Carré international for the equipment attached to the English learning intervention and the Cyceron center for the equipment attached to the meditation intervention. In both cases, the equipment is stored in locked cabinets, in locked rooms. In addition, to enter these buildings, it is necessary to use a biometric code to gain access.

Concerning the traceability of the data, they are recovered by wire from the tablet and without the need for an internet network. The saved file has the date of recording and data collection.

These data files collected via the tablets will then be decrypted and formatted at Cyceron by a member of Antoine Lutz's team and transferred to an Excel spreadsheet. This Excel table will then be sent in a secure way to the data management team who will keep this file for later availability with the database from the eCRF, the anonymization code being the element that allows to match the collected data to the right participant.

Data will be collected by the referents and investigators throughout the protocol. The information collected will be kept in the source file and the scores/information collected will be entered into the e-CRF by the referents and investigators.

The imaging data will be acquired on the Cyceron Center's imaging equipment and transferred in electronic format via the Center's internal network to the protocol investigators. The volume and perfusion of the anterior cingulate cortex and insula will be obtained after transformation of the data via SPM and/or Freesurfer and extracted to be entered into the eCRF.

For biological data, blood samples will be sent directly from the CRC of the Caen University Hospital to the different research laboratories in charge of the analyses (see section 7.3). Once the results of the different analyses have been received at the CRC, the data will be sent pseudo-anonymized with the participant code of the trial to the Inserm referent for entry into the eCRF.

Cognitive data will be collected directly in paper and electronic format and scored by the referent and/or neuropsychologist.

Finally, actimetry and polysomnography data will be directly retrieved in electronic format by the investigators.

10.5. *Computerized data entry*

Until December 31, 2020, the eCRF and database are the responsibility of EUCLID. After this date, the frozen database with data from inclusion to the end of intervention visit (V3) will be transferred to and under the responsibility of the IT department of the Cyceron Center in Caen. The Cyceron Center, through the MMA funding, will call upon the easyCRF service provider who will develop the part of the eCRF corresponding to the data collected during the follow-up visit at 29 months post-intervention (V4), the inclusion visit of the experts (V1) and who will consolidate the database and ensure its security.

Software used

EUCLID: The software used for data management is Ennov Clinical, from Ennov, in its version 7.0 or any later version. This software has been developed by Ennov-Clinical in compliance with the FDA Guidance for Computerized System Used in Clinical Trials, electronic signature and audit trail (21CFR Part 11) as well as international standards (CDISC, ICH, GCP 2001/20/EC...).

easyCRF: The software used for data management is Easy-CRF, from the company Easy-CRF, in its version 6.2.0 or any later version. This software has been developed by Easy-CRF in compliance with the FDA's Guidance for Computerized Systems Used in Clinical Trials (2007), electronic signature and audit trail (21CFR Part 11) as well as ICH, GCP 2001/20/EC.

Data hosting

EUCLID: Ennov-Clinsight ensures the storage and maintenance of the database in accordance with the specifications defined in detail in the Ennov Clinical Supplier CDC and Ennov-Clinsight procedures. OVH hosts the server in a data center located in Roubaix (59), France.

easyCRF: The company Easy-CRF ensures the storage and the maintenance of the database according to its own procedures (backup, restoration, disaster recovery plan, change management). The company AZNetwork hosts the server in a data center located in France in Alencon (61).

Data safety

EUCLID: Ennov-Clinsight ensures the security of the hosted database. The local server is located in Strasbourg, France and an external backup server is located in Gravelines. According to the rules described in the standard operating procedure, the management of access rights is the responsibility of the EUCLID CS administrator and the management of access to the eCRF for investigators and their collaborators is the responsibility of the EUCLID ARC.

easyCRF: Easy-CRF has implemented a certain number of logical measures to ensure the security of the data located on its server (firewall, antivirus, rootkit detection, intrusion detection). The physical protection measures are implemented by AZNetwork in accordance with the specifications for hosting health data. A login/password access is associated to each user. The rights of each user are configured according to their profile (promoter, investigator, CRA, etc.) and their auditable values. A trace of connections, accesses to the different pages of the eCRF, and registrations is kept for the entire duration of the study's operation.

Data entry

Trial data should be entered by the investigator or delegate, who should date and sign the eCRF. If the investigator delegates and authorizes other staff members to make entries in the eCRF, their names, functions, signatures and initials must be documented in writing (e.g., Center Delegation Form). The eCRF must be completed during/after each trial visit.

All individuals responsible for entering data into the eCRF must be trained and designated to perform this task.
Access to the eCRF for data entry or review will require a separate individual access code assigned only to center staff members who will be entering trial data and to members involved in trial monitoring who may be reviewing trial data. Data are collected in the eCRF, to which the sponsor and monitor have "read only" access.

Database archiving

The archiving of the database is the responsibility of the sponsor.

10.6. *Description of data processing, verification and validation methods (data management)*

10.6.1. Processing of eCRF data

Data validation

Consistency tests are scheduled by the data manager to check the consistency and the data entry in the eCRF. The list of consistency tests is predefined by the trial project team, then transmitted to the data manager who writes the validation plan for the data.
The management of correction requests is done by the data manager in due time. Additional correction requests can also be made by the sponsor's CRA. Correction requests are sent to the investigating center via the eCRF. The investigators respond directly in the eCRF.

Data coding

Treatment and clinical events are coded in the eCRF by the sponsoring ARC to monitor and analyze the data.
The following dictionaries are used to code medical verbatims:

- MedDRA: *version 18.1, for the coding of pathologies*
- WHODRUG: *June 2016 version, for treatment coding*

Database freeze

The database must be frozen in order to perform the final analysis. A review of the data is performed before the database is frozen by the entire project team in charge of the trial.

Data transfer

Data transfers (sending, receiving) are carried out in accordance with the procedure in force. The data transfer procedures must be defined in the Data Management Plan. For security reasons, data files are pseudo-anonymized and then transferred via a secure platform.

10.6.2. Preprocessing of the imaging data

Anatomical MRI data

- **MRI-T1**. The data will be pre-processed in order to i) isolate the voxels (3D pixels) corresponding to the cerebral grey matter, and ii) to "normalize" the brains (deform them so that they are superimposable and therefore comparable between individuals) via the SPM12 software (Statistical Parametric Mapping; Wellcome Dept of Cognitive Neurology, London, UK). These data will then be used to obtain indices of cortical volume or thickness. In addition, T1-MRI data will be used as a "support" for the analysis of most other imaging modalities (fMRI, DKI, PET...).

  Among others, volume values of the anterior cingulate cortex and insula will be extracted from the pre-processed MRI images ("region of interest" analysis) in order to perform the analyses to address the primary evaluation criterion.
- **MRI-FLAIR**. The data will be preprocessed to extract the volume, number and location of white matter hyperintensities using WHASA software (Samaille et al., Plos One 2012).
- **MRI-T2-HCP**. These high-resolution hippocampal-targeted images will allow for segmenting, and thus calculating the volume of hippocampal subfields, both by manual tracing following a protocol developed (La Joie et al., Neuroimage 2010) and used (La Joie et al., Neuroimage Clinical 2013; de Flores et al., HBM 2015) in the laboratory and an automated procedure via ASHS software (Yushkevich et al., HBM 2015).
- **MRI-DKI**. Diffusion imaging data (DKI) will allow, after data processing with FSL (Jenkinson et al., Neuroimage 2012) , ACID (SPM Toolbox; Mohammadi, 2010) and DKE (Jensen et al., NMR Biomed 2010; Tabesh et al., Magn Reson 2011), to reconstruct axonal fiber bundles and calculate diffusion and "kurtosis" parameters within the white matter. These data will thus provide clues to the structural connectivity.
- **MRI-QSM**. Magnetic susceptibility imaging data will be processed using imaging software. The magnetization vector phase information will be used in the form of a mask applied to the magnitude images to enhance the contrast and thus highlight susceptibility differences between tissues (Acosta-Cabronero, 2013).
- **MRI-ASL.** The perfusion imaging data will be processed with imaging software. These data will allow after processing to calculate an index of cerebral blood perfusion.
- **MRI-HR-DTI-Locus.** High-resolution imaging data will be processed with imaging software to better assess the links between hippocampal subregions and locus coeruleus.

The processing of these acquisitions with imaging software will thus allow to obtain cerebral "maps" or average values extracted in certain regions (analysis in regions of interest) which will allow to analyze the cerebral anatomy of all participants with the help of different indices: density/volume of grey and white matter, cortical thickness, hippocampal subfields, white matter bundles, magnetic susceptibility of tissues... and compare brain anatomy between groups or within the same group before and after the intervention.

Functional MRI data (at rest and in activation)

The different steps of pre-processing of the fMRI images will be performed with the SPM12 software. This procedure includes the realignment of the different volumes acquired: correction of movements during the functional acquisition (realignment of the different functional volumes on the first functional volume acquired), correction of the temporal shift ("Slice Timing" function of the SPM12 software), realignment of the functional images on the corresponding T1-MRI, spatial normalization and data filtering (see Villain et al., 2010).

- **fMRI at rest:** Co-variance maps (ICA maps) and functional connectivity maps for each participant (first level analyses) will be made.
- **Activation fMRI:** the variations of the BOLD signal amplitude in the different experimental conditions will be modeled in a first level analysis in each participant in order to reflect in each participant the neural activity specific to a particular cognitive activity (= activity specific to an experimental condition).

The imaging treatments will thus allow to have activation "maps" for each acquisition. The average activity over the whole sequence will be evaluated for the sequences at rest and can thus be compared between groups or within groups before and after intervention. For the activation sequences, the neuronal activity in the different experimental conditions will be isolated in order to be able to compare the cerebral substrates of each of them, between the groups and before versus after intervention.

PET data

PET data will initially be corrected for partial volume effects using a three-compartment method (Giovacchini et al., J Nucl Med 2004), implemented in the 'PMOD' software (PMOD Technologies Ltd., Adliswil, Switzerland) and already used in the study of older adults in the laboratory (La Joie et al., J Neurosci 2012; Besson et al., J Neurosci 2015). The remaining pre-processing operations will be performed using SPM12 software. The corrected PET data will first be realigned to the corresponding anatomical MRI images. The spatial normalization of the PET images will be optimized by applying the normalization parameters of the corresponding T1-MRI. The PET data will then be quantitatively normalized individually by dividing each value by the average value of a so-called "reference" region (e.g. cerebellum, white matter) determined by the corresponding T1-MRI and an anatomical atlas (Tzourio-Mazoyer et al., Neuroimage 2002); the final image will thus be a Standardized Uptake Value Ratio (SUVR). The reference region may vary according to the analyses and will be chosen because of its preservation in terms of beta-amyloid deposits, tau pathology and hypoperfusion.

- **PET-Amyvid® *early-frame*.** Data acquired within 10 min after tracer injection will be preprocessed as described by Hsiao et al (EurJNuclMedMolImaging 2012) to create brain perfusion maps that will be analyzed in the whole brain (voxel-to-voxel analysis via SPM12) or by an analysis method in regions of interest.

  In addition, volume values of the anterior cingulate cortex and insula will be extracted from the pre-processed MRI images ("region of interest" analysis) to perform the analyses to address the primary evaluation criterion.

- **PET-Amyvid®** ***late-frame.*** The data acquired within 50 min after tracer injection will be preprocessed to create maps of amyloid deposition labeling that will be analyzed in the whole brain (voxel-to-voxel analysis via SPM12) or by an analysis method in regions of interest. Furthermore, the preprocessed data will allow to obtain a global cortical labeling value after extraction of the average SUVR value in a mask previously developed in the laboratory (Besson et al., J Neurosci 2015) in order to interrogate regions particularly susceptible to beta-amyloid deposits.
- **PET-^{18}F-FDG.** Data acquired within 50 min after tracer injection will be preprocessed to create maps of radiolabeled glucose labeling that will be analyzed in the whole brain (voxel-to-voxel analysis via SPM12) as well as by a method of analysis in regions of interest corresponding to the most affected regions in AD (the posterior cingulate and temporo-parietal cortex; Besson et al., J Neurosci 2015).

EEG data (polysomnography)

Awake EEG data will be processed with ELAN tools developed in the Brain Dynamics and Cognition laboratory (Aguera et al. 2011, http://elan.lyon.inserm.fr), as well as using SPM12 software (Welcome Department of Cognitive Neurology, London, UK; http://www.fil.ion.ucl.ac.uk/spm). Spectral analysis of the continuous EEG signal will be done using time-frequency analysis programs developed in the team. Muscle artifact rejection will be done using the EEGlab program (Makeig and Delorme 2004; http://sccn.ucsd.edu/eeglab/).

Sleep EEG data will be analyzed by a sleep neurologist according to international criteria (Berry et al., 2014). Total sleep time, sleep latency, sleep efficiency, number and duration of nocturnal awakenings, and time spent in each sleep stage will be determined. More detailed analyses of the recordings will also be carried out by means of spectral analyses, which make it possible to highlight modifications of the cerebral activity in certain frequency bands. We will also calculate the density of different oscillations (slow waves, sleep spindles, ...) known to be involved in the process of memory consolidation during sleep and sensitive to the effects of age and Alzheimer's disease.

For the pre-processing of all brain imaging data (MRI and PET), the investigators have the possibility to use new software (or updates of the aforementioned softwares) developed before the pre-processing and data analysis phase in order to guarantee an optimal use of the imaging data.
The data obtained with the Somno-Art® device will be analyzed by the company PPRS, which developed the device and the processing algorithms. The algorithms will collect the following information for each night: total sleep time, sleep efficiency, sleep latency, WASO (wake after sleep onset), quantities of the different sleep stages, number of cycles, number of awakenings, number of transitions between stages and sleep instability index.

10.6.3. Verification of imaging data

The quality of the imaging data will be routinely assessed, in the days following acquisition, according to standard laboratory procedures. Each image is first checked visually by an investigator with expertise in this imaging modality. Specific procedures are also implemented for specific modalities:

- **fMRI:** the verification of movements and susceptibility artifacts will be performed both by visual analysis and via dedicated toolboxes such as the "TSDiffana" tool (http://imaging.mrc-cbu.cam.ac.uk/imaging/DataDiagnostics).

- **PET Scan:** the dose of product injected and the acquisition time window (0-10 min post-injection and 50-60 min post-injection for PET-Amyvid® early-frame and PET-Amyvid® late-frame respectively; 50-60 min post-injection for 18F-FDG PET) will be checked.

Finally, a procedure integrated into the SPM software will make it possible to detect, once the data have been pre-processed, extreme data ("outliers", likely to be defective data due to an anomaly during acquisition or processing) via the software *check sample homogeneity* (http://www.neuro.uni-jena.de/vbm/check-sample-homogeneity/).

10.7. *Management and archiving conditions of the trial documents*

Trial documents will be archived during and after the trial at the Cyceron Center in a locked filing cabinet.

11. STATISTICAL ANALYSIS OF DATA

11.1. *Responsibility for statistical analysis*

The statistical analysis of the main objectives will be performed by the EUCLID statistician (EUropean CLInical trials platform and Development, CHU Bordeaux and Inserm, F-CRIN).

The statistical analysis of the secondary objectives, including the neuroimaging data, will be carried out within the work packages dedicated to the evaluation of the different objectives, as follows:

- Objective 2: WP 1 to 6;
- Objective 3: WP 1 to 6;;
- Objective 4: WP 1 to 6;
- Objective 5: WP 1;
- Objective 6: WP 2 to 6;
- Objective 7: WP 6
- Objective 8: WP 6
- Objective 9: WP 1 to 6.

The EUCLID statistician will be involved in the definition of the secondary statistical analyses and will be able to assist the WPs in their realization .

11.2. *Calculation of the study size*

Study on non-expert seniors:

The comparison between the meditation group and the no-intervention group is going to focus on the difference in 1) volume and 2) perfusion of the anterior cingulate cortex 18 months after the start of the intervention compared to the value at inclusion, with an expected effect size of 0.75 (effect size of short-term meditation vs. no intervention, from a meta-analysis on the effects of meditation on brain imaging markers: Fox et al, 2014). Given that the intervention in the current protocol is 18 months, we consider that the effect size will be at least equal to 0.75.

The same effect size of 0.75 is expected for the comparison between the meditation group and the English learning group on the difference in 1) insula volume and 2) insula perfusion 18 months after the start of the intervention compared with the inclusion value.

To show an effect size of 0.75 for each comparison, with 80% power and a two-sided first-species risk of 1.25% (Bonferroni correction because four comparisons will be performed) 42 participants per group must be recruited for a total of 126 participants.

Assuming that the effect size assumptions are conservative and that precautions are taken at the time of recruitment to best ensure adherence of participants to the trial, we expect that the risk of dropout after knowledge of the randomization group is very low and that the power of the study is preserved. However, we will implement close monitoring of these refusals at the beginning of the trial. In order to increase the statistical power of the secondary analysis on the FDG-PET examination carried out from

the second wave of inclusion, 24 participants will be recruited in addition, i.e. 8 additional participants per group (meditation, English, passive control) for a total of 150 participants.

Study on the expert meditators:
Studies of expert meditators assessing meditation-related changes in brain activity have shown an effect size of d=0.74 (calculated for fMRI change in the insula in response to emotional stimuli; Lutz et al., 2013).
For reasons of recruitment capacity, it was agreed to recruit 30 expert meditators into the trial.
The inclusion of 30 expert meditating participants will allow us to have a power of 95% for the comparison to the 126 non-expert participants, with a first species risk of 5%, to show an effect size of 0.74 in the comparison of brain imaging data.
For the comparison of the 30 expert participants to the 126 non-expert participants, we will have 90% power to show an effect size of 0.67 and 80% power to show an effect size of 0.58, with a two-sided first species risk of 5%.

11.3. *Description of the statistical analysis plan*

This protocol aims to determine the effects of a long-term meditative practice on biological and behavioral markers notably associated with aging and Alzheimer's disease (AD), and to better understand the cognitive and emotional mechanisms underlying these effects. To meet the main and secondary objectives (see part 2), a set of statistical analyses will be conducted.

The statistical analysis plan will be the subject of a complete and specific document separate from the protocol. The main elements are included in this chapter.

11.3.1. Analysis strategy

The main analysis will be carried out on an intention-to-treat basis, i.e. all randomized patients will be included in the analysis in the group in which they were initially randomized, and all their data will be used regardless of their changes in strategy during the research, with the "missing = failure" strategy, i.e. any missing value will be replaced in the analysis by the value corresponding to failure (minimum difference between 18 months after the intervention and inclusion), estimated on the data observed in all groups combined.

In order to assess the robustness of the results, a sensitivity analysis to missing data will be performed using the maximum bias strategy by using the values reflecting the failure and success (minimum and maximum difference respectively) estimated on the observed data for all groups combined.

A sensitivity analysis will also be performed by including only participants who attended at least 20% of the meditation sessions and exercises and who completed the examinations for the primary judgement criterion.

Some features may be strongly associated with volume and perfusion of the anterior cingulate cortex or insula. These characteristics could play a confounding role if, despite randomization, an imbalance was observed. Therefore, the comparative analysis of the primary judgemental criteria could be adjusted for potential confounders such as age, education, and gender. These adjustments may require the use of appropriate models, the relevance of which will be discussed according to the distribution and type of variables.

The description will always be done globally and by treatment group.

Tests for the primary criteria will be performed at the risk of error of the first kind α= 1.25%.

Tests for secondary criteria will be performed at the overall risk of error of the first kind α= 5%.

11.3.2. Descriptive statistical methods

Categorical variables will be described in terms of numbers, percentages and possibly 95% confidence intervals according to the exact binomial distribution.

Quantitative variables will be described in terms of number, mean, standard deviation, possibly 95% confidence interval of the mean, median, range and interquartile range.

11.3.3. Participants included in the analysis

Only participants who meet at least one of the following conditions may be excluded from the analysis:

- Participants who never followed the research procedure (provided they were not aware of the group to which they were randomized),
- Participants wrongly included for unsigned consent,
- Participants who withdrew their consent.

This exclusion decision will be taken by the Scientific Council of the trial after documentation of the observation by the Methodology and Data Management Center (EUCLID), without regard to the strategy group and the participant's evolution after inclusion.

Apart from these exclusions, all participants who died, were lost to follow-up, or dropped out of the research will be included in the analysis.

11.3.4. Characteristics of the participants

Participants will be described according to the following variables:

- Compliance with eligibility criteria;
- Epidemiological characteristics (demographics, disease history, and characteristics at inclusion);
- Clinical characteristics;
- Biological characteristics.

A description of the protocol violations and the participants distributed according to these violations will be provided .

A description of causes of death and dropout will be made and participants who died, were lost to follow-up, or dropped out of the research will be described and compared to other participants.

Epidemiological characteristics at inclusion of meditating seniors will be compared to non-expert seniors.

11.3.5. Analysis of the main criteria

The comparison between the meditation group and the foreign language learning group will look at the difference in 1) volume and 2) perfusion of the anterior cingulate cortex 18 months after the start of the intervention compared to the value at inclusion.
The comparison between the meditation group and the no-intervention group will look at the difference in 1) insula volume and 2) insula perfusion 18 months after the start of the intervention compared to the value at baseline.
The main criteria will be compared between groups by Student's t test or Wilcoxon test if the hypothesis of normality of the criteria is not verified.

An analysis of the effect of the level of exposure to meditation (number of hours of practice, number of sessions, etc.) will be performed by including all participants.

11.3.6. Analysis of the secondary criteria

The qualitative criteria will be compared between groups by the Chi-square, adjusted Chi-square or Fisher exact test, depending on the expected number of participants under the assumption of independence. Logistic regression models will be used to adjust the comparison for confounding factors where appropriate. The conditions of application of the regression models will be verified.

Quantitative criteria will be compared between groups by Student's t test or Wilcoxon test if the normality of the criterion is not verified. Transformations to normalize the criterion can be performed if necessary. Linear regression models will be used to adjust the comparison on confounding factors if necessary. The conditions of application of the regression models will be verified.

Concerning the imaging data, voxel-to-voxel analyses will be conducted using SPM software, either by using intergroup and intragroup comparison statistics (Two-sample T-test, Full Factorial Design, Flexible Factorial Design... to study differences between different groups at the same visit), and regression (Multiple regression, to study the impact of variables of interest on our measures), or by applying specific and elaborate methods for longitudinal analyses, for which the investigators have developed experience, before performing repeated measures ANOVA. Analyses in regions of interest can also be performed by extracting values (volume, perfusion, amyloid marking, etc.) from the brain maps obtained in the different imaging modalities in order to perform statistical analyses on specific regions.

All analyses will be adjusted for potential confounding factors, which will be identified for each analysis (e.g., age, education level, gender).

11.3.7. Other analysis

The database will be frozen when 1/3, 2/3, and all participants are recruited in order to allow analyses that will be defined a priori in the statistical analysis plan. Additional analyses will be performed on the data acquired at inclusion with regard to the secondary objectives defined in the protocol.

Further analyses will be conducted by the investigators based on (i) the advancement of knowledge in this area and (ii) the results of the analyses that meet the primary and/or secondary objectives outlined above. For example, if a gender effect (male/female) is found on the effects of the intervention, post-hoc analyses will be conducted to better understand the mechanisms of these differential effects.
These analyses may be performed as data become available at the request of the investigators, after prior validation by the Scientific Council. They must be integrated in the statistical analysis plan of the trial.
A record of all tests performed will be maintained by the sponsor.

12. Communication

12.1. *Terms and conditions for the publication of results*

All data collected in the course of this research are the property of the sponsor and may not be disclosed to any third party without the written consent of the investigator.

The results are published in the form of scientific articles in peer-reviewed journals and presented at national and international conferences. Any publication or communication (oral or written) is decided by mutual agreement between the coordinating and principal investigators as well as the scientific managers and will respect the international recommendations: "Uniforms Requirements for Manuscripts Submitted to Biomedical Journals" (http://www.cma.ca/publications/mwc/uniform.htm).

All publications must follow the rules set out in the AVIESAN publication charter. The mention of the origin of the funding, the authorizations of the competent authorities, the consent of the participants must appear in the acknowledgements according to the model suggested below: */Ethics statement /*/This study is part of clinical trial ****CXX-XX** sponsored by Inserm. It was granted approval by local Ethics Committee or “Comité de Protection des Personnes” on ---****DATE**---, authorized by the French authorities (****ANSM** ****NB**), and registered in a public trials registry (****CT XXXX**).

All participants gave their informed, written consent to participation, in line with French ethical guidelines.

The signatories of the publications are the people who actually participated in the elaboration of the protocol, its progress and the writing of the results. The executive committee of the European consortium "Medit-Ageing" (ExCom) has the task of deciding on the analyses and publications carried out as well as on the signatories of the publications resulting from this protocol.

12.2. *Procedures for writing the final report*

The final report of the research will be written, in collaboration, by the coordinator and the biostatistician of this research. This report will be submitted to each of the investigators for review. Once a consensus has been reached, the final version must be endorsed by the signature of each investigator and sent to the sponsor as soon as possible after the research has been completed. A report written according to the reference plan of the competent authority must be sent to the competent authority and to the CPP within one year after the end of the research, understood as the last follow-up visit of the last participant included. This time limit is reduced to 90 days in the event of premature termination of the research.

12.3. *Procedures for informing participants about the overall results of the research*

Individuals who participated in the trial may, if they wish, be informed of the overall results of the research by the coordinating investigator after the research is completed.

Articles and abstracts from this study will be sent to participants who wish to receive them.

12.4. *Procedures for informing individuals about health data during and after research*

Individuals may be informed at any time of the health data concerning them, upon written request to the coordinating investigator.

Any clinically significant abnormality detected in the examination or test results will be communicated to the participant and the physician previously selected by the participant.

12.5. *Terms and conditions relating to press communication*

The articles and abstracts resulting from this study will be sent before publication to the executive committee of the European "Medit-Ageing" consortium (ExCom) where the Clinical Research Unit is represented and the Department of Scientific Information and Communication (DSIC) will also be kept informed.

13. Confidentiality

13.1. *Terms of confidentiality with respect to the individuals*

The information required by the protocol will be collected in the eCRF and an explanation will be given for each missing data. Data will be completed in the eCRF as they are obtained whether they are clinical or para-clinical data. Pseudo-anonymization of participants will be ensured by an anonymization code on all documents required for the research. Computerized data on a file will be declared to the CNIL according to the procedure adapted to the case

13.2. *Terms of confidentiality with respect to the trial*

Access to clinical and source data will be direct in case of monitoring, audits commissioned by the sponsor, and/or inspections by competent administrative authorities.

14. Protection of persons

14.1. *Ethical rationale of the protocol*

Anticipated benefits: For the participants, there is a direct benefit to participating in this trial. They will benefit from very good care, from an in-depth follow-up over 18 months thanks to this protocol. For all the volunteers, they will contribute to improving the understanding of Alzheimer's disease and its early detection, the impact of life factors, the effects and mechanisms of mental training such as meditation or learning a foreign language on the mental health and well-being of the older adults.

Predictable risks:

Neuropsychological tests: they do not involve any risk to the participant.

MRI: as performed in this trial (i.e. without injection of contrast medium), MRI has no known risks; the only inconvenience is to have to lie down without moving the head during the examination.

PET Scan: exposure to ionizing radiation is low and does not present a significant risk (the estimated dosimetries are reported below). The main inconveniences are the insertion of a venous catheter and lying down without moving the head for about 20 minutes during the acquisition of images. Before each PET scan, renal and hepatic functions will be evaluated, and, in addition, a blood sugar control will be performed before the FDG-PET scan.

For Amyvid®: The probability of occurrence of cancer or hereditary abnormalities is low. No serious adverse events related to the administration of Amyvid® have been reported. The most frequently observed adverse events are headache (infrequent events are described in the Amyvid® Product Monograph).

For the FDG-PET:

For Glucotep®, exposure to ionizing radiation may induce cancer or the development of hereditary deficiencies. As the effective dose is 7.6 mSv for a maximum recommended activity of 400 MBq, the occurrence of these adverse effects is unlikely (see SPC).

Polysomnographic, Somno-Art® and actimetric recordings: these recordings present no risk to the participant.

Dosimetry:

The injected activity of Amyvid® is optimized and equal to 4 MBq/kg. The effective dose for Amyvid® is estimated to be 18.6 µSv/MBq (SPC data). The effective dose resulting from the administration of a 280 MBq dose of Amyvid® for a 70 kg adult is therefore less than 5.5 mSv. For an administered activity of 280 MBq, the dose delivered to the target organ (brain) is 2.8 mGy.

The effective dose of FDG is 0.019 mSV/MBq, i.e. here 3.80 mSv for an injected activity of 200 MBq. The dose absorbed by the bladder (critical organ) is 0.13 mGy/MBq, i.e. 26 mGy for 200 MBq injected (data according to the Glucotep® SPC).

The participant will be asked to hydrate and urinate approximately one hour before the start of the examination, and then immediately afterwards. These precautions allow us to reduce as much as possible the quantity of product present in the bladder and thus the irradiation of this organ. The duration of the examination is approximately 1h30 including the installation of the volunteer, the injection of the radiopharmaceutical and the acquisition of the images. The acquisition of the PET images lasts 10 minutes and then 20 minutes for the PET-Amyvid® and 10 minutes for the PET-^{18}F-FDG during which the participant must remain immobile, preceded by the acquisition of the X-ray scanner for the attenuation correction.

For the PET scan, the attenuation correction is made from an estimate of the attenuation map by X-ray scanner acquired according to a low dose protocol. The scanner acquisition field is limited to the brain, and includes the lens which (1) cannot be protected and (2) is mainly irradiated by scattered radiation. For the parameters used (120 kV, 10 mA, rotation time 0.5 s, pitch of 1.375, collimation: 4x3.75 mm), the effective dose and equivalent dose for the lens are respectively 0.038 mSv and 0.75 mSv for an examination.

The following summarizes the total effective radiation doses received by participants during the entire trial:

Visits	PET Scan	Male (mSv)	Female (mSv)
Inclusion (V1)	PET-Amyvid®	5,32 + (2 x 0,038)	5,32 + (2 x 0,038)
	PET-^{18}F-FDG	3,80 + 0,038	3,80 + 0,038
18 months later (V3)	PET-Amyvid®	5,32 + (2 x 0,038)	5,32 + (2 x 0,038)
	PET-^{18}F-FDG	3,80 + 0,038	3,80 + 0,038
29 ± 6 months post-intervention (V4)	PET-^{18}F-FDG	3,80 + 0,038	3,80 + 0,038
Total for the whole trial		22,306	22,306

14.2. *Ethical and regulatory provisions*

The research will be carried out in compliance with the French regulations in force, in particular the provisions relating to biomedical research of the Public Health Code, article L 1121-1 and following, the Bioethics laws, the Data Protection Act, the Helsinki Declaration, as well as the Good Clinical Practices and the present protocol.

The investigator agrees to conduct the research in accordance with these ethical and regulatory provisions. They are aware that all documents and data relating to the research may be subject to audits and inspections carried out in compliance with professional secrecy and without any possibility of medical secrecy being invoked. The investigator acknowledges that the results of the research are the property of Inserm, the promoter of the research.

14.3. *Comité de Protection des Personnes (CPP)*

Before implementing the research, the sponsor will submit the project to the local CPP for its opinion and will provide it with all the necessary information (research protocol, data collection medium (adapt this term to the research - observation book, questionnaire, etc.), information and consent form, any other relevant document that must be presented to the Committee, etc.).

The trial cannot begin until Inserm has been informed of the unreserved favorable opinion issued by the CPP on the submitted protocol. This opinion will include the title and number of the protocol assigned by the sponsor, the documents examined, as well as the date of its examination and the list of CPP members who participated in it.

The sponsor will inform the CPP of all subsequent amendments and of all serious or unexpected adverse events and developments that arise during the course of the research and that are likely to affect the safety of the individuals involved, in accordance with the sponsor's procedures described in this protocol.

14.4. *Insurance and funding*

Inserm, as promoter, has taken out a civil liability insurance policy for the entire duration of the trial under the number (0100846414031-160021-10998), in accordance with French legal and regulatory provisions on biomedical research.

The Certificate of Insurance for this protocol is attached as annex 11.

Research funding is provided by the EU (H2020-PHC22-2015 program).

14.5. *Procedures for obtaining consent from individuals*

The written informed consent of all persons participating in the research must be obtained by the investigator, registered as a physician and declared as an investigator to the sponsor, prior to any act performed within the framework of the research protocol and regardless of the act performed, in accordance with the regulations.

The information will be given orally and in writing in the first part of the information and consent form (Annex 2). The information will be written in clear language, perfectly understandable for the

person. It must contain all the elements that the person must be informed of, in accordance with the Public Health Code, article L 1122-1.

The person's consent to participate in the research is collected in writing on the second part of the information and consent form. This second part must be written in clear language that is perfectly understandable to the person who is to undergo the research. It must contain all the elements to which the person consents. Consent is given by signing and writing the first name, surname and date in the handwriting of the person who is to undergo the research.

In addition, the investigator who collects the consent form dates and signs the form in the space provided and ensures that (1) the form is accurate and that there are no missing entries or dates, and (2) an original copy of this document is given to the participant. They keep a second original copy in a secure, access-controlled location.

The investigator should ensure that the person undergoing the research has had time to make a free decision and has read and understood the information and consent form.

The information and consent form is a document that will have been approved prior to the implementation of the research by the CPP, during the review of the protocol.

The participation of the persons to the present protocol will be registered on the national file of the persons lending themselves to a biomedical research, managed by the Ministry of Health. Indeed, the non-expert seniors participating in this research will receive an indemnity for the constraints suffered and in compensation for the travels carried out within the framework of this research up to 600€ at the inclusion, 200€ at intermediate, 800€ at the end of the intervention, and finally 500€ (if they accept the optional long-term follow-up) at the end of their participation in this research. The senior experts will receive a compensation of 550€ upon inclusion and will be reimbursed upon presentation of receipts for expenses related to their participation in this trial. This indemnity will be due to them in proportion to their participation in the research. It will be paid in full only if they participate in all the visits, examinations and tests planned by the research.

They are not allowed to participate simultaneously in any other research during a 12-month exclusion period.

For participants in the "passive control" group who wish to do so, it will be proposed to finance a 2-month MBSR (Mindfulness Based Stress Reduction) meditation course (2h/week for 8 weeks + 1 day) or an English course (module: 15h-20h), at the end of the trial. This internship must be completed within 6 months after the end of the interventions.

15. Quality

15.1. *Description*

The research will be supervised according to Inserm's standard operating procedures.

The research in the investigating centers and the care of the participants will be carried out in accordance with the protocol, the Declaration of Helsinki and Good Clinical Practices.

The investigator is above all the guarantor of the quality of the conduct of the trial.

The role of quality assurance is to guarantee the safety of the people who take part in biomedical research and to ensure the credibility of the data resulting from this research and its recognition by the medical and scientific community.

15.2. *Monitoring (quality control of the trial)*

The sponsor's representatives will make visits to the investigating center based on the rate of inclusion and the level of risk that has been assigned to this protocol.

An implementation of the protocol will be carried out by the representatives of the promoter.

Monitoring visits will be conducted by the sponsoring ARC according to current procedures.

At the end of the trial, a closing visit will be made.

At the end of each visit a report will be written by the ARC.

16. Substantial amendments to the protocol

16.1. *Sponsor's procedure for substantial changes*

Any request for substantial modification of the research project in relation to the initially authorized research project must be submitted by the coordinating investigator to the Inserm Pre-selection Unit (CPS) for its opinion.

The terms of submission are available on the website Inserm.fr.

After a favourable opinion from the CPS, Inserm will implement the regulatory administrative procedures necessary to obtain authorisation for these substantial modifications from the CPP and/or the competent administrative authority.

17. Bibliography

Acosta-Cabronero, J., Williams, G.B., Cardenas-Blanco, A., Arnold, R.J., Lupson, V., and Nestor, P.J. (2013). In vivo quantitative susceptibility mapping (QSM) in Alzheimer's disease. PLoS ONE 8, e81093.

Ainsworth, B., Eddershaw, R., Meron, D., Baldwin, D. S., & Garner, M. (2013). The effect of focused attention and open monitoring meditation on attention network function in healthy volunteers. Psychiatry Research, 210(3), 1226-1231.

Albert MS, DeKosky ST, Dickson D, Dubois B, Feldman HH, Fox NC, Gamst A, Holtzman DM, Jagust WJ, Petersen RC, Snyder PJ, Carrillo MC, Thies B, Phelps CH (2011).The diagnosis of mild cognitive impairment due to Alzheimer's disease: recommendations from the National Institute on Aging-Alzheimer's Association workgroups on diagnostic guidelines for Alzheimer's disease. Alzheimers Dement. 7(3):270-9. doi: 10.1016/j.jalz.2011.03.008.

Alzheimer's Association. 2014 Alzheimer's disease facts and figures. Alzheimers Dement. 10, e47–92 (2014).

Babiloni, C., R. Lizio, N. Marzano, P. Capotosto, A. Soricelli, A.I.Triggiani, S. Cordone, L. Gesualdo, and C. Del Percio. (2016). Alzheimer Disease as Revealed by Resting State EEG Rhythms." International Journal of Psychophysiology: 103 (May): 88–102.

Barkhof, F., and Scheltens, P. (2002). Imaging of white matter lesions. Cerebrovasc. Dis. 13 Suppl 2, 21–30.

Baron, J.C., Chételat, G., Desgranges, B., Perchey, G., Landeau, B., de la Sayette, V., and Eustache, F. (2001). In vivo mapping of gray matter loss with voxel-based morphometry in mild Alzheimer's disease. Neuroimage 14, 298–309.

Barua, A., Ghosh, M.K., Kar, N., and Basilio, M.A. (2011). Prevalence of depressive disorders in the elderly. Ann Saudi Med 31, 620–624.

Bastien, C.H., Vallières, A., and Morin, C.M. (2001). Validation of the Insomnia Severity Index as an outcome measure for insomnia research. Sleep Med. 2, 297–307.

Bastin, C., Manrique, A., Scheurich, A., et al. (2013). Relationships between brain metabolism decrease in normal aging and changes in structural and functional connectivity. Neuroimage 76, 167–177.

Beaulieu-Bonneau, S., and Hudon, C. (2009). Sleep disturbances in older adults with mild cognitive impairment. International Psychogeriatrics 21, 654–666.

Berry RB, Brooks R, Gamaldo CE, Harding SM, Lloyd RM, Marcus CL and Vaughn BV (2014) pour l'American Academy of Sleep Medicine. Les règles AASM pour le codage du sommeil et des évènements associés: "The AASM Manual for the Scoring of Sleep and Associated Events: Rules, Terminology and Technical Specifications", Version 2.1. Darien, Illinois: American Academy of Sleep Medicine. Traduction Française par la Société Française de Recherche et Médecine du Sommeil.

Besson, F.L., La Joie, R., Doeuvre, L., Gaubert, M., Mézenge, F., Egret, S., Landeau, B., Barré, L., Abbas, A., Ibazizene, M., et al. (2015a). Cognitive and Brain Profiles Associated with Current Neuroimaging Biomarkers of Preclinical Alzheimer's Disease. J. Neurosci. 35, 10402–10411.

Biedermann, B., P. de Lissa, Y. Mahajan, V. Polito, N. Badcock, M.H. Connors, L.Quinto, L. Larsen, and G. McArthur. (2016) Meditation and auditory attention: An ERP study of meditators and non-meditators. International Journal of Psychophysiology 109: 63–70.

Bonanni, E., Maestri, M., Tognoni, G., Fabbrini, M., Nucciarone, B., Manca, M.L., Gori, S., Iudice, A., and Murri, L. (2005). Daytime sleepiness in mild and moderate Alzheimer's disease and its relationship

with cognitive impairment. J Sleep Res 14, 311–317.

Bourlon, C., Chokron, S., Bachoud-Lévi, A.-C., Coubard, O., Bergeras, I., Moulignier, A., Viret, A.-C., and Bartolomeo, P. (2009). [Presentation of an assessment battery for visual mental imagery and visual perception]. Rev. Neurol. (Paris) 165, 1045–1054.

Braver, T.S., Barch, D.M., Keys, B.A., Carter, C.S., Cohen, J.D., Kaye, J.A., Janowsky, J.S., Taylor, S.F., Yesavage, J.A., Mumenthaler, M.S., et al. (2001). Context processing in older adults: evidence for a theory relating cognitive control to neurobiology in healthy aging. J Exp Psychol Gen 130, 746–763.

Braver, T.S., Satpute, A.B., Rush, B.K., Racine, C.A., and Barch, D.M. (2005). Context processing and context maintenance in healthy aging and early stage dementia of the Alzheimer's type. Psychol Aging 20, 33–46.

Braak H, Braak E. Diagnostic criteria for neuropathologic assessment of Alzheimer's disease. Neurobiol Aging. 1997 Jul-Aug;18(4 Suppl):S85-8.

Brassen, S., Gamer, M. & Büchel, C. Anterior cingulate activation is related to a positivity bias and emotional stability in successful aging. Biol. Psychiatry 70, 131–137 (2011).

Brefczynski-Lewis, J.A., Lutz, A., Schaefer, H.S., Levinson, D.B., and Davidson, R.J. (2007). Neural correlates of attentional expertise in long-term meditation practitioners. Proc. Natl. Acad. Sci. U.S.A. 104, 11483–11488.

Brewer, J.A., Worhunsky, P.D., Gray, J.R., Tang, Y.-Y., Weber, J., and Kober, H. (2011). Meditation experience is associated with differences in default mode network activity and connectivity. Proc. Natl. Acad. Sci. U.S.A. 108, 20254–20259.

Buysse, D.J., Reynolds, C.F., Monk, T.H., Berman, S.R., and Kupfer, D.J. (1989). The Pittsburgh Sleep Quality Index: a new instrument for psychiatric practice and research. Psychiatry Res 28, 193–213.

Caprara, G. V., Steca, P., Zelli, A., & Capanna, C. (2005). A new scale for measuring adults' prosocialness. European Journal of Psychological Assessment, 21(2), 77-89.

Celone, K.A., Calhoun, V.D., Dickerson, B.C., Atri, A., Chua, E.F., Miller, S.L., DePeau, K., Rentz, D.M., Selkoe, D.J., Blacker, D., et al. (2006). Alterations in memory networks in mild cognitive impairment and Alzheimer's disease: an independent component analysis. J. Neurosci. 26, 10222–10231.

Charlson, M.E., Charlson, R.E., Peterson, J.C., Marinopoulos, S.S., Briggs, W.M., Hollenberg, J.P. (2008) The Charlson comorbidity index is adapted to predict costs of chronic disease en primary care patients. J. Clin. Epidemiol. 61, 1234-1240.

Chen, K.W., Berger, C.C., Manheimer, E., Forde, D., Magidson, J., Dachman, L., and Lejuez, C.W. (2012). Meditative therapies for reducing anxiety: a systematic review and meta-analysis of randomized controlled trials. Depress Anxiety 29, 545–562.

Chêne, G., Beiser, A., Au, R., Preis, S.R., Wolf, P.A., Dufouil, C., and Seshadri, S. (2015). Gender and incidence of dementia in the Framingham Heart Study from mid-adult life. Alzheimers Dement 11, 310–320.

Chételat, G., Eustache, F., Viader, F., De La Sayette, V., Pélerin, A., Mézenge, F., Hannequin, D., Dupuy, B., Baron, J.-C., and Desgranges, B. (2005). FDG-PET measurement is more accurate than neuropsychological assessments to predict global cognitive deterioration in patients with mild cognitive impairment. Neurocase 11, 14–25.Chételat, G., Landeau, B., Salmon, E., Yakushev, I., Bahri, M.A., Mézenge, F., Perrotin, A., Bastin, C., Manrique, A., Scheurich, A., et al. (2013). Relationships between brain metabolism decrease in normal aging and changes in structural and functional connectivity. Neuroimage 76, 167–177.

Cherdieu M, Reynaud E, Uhlrich J, Versace R, Mazza S. (2014). Does age worsen sleep-dependent memory consolidation? J Sleep Res., 23(1):53-60. doi: 10.1111/jsr.12100.

Chiesa, A., & Serretti, A. (2009). Mindfulness-based stress reduction for stress management in healthy people: a review and meta-analysis. Journal of Alternative and Complementary Medicine, 15(5), 593-600.

Chiesa, A., Calati, R., and Serretti, A. (2011a). Does mindfulness training improve cognitive abilities? A systematic review of neuropsychological findings. Clin Psychol Rev 31, 449–464.

Choi, S.R., Golding, G., Zhuang, Z., Zhang, W., Lim, N., Hefti, F., Benedum, T.E., Kilbourn, M.R., Skovronsky, D., and Kung, H.F. (2009). Preclinical properties of 18F-AV-45: a PET agent for Abeta plaques in the brain. J. Nucl. Med. 50, 1887–1894.

Chung, F., Yegneswaran, B., Liao, P., Chung, S. A., Vairavanathan, S., Islam, S., … Shapiro, C. M. (2008). STOP Questionnaire. Anesthesiology, 108(5), 812–821. https://doi.org/10.1097/ALN.0b013e31816d83e4

Clark, C.M., Schneider, J.A., Bedell, B.J., Beach, T.G., Bilker, W.B., Mintun, M.A., Pontecorvo, M.J., Hefti, F., Carpenter, A.P., Flitter, M.L., et al. (2011). Use of florbetapir-PET for imaging beta-amyloid pathology. JAMA 305, 275–283.

Costa, P. T., & McCrae, R. R. (1992). Normal personality assessment in clinical practice: The NEO Personality Inventory. Psychological assessment, 4(1), 5.

Craik, F. I. M., & Salthouse, T. A. (Editors.). (2008). The Handbook of Aging and Cognition (3rd Ed. ed.). New York,NY: Psychology Press.

Creswell, J. D., Irwin, M. R., Burklund, L. J., Lieberman, M. D., Arevalo, J. M., Ma, J., Cole, S. W. (2012). Mindfulness-Based Stress Reduction training reduces loneliness and pro inflammatory gene expression in older adults: a small randomized controlled trial. Brain, Behavior, and Immunity, 26(7), 1095-1101.

Damoiseaux, J.S., Rombouts, S. a. R.B., Barkhof, F., Scheltens, P., Stam, C.J., Smith, S.M., and Beckmann, C.F. (2006). Consistent resting-state networks across healthy subjects. Proc. Natl. Acad. Sci. U.S.A. 103, 13848–13853.

Davis, M. H. (1983). Measuring individual differences in empathy: Evidence for a multidimensional approach. Journal of personality and social psychology, 44(1), 113.

Deary, I.J., Corley, J., Gow, A.J., Harris, S.E., Houlihan, L.M., Marioni, R.E., Penke, L., Rafnsson, S.B., and Starr, J.M. (2009). Age-associated cognitive decline. Br. Med. Bull. 92, 135–152.

De Flores, R., La Joie, R., Landeau, B., Perrotin, A., Mézenge, F., de La Sayette, V., Eustache, F., Desgranges, B., and Chételat, G. (2015). Effects of age and Alzheimer's disease on hippocampal subfields: comparison between manual and FreeSurfer volumetry. Hum Brain Mapp 36, 463–474.

Delis, D. C., Kramer, J. H., Kaplan, E., & Ober, B. A. (2000). CVLT-II California Verbal Learning Test Manual Adult Version. San Antonio, Texas: The Psychological Corporation.

De Vibe M, Solhaug I, Tyssen R, Friborg O, Rosenvinge JH, Sørlie T, Bjørndal A. (2013) Mindfulness training for stress management: a randomised controlled study of medical and psychology students. BMC Med Educ, 13:107.

Devilly, G.J., and Borkovec, T.D. (2000). Psychometric properties of the credibility/expectancy questionnaire. J Behav Ther Exp Psychiatry 31, 73–86.

Dickenson, J., Berkman, E. T., Arch, J., & Lieberman, M. D. (2013). Neural correlates of focused attention during a brief mindfulness induction. Soc Cogn Affect Neurosci, 8(1), 40-47.

Diener, E. D., Emmons, R. A., Larsen, R. J., & Griffin, S. (1985). The satisfaction with life scale. Journal of personality assessment, 49(1), 71-75.

Djernes, J.K. (2006). Prevalence and predictors of depression in populations of elderly: a review. Acta Psychiatr Scand 113, 372–387.

Downing, J.E., Miyan, J.A. (2000). Neural immunoregulation: emerging roles for nerves in immune homeostasis and disease. Immunol. Today 21,(6), 281-289.

Eisenlohr-Moul, T. A., Walsh, E. C., Charnigo, R. J., Lynam, D. R., & Baer, R. A. (2012). The "What" and the "How" of dispositional mindfulness: Using interactions among subscales of the Five-Facet Mindfulness Questionnaire to understand its relation to substance use. Assessment, 1073191112446658.

Elias AN, Wilson AF. (1995) Serum hormonal concentrations following transcendental meditation--potential role of gamma aminobutyric acid. Med Hypotheses, 44(4):287-91.

Ellis, B.W., Johns, M.W., Lancaster, R., Raptopoulos, P., Angelopoulos, N., and Priest, R.G. (1981). The St. Mary's Hospital sleep questionnaire: a study of reliability. Sleep 4, 93–97.

Eryilmaz, H., Van De Ville, D., Schwartz, S., & Vuilleumier, P.. (2011). Impact of transient emotions on functional connectivity during subsequent resting state: A wavelet correlation approach. NeuroImage, 54(3), 2481-2491.

Eryilmaz, H., Van De Ville, D., Schwartz, S., & Vuilleumier, P.. (2014). Lasting Impact of Regret and Gratification on Resting Brain Activity and Its Relation to Depressive Traits. The Journal of Neuroscience, 34(23), 7825-7835.

Eustache, F., Giffard, B., Rauchs, G., Chételat, G., Piolino, P., and Desgranges, B. (2006). [Alzheimer's disease and human memory]. Rev. Neurol. (Paris) 162, 929–939.

Folstein, M.F., Folstein, S.E., and McHugh, P.R. (1975). "Mini-mental state". A practical method for grading the cognitive state of patients for the clinician. J Psychiatr Res 12, 189–198.

Ford, G.A., Bryant, C.A., Mangoni, A.A., and Jackson, S.H.D. (2004). Stroke, dementia, and drug delivery. Br J Clin Pharmacol 57, 15–26.

Forman, E. M., Herbert, J. D., Moitra, E., Yeomans, P. D., & Geller, P. A. (2007). A randomized controlled effectiveness trial of acceptance and commitment therapy and cognitive therapy for anxiety and depression. Behavior modification, 31(6), 772-799.

French, D.J., Sargent-Cox, K.A., Kim, S., and Anstey, K.J. (2014). Gender differences in alcohol consumption among middle-aged and older adults in Australia, the United States and Korea. Aust N Z J Public Health 38, 332–339.

Fox, K.C.R., Nijeboer, S., Dixon, M.L., Floman, J.L., Ellamil, M., Rumak, S.P., Sedlmeier, P., and Christoff, K. (2014). Is meditation associated with altered brain structure? A systematic review and meta-analysis of morphometric neuroimaging in meditation practitioners. Neurosci Biobehav Rev 43, 48–73.

Fox, K.C.R., Spreng, R.N., Ellamil, M., Andrews-Hanna, J.R., and Christoff, K. (2015). The wandering brain: meta-analysis of functional neuroimaging studies of mind-wandering and related spontaneous thought processes. Neuroimage 111, 611–621.

Framson, C., Kristal, A. R., Schenk, J. M., Littman, A. J., Zeliadt, S., & Benitez, D. (2009). Development and validation of the mindful eating questionnaire. Journal of the American Dietetic Association, 109(8), 1439-1444.

Gard, T., Taquet, M., Dixit, R., Hölzel, B.K., de Montjoye, Y.-A., Brach, N., Salat, D.H., Dickerson, B.C., Gray, J.R., and Lazar, S.W. (2014). Fluid intelligence and brain functional organization in aging yoga and meditation practitioners. Front Aging Neurosci 6, 76.

Gard, T., Hölzel, B.K., and Lazar, S.W. (2014). The potential effects of meditation on age-related cognitive decline: a systematic review. Ann. N. Y. Acad. Sci. 1307, 89–103.

Gefen, T., Peterson, M., Papastefan, S.T., Martersteck, A., Whitney, K., Rademaker, A., Bigio, E.H., Weintraub, S., Rogalski, E., Mesulam, M.-M., et al. (2015). Morphometric and histologic substrates of cingulate integrity in elders with exceptional memory capacity. J. Neurosci. 35, 1781–1791.

Gillies, G.E., and McArthur, S. (2010). Estrogen actions in the brain and the basis for differential action in men and women: a case for sex-specific medicines. Pharmacol. Rev. 62, 155–198.

Giovacchini, G., Lerner, A., Toczek, M.T., Fraser, C., Ma, K., DeMar, J.C., Herscovitch, P., Eckelman, W.C., Rapoport, S.I., and Carson, R.E. (2004). Brain incorporation of 11C-arachidonic acid, blood volume, and blood flow in healthy aging: a study with partial-volume correction. J. Nucl. Med. 45, 1471–1479.

Godefroy, O. (2008). GREFEX. Fonctions exécutives et pathologies neurologiques et psychiatriques.

Goyal, M., Singh, S., Sibinga, E. M., Gould, N. F., Rowland-Seymour, A., Sharma, R., and Haythornthwaite, J. A. (2014). Meditation programs for psychological stress and well-being: a systematic review and meta-analysis. JAMA Intern Med, 174(3), 357-368.

Greicius, M.D., and Menon, V. (2004). Default-mode activity during a passive sensory task: uncoupled from deactivation but impacting activation. J Cogn Neurosci 16, 1484–1492.

Groot V., Beckerman H., Lankhorst G.J., Bouter L.M. (2003). How to measure comorbidity. a critical review of available methods. J Clin Epidemiol. Mar, 56(3), 221-9.

Gross, J. J., & John, O. P. (2003). Individual differences in two emotion regulation processes: implications for affect, relationships, and well-being. Journal of personality and social psychology, 85(2), 348.

Gur, R.E., and Gur, R.C. (2002). Gender differences in aging: cognition, emotions, and neuroimaging studies. Dialogues Clin Neurosci 4, 197–210.

Hasenkamp, W., Wilson-Mendenhall, C. D., Duncan, E., & Barsalou, L. W. (2012). Mind wandering and attention during focused meditation: a fine-grained temporal analysis of fluctuating cognitive states. Neuroimage, 59(1), 750-760.

Harand C, Bertran F, Doidy F, Guénolé F, Desgranges B, Eustache F, Rauchs G. (2012). How aging affects sleep-dependent memory consolidation. Front Neurol., 3:8. doi: 10.3389/fneur.2012.00008.

Hentschel, F., Damian, M., Krumm, B., and Froelich, L. (2007). White matter lesions - age-adjusted values for cognitively healthy and demented subjects. Acta Neurol. Scand. 115, 174–180.

Herholz, K., and Ebmeier, K. (2011). Clinical amyloid imaging in Alzheimer's disease. Lancet Neurol 10, 667–670.

Hita-Yañez, E., Atienza, M., and Cantero, J.L. (2013). Polysomnographic and subjective sleep markers of mild cognitive impairment. Sleep 36, 1327–1334.

Hofmann, S. G., Sawyer, A. T., Witt, A. A., & Oh, D. (2010). The effect of mindfulness-based therapy on anxiety and depression: A meta-analytic review. Journal of Consulting and Clinical Psychology, 78(2), 169-183.

Hölzel, B.K., Ott, U., Gard, T., Hempel, H., Weygandt, M., Morgen, K., and Vaitl, D. (2008). Investigation of mindfulness meditation practitioners with voxel-based morphometry. Soc Cogn Affect Neurosci 3, 55–61.

Hölzel, B.K., Carmody, J., Vangel, M., Congleton, C., Yerramsetti, S.M., Gard, T., and Lazar, S.W. (2011). Mindfulness practice leads to increases in regional brain gray matter density. Psychiatry Res 191, 36–43.

Horne, J.A., and Ostberg, O. (1976). A self-assessment questionnaire to determine morningness-eveningness in human circadian rhythms. Int J Chronobiol 4, 97–110.

Hot, P., Rauchs, G., Bertran, F., Denise, P., Desgranges, B., Clochon, P., and Eustache, F. (2011). Changes in sleep theta rhythm are related to episodic memory impairment in early Alzheimer's disease. Biol Psychol 87, 334–339.

Hsiao, I.-T., Huang, C.-C., Hsieh, C.-J., Hsu, W.-C., Wey, S.-P., Yen, T.-C., Kung, M.-P., and Lin, K.-J. (2012). Correlation of early-phase 18F-florbetapir (AV-45/Amyvid) PET images to FDG images: preliminary studies. Eur. J. Nucl. Med. Mol. Imaging 39, 613–620.

Hughes, M. E., Waite, L. J., Hawkley, L. C., & Cacioppo, J. T. (2004). A short scale for measuring loneliness in large surveys results from two population-based studies. Research on aging, 26(6), 655-672.

Innes, K.E., and Selfe, T.K. (2014). Meditation as a therapeutic intervention for adults at risk for Alzheimer's disease - potential benefits and underlying mechanisms. Front Psychiatry 5, 40.

Jacobs TL, Epel ES, Lin J, Blackburn EH, Wolkowitz OM, Bridwell DA, Zanesco AP, Aichele SR, Sahdra BK, MacLean KA, King BG, Shaver PR, Rosenberg EL, Ferrer E, Wallace BA, Saron CD. (2011) Intensive meditation training, immune cell telomerase activity, and psychological mediators. Psychoneuroendocrinology, 36(5):664-81.

Jack CR Jr1, Albert MS, Knopman DS, McKhann GM, Sperling RA, Carrillo MC, Thies B, Phelps CH (2011). Introduction to the recommendations from the National Institute on Aging-Alzheimer's Association workgroups on diagnostic guidelines for Alzheimer's disease. Alzheimers Dement.;7(3):257-62. doi: 10.1016/j.jalz.2011.03.004. Epub 2011 Apr 21.

Jané-Llopis E, Gabilondo A. (editors). (2008). Mental Health in Older People. Consensus Paper. Luxembourg: European Communities.

Jansen, W.J., Ossenkoppele, R., Knol, D.L., Tijms, B.M., Scheltens, P., Verhey, F.R.J., Visser, P.J., Amyloid Biomarker Study Group, Aalten, P., Aarsland, D., et al. (2015). Prevalence of cerebral amyloid pathology in persons without dementia: a meta-analysis. JAMA 313, 1924–1938.

Jenkinson, M., Beckmann, C.F., Behrens, T.E.J., Woolrich, M.W., and Smith, S.M. (2012). FSL. Neuroimage 62, 782–790.

Jensen, J.H., and Helpern, J.A. (2010). MRI quantification of non-Gaussian water diffusion by kurtosis analysis. NMR Biomed 23, 698–710.

Jessen, F., Amariglio, R. E., van Boxtel, M., Breteler, M., Ceccaldi, M., Chetelat, G.,

Subjective Cognitive Decline Initiative Working, G. (2014). A conceptual framework for research on subjective cognitive decline in preclinical Alzheimer's disease. Alzheimer's & dementia: the journal of the Alzheimer's Association.

Johns, M.W. (1991). A new method for measuring daytime sleepiness: the Epworth sleepiness scale. Sleep 14, 540–545.

Ju, Y.E.S., Lucey, B.P., and Holtzman, D.M. (2014). Sleep and Alzheimer disease pathology--a bidirectional relationship. Nat Rev Neurol 10, 115–119.

Kaliman P, Alvarez-López MJ, Cosín-Tomás M, Rosenkranz MA, Lutz A, Davidson RJ. (2014) Rapid changes in histone deacetylases and inflammatory gene expression in expert meditators. Psychoneuroendocrinology, 40:96 -107.

Kalpouzos, G., Chételat, G., Baron, J.-C., Landeau, B., Mevel, K., Godeau, C., Barré, L., Constans, J.-M., Viader, F., Eustache, F., et al. (2009). Voxel-based mapping of brain gray matter volume and glucose metabolism profiles in normal aging. Neurobiol. Aging 30, 112–124.

Kang, J.-E., Lim, M.M., Bateman, R.J., Lee, J.J., Smyth, L.P., Cirrito, J.R., Fujiki, N., Nishino, S., and Holtzman, D.M. (2009). Amyloid-beta dynamics are regulated by orexin and the sleep-wake cycle. Science 326, 1005–1007.

Kipling D. Williams , Blair Jarvis (2006). Cyberball: A program for use in research on interpersonal ostracism and acceptance. Behavior Research Methods. 38, 174-180

Klimecki, O. M., Leiberg, S., Lamm, C., & Singer, T. (2013). Functional Neural Plasticity and Associated Changes in Positive Affect After Compassion Training. Cerebral Cortex, 23(7), 1552-1561.

Klimecki, O. M., Leiberg, S., Ricard, M., & Singer, T. (2014). Differential pattern of functional brain plasticity after compassion and empathy training. Social Cognitive and Affective Neuroscience, 9(6), 873-879.

Klimecki, O.M., Mayer, S., Jusyte, A., Scheef, J., & Schönenberg, M. (submitted). Empathy promotes altruistic behaviour in economic interactions.

Kriska, A. M., & Caspersen, C. J. (1997). Introduction to a collection of physical activity questionnaires. Medicine & Science in Sports & Exercise, 29(6), 5-9.

La Joie, R., Fouquet, M., Mézenge, F., Landeau, B., Villain, N., Mevel, K., Pélerin, A., Eustache, F., Desgranges, B., and Chételat, G. (2010). Differential effect of age on hippocampal subfields assessed using a new high-resolution 3T MR sequence. Neuroimage 53, 506–514.

La Joie, R., Perrotin, A., Barré, L., Hommet, C., Mézenge, F., Ibazizene, M., Camus, V., Abbas, A., Landeau, B., Guilloteau, D., et al. (2012). Region-specific hierarchy between atrophy, hypometabolism, and β-amyloid (Aβ) load in Alzheimer's disease dementia. J. Neurosci. 32, 16265–16273.

La Joie, R., Perrotin, A., de La Sayette, V., Egret, S., Doeuvre, L., Belliard, S., Eustache, F., Desgranges, B., and Chételat, G. (2013). Hippocampal subfield volumetry in mild cognitive impairment, Alzheimer's disease and semantic dementia. Neuroimage Clin 3, 155–162.

Landau, S.M., Harvey, D., Madison, C.M., Reiman, E.M., Foster, N.L., Aisen, P.S., Petersen, R.C., Shaw, L.M., Trojanowski, J.Q., Jack, C.R., et al. (2010). Comparing predictors of conversion and decline in mild cognitive impairment. Neurology 75, 230–238.

Larrieu, S., Letenneur, L., Berr, C., Dartigues, J.F., Ritchie, K., Alperovitch, A., Tavernier, B., and Barberger-Gateau, P. (2004). Sociodemographic differences in dietary habits in a population-based

sample of elderly subjects: the 3C study. J Nutr Health Aging 8, 497–502.

Lazar, S.W., Kerr, C.E., Wasserman, R.H., Gray, J.R., Greve, D.N., Treadway, M.T., McGarvey, M., Quinn, B.T., Dusek, J.A., Benson, H., et al. (2005). Meditation experience is associated with increased cortical thickness. Neuroreport 16, 1893–1897.

Lenze, E.J., Hickman, S., Hershey, T., Wendleton, L., Ly, K., Dixon, D., Doré, P., and Wetherell, J.L. (2014). Mindfulness-based stress reduction for older adults with worry symptoms and co-occurring cognitive dysfunction. Int J Geriatr Psychiatry 29, 991–1000.

Lin, K.-J., Hsu, W.-C., Hsiao, I.-T., Wey, S.-P., Jin, L.-W., Skovronsky, D., Wai, Y.-Y., Chang, H.-P., Lo, C.-W., Yao, C.H., et al. (2010). Whole-body biodistribution and brain PET imaging with [18F]AV-45, a novel amyloid imaging agent — a pilot study. Nuclear Medicine and Biology 37, 497–508.

Luders, E., Thompson, P.M., Kurth, F., Hong, J.-Y., Phillips, O.R., Wang, Y., Gutman, B.A., Chou, Y.-Y., Narr, K.L., and Toga, A.W. (2013). Global and regional alterations of hippocampal anatomy in long-term meditation practitioners. Hum Brain Mapp 34, 3369–3375.

Lu T, Aron L, Zullo J, Pan Y, Kim H, Chen Y, Yang TH, Kim HM, Drake D, Liu XS, Bennett DA, Colaiácovo MP1, Yankner BA1. (2014) REST and stress resistance in ageing and Alzheimer's disease. Nature. 2014 Mar 27;507(7493):448-54.

Lustig, C., Snyder, A.Z., Bhakta, M., O'Brien, K.C., McAvoy, M., Raichle, M.E., Morris, J.C., and Buckner, R.L. (2003). Functional deactivations: change with age and dementia of the Alzheimer type. Proc. Natl. Acad. Sci. U.S.A. 100, 14504–14509.

Lutz, A., Slagter, H.A., Dunne, J.D., and Davidson, R.J. (2008). Attention regulation and monitoring in meditation. Trends Cogn. Sci. (Regul. Ed.) 12, 163–169.

Lutz A, McFarlin DR, Perlman DM, Salomons TV, Davidson RJ. Altered anterior insula activation during anticipation and experience of painful stimuli in expert meditators. Neuroimage. 2013 Jan 1;64:538-46.

Landau, S. M., Harvey, D., Madison, C. M., Reiman, E. M., Foster, N. L., Aisen, P. S., Jagust, W. J. (2010). Comparing predictors of conversion and decline in mild cognitive impairment. Neurology, 75(3), 230-238.

Marciniak, R., Sheardova, K., Cermáková, P., Hudeček, D., Sumec, R., and Hort, J. (2014). Effect of meditation on cognitive functions in context of aging and neurodegenerative diseases. Front Behav Neurosci 8, 17.

Mårtensson, J. et al. Growth of language-related brain areas after foreign language learning. NeuroImage 63, 240–244 (2012).

Mattis S. (1976). Mental status examination for organic mental syndrome in the elderly patient. Geriatric psychiatry: a handbook for psychiatrists and primary care physicians. 77-121.

McKee, K. J., Philp, I., Lamura, G., Prouskas, C., Öberg, B., Krevers, B., ... & Szczerbinska, K. (2003). The COPE index--a first stage assessment of negative impact, positive value and quality of support of caregiving in informal carers of older people. Aging & Mental Health, 7(1), 39-52.

Mehling, W. E., Price, C., Daubenmier, J. J., Acree, M., Bartmess, E., & Stewart, A. (2012). The multidimensional assessment of interoceptive awareness (MAIA). PLoS One, 7(11), e48230.Menon, V. & Uddin, L. Q. Saliency, switching, attention and control: a network model of insula function. Brain Struct. Funct. 214, 655–667 (2010).

Mevel, K., Grassiot, B., Chételat, G., Defer, G., Desgranges, B., and Eustache, F. (2010). [The default mode network: cognitive role and pathological disturbances]. Rev. Neurol. (Paris) 166, 859–872.

Meyer, T. J., Miller, M. L., Metzger, R. L., & Borkovec, T. D. (1990). Development and validation of the Penn State worry questionnaire. Behaviour research and therapy, 28(6), 487-495.

Mohammadi, S., Möller, H.E., Kugel, H., Müller, D.K., and Deppe, M. (2010). Correcting eddy current and motion effects by affine whole-brain registrations: evaluation of three-dimensional distortions and comparison with slicewise correction. Magn Reson Med 64, 1047–1056.

Montgomery, S.A., and Asberg, M. (1979). A new depression scale designed to be sensitive to change. Br J Psychiatry 134, 382–389.

Mulligan, R.S., Villemagne, V.L., Akatsu, H., Yamamoto, T., et al. (2013). Novel 18F-Labeled Arylquinoline Derivatives for Noninvasive Imaging of Tau Pathology in Alzheimer Disease. J Nucl Med 54, 1420–1427.

Neff, K. D. (2003). The development and validation of a scale to measure self-compassion. Self and identity, 2(3), 223-250.

Newberg AB, Iversen J. (2003) The neural basis of the complex mental task of meditation: neurotransmitter and neurochemical considerations. Med Hypotheses, 61(2):282-91.

Newberg, A.B., Wintering, N., Waldman, M.R., Amen, D., Khalsa, D.S., and Alavi, A. (2010). Cerebral blood flow differences between long-term meditators and non-meditators. Conscious Cogn 19, 899–905.

Newberg, A.B., Serruya, M., Wintering, N., Moss, A.S., Reibel, D., and Monti, D.A. (2014). Meditation and neurodegenerative diseases. Ann. N. Y. Acad. Sci. 1307, 112–123.

Norton, S., Matthews, F.E., Barnes, D.E., Yaffe, K., and Brayne, C. (2014). Potential for primary prevention of Alzheimer's disease: an analysis of population-based data. Lancet Neurol 13, 788–794.

Ohayon, M.M., and Vecchierini, M.-F. (2005). Normative sleep data, cognitive function and daily living activities in older adults in the community. Sleep 28, 981–989.

Oldfield RC (1971). The assessment and analysis of handedness: the edinburgh inventory. Neuropsychologia. 9(1):97-113.

Ooms, S., Overeem, S., Besse, K., Rikkert, M.O., Verbeek, M., and Claassen, J.A.H.R. (2014). Effect of 1 night of total sleep deprivation on cerebrospinal fluid β-amyloid 42 in healthy middle-aged men: a randomized clinical trial. JAMA Neurol 71, 971–977.

Pace, T.W.W., Negi, L.T., Adame, D.D., Cole, S.P., Sivilli, T.I., Brown, T.D., Issa, M.J., and Raison, C.L. (2009). Effect of compassion meditation on neuroendocrine, innate immune and behavioural responses to psychosocial stress. Psychoneuroendocrinology 34, 87–98.

Pace TW, Negi LT, Sivilli TI, Issa MJ, Cole SP, Adame DD, Raison CL. (2010) Innate immune, neuroendocrine and behavioural responses to psychosocial stress do not predict subsequent compassion meditation practice time. Psychoneuroendocrinology, 35(2):310-5.

Paquette, D., Laporte, L., Bigras, M., & Zoccolillo, M. (2004). Validation de la version française du CTQ et prévalence de l'histoire de maltraitance. Santé Mentale Au Québec, 29(1), 201.

Parrott, A.C., and Hindmarch, I. (1980). The Leeds Sleep Evaluation Questionnaire in psychopharmacological investigations - a review. Psychopharmacology (Berl.) 71, 173–179.

Paxton, J.L., Barch, D.M., Racine, C.A., and Braver, T.S. (2008). Cognitive control, goal maintenance, and prefrontal function in healthy aging. Cereb. Cortex 18, 1010–1028.

Piolino, P., Desgranges, B., Benali, K., and Eustache, F. (2002). Episodic and semantic remote autobiographical memory in ageing. Memory 10, 239–257.

Piolino, P., Desgranges, B., Belliard, S., Matuszewski, V., Lalevée, C., De la Sayette, V., and Eustache, F. (2003). Autobiographical memory and autonoetic consciousness: triple dissociation in neurodegenerative diseases. Brain 126, 2203–2219.

Prakash, R., Dubey, I., Abhishek, P., Gupta, S. K., Rastogi, P., & Siddiqui, S. V. (2010). Long-term Vihangam Yoga meditation and scores on tests of attention. Perceptual and Motor Skills, 110(3 Pt 2), 1139-1148.

Prakash, R., Rastogi, P., Dubey, I., Abhishek, P., Chaudhury, S., & Small, B. J. (2012). Long-term concentrative meditation and cognitive performance among older adults. Neuropsychol Dev Cogn B Aging Neuropsychol Cogn, 19(4), 479-494.

Raes, F., Pommier, E., Neff, K. D., & Van Gucht, D. (2011). Construction and factorial validation of a short form of the self-compassion scale. Clinical psychology & psychotherapy, 18(3), 250-255.

Rasch B, Born J.(2013). About sleep's role in memory. Physiol Rev., 93(2):681-766. doi: 10.1152/physrev.00032.2012.

Rasch B, Büchel C, Gais S, Born J. (2007). Odor cues during slow-wave sleep prompt declarative memory consolidation. Science, 315(5817):1426-9.

Rauchs, G., Schabus, M., Parapatics, S., Bertran, F., Clochon, P., Hot, P., Denise, P., Desgranges, B., Eustache, F., Gruber, G., et al. (2008). Is there a link between sleep changes and memory in Alzheimer's disease? Neuroreport 19, 1159–1162.

Rauchs G, Desgranges B, Foret J, Eustache F. (2005). The relationships between memory systems and sleep stages. J Sleep Res., 14(2):123-40.

Reiman EM, Caselli RJ, Yun LS, Chen K, Bandy D, Minoshima S, et al. (1996). Preclinical evidence of Alzheimer's disease in persons homozygous for the epsilon 4 allele for apolipoprotein E. N Engl J Med 334(12):752–8.

Ricci, S., Fuso, A., Ippoliti, F., and Businaro, R. (2012). Stress-induced cytokines and neuronal dysfunction in Alzheimer's disease. J. Alzheimers Dis. 28, 11–24.

Rocca, W.A., Mielke, M.M., Vemuri, P., and Miller, V.M. (2014). Sex and gender differences in the causes of dementia: a narrative review. Maturitas 79, 196–201.

Roulet, L., Asseray, N., Foucher, N., Potel, G., Lapeyre-Mestre, M., & Ballereau, F. (2013). A questionnaire to document self-medication history in adult patients visiting emergency departments. Pharmacoepidemiology and drug safety, 22(2), 151-159.

Rosano, C., Aizenstein, H.J., Newman, A.B., Venkatraman, V., Harris, T., Ding, J.,Satterfield, S., Yaffe, K., and Health ABC Study (2012). Neuroimaging differences between older adults with maintained versus declining cognition over a 10-year period. Neuroimage 62, 307–313.

Rosenkranz, M.A., Lutz, A., Perlman, D.M., Bachhuber, D.R.W., Schuyler, B.S., MacCoon, D.G., and Davidson, R.J. (2016). Reduced stress and inflammatory responsiveness in experienced meditators compared to a matched healthy control group. Psychoneuroendocrinology 68, 117–125.

Rosenkranz MA, Lutz A, Perlman DM, Bachhuber DR, Schuyler BS, MacCoon DG, Davidson RJ. Reduced stress and inflammatory responsiveness in experienced meditators compared to a matched healthy control group. Psychoneuroendocrinology. 2016 Feb 20;68:117-125.

Ruzzoli, Manuela, Cornelia Pirulli, Veronica Mazza, Carlo Miniussi, and Debora Brignani. 2016. nThe Mismatch Negativity as an Index of Cognitive Decline for the Early Detection of Alzheimer's Disease." Scientific Reports 6 (September): 33167. doi:10.1038/srep33167.

Ryff, C. D., & Keyes, C. L. M. (1995). The structure of psychological well-being revisited. Journal of personality and social psychology, 69(4), 719.

Salmon, D.P., and Bondi, M.W. (2009). Neuropsychological assessment of dementia. Annu Rev Psychol 60, 257–282.

Samaille, T., Fillon, L., Cuingnet, R., Jouvent, E., Chabriat, H., Dormont, D., Colliot, O., and Chupin, M. (2012). Contrast-based fully automatic segmentation of white matter hyperintensities: method and validation. PLoS ONE 7, e48953.

Scheef L, Spottke A, Daerr M, Joe A, Striepens N, Kölsch H, Popp J, Daamen M, Gorris D, Heneka MT, Boecker H, Biersack HJ, Maier W, Schild HH, Wagner M, Jessen F. (2012). Glucose metabolism, gray matter structure, and memory decline in subjective memory impairment. Neurology. 25;79(13):1332-9.

Sheikh, J. A., & Yesavage, J. A. (1986). Geriatric Depression Scale (GDS): Recent findings and development of a shorter version In: Brink TL, Ed. Clinical gerontology: a guide to assessment and intervention.

Sherbourne, C. D., & Stewart, A. L. (1991). The MOS social support survey. Social science & medicine, 32(6), 705-714.

Schneider, R.H., Grim, C.E., Rainforth, M.V., Kotchen, T., Nidich, S.I., Gaylord-King, C., Salerno, J.W., Kotchen, J.M., and Alexander, C.N. (2012). Stress reduction in the secondary prevention of cardiovascular disease: randomized, controlled trial of transcendental meditation and health education in Blacks. Circ Cardiovasc Qual Outcomes 5, 750–758.

Schutte, N.S., and Malouff, J.M. (2014). A meta-analytic review of the effects of mindfulness meditation on telomerase activity. Psychoneuroendocrinology 42, 45–48.Schröder, H., Fitó, M., Estruch, R., Martínez-González, M. A., Corella, D., Salas-Salvadó, J., ... & Lapetra, J. (2011). A short screener is valid for assessing Mediterranean diet adherence among older Spanish men and women. The Journal of nutrition, 141(6), 1140-1145.

Shepard, R.N., and Metzler, J. (1971). Mental rotation of three-dimensional objects. Science 171, 701–703.

Slagter, H.A., Davidson, R.J., and Lutz, A. (2011). Mental training as a tool in the neuroscientific study of brain and cognitive plasticity. Front Hum Neurosci 5, 17.

Smith AK, Conneely KN, Kilaru V, Mercer KB, Weiss TE, Bradley B, et al. Differential immune system DNA methylation and cytokine regulation in post-traumatic stress disorder. Am J Med Genet B Neuropsychiatr Genet. 2011;156B:700–8.

Sperling RA1, Aisen PS, Beckett LA, Bennett DA, Craft S, Fagan AM, Iwatsubo T, Jack CR Jr, Kaye J, Montine TJ, Park DC, Reiman EM, Rowe CC, Siemers E, Stern Y, Yaffe K, Carrillo MC, Thies B, Morrison-Bogorad M, Wagster MV, Phelps CH. (2011) Toward defining the preclinical stages of Alzheimer's disease: recommendations from the National Institute on Aging-Alzheimer's Association workgroups on diagnostic guidelines for Alzheimer's disease. Alzheimers Dement. 7(3):280-92. doi: 10.1016/j.jalz.2011.03.003.

Spielberger, C. D. (1983). Manual for the State-Trait Anxiety Inventory STAI (form Y)(" self-evaluation questionnaire").

Spira, A.P., Gamaldo, A.A., An, Y., Wu, M.N., Simonsick, E.M., Bilgel, M., Zhou, Y., Wong, D.F., Ferrucci, L., and Resnick, S.M. (2013). Self-reported sleep and β-amyloid deposition in community-dwelling older adults. JAMA Neurol 70, 1537–1543.

Splevins, K., Smith, A., & Simpson, J. (2009). Do improvements in emotional distress correlate with becoming more mindful? A study of older adults. Aging Ment Health, 13(3), 328-335.

Tabesh, A., Jensen, J.H., Ardekani, B.A., and Helpern, J.A. (2011). Estimation of tensors and tensor-derived measurements in diffusional kurtosis imaging. Magn Reson Med 65, 823–836.

Tago, T., Furumoto, S., Okamura, N., Harada, R., Adachi, H., Ishikawa, Y., Yanai, K., Iwata, R., and Kudo, Y. (2016). Structure-Activity Relationship of 2-Arylquinolines as PET Imaging Tracers for Tau Pathology in Alzheimer Disease. J. Nucl. Med. 57, 608–614.

Tang, Y.-Y., Lu, Q., Feng, H., Tang, R., and Posner, M.I. (2015). Short-term meditation increases blood flow in anterior cingulate cortex and insula. Front Psychol 6, 212.

Teipel, S., Drzezga, A., Grothe, M.J., Barthel, H., Chételat, G., Schuff, N., Skudlarski, P., Cavedo, E., Frisoni, G.B., Hoffmann, W., et al. (2015). Multimodal imaging in Alzheimer's disease: validity and usefulness for early detection. Lancet Neurol 14, 1037–1053.

Templer, D. I., Harville, M., Hutton, S., Underwood, R., Tomeo, M., Russell, M., ... & Arikawa, H. (2002). Death depression scale-revised. OMEGA-Journal of Death and Dying, 44(2), 105-112.

Teper, R., & Inzlicht, M. (2013). Meditation, mindfulness and executive control: the importance of emotional acceptance and brain-based performance monitoring. Soc Cogn Affect Neurosci, 8(1), 85-92.

Tomaszewski Farias, S., Mungas, D., Harvey, D.J., Simmons, A., Reed, B.R., and Decarli, C. (2011). The measurement of everyday cognition: development and validation of a short form of the Everyday Cognition scales. Alzheimers Dement 7, 593–601.

Treynor, W., Gonzalez, R., & Nolen-Hoeksema, S. (2003). Rumination reconsidered: A psychometric analysis. Cognitive therapy and research, 27(3), 247-259.

Tzourio-Mazoyer, N., Landeau, B., Papathanassiou, D., Crivello, F., Etard, O., Delcroix, N., Mazoyer, B., and Joliot, M. (2002). Automated anatomical labeling of activations in SPM using a macroscopic anatomical parcellation of the MNI MRI single-subject brain. Neuroimage 15, 273–289.

Valenzuela, M. J., & Sachdev, P. (2007). Assessment of complex mental activity across the lifespan: development of the Lifetime of Experiences Questionnaire (LEQ). Psychological medicine, 37(07), 1015-1025.

Van der Linden, M., and Juillerat, A.-C. (2004a). [Neuropsychological rehabilitation in early stage Alzheimer's disease: principles, methods and perspectives]. Rev. Neurol. (Paris) 160, S64-70.

Van der Linden, M., and Juillerat, A.-C. (2004b). [Neuropsychological rehabilitation in early stage Alzheimer's disease: principles, methods and perspectives]. Rev. Neurol. (Paris) 160, S64-70.

Villain, N., Landeau, B., Groussard, M., Mevel, K., Fouquet, M., Dayan, J., Eustache, F., Desgranges, B., and Chételat, G. (2010). A simple way to improve anatomical mapping of functional brain imaging. J Neuroimaging 20, 324–333.

Villemagne, V.L., and Chételat, G. (2016). Neuroimaging biomarkers in Alzheimer's disease and other dementias. Ageing Res. Rev.

Villemagne, V.L., Fodero-Tavoletti, M.T., Pike, K.E., Cappai, R., Masters, C.L., and Rowe, C.C. (2008). The ART of loss: Abeta imaging in the evaluation of Alzheimer's disease and other dementias. Mol. Neurobiol. 38, 1–15.Villemagne, V.L., Pike, K.E., Chételat, G., Ellis, K.A., Mulligan, R.S., Bourgeat, P., Ackermann, U., Jones, G., Szoeke, C., Salvado, O., et al. (2011). Longitudinal assessment of Aβ and cognition in aging and Alzheimer disease. Ann. Neurol. 69, 181–192.

Vuillemin, A., Oppert, J. M., Guillemin, F., Essermeant, L., Fontvieille, A. M., Galan, P. I. L. A. R., ... & Hercberg, S. E. R. G. E. (2000). Self-administered questionnaire compared with interview to assess past-year physical activity. Medicine and Science in Sports and Exercise, 32(6), 1119-1124.

Washburn, R. A., Smith, K. W., Jette, A. M., & Janney, C. A. (1993). The Physical Activity Scale for the Elderly (PASE): development and evaluation. Journal of clinical epidemiology, 46(2), 153-162.

Watson, D., Clark, L. A., & Tellegen, A. (1988). Development and validation of brief measurements of positive and negative affect: the PANAS scales. Journal of personality and social psychology, 54(6), 1063.

Wenk-Sormaz, H. (2005). Meditation can reduce habitual responding. Alternative Therapies in Health and Medicine, 11(2), 42-58.

Williams, K.D., Cheung, C.K., and Choi, W. (2000). Cyberostracism: effects of being ignored over the Internet. J Pers Soc Psychol 79, 748–762.

Wenk-Sormaz, H. (2005). Meditation can reduce habitual responding. Altern Ther Health Med 11, 42–58.

Westerberg, C.E., Mander, B.A., Florczak, S.M., Weintraub, S., Mesulam, M.-M., Zee, P.C., and Paller, K.A. (2012). Concurrent impairments in sleep and memory in amnestic mild cognitive impairment. J Int Neuropsychol Soc 18, 490–500.

Wilson, R.S., Barnes, L.L., Mendes de Leon, C.F., Aggarwal, N.T., Schneider, J.S., Bach, J., Pilat, J., Beckett, L.A., Arnold, S.E., Evans, D.A., et al. (2002). Depressive symptoms, cognitive decline, and risk of AD in older persons. Neurology 59, 364–370.

Wilson, R. S., Barnes, L. L., & Bennett, D. A. (2003). Assessment of lifetime participation in cognitively stimulating activities. Journal of Clinical and Experimental Neuropsychology, 25(5), 634-642.

Winbush, N.Y., Gross, C.R., and Kreitzer, M.J. (2007a). The effects of mindfulness-based stress reduction on sleep disturbance: a systematic review. Explore (NY) 3, 585–591.

Winbush, N.Y., Gross, C.R., and Kreitzer, M.J. (2007b). The effects of mindfulness-based stress reduction on sleep disturbance: a systematic review. Explore (NY) 3, 585–591.

Wong, D.F., Rosenberg, P.B., Zhou, Y., Kumar, A., Raymont, V., Ravert, H.T., Dannals, R.F., Nandi, A., Brasić, J.R., Ye, W., et al. (2010). In vivo imaging of amyloid deposition in Alzheimer disease using

the radioligand 18F-AV-45 (florbetapir [corrected] F 18). J. Nucl. Med. 51, 913–920.

World Health Organization. (1996). What quality of life? The WHOQOL Group. World Health Organization Quality of Life Assessment. In World Health Forum (Vol. 17, No. 4, pp. 354-356).

Yu X, Fumoto M, Nakatani Y, Sekiyama T, Kikuchi H, Seki Y, Sato-Suzuki I, Arita H. (2011) Activation of the anterior prefrontal cortex and serotonergic system is associated with improvements in mood and EEG changes induced by Zen meditation practice in novices. Int J Psychophysiol., 80(2):103-11.

Yushkevich, P.A., Pluta, J.B., Wang, H., Xie, L., Ding, S.-L., Gertje, E.C., Mancuso, L., Kliot, D., Das, S.R., and Wolk, D.A. (2015). Automated volumetry and regional thickness analysis of hippocampal subfields and medial temporal cortical structures in mild cognitive impairment. Hum Brain Mapp 36, 258–287.

Zellner Keller, B., Singh, N.N., and Winton, A.S.W. (2014). Mindfulness-Based Cognitive Approach for Seniors (MBCAS): Program Development and Implementation. Mindfulness (N Y) 5, 453–459.

Zhou, W., Zhang, J., Ye, F., Xu, G., Su, H., Su, Y., … Alzheimer's Disease Neuroimaging Initiative. (2017). Plasma neurofilament light chain levels in Alzheimer's disease. Neuroscience Letters, 650, 60–64. https://doi.org/10.1016/j.neulet.2017.04.027

ANNEXES

17.1. Protocol annex: Age-Well Lockdown substudy entitled "Impact of Lockdown in the Age-Well Cohort"

Protocol annex: Age-Well Lockdown substudy entitled "Impact of Lockdown in the Age-Well Cohort"

Title: Impact of Lockdown in the Age-Well cohort

The objective of the experiment is to measure the psychological consequences related to the lockdown and other health measures related to the COVID-19 epidemic in an older adults' population.

1. SUMMARY DESCRIPTION OF THE PROJECT

Scientific background and interest

The coronavirus crisis is forcing us to remain confined to our homes for medical and health reasons. Nevertheless, previous studies have shown negative effects of confinement (recent review in Brooks et al, 2020). And isolation is known to be a risk factor for many psychiatric conditions (Michalska da Rocha et al, 2018; Narita et al, 2020). With the prolongation of the crisis, strict containment measures have evolved into other forms of measures that promote the isolation of populations, especially among the older adults (distancing measures, curfews, etc.). Beyond the containment and distancing measures, the current situation presents a major source of stress and anxiety, all the more worrying as it persists. The Age-Well protocol aims to develop and test approaches to maintain the quality of life of seniors, to preserve their physical and mental conditions and to prevent cognitive decline, dementia, sleep disorders and depression, which increase with age. This project is based on the postulate that mental training in the regulation of stress and emotions through meditation would reduce these effects and thus improve the well-being and mental health of seniors. In this context of deteriorating living conditions, it seems relevant to evaluate how the Age-Well population is affected and reacts to this health situation, and the impact of the mental training tested in the Age-Well clinical trial on the quality of life of seniors during a pandemic.

A first confinement started on March 17, 2020 in France, and has also started in the USA, where a collaborator has obtained approval for a study on the psychological effects of confinement (Sohee Park in Nashville). We propose to have the participants recruited in the Age-Well trial fill in the questionnaires used in the USA, adapted and translated into French, as soon as we receive the signed consent of the participant (Lockdown Questionnaire n°1), then one month (Lockdown Questionnaire n°2) and 6 months later. This Lockdown Questionnaire n°3, in view of the persistence of the crisis, was proposed during the second national lockdown (announced on October 30, 2020). Three mailings of these questionnaires (Lockdown Questionnaires n°4, 5 and 6) will be proposed again during the different long-term evolution phases of the pandemic (including an evaluation in spring 2021, i.e. ~1 year after the beginning of the pandemic and during the vaccination campaign, an evaluation in the fall of 2021, when the vaccination campaign should be sufficiently underway to signal a gradual return to normalcy, and an evaluation in the spring of 2022, ~2 years after the onset of the pandemic which - ideally - would correspond to a return to normal). The questionnaires will make it possible to evaluate how individual factors (illness, traumatic history, conditions of confinement) modulate the quality of life of seniors during confinement and how the mental training followed modulates this quality of life during confinement.

The funding of this sub-study is ensured by the MMA financing of Lyon and Caen, as well as the financing of the Normandy Label of Excellence Region, which cover the total duration of this sub-study.

Objectives and judging criteria

Main objective:

- Quantify the psychological effects of the health situation with questionnaires.

Secondary objectives:

- To evaluate the effects of mental training on the experience of this health crisis.
- To evaluate the effects of meditation expertise on the experience of this health crisis.

Primary and secondary judgement criteria:

- Behavioral measures: a composite score per function assessed (anxiety, perceived stress, self-compassion...) by the questionnaires.

General considered methodology

Our approach is quantitative.

General hypotheses
Main objective hypothesis:
We expect to observe changes in well-being/unwell-being and quality of life over time, which will depend on the subjects' isolation. Finally, we expect that the conditions of confinement and the medical history, the individual factors measured in the questionnaires, will aggravate the psychological effects linked to the health context.

Secondary objectives hypotheses:
- We expect the seniors who received the meditation intervention to cope better than the other two groups of seniors (i.e., those who received the English learning intervention and those in the control group who did not change their lifestyle habits).
- We expect the meditating experts to be only slightly affected by this period of crisis or to a lesser extent than the seniors in the control group.

2. MATERIEL ET METHODES

A. Participants

Number of participants:
We will offer these questionnaires to the 135 seniors and 25 experts included in the Age-Well clinical trial on April 8th, 2020.

Recruitment of participants:
Recruitment method: Volunteers are already included in Age-Well. The information leaflet and the consent form will be sent by mail to the participant who will return it by mail to the coordinating investigator using a pre-stamped return envelope provided by the investigator (To the attention of Dr Vincent de la Sayette, Silver Santé Study, GIP CYCERON Boulevard Henri Becquerel - BP 5229 14074 CAEN cedex 5; original signature) and will inform the investigator in parallel by email. The consents will then be kept in the participants' source files. For participants who do not have an e-mail address, we will send them the information notice and the consent form by mail.
The consent annex for the sending of the Lockdown questionnaires n°4, 5 and 6 will only be proposed to the Age-Well participants having signed the initial consent for the Age-Well Lockdown sub-study. The rest of the procedure is identical to the one we have set up for the first part of this sub-study.

Possible compensation of participants:
No compensation is provided.

B. Method: data collection, management and analysis

Description of the protocol
All questionnaires will be sent via a secure "LimeSurvey" link by email. They will be completed by the subjects upon receipt of the signed consent, and then 1 month and 6 months after the end of the first lockdown period. They will be offered again during the different phases of the long-term evolution of the pandemic, including an evaluation in spring 2021 (after ~1 year of pandemic), an evaluation in fall 2021 and an evaluation in spring 2022 (which - ideally - would correspond to a return to normal).
On the LimeSurvey homepage, we will include the information found in the information leaflet.
For participants who do not have an email address, the investigator will send the questionnaires by mail and by return post. We will provide return envelopes to facilitate this step.

Note that before the start of this Age-Well - Lockdown sub-study, the questionnaires used in the USA, adapted and translated into French via Lime Survey will be reported to the Data Protection Officer at cil@unicaen.fr by the coordinating investigator and the scientific leader of the Age-Well project (Inserm n°: C16-38). The Data Protection Officer has a permanent eye on all the questionnaires in order to verify that they comply with the European regulation 2016/679 (known as RGPD: General Data Protection Regulation) and with the law 78-17 of January 6, 1978 modified on August 6, 2004 (known as « Loi informatique et Libertés »).

Location(s) where the study will be conducted:

The subjects will be able to participate from home, and the results can be analyzed at the end of the lockdown at the laboratory INSERM U1237.

Schedule of assessments, observations or interviews:

- expected date for the start of the research: April 10th, 2020
- date scheduled for the end of the research: April 10th, 2022

The duration of the participation of each subject will be 2 hours for the questionnaires, 6 times.

Total duration of the study: 24 months.

Data analysis:
The statistical analysis of the data will focus on the scores of the questionnaires.
Compatibility with a Gaussian distribution will be tested for all quantitative variables with the Shapiro test. If needed for further analysis, the variables will be transformed with the Box-Cox method to approximate as closely as possible a Gaussian distribution.

The analyses will be performed with R and Statistica software in its most up-to-date version at the time of the analysis.

<u>Benefits of the study</u>
This study has no direct benefit to the subjects who will participate. It will allow a better understanding of the psychological effects of the confinement and the health situation.

<u>Risks of the study</u>

- There are no foreseeable risks to subjects in this study. No foreseeable adverse events are expected in this research.
- In a more general way, we put at their disposal an email address which will allow them to contact us for a care in case of psychological problems.
- - We restrict questions about privacy to those necessary to interpret the effects of lockdown. Since we expect a large number of responses, we believe that anonymity will be properly respected. We add in the information that if any questions bother the subjects they can refrain from answering.

C. Vigilance/ Premature study termination

<u>Study termination criteria</u> for a subject who participates in it

At any time a participant can leave the trial:
- on request;
- or according to the decision of the responsible researcher.

In all cases, the reasons for exclusion from the trial (either at the participant's request or at the researcher's decision) should be recorded in the observation book created for each subject. If it is the researcher's decision, the researcher will specify the extent to which the subject was bothered by the experimental approach.

3. DATA TREATMENT – Privacy and security

Privacy
In accordance with article R.5121-13 of the French Public Health Code, investigators and all persons called upon to collaborate in the trial are bound by professional secrecy concerning, in particular, the persons involved and the results obtained. The principal investigator must ensure that the anonymity of patients is respected.

Anonymization / confidentiality process
The questionnaires will not be sent via the "invitation" functionality in Limesurvey. Indeed, this functionality allows to enter the email addresses of the participants to the study, but also allows to know who has answered or not. As a result, the "anonymous" character of the answers would no longer be guaranteed. The mailings will be done by email via a unique link to the Limesurvey questionnaire.
Age-Well subjects will use their anonymization number to identify the questionnaires they will send via Limesurvey. A separate file will be created by the investigator where they will collect the consents sent by email. The data will be analyzed in a pseudo-anonymized way. Only the members of the research team involved in the analyses will have access to the answers in Limesurvey.
Aucune donnée publiée ne pourra permettre d'identifier les participants à cette étude; seule la moyenne et l'écart-type de l'âge, et le ratio femmes/hommes sur les groupes seront fournis dans les données publiées.

Archiving

Type of archived data (specify whether the data allows identification, directly or by cross-checking):
The archived data will be the research documents (protocol, signed informed consents) and the measurements performed in this research (performance measurements). The data will not allow identification of subjects. Consents will be archived separately from the protocol data, to which they cannot be linked.
Exports of responses from Limesurvey will be stored in a dedicated file, different from the one used to store consents. Once compiled in an Excel table, the scores will be sent in a secure way to the data management structure so that it can be linked to the general Age-Well database.

Duration of the archive:
The duration of the archive will be 15 years from the end of the study.

Location of the archive:
At the end of the research, the paper documents will be stored in the archives of the research site (a locked office), and considered as confidential material. The recorded data will be archived in electronic form on a secure PC by the IT department of the Cyceron research center.

Person responsible for archiving: the researcher in charge of the project, or a person mandated by them.

Possibility of destruction at the request of the participant: Participants may request that their survey responses be destroyed by giving us their anonymization number. We will destroy these files without consulting their content. Unless the participant objects, we will analyze the data collected between the date of signing the consent form and the date of the request to destroy the data.

Persons with access to the data: the researcher in charge of the project and their collaborators (other associated researcher and PhD student(s))

4. INFORMED CONSENT FORM INCLUDING INFORMATION TO BE GIVEN TO PARTICIPANTS

Clarification of the signing and delivery of the informed consent to the participant:

After ensuring that the information provided is understood by e-mail or telephone if necessary (Annex 1), the investigator will ask the participant for consent to participate in the study (Annex 2). If they accepts, the information leaflet and the consent form will be sent by

mail to the participant and the participant will return the completed and signed consent by mail to the coordinating investigator via a pre-stamped return envelope provided by the investigator (To the attention of Dr Vincent de la Sayette, Silver Santé Study, GIP CYCERON Boulevard Henri Becquerel - BP 5229 14074 CAEN cedex 5; original signature) and will inform the investigator in parallel by email. For participants who do not have an email address, we will send them the information notice and consent by mail (Annex 2).

5. REFERENCES

Allé MC, Potheegadoo J, Köber C, Schneider P, Coutelle R, Habermas T, Danion JM, Berna F (2015). Impaired coherence of life narratives of patients with schizophrenia. Sci Rep. 5:12934. doi: 10.1038/srep12934

Allé MC, d'Argembeau A, Schneider P, Potheegadoo J, Coutelle R, Danion JM, Berna F (2016). Self-continuity across time in schizophrenia: An exploration of phenomenological and narrative continuity in the past and future. Compr Psychiatry. 69:53-61. doi: 10.1016/j.comppsych.2016.05.001.

Brooks SK, Webster RK, Smith LE, Woodland L, Wessely S, Greenberg N, Rubin GJ (2020). The psychological impact of quarantine and how to reduce it: rapid review of the evidence. *Lancet* 395: 912–20

Michalska da Rocha B, Rhodes S, Vasilopoulou E, Hutton P (2020). Loneliness in Psychosis: A Meta-analytical Review. Schizophr Bull. 2018 Jan 13;44(1):114-125. doi: 10.1093/schbul/sbx036.

Narita Z, Stickley A, DeVylder J (2020). Loneliness and psychotic experiences in a general population sample. Jan 31. pii: S0920-9964(20)30034-7. doi: 10.1016/j.schres.2020.01.01.

INTERVENTIONAL STUDY IN COGNITIVELY INTACT SENIORS AIMING TO ASSESS THE EFFECTS OF MEDITATION TRAINING AND FOREIGN LANGUAGE LEARNING ON BEHAVIORAL, BIOLOGICAL AND NEUROIMAGING MEASURES

AGE-WELL

EVOLUTION TABLE		
**Version**	**DATE**	**REASON**
1.0	18/11/2016	Creation
2.0	20/12/2018	Update for protocol v6.0 + Precisions on analysis populations + exclusion of secondary objectives
3.0	15/10/2020	Precisions on some descriptive variables and suppression of the description of expert participants

TABLE OF APPROVALS				
	**AUTHOR**	**VALIDATION**	**APPROBATION**	
Name :	Julien Asselineau	Eric Frison	Trial Steering Committee or Scientific responsible	Hélène Esperou
Position :	EUCLID Biostatistician	EUCLID Methodologist		PRC Director
Date :				
Signature :	Julien ASSELINEAU<br>Signature numérique de Julien ASSELINEAU DN : cn=Julien ASSELINEAU, o=CHU de BORDEAUX, ou=USMR, email=julien.asselineau@u-bordeaux.fr, c=FR Date : 2020.10.15 16:50:21 +02'00'	Eric FRISON<br>Signature numérique de Eric FRISON Date : 2020.10.15 16:42:39 +02'00'		

Acronyms

CI Confidence Interval

EUCLID EUropean CLInical trials & Development

ICH International Conference on Harmonisation

IQR Interquartile Range

ITT Intent To Treat

PT Preferred Terms

SAE Serious Adverse Event

SOC System Organ Class

WP Work Package

PURPOSE

This document describes the steps and methods of the statistical analysis to implement in order to answer the research question of the study expressed by the primary objectives of the protocol. This document must be approved prior to analysis and database freezing.

Contents

ACRONYMS **2**

PURPOSE **2**

CONTENTS **3**

1. TRIAL SUMMARY **5**

1.1 CONTEXT 5

1.2 TRIAL OBJECTIVES 5
1.2.1 Main objectives 5
1.2.2 Secondary objectives 5

1.3 TRIAL DESIGN 6

1.4 INTERVENTIONS STUDIED 6

1.5 RANDOMIZATION 7

1.6 ELIGIBILITY CRITERIA 7
1.6.1 Inclusion criteria 7
1.6.2 Exclusion criteria 7

1.7 OUTCOMES 8
1.7.1 Primary outcomes 8
1.7.2 Secondary outcomes 8

2. STATISTICAL ASPECTS **10**

2.1 ANALYSIS STRATEGY 10
2.1.1 Definition of analysis populations 10
2.1.2 Definition of strategies with missing data 10
2.1.3 Type I error rate 10

2.2 DESCRIPTIVE ANALYSIS 10

2.3 COMPARATIVE ANALYSIS 11

2.4 CALCULATION CONVENTIONS FOR STATISTICAL ANALYSIS 11
2.4.1 Conventions for time calculations 11
2.4.2 Conventions for the calculation of the change (delta) of measurement between 2 visits 11
2.4.3 Conventions for BMI calculations 11
2.4.4 Conventions for handling missing data 11

2.5 SOFTWARE USED 12

3. ANALYSIS PLAN 13

3.1 DESCRIPTION OF RANDOMIZATION 13

3.2 DESCRIPTION OF PROTOCOL DEVIATIONS 13

3.3 DESCRIPTION OF ENROLMENT AND FOLLOW-UP 13

3.4 PARTICIPANTS' CHARACTERISTICS 13

3.4.1 At enrolment 13

3.4.2 At the end of the trial 14

3.5 PARTNERS' CHARACTERISTICS 14

3.6 ANALYSIS OF TRIAL OBJECTIVES 14

3.6.1 Primary objectives (Objectives 1 and 1bis) 14

REFERENCES 16

1. Trial summary

This document is based on the current version of the protocol v5.0 of 24/04/2017.

1.1 Context

As the number of older people in Europe grows, increasing healthy life years is a priority. As we live longer, ensuring good mental as well as physical health into later years is becoming ever more important. Cognitive decline, dementia (e.g. Alzheimer's disease, AD), sleep disturbances and depression, all related to psychological distress and anxiety, are significant drivers of reduced quality of life in older adults. This project builds on evidence that meditation practice have the potential to downregulate these adverse factors and positively impact mental and neurological conditions including AD.

1.2 Trial objectives

1.2.1 Main objectives

OBJECTIVE 1: To compare the effect of an 18-month mindfulness-based intervention versus no intervention on volume and perfusion of anterior cingulate cortex, in healthy older adults.

OBJECTIVE 1BIS: To compare the effect of an 18-month mindfulness-based intervention versus an 18-month intervention based on language learning, on volume and perfusion of insula, in healthy older adults.

1.2.2 Secondary objectives

- In relation to the interventions studied:

OBJECTIVE 2: To compare the effect of an 18-month mindfulness-based intervention, an 18-month intervention based on language learning and no intervention, on areas of cognition, psycho-affective/emotion, compassion, support, mindfulness, sleep, wellbeing and quality of life, and on biological and radiological measures, including sex-specific effects.

OBJECTIVE 2 bis: To estimate the maintenance of the effects of the interventions (mindfulness-based training and language learning) 21 months after the end of the intervention on areas of cognition, psycho-affective/emotion, compassion, support, mindfulness, sleep, wellbeing, quality of life (and on biological and radiological measures depending on budget availability).

OBJECTIVE 2 ter: To compare the long-term effects (21 months after the end of the intervention) of the two interventions (the 18-month mindfulness-based intervention and the 18-month intervention based on language learning) on areas of cognition, psycho-affective/emotion, compassion, support, mindfulness, sleep, wellbeing, quality of life (and on biological and radiological measures depending on budget availability).

OBJECTIVE 3: To compare the effect of an 18-month mindfulness-based intervention and of an 18-month intervention based on language learning vs. no intervention on participants' partners (perception of participant's changes, interactions with the participant, willingness to help the participant).

OBJECTIVE 3 bis: To estimate the effects of the interventions (meditation and foreign language learning) on participants' partners (perception of participant's changes, interactions with the participant, willingness to help the participant) 21 months after the end of the intervention.

OBJECTIVE 3 ter: To estimate the effects of the interventions (meditation and foreign language learning, during 18 months) (acceptance of the interventions, perception of changes...).

- Without relation to the interventions studied:

OBJECTIVE 4: To estimate the association between meditation practice and behavioral and biological measures (cognition, psycho-affective, lifestyle, blood, sleep and radiological measures), by comparing expert meditators and participants with no previous meditation practice.

OBJECTIVE 5: To identify neural signatures of mindfulness and compassion meditations in senior expert meditators.

OBJECTIVE 6: To estimate the association between lifestyle factors (cognitive activities, diet, physical activity), and psycho-affective, behavioral, and biological measures, including sex-specific or sex-mediated associations.

OBJECTIVE 7: To explore pathophysiological processes underlying Alzheimer's disease, by studying associations between several measures, and the association between structural and functional connectivity and lesion propagation, including sex-specific associations (link with data collected in the IMAP+ study).

OBJECTIVE 8: To compare PET-FDG measurement to early PET-Amyvid® perfusion measurement to better understand the links (and possible modifications of coupling) between metabolism and perfusion and compare the sensitivity of the two measures to evaluate the effects of the 2 interventions.

OBJECTIVE 9: To identify the determinants of well-being and mental health in ageing on participants of the passive condition (control group) and participant' partners on areas of cognition, psycho-affective/emotion, compassion, support, mindfulness, sleep, wellbeing, quality of life (and on biological and radiological measures depending on budget availability).

1.3 Trial design

- Cross-sectional observational study, aiming to estimate the association of meditation practice with health markers, by recruiting senior expert meditators and comparing them with participants without previous meditation practice.
- Randomized, controlled, observer-blinded monocentric, superiority trial, with three parallel groups (an 18-month mindfulness-based intervention, an 18-month intervention based on language learning and no intervention), on healthy older adults aged 65 years and more, and with no previous meditation practice.

1.4 Interventions studied

For both the mindfulness-based intervention and the intervention based on language learning, participants will follow:

- 2-hour weekly group sessions,
- Daily home practice (at least 20 minutes per day),
- Some days or half-days of more intense practice.

Each weekly group session will be divided into three parts: presentation, sharing, and practice. Participants will be strongly encouraged to participate in all those activities during the whole period of intervention (i.e. 18 months).

Meditation arm:
The intervention in the meditation arm will be provided by expert meditator instructors at the "Pôle de formations et de recherche en santé (PFRS)", in Caen. This intervention will consist in a secular programme of meditation training proposed in an objective of personal development and healthy ageing. This 18-month intervention programme is designed for individuals aged 65 years or older, retired, in good general health and leaving at home. The objective of this intervention programme is to develop mindfulness and compassion abilities as additional psychological resources to cope with challenges related with ageing on physical, cognitive and psychological aspects.

Language learning arm:
The intervention in the language (English) learning arm will be performed by teachers from the Carré International, a department from Caen University specialized in foreign language teaching and experienced in teaching to seniors. This intervention will consist in English exercises designed to reinforce each participant's abilities in understanding, writing and speaking.

No intervention arm:
No intervention will be delivered to participants assigned to this arm, during the study duration.

1.5 Randomization

The randomization list will be generated centrally by the biostatistician at the EUCLID clinical trials platform prior to the start of the study and kept confidentially in a secure environment. The sample size of each randomized group will be balanced with a ratio of about 1:1:1.

- Group 1: Meditation
- Group 2: Learning of English (active control)
- Group 3: No intervention

A working sheet presenting the entire randomization process will be kept confidentially at the EUCLID clinical trials platform

1.6 Eligibility criteria

1.6.1 Inclusion criteria

For all participants

- Age ≥ 65 years;
- To be autonomous (assessed with the following question asked by the clinician: “would you be able to live alone (from a functional (not affective) point of view »?);
- Live in their home;
- Educational level ≥ 7 years (from the Preparatory Course (First grade) included);
- To be registered to the social security system;
- To be motivated to effectively participate in the project and to sign the consent form in agreement with the local ethic committee (Comité de Protection des Personnes);
- Neuropsychological performances within the normal range (according to age, sex, and educational level), as assessed by the diagnostic battery.

For participants without previous meditation practice

- French mother tongue;
- Available for the trial duration (24 months);
- Retired since 1 year or more;
- No preference regarding the intervention group;
- Not having regularly or intensively practiced meditation or comparable practices (yoga, Qi Gong, Alexander technique)
    - more than one day per week for more than six months consecutively over the last 10 years,
    - intensively (internship or retreat > five consecutive days) over the past 10 years,
    - more than 25 days of retreats (cumulatively) prior to the last 10 years;
- Not speaking English fluently.

For expert meditators

- Formal meditation practice > 10 000 hours in the whole life, including at least 6 cumulated months of retreat;
- Daily meditation practice (at least 6 days/week, 45 minutes/day);
- Mindfulness meditation practice (i.e. mindfulness, Samatha/ Vipassana, Zazen (Zen), Shikantaza (Zen), focused attention, Mahamudra/ Dzogchen), and compassion mediation practice (i.e. tonglen, metta / karuna, meditation on Bodhichitta).

1.6.2 Exclusion criteria

- Contraindication to MRI or PET Amyvid® or PET Glucotep® ;
- For security reasons related with the use of Amyvid®, a blood sampling allowing to measure hepatic and renal functions will be performed at the V1 visit before the PET-Amyvid® scan. In case anomalies are detected of grade 3 or higher severity, the PET-Amyvid® scan will not be performed.
- Hypersensitivity to Amyvid® or Glucotep®
- Presence of a major neurological or psychiatric disorder (including an addiction to alcohol or drugs);

- History of cerebral disease (vascular, degenerative, physical malformation, tumor, or head trauma with loss of consciousness for more than an hour);
- Presence of a chronic disease or acute unstable illness (respiratory, cardiovascular, digestive, renal, metabolic, hematologic, endocrine or infectious);
- Current or recent medication that may interfere with cognitive action or radiological measures (psychotropic drugs, antihistamines, anti-Parkinsonian drugs, benzodiazepines, non-steroidal anti-inflammatory agents, antiepileptics, central analgesic and muscle relaxants);
- Under legal guardianship or incapacitation;
- Inclusion in another biomedical research protocol at baseline, if including use of a radiolabel for radiological measures;
- Physical of behavioral inabilities to perform the follow-up visits as planned in the study protocol. Sample size

For the randomized clinical trial with non-expert seniors:

The comparison of the mediation vs. no intervention arms will focus on the change in 1) volume and 2) metabolism of the anterior cingulate cortex from baseline to the end of the 18-months intervention, with an expected effect size of 0.75.
We expect the same effect size of 0.75 for the comparison between the meditation arm and learning of English arm on the change in 1) volume and 2) metabolism of the insula from baseline to the end of the 18-months intervention.
To demonstrate an effect size of 0.75 for each of the four comparisons, with 80% power and a two-sided type I error of 1.25% (Bonferroni correction for test multiplicity), 42 participants per arm need to be included for a total of 126 participants.

Experienced meditators :

For recruitment capacity reasons, it is possible to recruit 30 experienced meditators in the study. With the inclusion of 30 experienced meditators and 126 non-expert seniors, our power to highlight an effect size of 0.74 in the comparison of brain imaging data with a significance level of 5%, is 95%.

1.7 Outcomes

1.7.1 Primary outcomes

For the comparison between meditation intervention and no intervention (OBJECTIVE 1):
- Change in anterior cingulate cortex volume (T1-weighted MRI) from baseline to 18 months;
- Change in anterior cingulate cortex perfusion (early PET-Amyvid® scan) from baseline to 18 months).

For the comparison between meditation intervention and language learning intervention (OBJECTIVE 1BIS):
- Change in insula volume (T1-weighted MRI) from baseline to 18 months;
- Change in insula perfusion (early PET-Amyvid® scan) from baseline to 18 months).

1.7.2 Secondary outcomes

Behavioral outcomes (**OBJECTIVES 2, 4, 6 et 7**) **:**

One composite score per cognitive area assessed by the neuropsychological tests and the questionnaires. The composite scores will correspond to the mean of z-scores calculated from all the cognitive scores obtained for the corresponding cognitive function that will have a normal distribution and will not have floor or ceiling effect. (Additional exploratory analyses will be conducted on all individual behavioural measures obtained).

Radiological outcomes (OBJECTIVES 2, 4, 6 et 7):
- Grey and white matter volume in all brain voxels;
- hippocampal and hippocampal subfield volumes;
- Brain perfusion in all brain voxels;
- Fractional anisotropy and mean diffusivity in all brain voxels;
- number, size, type and location of white matter lesions;
- magnetic susceptibility index in all brain voxels;

- brain funcitonal connectivity measures in all brain voxels at rest in a non-meditative state ;
- Brain amyloid load (of the PET-Amyvid® radiotracer) measured in all brain voxels as well as in a global grey matter mask;
- Brain glucose uptake at rest;
- Brain activity measured with fMRI specifically associated with emotional processes (comparing emotional to neutral items) during the Rest-SoVT task.
- Brain activity measured with fMRI specifically associated with attentional processes (altertness, inhibition, sustained attention) during the AX-CPT task.

Sleep-related outcomes (OBJECTIVES 2, 4, 6 et 7):
- Subjective measures of sleep collected via sleep questionnaires
- Data collected by actimetrics record (mean duration of sleep, fragmentation indices during activity and resting states, regularity of the rest-activity cycle).
- Data collected during polysomnographic sleep record: Total sleep time, Sleep onset latency, Sleep efficiency, % of Stage N1, % of Stage N2, % of Stage N3, % of Stage REM sleep, Wake After Sleep Onset (WASO), Apnea-hypopnea index (/TST), Arousal index
- Data collected through Somno-Art.

Biological outcomes (OBJECTIVES 2, 4, 6 et 7):
- Total cholesterol, Triglycerides, HDL-cholesterol, LDL-cholesterol, serum urea, serum creatinine, gamma glutamyl transferase, glutamate-oxaloacetate transaminase and glutamate-pyruvate transaminase, complete blood count, high-sensitivity CRP, Cytokines, insulin-like growth factor-1, Super Oxide Dismutase, Brain Natriuretic Protein, Estradiol, Dehydroepiandrosterone-Sulfate, Cortisol, Sex Hormone Binding Globulin, bioavailable testosterone, Thyroid Stimulating Hormone, serotonin, tissue plasminogen activator, PAI 1, Brain-Derived Neurotrophic Factor, Tau and PhosphoTau, Aβ 42, lymphocytic immunotyping, mRNA Lymphocytes, telomeres and telomerase, ApoE4, cDNA/mRNA REST, GWAS, circulating antibodies.

Impact on participants' partners (OBJECTIVE 3):
- Questionnaires results

Neural signature of meditation practices (OBJECTIVE 5) :
- Measure of neural activity in all brain voxels measured with resting-state fMRI as well as with fMRI during the SOVT-Rest task, in a mindfulness state versus a non-meditative state.
  Measure of neural activity in all brain voxels measured with resting-state fMRI as well as with fMRI during the SOVT-Rest task, in a compassion state versus a non-meditative state.

Safety and acceptance (OBJECTIVE 2):
- Adverse events, measures of anxiety, depression, satisfaction, and well-being.

2. Statistical aspects

The statistical analysis of the primary outcomes (objectives 1 + 1bis) will be conducted by the biostatistician of EUCLID.
The statistical analysis of secondary outcomes will be performed by respective WPs dedicated to the evaluation of the different objectives as follows:
WP1 Meditation: Antoine Lutz
WP2 Lifestyle: Eider Arenaza-Urquijo
WP3 Attention: Fabienne Collette
WP4 Emotion: Olga Klimecki
WP5 Cognition and Wellbeing: Natalie Marchant
WP6 Biomarkers: Gaël Chételat
Details of the statistical aspects regarding those secondary objectives are listed in the corresponding Statistical Analysis Plan.

2.1 Analysis strategy

2.1.1 Definition of analysis populations

Only participants presenting at least one of the following conditions can be excluded from the analysis:

- participants who never followed the study strategy (if they had no knowledge of the group they were randomized in);
- participants wrongly included with an unsigned consent;
- participants wrongly included for major non respected eligibility criteria;
- participants who withdrew their informed consent.

Decisions about exclusion will be made by the Trial Scientific Committee after documentation of observations by the EUCLID platform, blinded to the strategy group and to the participants' evolution after enrolment.
Except for these exclusions, the participants who died or who were lost to follow-up, or who interrupted the intervention or who left the study will all be included in the analysis.

Intent to treat analysis: All randomized participant will be included in the analysis in the group to which they were initially randomized and all their data is used, regardless of protocol deviations during the trial.

Per protocol analysis: randomized participants who attended at least 20% of their allocated interventions classes, or neither meditation nor foreign language classes if in the passive control group, and with primary endpoints available.

2.1.2 Definition of strategies with missing data

Data available: inclusion of only participants whose data considered is available

Missing=failure: Any missing value will be replaced by the value corresponding to failure

Maximum bias strategy: Strategy comparing the results of two analyzes, one replacing missing data by a failure of the strategy in one group and by a success of the strategy in the other. And inversely in a second time.

2.1.3 Type I error rate

The statistical tests for the main objectives (1 + 1bis) will be performed with 1.25% error rate each, controlling the family-wise error rate to 5% for the 4 comparisons.

2.2 Descriptive analysis

The frequency and proportion of available data will be described for each variable.
Qualitative variables will be described in terms of number, proportion and exact binomial confidence interval of proportion.

Quantitative variables will be described in terms of absolute frequency, mean, standard deviation, confidence interval of the mean, median, interquartile range, minimum, and maximum.
Descriptive analysis will be performed overall and by intervention group.

2.3 Comparative analysis

Qualitatives variables will be compared between the groups with the χ2 test or the corrected χ2 or non-parametric Fisher's exact test, according to the values of expected frequency under the independence assumption. A logistic or polytomic model will be used to consider adjustments if necessary. The assumption of log-linearity of the association will be systematically checked.

Quantitative variables will be compared between 2 groups of intervention by Student t test if the conditions of validity are respected (normal distribution, homogeneous variances). If the variances are unequal between the 2 groups, a t-test for unequal variances will be used, if the distribution is not normal, a non-parametric Wilcoxon test will be used.

A linear regression model will be used to adjust on variables if necessary. The assumption of linearity of the association will be systematically checked. The other assumptions of the model (normality and homoscedasticity of residuals) will be studied. A transformation of the quantitative variable may be considered if necessary.

2.4 Calculation conventions for statistical analysis

2.4.1 Conventions for time calculations

- Time between 2 dates **in months** $= \frac{(\text{Date 2 - Date 1})}{30.4375}$
- Time between 2 dates **in years** will be estimated as the number of year between two dates. The function "YRDIF" of SAS will be used with the "Actual" option.

Convention for dates: Except in special cases, if the day is missing, the convention is to use the value "15", i.e.mid-month. If the day and month are missing, the convention is to use the value "1" for days and the value "7" for month, i.e. mid-year. This convention allows us to measure time estimates. The biostatistician makes these imputations at the time of analysis and consistency is checked at the time of data review.

2.4.2 Conventions for the calculation of the change (delta) of measurement between 2 visits

The difference between two measurements, one taken at D00 and a second taken at a visit posterior to D00 (follow-up visit DXX) will be calculated using the following formula:

- Delta = (measurement at DXX – measurement at D00)

2.4.3 Conventions for BMI calculations

- BMI = $= \frac{Weight(\text{kg})}{\text{Height}^2\ (\text{m})}$

2.4.4 Conventions for handling missing data

The following values are used in the database to describe the missing data:
- « NK » or « K »: Unknown

- « ND » or « D »: Not done
- « NA » or « A »: Not applicable

There will be no distinction in the statistical analyses according to the type of missing data.

2.5 Software used

The analyses will be performed using SAS® software (version 9.3 or higher).

3. Analysis plan

3.1 Description of randomization

- Description of the method of randomization: size of blocs, fixed or random method…
- Description of the deviations of randomization

3.2 Description of protocol deviations

For all eligibility criteria (inclusion and non-inclusion), the biostatistician will check that the inclusion criteria is "yes" and the non-inclusion criteria is "no" in the corresponding SAS table. Additional verifications can be made if needed.
The other following deviations are described per category:

- Randomized participants who did not receive the trial intervention
- Participants who received the wrong intervention
- Participants who received a forbidden concomitant treatment. The following therapies should be avoided: psychotropic drugs, antihistamines, anti-Parkinsonian drugs, benzodiazepines, non-steroidal anti-inflammatory agents, antiepileptics, analgesic drugs and muscle relaxants (see exclusion criteria)

Deviations per participant are described.

3.3 Description of enrolment and follow-up

- Participant enrolment and follow-up are described based on the study profile as defined in the CONSORT recommendations (3). According to CONSORT diagram,
  - Participants assessed for eligibility (n)
  - Participants excluded and reasons (n, %)
  - Participants randomized in total and in each group (n, %)
  - Participants having received the trial intervention (n, %)
  - Participants who did not received allocated intervention (n, %)
  - Participants lost to follow-up (n, %)
  - Participants to include in the analysis (n, %)
  - Participants to exclude from the analysis and reasons (n, %)
- Review of the database: number of records in each table

3.4 Participants' characteristics

The following characteristics are described for all enrolled participants overall and per group.

3.4.1 At enrolment

- Socio-demographic characteristics
  - Gender
  - Age (years)
  - Level of education (in number of years from the 1st year of school = CP in France)
  - Number of years in retirement
  - Memory Complaint (score at MacNair)
  - Familial history of AD (percentage yes)

- Clinical characteristics
  - Diastolic and systolic blood pressure (mmHg)
  - Height (m), weight (kg) and BMI (in kg/m², in classes)
  - Hip and waist circumferences (cm)
- Diagnostic battery test
  - Laterality-Edimburgh
  - MMSE
  - MADRS
  - Grober and Buschke (FR1+FR2+FR3)
  - Modified (Wisconsin) Cart Sorting Test
  - English test

3.4.2 At the end of the trial

- Death
- Compliance
  - Total number of classes attended
  - Total number of reported practice sessions
  - Total number of minutes of formal practice
  - Total number of minutes of informal practice
- Adverse events
  - Severity
  - Relationship to the study intervention or radiopharmaceutical drugs

3.5 Partners' characteristics

- Socio-demographic characteristics
  - Gender
  - Age (years)
  - Relationship to participant
  - Live with participant (Yes/No)
  - Number of years knowing the participant
  - Number of hours per week spent with the participant

3.6 Analysis of trial objectives

3.6.1 Primary objectives (Objectives 1 and 1bis)

The difference between two measures will be calculated with the following formula:

Delta = (measure at 18 months – measure at inclusion)

The four comparisons will be first conducted according to the intent to treat principle with “missing = failure” strategy (see §2.3 for comparative statistical tests) for handling missing data on primary endpoints. A failure is defined as the minimum change between baseline and 18 months observed in the whole population study. This first analysis will be the main result of the study.

This first analysis will be complemented with a sensitivity analysis to check the robustness of the main analysis to missing data. The maximum bias strategy for missing data will be performed. Missing data will be replaced by a failure of the strategy in one group and by a success of the strategy in the other, and vice versa. The success is defined as the maximum change between baseline and 18 months observed in the whole population study. The failure is defined as the minimum change between baseline and 18 months observed in the whole population study.

We will be able to conclude that the first analysis is robust if all sensitivity analyses have a similar conclusion.

The main and sensitivity comparative analyses will be performed with adjustment on baseline prognostic factors (age, education, gender, MMSE) (see §2.3 for statistical models).

A per-protocol analysis will be performed, using the strategy defined in §2.1.1.

An analysis of the effect of the exposure to meditation (number of hours of practice, number of sessions...) will be performed using the strategy described in §2.3.

REFERENCES

1. Guideline ICH - E9: Statistical Principles for Clinical Trials. 1998.

2. Guideline ICH - E3: Structure and Content of Clinical Trials. 1995.

3. Moher D, Schulz KF, Altman DG. The CONSORT statement: revised recommendations for improving the quality of reports of parallel-group randomized trials. Lancet. 14 avr 2001;357(9263):1191‑ 1194.

4. Basic Statistical Reporting for Articles Published in Biomedical Journals: The « Statistical Analyses and Methods in the Published Literature » or The SAMPL Guidelines” | The EQUATOR Network [Internet]. [cité 9 mai 2014]. Disponible sur: http://www.equator-network.org/reporting-guidelines/sampl/

STATISTICAL ANALYSIS PLAN – SECONDARY OBJECTIVES

INTERVENTIONAL STUDY IN COGNITIVELY INTACT SENIORS AIMING TO ASSESS THE EFFECTS OF MEDITATION TRAINING AND FOREIGN LANGUAGE LEARNING ON BEHAVIORAL, BIOLOGICAL AND NEUROIMAGING MEASURES

AGE-WELL

EVOLUTION TABLE		
**Version**	**DATE**	**REASON**
0.3	25/09/2018	Creation
1.0	19/01/2021	Update with protocols v9.0 and v10.0

TABLE OF APPROVALS			
	**AUTHOR**	**VALIDATION**	**APPROBATION**
Name :	Géraldine Poisnel	Gaël Chételat	Hélène Espérou
Position :	Project Manager	Scientific responsible on behalf of the Trial Steering Committee	PRC Director
Date :	20/01/2020	20/01/2020	
Signature :	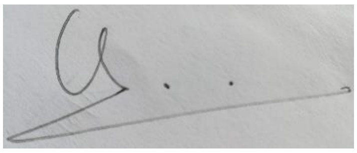	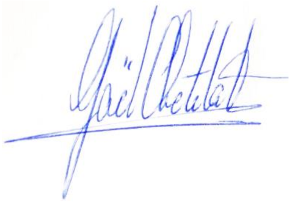	

Acronyms

CI Confidence Interval

EUCLID EUropean CLInical trials & Development

ICH International Conference on Harmonisation

WP Work Package

PURPOSE

This document describes the steps and methods of the statistical analysis to implement in order to answer the research question of the study expressed by the secondary objectives (objectives 2 to 9) of the protocol. This document must be approved by the trial steering committee (TSC) prior to any analysis and database freezing.

Contents

ACRONYMS **2**

PURPOSE **2**

CONTENTS **3**

TRIAL SUMMARY **5**

CONTEXT 5

TRIAL OBJECTIVES 5

Main objectives 5

Secondary objectives 5

TRIAL DESIGN 6

INTERVENTIONS STUDIED 6

RANDOMIZATION 6

ELIGIBILITY CRITERIA 7

Inclusion criteria 7

Exclusion criteria 7

OUTCOMES 8

Primary outcomes 8

Secondary outcomes 8

STATISTICAL ASPECTS **10**

ANALYSIS STRATEGY 10

Definition of analysis populations 10

Definition of strategies with missing data 10

Type I error rate 11

DESCRIPTIVE ANALYSIS 11

COMPARATIVE/CORRELATIONAL ANALYSIS 11

ANALYSIS OF IMAGING DATA 11

CALCULATION CONVENTIONS FOR STATISTICAL ANALYSIS 12

Conventions for time calculations 12

Conventions for the calculation of the change (delta) of measurement between 2 visits .. 12

Conventions for BMI calculations 12

Conventions for handling missing data 12

SOFTWARE USED 12

ANALYSIS PLAN 13

PARTICIPANTS CHARACTERISTICS 13
At enrolment 13
At the end of the trial 13

PARTNERS' CHARACTERISTICS 14

ANALYSIS OF TRIAL OBJECTIVES 14
Primary objectives (Objectives 1 and 1bis) 14
Secondary objectives 14

REFERENCES 21

Trial summary

This document is based on protocol v9.0 of 08 July 2020 and protocol v10.0 of 10 December 2020.

Context

As the number of older people in Europe grows, increasing healthy life years is a priority. As we live longer, ensuring good mental as well as physical health into later years is becoming ever more important. Cognitive decline, dementia (e.g. Alzheimer's disease, AD), sleep disturbances and depression, all related to psychological distress and anxiety, are significant drivers of reduced quality of life (QoL) in older adults. This project builds on evidence that meditation practice have the potential to downregulates these adverse factors and positively affects mental and neurological conditions including AD.

Trial objectives

Main objectives

OBJECTIVE 1: To compare the effect of an 18-month mindfulness-based intervention versus no intervention on volume and perfusion of anterior cingulate cortex, in healthy older adults.

OBJECTIVE 1BIS: To compare the effect of an 18-month mindfulness-based intervention versus an 18-month intervention based on language learning, on volume and perfusion of insula, in healthy older adults.

Secondary objectives

- In relation to the interventions studied:

OBJECTIVE 2: To compare the effect of an 18-month mindfulness-based intervention, an 18-month intervention based on language learning and no intervention, on areas of cognition, psycho-affective/emotion, compassion, support, mindfulness, sleep, wellbeing, quality of life, and on biological and radiological measures, including sex-specific effects;

OBJECTIVE 2bis: To estimate the maintenance of the effects of the interventions (mindfulness-based training and language learning) 29 mois +/- 6 mois months after the end of the intervention on areas of cognition, psycho-affective/emotion, compassion, support, mindfulness, sleep, wellbeing, quality of life (and on biological and radiological measures depending on budget availability);

OBJECTIVE 2ter: To compare the long-term effects (29 mois +/- 6 mois months after the end of the intervention) of the two interventions (the 18-month mindfulness-based intervention and the 18-month intervention based on language learning) on areas of cognition, psycho-affective/emotion, compassion, support, mindfulness, sleep, wellbeing, quality of life (and on biological and radiological measures depending on budget availability);

OBJECTIVE 3: To compare the effect of an 18-month mindfulness-based intervention and of an 18-month intervention based on language learning vs. no intervention on participants' partners (perception of participant's changes, interactions with the participant, willingness to help the participant).

OBJECTIVE 3bis: To estimate the effects of the interventions (meditation and foreign language learning) on participants' partners (perception of participant's changes, interactions with the participant, willingness to help the participant) 21 months after the end of the intervention.

OBJECTIVE 3TER: To estimate the effects of the interventions (meditation and foreign language learning, during 18 months) (acceptance of the interventions, perception of changes…).

- Without relation to the interventions studied:

OBJECTIVE 4: To estimate the association between meditation practice and behavioral and biological measures (cognition, psycho-affective, lifestyle, blood, sleep and radiological measures), by comparing expert meditators and participants with no previous meditation practice;

OBJECTIVE 5: To identify neural signatures of mindfulness and compassion meditations in senior expert meditators;

OBJECTIVE 6: To estimate the association between lifestyle factors (cognitive activities, diet, physical activity), and psycho-affective, behavioral, and biological measures, including sex-specific or sex-mediated associations;

Objective 7: To explore pathophysiological processes underlying Alzheimer's disease, by studying associations between several measures, and the association between structural and functional connectivity and lesion propagation, including sex-specific associations (link with data collected in the IMAP+ study).

Objective 8: To compare PET-FDG measurement to early PET-Amyvid® perfusion measurement to better understand the links (and possible modifications of coupling) between metabolism and perfusion and compare the sensitivity of the two measures to evaluate the effects of the 2 interventions;

Objective 9: To identify the determinants of well-being and mental health in ageing on participants of the passive condition (control group) and participant' partners on areas of cognition, psycho-affective/emotion, compassion, support, mindfulness, sleep, wellbeing, quality of life (and on biological and radiological measures depending on budget availability).

Trial design

- Randomized, controlled, observer-blinded monocentric, superiority trial, with three parallel groups (an 18-month mindfulness-based intervention, an 18-month intervention based on language learning and no intervention), on healthy older adults aged 65 years and more and with no previous meditation practice.
- Cross-sectional observational study, aiming to estimate the association of meditation practice with health markers, by recruiting senior expert meditators and comparing them with participants without previous meditation practice.

Interventions studied

For both the mindfulness-based intervention and the intervention based on language learning, participants will follow:

- 2-hour weekly group sessions,
- Daily home practice (at least 20 minutes per day),
- Some days or half-days of more intense practice.

Each weekly group session will be divided into three parts: presentation, sharing, and practice. Participants will be strongly encouraged to participate in all those activities during the whole period of intervention (i.e. 18 months).

Meditation arm:
The intervention in the meditation arm will be provided by expert meditator instructors at the "Pôle de formations et de recherche en santé (PFRS)", in Caen. This intervention will consist in a secular programme of meditation training proposed in an objective of personal development and healthy ageing. This 18-month intervention programme is designed for individuals aged 65 years or older, retired, in good general health and leaving at home. The objective of this intervention programme is to develop mindfulness and compassion abilities as additional psychological resources to cope with challenges related with ageing on physical, cognitive and psychological aspects.

Language learning arm:
The intervention in the language (English) learning arm will be performed by teachers from the Carré International, a department from Caen University specialized in foreign language teaching and experienced in teaching to seniors. This intervention will consist in English exercises designed to reinforce each participant's abilities in understanding, writing and speaking.

No intervention arm:
No intervention will be delivered to participants assigned to this arm, during the study duration.

Randomization

The randomization list will be generated centrally by the biostatistician at the EUCLID clinical trials platform prior to the start of the study and kept confidentially in a secure environment. The sample size of each randomized group will be balanced with a ratio of about 1:1:1.

- Group 1: Meditation

- Group 2: Learning of English (active control)

- Group 3: No intervention

A working sheet presenting the entire randomization process will be kept confidentially at the EUCLID clinical trials platform

Eligibility criteria

Inclusion criteria

For all participants

- Age ≥ 65 years;
- To be autonomous (assessed with the following question asked by the clinician: “would you be able to live alone (from a functional (not affective) point of view »?);
- Live in their home;
- Educational level ≥ 7 years (from the Preparatory Course (First grade) included);
- To be registered to the social security system;
- To be motivated to effectively participate in the project and to sign the consent form in agreement with the local ethic committee (Comité de Protection des Personnes);
- Neuropsychological performances within the normal range (according to age, sex, and educational level), as assessed by the diagnostic battery.

For participants without previous meditation practice

- French mother tongue;
- Available for the trial duration (24 months);
- Retired since 1 year or more;
- No preference regarding the intervention group;
- Not having regularly or intensively practiced meditation or comparable practices (yoga, Qi Gong, Alexander technique)
    - more than one day per week for more than six months consecutively over the last 10 years,
    - intensively (internship or retreat > five consecutive days) over the past 10 years,
    - more than 25 days of retreats (cumulatively) prior to the last 10 years;
- Not speaking English fluently.

For expert meditators

- Formal meditation practice > 10 000 hours in the whole life, including at least 6 cumulated months of retreat;
- Daily meditation practice (at least 6 days/week, 45 minutes/day);
- Mindfulness meditation practice (i.e. mindfulness, Samatha/ Vipassana, Zazen (Zen), Shikantaza (Zen), focused attention, Mahamudra/ Dzogchen), and compassion mediation practice (i.e. tonglen, metta / karuna, meditation on Bodhichitta).

Exclusion criteria

- Contraindication to MRI or PET Amyvid® or Glucotep®;
- For security reasons related with the use of Amyvid® and Glucotep®, a blood sampling allowing to measure hepatic and renal functions will be performed at the V1 visit and V4 (after 18 months) before the PET scan. In particular, the glomerular filtration rate will be calculated from the results obtained. In case of kidney failure, hepatic failure, diabetes (glycaemia>1.6 g/L) or any other biological anomalies of grade 3 or higher severity detected, the PET scan will not be performed.
- Hypersensitivity to Amyvid® or to Glucotep®;
- Presence of a major neurological or psychiatric disorder (including an addiction to alcohol or drugs);
- History of cerebral disease (vascular, degenerative, physical malformation, tumor, or head trauma with loss of consciousness for more than an hour);
- Presence of a chronic disease or acute unstable illness (respiratory, cardiovascular, digestive, renal, metabolic, hematologic, endocrine or infectious);
- Current or recent medication that may interfere with cognitive action or radiological measures (psychotropic drugs, antihistamines acting anticholinergic, anti-Parkinsonian drugs, benzodiazepines, non-steroidal anti-inflammatory agents, antiepileptic, central analgesic and muscle relaxants agents);
- Under legal guardianship or incapacitation;
- Inclusion in another biomedical research protocol at baseline, if including use of a radiolabel for radiological measures;

- Physical of behavioral inabilities to perform the follow-up visits as planned in the study protocol.

Sample size

For the randomized clinical trial with non-expert seniors:

The comparison of the meditation vs. no intervention arms will focus on the change in 1) volume and 2) metabolism of the anterior cingulate cortex from baseline to the end of the 18-months intervention, with an expected effect size of 0.75.
We expect the same effect size of 0.75 for the comparison between the meditation arm and learning of English arm on the change in 1) volume and 2) metabolism of the insula from baseline to the end of the 18-months intervention.
To demonstrate an effect size of 0.75 for each of the four comparisons, with 80% power and a two-sided type I error of 1.25% (Bonferroni correction for test multiplicity), 42 participants per arm need to be included for a total of 126 participants.
In order to increase the statistical power of the secondary analysis on the PET-FDG examination carried out starting from the second wave of inclusion, 24 additional participants will be recruited (additional 8 participants per group) for a total of 150 participants.

Experienced meditators:

For recruitment capacity reasons, it is possible to recruit 30 experienced meditators in the study. With the inclusion of 30 experienced meditators and 126 non-expert seniors, our power to highlight an effect size of 0.74 in the comparison of brain imaging data with a significance level of 5%, is 95%.

Outcomes

Primary outcomes

For the comparison between meditation intervention and no intervention (OBJECTIVE 1):
- Change in anterior cingulate cortex volume (T1-weighted MRI) from baseline to 18 months;
- Change in anterior cingulate cortex perfusion (early PET-Amyvid® scan) from baseline to 18 months).

For the comparison between meditation intervention and language learning intervention (OBJECTIVE 1BIS):
- Change in insula volume (T1-weighted MRI) from baseline to 18 months;
- Change in insula perfusion (early PET-Amyvid® scan) from baseline to 18 months).

Secondary outcomes

Behavioral outcomes (OBJECTIVES 2, 4, 6, 7 and 9):

The main behavioural outcome will correspond to the comparison between meditation intervention and language learning intervention on the global metacognitive composite score, consisting in the mean of the attentional, constructive and deconstructive subscores as defined in (Dahl et al., 2015) and updated in (Schlosser et al., Submitted); see Section "*Analysis of trial objectives"* below for further details about this score.

Additional composite scores will be computed as z-scores measured from the neuropsychological tests and/or the questionnaires. Composite scores will correspond to the mean of z-scores calculated from all the cognitive scores obtained for the corresponding cognitive function that will have a normal distribution and will not have floor or ceiling effect. Additional exploratory analyses will be conducted on all individual behavioural measures obtained.

Radiological outcomes (OBJECTIVES 2, 4, 6, 7 and 8):
- Grey and white matter volume in all brain voxels;
- hippocampal and hippocampal subfield volumes;
- Brain perfusion in all brain voxels;
- Fractional anisotropy and mean diffusivity in all brain voxels;
- number, size, type and location of white matter lesions;
- magnetic susceptibility index in all brain voxels;
- brain funcitonal connectivity measures in all brain voxels at rest in a non-meditative state ;
- Brain amyloid load (of the PET-Amyvid® radiotracer) measured in all brain voxels as well as in a global grey matter mask;

- Brain activity measured with fMRI specifically associated with emotional processes (comparing emotional to neutral items) during the Rest-SoVT task.
- Brain activity measured with fMRI specifically associated with attentional processes (altertness, inhibition, sustained attention) during the AX-CPT task.

Sleep-related outcomes (OBJECTIVES 2, 4, 6, 7 and 9):
- Subjective measures of sleep collected via sleep questionnaires
- Data collected by actimetrics record (mean duration of sleep, fragmentation indices during activity and resting states, regularity of the rest-activity cycle).
- Data collected during polysomnographic sleep record: Total sleep time, Sleep onset latency, Sleep efficiency, % of Stage N1, % of Stage N2, % of Stage N3, % of Stage REM sleep, Wake After Sleep Onset (WASO), Apnea-hypopnea index (/TST), Arousal index

Biological outcomes (OBJECTIVES 2, 4, 6 and 7):
- Total cholesterol, Triglycerides, HDL-cholesterol, LDL-cholesterol, urea, creatinine, gamma glutamyl transferase, glutamate-oxaloacetate transaminase and glutamate-pyruvate transaminase, complete blood count, glycemia, insulin, high-sensitivity CRP, Cytokines, insulin-like growth factor-1, Peroxyredoxin, Brain Natriuretic Protein, Estradiol, Dehydroepiandrosterone-Sulfate, Cortisol, Sex Hormone Binding Globulin, bioavailable testosterone, Thyroid Stimulating Hormone, serotonin, tissue plasminogen activator, PAI 1, Brain-Derived Neurotrophic Factor, Tau and PhosphoTau, Aβ 40 & 42, NFL, lymphocytic immunotyping, telomeres and telomerase, ApoE4, cDNA/mRNA REST, GWAS,.

Impact on participants' partners (OBJECTIVE 3 and 9):
- Questionnaires results

Neural signature of meditation practices (OBJECTIVE 5) :
- Measure of neural activity in all brain voxels measured with resting-state fMRI as well as with fMRI during the SOVT-Rest task, in a mindfulness state versus a non-meditative state.
  Measure of neural activity in all brain voxels measured with resting-state fMRI as well as with fMRI during the SOVT-Rest task, in a compassion state versus a non-meditative state.

Safety and acceptance (OBJECTIVE 2, 3ter):
- Adverse events, measures of anxiety, depression, satisfaction, and well-being.

Statistical aspects

The statistical analysis of the primary outcomes (objectives 1 + 1bis) will be conducted by the biostatistician team of EUCLID. Details of the statistical aspects regarding the primary objectives are listed in the corresponding Statistical Analysis Plan written by EUCLID.
The statistical analysis of secondary outcomes will be performed by respective WPs dedicated to the evaluation of the different objectives as follows:
WP1 Meditation: Antoine Lutz
WP2 Lifestyle: Eider Arenaza-Urquijo/Julie Gonneaud
WP3 Attention: Fabienne Collette
WP4 Emotion: Olga Klimecki
WP5 Cognition and Wellbeing: Natalie Marchant
WP6 Biomarkers: Gaël Chételat

Analysis strategy

Definition of analysis populations

The statistical analyses for the main objectives (primary endpoints) are described in the corresponding document.
For the secondary objectives (secondary endpoints) two types of analyses will be performed:
Analyses will be performed in intent to treat. All randomized participant will be included in the analysis in the group to which they were initially randomized and all their data is used, regardless of protocol deviations during the trial. Any missing value (missing= failure) will be replaced by the value corresponding to failure.
In order to assess the robustness of the results, a sensitivity analysis for the missing data will be performed using the maximum bias strategy using values reflecting failure and success (minimum and maximum difference, respectively) estimated on the observed data of all groups.
In addition, “per protocol” analyses will be performed: Participants’ data will be analyzed in the group according to the treatment they actually received. Per protocol population includes "participants who attended at least 20% of intervention classes, or neither meditation nor foreign language training for participants in the passive control group.

For exploratory analyses not related with the intervention (e.g. analyses on baseline data independent from intervention group), all participants will be included in the analyses.

Decisions about which of these analysis strategies should be used will depend on the specific analyses and decided by the PI and validated by the ExCom.

Only participants presenting at least one of the following conditions can be excluded from the analysis:

- participants wrongly included with an unsigned consent;
- participants wrongly included for major non respected eligibility criteria;
- participants who withdrew their informed consent.

Decisions about exclusion will be made by the Trial Scientific Committee after documentation of observations by the EUCLID platform, blinded to the strategy group and to the participants’ evolution after enrolment.
Except for these exclusions, the participants who died or who were lost to follow-up, or who interrupted the intervention or who left the study will all be included in the analysis.

Definition of strategies with missing data

Data available: inclusion of only participants whose criterion considered is filled

Missing=failure: Any missing value will be replaced by the value corresponding to failure

Maximum bias strategy: Strategy comparing the results of two analyzes, one replacing missing data by a failure of the strategy in one group and by a success of the strategy in the other. And inversely in a second time.

Type I error rate

For the main secondary outcome, the main effect (of the meditation intervention compared to the English intervention) on the global metacognitive composite score will be tested with a two-sided type I error rate α=5%; if significant, the main effect on each of the 3 composite subscores (attentional, constructive and deconstructive) will be tested with a Bonferroni correction (α=5/3%=0.0166%) to strictly control for type I error inflation.
The evolution of the same scores in the passive control group will be provided for the sake of description.
Each statistical test for the other secondary endpoints will be performed with a two-sided type I error rate α=5%.

Descriptive analysis

The frequency and proportion of available data will be described for each variable.
Qualitative variables will be described in terms of number, proportion and exact binomial confidence interval of proportion.
Quantitative variables will be described in terms of absolute frequency, mean, standard deviation, confidence interval of the mean, median, interquartile range, minimum, and maximum.
Descriptive analysis will be performed overall and by intervention group.

Comparative/Correlational analysis

Qualitatives variables will be compared between the groups with the χ2 test or the corrected χ2 or non-parametric Fisher's exact test, according to the values of expected frequency under the independence assumption. A logistic or polytomic model will be used to consider adjustments if necessary. The assumption of log-linearity of the association will be systematically checked.

Quantitative variables will be compared between 2 groups of intervention by Student t test if the conditions of validity are respected (normal distribution, homogeneous variances). If the variances are unequal between the 2 groups, a t-test for unequal variances will be used, if the distribution is not normal, a non-parametric Wilcoxon test will be used.
Quantitative variables will be compared between 3 groups of intervention with analysis of variance (ANOVA) if the conditions of validity are respected (normal distribution, homogeneous variances). If the distribution is not normal, a non-parametric Kruskal-Wallis test will be used.
Covariates will be added to the model, or linear regression model will be used to adjust on variables, if necessary. The assumption of linearity of the association will be systematically checked. The other assumptions of the model (normality and homoscedasticity of residuals) will be studied. A transformation of the quantitative variable may be considered if necessary.
Multiple regression models, general linear models and/or independent component analyses, principal component analyses or confirmatory factor analyses would be performed to assess the relationships between variables and/or to extract components from multiple variables,

Analysis of imaging data

For voxel-wise analyses, MRI and PET data will be processed and analyzed using Statistical Parametric Mapping software (Wellcome Trust Centre for Neuroimaging, London, United Kingdom). T1-weighted MRI images will be segmented, spatially normalized to the MNI space, modulated to correct for nonlinear warping effects using the Voxel-Based Morphometry toolbox and smoothed. PET images will be co-registered onto corresponding MRI, normalized using the deformation parameters defined from the VBM procedure performed on the corresponding MRI, scaled using the mean PET value of the cerebellar gray matter and smoothed. Quality control for raw data and for each step of data processing will be performed by experts. PET data will be corrected for partial volume effects (PVE, PMOD Technologies) using a 2-tissue compartment model and analyses will be repeated with PVE-corrected PET data whenever required. Statistical analyses will include group comparisons (with ANOVA) and correlations (regression analyses) with SPM. For T1-MRI, the freesurfer software will also be used for complementary analyses on cortical thickness. Analyses will include age, MMSE, sexe and education as covariates when required. Other possible covariates (risk factors for AD, cardiovascular risk factors, neuroticism) will be included according to analyses.
For resting-state fMRI data, data will be processed as described in La Joie et al. (2014), including slice timing correction, realignment to the first volume, spatial normalization, smoothing, masking to include only gray matter voxels and exclude the cerebellum (based on the T1-weighted and non EPI-T2* volumes) and temporal band pass filtering (0.01–0.08 Hz). Analyses will include seed-based functional connectivity analyses and independent component analyses (see Mevel et al., 2013; La Joie et al., 2014; Arenaza-Urquijo, 2015).

For fMRI data acquired during attentional and emotional tasks, preprocessing steps (including slice timing correction, realignment to the first volume, spatial normalization and smoothing will be performed using Statistical Parametric Mapping software (Wellcome Trust Centre for Neuroimaging, London, United Kingdom). Statistical analyses will include comparison of brain activity between various task condition for the different groups using ANOVAs and regression analyses. Due to task specificities, these analyses will be preferentially performed with a multivariate (PLS) technique for the attentional task and an univariate (SPM) technique for the emotional task. Analyses will include age, MMSE, sex and education as covariates when required. Other possible covariates (risk factors for AD, cardiovascular risk factors, neuroticism) will be included according to analyses. Based on these results, supplementary analyses will be performed to determine brain areas common to the attentional and emotional tasks in each group.

Calculation conventions for statistical analysis

Conventions for time calculations

- Time between 2 dates **in months** $= \frac{(\text{Date 2 - Date1})}{30.4375}$
- Time between 2 dates **in years** will be estimated as the number of year between two dates. The function “YRDIF” of SAS will be used with the “Actual” option.

Convention for dates: Except in special cases, if the day is missing, the convention is to use the value “15”, i.e.mid-month. If the day and month are missing, the convention is to use the value “1” for days and the value “7” for month, i.e. mid-year. This convention allows us to measure time estimates. The biostatistician makes these imputations at the time of analysis and consistency is checked at the time of data review.

Conventions for the calculation of the change (delta) of measurement between 2 visits

The difference between two measurements, one taken at D00 and a second taken at a visit posterior to D00 (follow-up visit DXX) will be calculated using the following formula:

- Delta = (measurement at DXX – measurement at D00)

Conventions for BMI calculations

- BMI = $= \frac{Weight(\text{kg})}{\text{Height}^2\ (\text{m})}$

Conventions for handling missing data

The following values are used in the database to describe the missing data:

- « NK » or « K »: Unknown
- « ND » or « D »: Not done
- « NA » or « A »: Not applicable

There will be no distinction in the statistical analyses according to the type of missing data.

Software used

The analyses will be performed using Statistica, Matlab/SPM and R softwares (last version version or otherwise indicated); other softwares might be used and this will be indicated in the corresponding article.

Analysis plan

Participants characteristics

The following characteristics are described for all enrolled participants overall and per group.

At enrolment

- Socio-demographic characteristics
  - Gender
  - Age (years)
  - Level of education (in number of years from the 1st year of school = CP in France)
  - Number of years in retirement
  - Memory Complaint (score at MacNair)
  - Familial history of AD (percentage yes)
- Clinical characteristics
  - Diastolic and systolic blood pressure (mmHg)
  - Height (m), weight (kg) and BMI (in kg/m^2, in classes)
  - Hip and waist circumferences (cm)
- Diagnostic battery test
  - Laterality-Edimburgh
  - MMSE
  - MADRS
  - Grober and Buschke
  - Short Version Winsconsin
  - English test
- For expert meditators,
  - Total number of hours in life practicing formal meditation
  - Total number of hours in life practicing formal meditation in retreats
  - Total number of daily meditation hours in life
  - Frequency of daily practice (days per week)
  - Total number of meditation hours in life in practices related to
    - compassion and loving-kindness meditation
    - concentration related meditations
    - mindfulness/wisdom related meditations
    - in ritual-related practices (chanting, puja,…)

At the end of the trial

- Death

- Compliance
    - Total number of formal home practice sessions and hours of
        - Total number of classes attended
        - Total number of reported practice sessions
        - Total number of minutes of formal practice
- Total number of minutes of informal practice Adverse events
    - Severity
    - Relationship to the study intervention or radiopharmaceutical drugs

Partners' characteristics

- Socio-demographic characteristics
    - Gender
    - Age (years)
    - Relationship to participant
    - Live with participant (Yes/No)
    - Number of years knowing the participant
    - Number of hours per week spent with the participant

Analysis of trial objectives

Primary objectives (Objectives 1 and 1bis)

See the attached SAP document for primary objectives

Secondary objectives

Objective 2 (WP 1 to 6)

For the main secondary outcome, the difference between the inclusion and 18 months of i) the global metacognitive composite score, and ii) the attentional, constructive and deconstructive subscores, will be compared between the meditation intervention and language learning intervention groups.

These scores will be computed as described in (Schlosser et al., Submitted). Briefly, the metacognitive composite score drew from the following self-report measures employed in the Age-Well randomised controlled trial (See Poisnel et al. for details and corresponding references): the Compassionate Love Scale (CLS; stranger-humanity version), the Drexel Defusion Scale (DDS), the Five Facet Mindfulness Questionnaire (FFMQ), the Interpersonal Reactivity Index (IRI), the Multidimensional Assessment of Interoceptive Awareness Version 2 (MAIA-2), and the Prosocialness Scale. Amongst those, seven subscales were judged to primarily capture attentional aspects: noticing (MAIA subscale), attention regulation (MAIA), emotional awareness (MAIA), self-regulation (MAIA), body listening (MAIA), observing (FFMQ), and acting with awareness (FFMQ). Four (sub)scales were judged to primarily capture constructive aspects: the CLS, empathic concern (IRI subscale), perspective taking (IRI), and the Prosocialness Scale. Another four scales were judged to primarily capture deconstructive aspects: the DDS, non-judging (FFMQ), non-reactivity (FFMQ), distress (IRI).

To compute each composite score, scale scores will be first standardised using their baseline mean and standard deviation, and the sum of the standardised scale scores will then be divided by the number of scales included in the respective composite. Scales will be reverse-scored if higher total scores reflect worse functioning. Ergo, higher composite scores will indicate higher capacities in the psychological processes characterising the three meditation families. Participants with missing scale scores will not be included in the composite score to which this scale is assigned.

The evolution of the same scores in the passive control group will be provided for the sake of description. Moreover, the difference between the inclusion and 18 months of all additional composite scores and individual behavioural cognitive, psychoaffectives, life, blood, sleep and imaging measures will be compared between

the three groups. (see Section "*Descriptive analysis"* above for comparative methods). Mixed models will be used to evaluate the evolution from baseline to M18, taking into account observations of M9. Analyses of the effect of the exposure to meditation/English learning (number of hours of practice, number of sessions...) will also be performed.

Objective 2bis (WP 1 to 6)

The difference between the post-intervention measures (18 months) and the measures made 29 months after the end of the intervention for all cognitive, psychoaffective, life factors, sleep measures (and blood and imaging measures depending on budget availability) will be assessed within the 2 interventional groups. Analyses of the effect of the exposure to meditation/English learning (number of hours of practice, number of sessions...) will also be performed.

Objective 2ter (WP 1 to 6)

The difference between post-intervention (18 months) and long-term (29 mois +/- 6 mois months after the end of the intervention) measures in all cognitive, psychoaffectives, life factors, sleep assessments (and blood and imaging measures depending on budget availability) will be compared between the 2 interventional groups. The exposure to meditation/English learning (number of hours of practice, number of sessions...) will also be compared between the 2 interventional groups and taken into account when relevant.

Objective 3 (WP 4 and 5)

The difference between the inclusion and 18 months of measures from partners' questionnaires will be compared between the three groups. (see Section "*Descriptive analysis"* above for comparative methods).

Objective 3bis

The difference between measures from partners' questionnaires obtained post-intervention (18 months) versus 29 mois +/- 6 mois months later (i.e. 29 mois +/- 6 mois months after the end of the intervention) will be compared between the three groups. (see Section "*Descriptive analysis"* above for comparative methods).

Objective 3ter

The data collected during the qualitative interview on the participant's experience post-intervention will be analyzed.

Objective 4 (WP 1 to 6)

All cognitive measures, psychoaffective measures, life factors, blood, sleep and imaging measures at inclusion will be compared between the group of expert in meditation and the non-expert seniors. (see Section "*Descriptive analysis"* above for comparative methods)

Objective 5 (WP 1 and 4)

The main criteria are the fMRI neural activity measured at rest and during the task of SoVT-Rest in meditative states and non-meditative state in the group of experts in meditation.

The different measurement will be compared between mindfulness meditation versus non-meditative state and compassionate meditation versus non-meditative state. (see Section "*Descriptive analysis"* above for comparative methods)

Objective 6 (WP 2, 5 and 6)

To explore associations and/or correlations between psychoaffective measures, life factors and other measures. All statistical analyses of this part will be conducted on the senior non-expert population participants of AGE-Well study at baseline visit (also including data from two other studies when relevant, i.e. SCD-Well and IMAP+).

1. **Harmonize and validate the LEQ across countries (PI: Eider Arenaza-Urquijo)**

In a first step (test / construct validity) we will perform multiple regression between LEQ items and items of similar questionnaires expected to measure the same construct (Cognitive Activities Questionnaire and Modifiable Activities Questionnaire). In a second step, we will assess internal consistency reliability using Cronbach's alpha between items expected to measure the same construct (within the questionnaire). Finally, to test for cross-cultural equivalence, we will perform confirmatory factor analyses.

2. **Compare the educational system across countries (PIs: Eider Arenaza-Urquijo/Julie Gonneaud and Natalie Marchant)**

From the years of education of the participants (from both AGE-Well and SCD-Well), and based on a detailed understanding of the education scheme for each country, we will implement an algorithm allowing to calculate equivalent measures of years of education across countries.

3. **Harmonize and validate the neuropsychological tests and the scale and questionaires (within and across countries, when relevant) (PIs: Eider Arenaza-Urquijo/Julie Gonneaud and Natalie Marchant).** For most tests and questionnaires, especially those that are not standardized, we would need to perform item per item analyses to validate the tests acriss and within countries. The same procedure as described for the objective a) above will be performed for all tests, scales and questionnaires, when relevant.

4. **Identify the relative relationships between early vs. late life activities and amyloid deposition vs. grey matter (GM) volume (PIs: Julie Gonneaud & Gael Chetelat)**

We hypothesize that higher engagement in cognitive activities will be associated with less amyloid and more grey matter volume; while early versus late life activities would show specific relationships with amyloid deposition vs. grey matter volume.

The relative relationship between early vs. late life activities and amyloid deposition vs. grey matter volume would be measured by LEQ questionnaire, florbetapir-PET and structural MRI, respectively.

Finally, multiple regression analyses, after adjusting for cognitive and emotional scores (MMSE, GDS, IRI) will be performed.

5. **Association between quality of life (QoL), lifestyle and amyloid deposition (PI: Julie Gonneaud)**

We hypothesize that higher score in QoL questionnaires will be associated with less amyloid deposition. The objectives of this exploratory analysis are to know (a) what is the QoL subscore that best predict amyloid deposition ? (b) is QoL related to other psychoaffective measurements? and finally, (c) are the effects of QoL on amyloid deposition independent of psychoaffective factors?

To answer to those questions, statistical analyses will be conducted on the data of LoQ questionnaires and florbetapir-PET.

Firstly, we will identify those lifestyle and psychoaffective factors that are associated to QoL as confounders, by using univariate analyses, from the following list and include the significant ones as covariates: Lifestyle (Modifiable Activities Questionnaire (MAQ) or Physical activity for the Elderly (PASE), Mediterranean Adherence Screener (MEDAS), Alcohol consumption, Self-medication, Smoking); Cognitive Complaints, Depression (GDS), anxiety (STAI).

Then, a multivariate analysis by using a multiple regression analysis (voxel-wise) between QoL scores and florbetapir PET (controlling for age and sex) will be performed.

Finally, in following models we will control for potential confounding variables (i.e., those that are significantly related to QoL) in order to test for independent effects.

6. **Identify lifestyle predictors of memory complaints and their clinical significance (PI: Julie Gonneaud)**

We hypothesize that work, social environment, lifestyle and personal attitudes and beliefs will be associated to subjective cognitive complaints and modulate the relationship between complaints and (a) cognition and (b) AD biomarkers. The objectives of this analysis are to know (a) what are the best lifestyle predictors of cognitive complaints; (b) How do those factors modulate the relation between cognitive complaints and cognition or AD biomarkers?

The statistical analysis will be conducted on two steps with available data including: lifestyle and QoL questionnaires, cognitive complaints questionnaire, AD biomarkers (whole brain structural MRI, FDG and AV45), cognition (z-scores).

- STEP 1: Multiple regression analysis between cognitive complaints and lifestyle factors (probably lifestyle measurements will be reduced in "dimensions" or "factors" using factors analyses)
- STEP 2: (a) Multivariate analyses between the factors identified in STEP1 and cognition (including anxiety (STAI), depression (GDS), age and sex as covariates). Then, we will test for main effects and interactions (i.e., how the identified factors modulate the relation between cognitive complaints and cognition) (b): Multivariate analyses between factors identified in STEP1 and AD and aging biomarkers (including anxiety (STAI), depression (GDS), age and sex as covariates). Then, we will test for main effects and interactions.

7. **Lifestyle by APOE interactions on vascular risk, hippocampal volume and amyloid deposition (PI: Julie Gonneaud)**

We hypothesize that APOE status will have differential as well as dose-dependent effects on vascular risk, hippocampal volume and amyloid deposition.

Accordingly, the primary aims of this analysis were to investigate: (a) what are the protective/risk effects of APOE polymorphism on vascular risk factors, hippocampal volume and amyloid deposition; (b) is there a dose-dependent effect of APOE4? (c) How do these protective or risk effects interact with lifestyle?

Thus, lifestyle questionnaires, cognition (z-scores), florbetapir-PET, FDG-PET, MRI, vascular risk factors (cholesterol levels, diabetes, BMI) will be exploited. A multivariate analyses will be conducted (sex and age as covariates) including APOE4 status (e2, e4, e4e4), lifestyle (factors reduced in dimensions) and vascular risk (as dependent variable-model 1); or hippocampal volume (as dependent variable-model 2) or florbetapir-PET (as dependent variable-model 3). Finally, we will test for main effects and interactions. If more than one model is significant, we will perform another model including the significant variables.

8. **Does lifestyle underlie the gender bias in AD? (PI: Julie Gonneaud)**

We hypothesize that there will be gender specificities in lifestyle variables that will be differentially associated to brain measurements. For this purpose, lifestyle questionnaires, vascular risk, florbetapir-PET, structural MRI, FDG-PET, APOE genotype, cognition (z-scores) will be exploited and several comparisons will be then performed:

(1) Between groups comparison in lifestyle habits (t-test, women vs men).

(2) Between groups comparison in AD biomarkers and cognition.

(3) Mediation and moderation analyses of lifestyle factors on the biomarkers or cognitive domains where there are gender differences.

(4) Sub-analyses in women.

9. **Identify the combination of lifestyle risk and protective factors that are associated with the cognition vs AD biomarkers across disease stages (PI: Eider Arenaza-Urquijo)**

This exploratory analysis aims to evaluate the combination of lifestyle risk and protective factors that are associated with the cognition versus AD biomarkers across disease stages. For that, lifestyle questionnaires, AD and aging biomarkers (whole brain structural MRI, FDG and AV45) as well as clinical status of participants, will appropriately be exploited. A multiple regression analyses between lifestyle factors and AD and aging biomarkers including clinical group will be conducted. Finally, we will test for main effects and interaction by group.

10. **Relationship between lifestyle and blood markers? (PI: Eider Arenaza-Urquijo)**

This exploratory analysis aims essentially to determine the relationship between lifestyle and blood markers. General health biomarkers and lifestyle variables will be exploited and regression analysis and group comparisons will be then performed

Objective 7 (WP 1 to 6)

All biomarkers variables and gender of both populations (experts in meditation and senior non-experts, separately) will be used to find a link between the biomarkers of Alzheimer disease (AD) and structural and/or functional connectivity on the spread of lesions. All analyses will be conducted on the participants of Age-Well study at baseline visit.

1. **Relationships between age and AD risk factors on the one hand and attention measured with the Flanker task on the other hand (PI: Antoine Lutz)**

We hypothesize that age and AD risk factors will be associated with lower attention performances in specific attention subscore.

The Flancker task (orienting, executive function, alerting measures), T1 volume of grey matter density and PET measures of FDG and amyloid measures, alzheimer risk factors, memory complaints and family background, will be used.

Group comparisons and regressions analyses will be done after adjusting for age, gender, education and years of retreat.

2. **Relationship between sleep quality and attention (PI: Antoine Lutz)**

We hypothesize that low sleep quality will be associated with decreased attentional performances.

For this purpose, orienting, executive function, possibly other behavioural measures of attention, measures of sleep quality including questionnaires, actimetry and polysomnography index will be used and group comparisons and/or regressions will be conducted after adjusting for age, gender, education and years of retreat.

3. **Links between AD-risk factors and attention performances and neural substrates (PI: Fabienne Collette)**

We hypothesize that AD risk factors will be associated with decreased attention performances related with changes in brain activations (both increase and decrease activations).

For this purpose, attentional and executive measures from the neuropsychological battery as well as whole-brain analyses of fMRI data acquired during the attentional task will be exploited and analyses between groups (after adjusting for sex and age) will be performed with or without AD risk factors.

4. **Relationship between emotion and personality traits on attentional efficiency (PI: Olga Klimecki + Fabienne Collette)**

We hypothesize that higher attentional efficiency is associated to some emotion and personality traits.

The objective of this analysis is to show if there is, or not, a link between brain emotional reactivity and cognitive performances.

For this purpose, functional MRI and behavioural scores will be exploited and a regression analysis, after adjusting for sex, age, cognitive emotional scores post-MRI will be used.

5. **Validation of SoVT-REST in elderly/ Relationships between emotional fMRI data and resting state fMRI data & Neural response to emotional task during and after stimulation (fMRI, Baseline) (PI: Olga Klimecki)**

The main objectives of this analysis are to evaluate i) differences between exposure to emotional vs. non emotional videos and emotional inertia levels; ii) Effects of connectivity patterns in Default Mode Network during resting state on brain reactivity during and after emotional exposure, iii) Relationship between Emotion probes and brain activity in SoVT-Rest.

For this purpose, fMRI, post-SoVT, and some questionnaires. Repeated measures MANOVA, T-tests, post hoc corrections (i.e. Bonferroni); Functional whole brain connectivity/ group comparisons; Regression analysis/ ROI analysis / data-driven lexico-semantic mapping will be used.

6. **Identify the relationships between age/AD risk factors and emotions (PI: Olga Klimecki)**

We hypothesize that age and AD risk factors will be associated with changes in emotion measures.

The main objective of this analysis is to determine the effects of AD biomarkers on emotional processing.

For this purpose, functional MRI during emotional task, ageing/AD biomarkers; memory complaints; family background; structural MRI, FDG-PET and AV-45 PET whole brain will be exploited. Correlation analysis and group comparisons will be conducted after adjustment for cognitive and emotional scores (MMSE, GDS, IRI).

7. **Impacts of traits on caregiving, loneliness and prosociality in elderly (PI : Olga Klimecki)**

We hypothesize that there will be significant relationships between specific traits and caregiving and/or prosociality.

For these purposes, all parameters related to mindfulness, compassion, emotions and caregiving will be exploited and then hierarchical linear model with sex (level 1), group (level 2) and outcome variables will be performed. If necessary, correlations, covariations and cluster analyses may be also used.

8. **Relationships between emotions, emotional fMRI data and sleep/stress/ lifestyle and serotonin levels (PI: Olga Klimecki).**

We hypothesize that quality of sleep and level of stress will be related with behavioural measures of emotions and fMRI measures of the neural substrates of emotion.

Several parameters including : sleep measures, emotion measures, blood measure of stress, emotion questionnaires, SoVT Rest, and lifestyle quality measures will be exploited and hierarchical linear model with sex (level 1), group (level 2) and outcome variables will be used. If necessary, correlations, covariations and cluster analyses may also be used.

9. **Cognitive debt and relation to dementia risk (biological markers) (PI: Natalie Marchant).**

We hypothesize that higher cognitive debt will be associated with higher dementia risk.

For this purpose, several parameters will be exploited including: depression, anxiety, neuroticism, rumination, worry, biomarkers, ((Alzheimer risk factors: APOE4, AMYLOID Abeta+/- (0.1 and linear); Memory complaints; Family background; structural MRI, FDG-PET and AV-45 PET whole brain), and cognition.

A multivariate analysis by using factor method will be used and regression analysis will be conducted to evaluate the relationship between these late factors.

10. **How is cognitive debt related to latent mindfulness / meditation factor? (PI: Natalie Marchant).**

For this exploratory analysis, several parameters will be exploited including: depression, anxiety, neuroticism, rumination, worry, biomarkers, ((Alzheimer risk factors: APOE4, AMYLOID Abeta+/-(0.1 and linear); Memory complaints; Family background; structural MRI, FDG-PET and AV-45 PET whole brain), and cognition.

A multivariate analysis by using factor method will be performed and regression analysis will be conducted to evaluate the relationship between these late factors.

11. **Relationships between cognitive debt and cognition and between cognition and mindfulness (PI: Natalie Marchant).**

For this exploratory analysis, several parameters will be exploited including: depression, anxiety, neuroticism, rumination, worry, biomarkers, and cognition.

A multivariate analysis by using factor method will be used and regression analysis will be made to evaluate the relationship between these late factors.

12. **Biological determinants of ageing and risk factors of AD (PI: Géraldine Poisnel)**

For these exploratory analyses, all blood biomarkers will be analysed and then a) regression analyses and b) group comparisons will be conducted to assess the effects of age on blood biomarkers, the links between the blood biomarkers and the other markers of mental health and well being, as well as how populations (defined according to their risk of AD) differ in terms of blood biomarkers.

13. **Relationships between lifestyle and subjective and objective sleep quality (PI: Géraldine Rauchs & Eider Arenaza-Urquijo)**

We hypothesize that lifestyle with little stress; stimulating social, physical and cognitive activities will be associated with better sleep quality.

For this purpose, sleep parameters and lifestyle variables will be analysed.

After that, group comparisons, multivariate analysis by using multiple regression analysis will be used after adjusting for covariates having a significant effect in the sample of participants or well-described in the literature.

14. **Relationships between objective and subjective sleep quality and blood-based markers of ageing and AD (PI: Géraldine Rauchs & Géraldine Poisnel)**

We hypothesize that poor sleep quality or objective sleep disturbances will be related to changes of some blood-based markers sensitive to stress, ageing and AD.

Several parameters including: sleep parameters, ageing, AD, stress, mood, inflammation blood markers, will be exploited and group comparisons, multivariate analysis by using multiple regression analysis will be used after adjusting for covariates having a significant effect in the sample of participants or well-described in the literature.

15. **Relationship between sex/gender and blood and neuroimaging markers (PI: Gaël Chételat & Géraldine Poisnel)**

We hypothesize that sex/gender will modify blood markers and neuroimaging markers.

For that, sex/gender and neuroimaging markers will be exploited and group comparisons, multivariate analysis by using multiple regression analysis, will be used.

16. **Relationships between neuroimaging markers of ageing and AD and depression/anxiety (PI: Gaël Chételat & Natalie Marchant)**

We hypothesize that there will be specific links in regions sensitive to AD or ageing involved in emotion.

For this purpose, baseline T1 MRI and PET neuroimaging data, anxiety and depression scales will be exploited and group comparisons, multivariate analysis by using multiple regression analysis, will be used.

17. **Link between connectivity/brain networks and risk factors for AD (PI: Gaël Chételat)**

All parameters related to baseline structural T1 MRI plus baseline resting-state fMRI and DTI will be exploited.

Then, group comparisons and multivariate analysis by using multiple regression analysis will be conducted.

18. **Relationships between brain connectivity and the topography and spread (progression) of atrophy, hypometabolism and amyloid deposition (PI: Gaël Chételat).**

Using a machine learning model / a penalized least-squares linear Ridge model we will assess how atrophy/hypometabolism/amyloid deposition topography and propagation scores can be predicted from multiple predictors (vectors) reflecting selected brain features including structural MRI T1-weighted data, resting-state fMRI data and DTI data from IMAP+, AGE-Well and publicly available data.

19. **Relationships between the different neuroimaging modalities PI: Gaël Chételat).**

To further our understanding of the nature of the neuroimaging measures, their physiopathological meaning (the mechanisms subtending their variation across individuals), and the substrates, we will assess correlation and regression analyses (voxel-wise and ROI-based) between the different neuroimaging data acquired in Age-Well (together with data from IMAP+ when available).

20. **Role of WM lesions in ageing and AD (PI: Gaël Chételat)**

We hypothesize that WM lesions will increase the load towards AD.

For this purpose, multimodal MRI data for WM lesion segmentation, AD biomarkers and cognition will be exploited.

Finally, group comparisons and multivariate analysis (by using multiple regression analysis) will be performed.

21. **Links between medication and blood and neuroimaging measures (PI: Géraldine Poisnel & Gaël Chételat)**

For this exploratory analysis, prescribed medication, all blood biomarkers and neuroimaging data will be exploited and then a) regression analysis and b) groups comparison will be conducted.

Objective 8 (WP 6)

Comparison and correlations between FDG-PET measurement and early PET-Amyvid® perfusion measurement to better understand the links (and possible modifications of coupling) between metabolism and perfusion and compare the sensitivity of the two measures to assess the effects of the interventions.

Objective 9 (WP 1 to 6)

Comparison and correlations between measures at baseline, post-intervention (18 months) and 29 mois +/- 6 mois months after the end of the intervention in participants of the passive condition (control group) and participant' partners to identify the determinants of mental health and well-being in ageing, considering areas of cognition, psycho-affective/emotion, compassion, support, mindfulness, sleep, wellbeing and quality of life measures (and biological and radiological measures depending on budget availability).

REFERENCES

1. Guideline ICH - E9: Statistical Principles for Clinical Trials. 1998.

2. Guideline ICH - E3: Structure and Content of Clinical Trials. 1995.

3. Basic Statistical Reporting for Articles Published in Biomedical Journals: The « Statistical Analyses and Methods in the Published Literature » or The SAMPL Guidelines" | The EQUATOR Network [Internet]. [cité 9 mai 2014]. Disponible sur: http://www.equator-network.org/reporting-guidelines/sampl/

4. Dahl CJ, Lutz A, Davidson RJ. Reconstructing and deconstructing the self: cognitive mechanisms in meditation practice. Trends in cognitive sciences. 2015 Sep 1;19(9):515-23.

5. Schlosser M, Requier F, Deza-Araujo YI, Chételat G, Collette F, Klimecki O, Barnhofer T, Lutz A. Capturing complexity: Assessing the empirical value of a theory-based taxonomy of meditation practices. Submitted.

6. Arenaza-Urquijo EM, Landeau B, La Joie R, Mevel K, Mézenge F, Perrotin A, Desgranges B, Bartrés-Faz D, Eustache F, Chételat G. Relationships between years of education and gray matter volume, metabolism and functional connectivity in healthy elders. Neuroimage. 2013 Dec;83:450-7. doi: 10.1016/j.neuroimage.2013.06.053. PubMed PMID: 23796547.

7. La Joie R, Landeau B, Perrotin A, Bejanin A, Egret S, Pélerin A, Mézenge F, Belliard S, de La Sayette V, Eustache F, Desgranges B, Chételat G. Intrinsic connectivity identifies the hippocampus as a main crossroad between Alzheimer's and semantic dementia-targeted networks. Neuron. 2014 Mar 19;81(6):1417-28. doi: 10.1016/j.neuron.2014.01.026. PubMed PMID: 24656258.

8. Mevel K, Landeau B, Fouquet M, La Joie R, Villain N, Mézenge F, Perrotin A, Eustache F, Desgranges B, Chételat G. Age effect on the default mode network, inner thoughts, and cognitive abilities. Neurobiol Aging. 2013 Apr;34(4):1292-301. doi: 10.1016/j.neurobiolaging.2012.08.018. PubMed PMID: 23084083

9. Moher D, Schulz KF, Altman DG. The CONSORT statement: revised recommendations for improving the quality of reports of parallel-group randomized trials. Lancet. 14 avr 2001;357(9263):1191-1194.
