# Supplementary material for: Effect of an 18-Month Meditation Training on Regional Brain Volume and Perfusion in Older Adults: The Age-Well Randomized Clinical Trial
Source: JAMA Neurol. 2022 Oct 10;79(11):1165–74. doi: 10.1001/jamaneurol.2022.3185 (PMC9552046; doi:10.1001/jamaneurol.2022.3185)
Supplement: Supplement 2. — eAppendix 1. Inclusion and exclusion criteria for the Age-Well clinical trial eAppendix 2. List of assessments and corresponding outcomes eAppendix 3. Method details eAppendix 4. Details on the 15 subscales included in the global composite score of attention regulation, socio-emotional and self-knowledge capacities eAppendix 5. Results of the moderator analyses eAppendix 6. Baseline values of the composite scores by group eAppendix 7. Longitudinal changes from baseline (Pre) to 18 months (Post) in the composite scores by group eTable. Primary outcome descriptive results eReferences [file jamaneurol-e223185-s002.pdf]

Supplemental Online Content

Chételat G, Lutz A, Klimecki O, et al; Medit-Ageing Research Group. Effect of an 18-month meditation training on regional brain volume and perfusion in older adults: the Age-Well randomized clinical trial. *JAMA Neurol*. Published online October 10, 2022. doi:10.1001/jamaneurol.2022.3185

eAppendix 1. Inclusion and exclusion criteria for the Age-Well clinical trial
eAppendix 2. List of assessments and corresponding outcomes
eAppendix 3. Method details
eAppendix 4. Details on the 15 subscales included in the global composite score of attention regulation, socio-emotional and self-knowledge capacities
eAppendix 5. Results of the moderator analyses
eAppendix 6. Baseline values of the composite scores by group
eAppendix 7. Longitudinal changes from baseline (Pre) to 18 months (Post) in the composite scores by group
eTable. Primary outcome descriptive results
eReferences

This supplemental material has been provided by the authors to give readers additional information about their work.

Appendix 1: Inclusion and exclusion criteria for the Age-Well clinical trial

- **Inclusion Criteria**
  - Age ≥ 65 years
  - Autonomous
  - Living at home
  - Educational level ≥ 7 years (from the Preparatory Course - 1st grade - included)
  - Registered to the social security system
  - Motivated to effectively participate in the project and signing the informed consent form
  - Performance within the normal range on standardized cognitive tests according to agreed study-specific standards (age, sex and education level when available)
  - Native French speaker
  - Available to attend the intervention for the trial duration (24 months)
  - Retired for at least one year
  - No strong preference or aversion for an intervention group
  - No present or past regular or intensive practice of meditation or comparable practices; the practice is considered as regular and/or intensive if i) it occured more than one day per week for more than six consecutive months over the last 10 years, and/or in case of more than five consecutive days of intensive practice (internship or retreat) over the past 10 years, and/or of more than 25 days of retreats (cumulatively) within the last 10 years
  - Not speaking fluent English
- **Exclusion Criteria**
  - Safety concerns in relation to MR scanning (claustrophobia, ferromagnetic object) or PET scanning (Blood sampling to check hepatic and renal functions are performed before the PET scans; known hypersensibility to Amyvid® or Glucotep®)
  - Presence of a major neurological or psychiatric disorder (including an addiction to alcohol or drugs)
  - History of cerebral disease (vascular, degenerative, physical malformation, tumor, or head trauma with loss of consciousness for more than an hour)
  - Presence of a chronic disease or acute unstable illness (respiratory, cardiovascular, digestive, renal, metabolic, hematologic, endocrine or infectious)
  - Current or recent medication that may interfere with cognitive functioning (psychotropic, antihistaminic with anticholinergic action, anti-Parkinson's, benzodiazepines, steroidal anti-inflammatory long-term treatment, antiepileptic or analgesic drugs), the interfering nature of the different treatments being at the discretion of the investigating doctor
  - Being under legal guardianship or incapacitation
  - Participation in another biomedical research protocol including the injection of radiopharmaceuticals
  - Physical or behavioral inability to perform the follow-up visits as planned in the study protocol

© 2022 Chételat G et al. *JAMA Neurology.*

Appendix 2: List of assessments and corresponding outcomes

<table>
<tr><th>Measures collected at baseline and post-intervention (and at 9 months follow-up for a selected set of behavioral measures)</th><th>Outcomes</th></tr>
<tr><td>Behavioral measures:
Series of neuropsychological tests, scales and questionnaires selected as being particularly sensitive to aging and AD (e.g. assessing episodic memory, attention, executive functions and metacognition) and/or meditation practices (e.g. assessing mindfulness, compassion, and interoception), emotions (e.g. assessing anxiety, depression, empathy, emotion regulation, positive and negative emotions), or as they allow to assess different aspects of sleep quality, lifestyle, well-being, prosociality, loneliness, social support and quality of life.
Questionnaires were also proposed to a partner (i.e. a participant's close relative or friend) to assess how the partner perceives the mindfulness, compassion, depression, anxiety, and prosocialness of the participant as well as questions on the social support and the role of an informal carer of the partner.</td><td>Composite scores and raw individual measures of cognitive performance, well-being, mindfulness and meta-cognition, emotion-related questionnaires, altruism, pro-sociality, sleep quality, lifestyle, and quality of life of the participants. Partner perception of the participant's mindfulness, compassion, depression, anxiety, and prosocialness as well as questions on the social support and the role of an informal carer of the partner.</td></tr>
<tr><td>Neuroimaging measures:
1) Structural MRI
a) 3D T1-weighted and Fluid-attenuated inversion recovery - FLAIR

b) High-resolution proton-density focused on the hippocampus
c) Diffusion Kurtosis Imaging – DKI
d) Quantitative Susceptibility Mapping - QSM

2) Functional MRI -fMRI
a) Resting-state fMRI
b) Task-related fMRI
i) The AX-CPT task[14]

ii) The SoVT-Rest task

3) Resting-state EEG

4) Auditory event-related potential (ERP) using a mismatch negativity protocol sensitive to aging[15]

5) PET scans
a) Glucotep® (FDG)- PET scan
b) Amyvid® (Florbetapir, AV45)-PET scan</td><td>- Gray and white matter volumes
- White matter lesions (number and size per type and location)
- Hippocampal subfield volumes

- Fractional anisotropy and mean diffusivity
- Magnetic susceptibility index

- Brain functional connectivity

- Behavioral and brain activity measures associated with attentional processes (alertness, inhibition, sustained attention)
- Behavioral and brain activity and connectivity changes associated with emotions and emotional inertia

- Resting-state spontaneous oscillatory activity

- ERP measures of brain activity associated with auditory mismatch negativity

- Resting-state brain glucose consumption
- Brain perfusion from early florbetapir-PET acquisition
- Brain amyloid load from late florbetapir-PET acquisition</td></tr>
<tr><td>Biological measures from blood:
Fasting sampling performed in the morning and after one day of diet excluding serotonin-rich food (tomatoes, avocados, pineapple, chocolate, bananas…). 18 tubes (68mL) of blood collected at V1 and 16 tubes (62mL) at V3.</td><td>- Global health: blood count, glucose, cholesterol/lipid profile, urea, creatinine, Gamma-GlutamylTransferase, Glutamic Oxaloacetic Transaminase, Glutamic Pyruvic Transaminase, Brain Natriuretic Peptide, Thyroid Stimulating Hormone, Fibrinogen
- Stress/inflammation: high-sensitivity C-Reactive Protein, cytokines, cortisol, Peroxyredoxin-1, Epinephrine, Norepinephrine
- Aging/AD: telomere length, telomerase activity, β-amyloid (Aβ) 1-40/42, Phospho-Tau, tissue Plasminogen Activator, Plasminogen Activator Inhibitor-1, Brain Derived Neurotrophic Factor, insulin, Insulin Growth Factor-1, lymphocyte immunophenotyping, Neurofilament Light, Glial Fibrillary Acidic Protein
- Mood: serotonin,
- Sex/gender: bioavailable testosterone, estradiol, Sex Hormone Binding Globulin, DeHydroEpiAndrosterone Sulphate
- Genetic: Apolipoprotein E, Genome Wide Association Study
- Epigenetics</td></tr>
</table>

© 2022 Chételat G et al. *JAMA Neurology.*

**Objective measures of sleep**:<br>1) 1-week wrist actigraphy recording<br>2) 2-night at-home polysomnography with 2D-object location task performed before and after night sleep<br>3) 5-night recording with Somno-Art®	- Indices of mean sleep duration, sleep fragmentation and regularity of the rest-activity cycle<br>- Multiple indices of sleep quality derived from EEG analyses and behavioral measures of overnight memory consolidation<br>- Indices of night-to-night variability of sleep quality and quantity.

© 2022 Chételat G et al. *JAMA Neurology.*

Appendix 3: Method details

i) For randomization, masking and the interventions

All study personnel were instructed to refrain from interactions with participants that could disclose their treatment allocation. Moreover, participants were continuously reminded throughout the study that they should not disclose their treatment allocation to any study personnel except their teachers.
The investigators actively tried to balance researcher allegiance to the two interventions and keep all communications about the study fully balanced regarding expected effects of meditation and non-native language learning, and underpinning the equipoise regarding their use.
The intervention programs were provided by meditation versus English (as the non-native language) expert teachers/instructors in Caen (France).

ii) for neuroimaging data processing and analyses

All participants were scanned at Cyceron Center (Caen, France) on the same MRI (Philips Achievia 3.0T scanner) and PET (Discovery RX VCT 64 PET-CT, General Electric Healthcare) cameras. All data were processed using the Statistical Parametric Mapping software (SPM, version 12, Wellcome Trust Centre for Neuroimaging, http://www.fil.ion.ucl.ac.uk/spm/software/spm12) as described elsewhere.[1,2]
T1-weighted images were segmented using FLAIR images, spatially normalized to the Montreal Neurological Institute (MNI) template and gray matter segments were modulated to correct for non-linear warping so that values in resultant images are expressed as volume corrected for brain size. Images from each step were quality checked to ensure the absence of artifacts or segmentation failures. Early and late Amyvid® positron emission tomography (PET) scans were acquired over 10 min beginning respectively immediately after, and 50 minutes after, the intravenous injection of ≈4MBq/Kg of Florbetapir. A transmission scan was performed for attenuation correction before the PET acquisition. Due to the high lipophilic nature of the tracer, early Amyvid®-PET images allow to measure brain perfusion, itself tightly coupled to cerebral glucose metabolism,[3,4] known to be particularly sensitive to aging and Alzheimer's disease. Early Amyvid®-PET images were reconstructed from 1 to 6 min, coregistered onto their corresponding T1-weighted MRI scans, normalized to the MNI template applying the deformation parameters from the corresponding MRI, and scaled by the white matter average binding to obtain standardized uptake values ratio (SUVR) images. As described in further details elsewhere,[5] the white matter reference region corresponds to the average of the segmented white matter images from a group of healthy adults aged between 20 and 60 years old, scanned at the same center (IMAP protocol, Cyceron center), thresholded to 0·8 and eroded with a 6 mm^3 sphere to avoid contamination by the gray matter and cerebrospinal fluid binding.
Average gray matter volume and perfusion were extracted from the processed images (i.e. modulated segmented gray matter images and normalized scaled early Amyvid®-PET SUVR images, respectively) in two regions of interest (ROI): the anterior cingulate cortex (corresponding to the bilateral pre and sup anterior cingulate areas of the AAL atlas[6–8]) and the insula (encompassing the bilateral dorsal agranular and dysgranular insula areas from the Brainnetome atlas[9]). To ensure only gray matter voxels were included, each ROI was masked using a gray matter mask generated from the averaged gray matter, white matter and CSF segments of all participants and selecting only voxels with a gray matter probability i) higher than both white matter and CSF probabilities (gray matter>white matter ∩ gray matter>CSF) and ii) above a threshold of 0·3 (gray matter>0·3), to reduce the probability of overlap with other tissue classes.[10]

The brain amyloid status of each participant was determined from the late Amyvid® PET scans using a standard technique classically used in our lab.[11] Briefly, late Amyvid®-PET images were coregistered onto their corresponding T1-weighted MRI scans, normalized to the MNI template applying the deformation parameters from the corresponding MRI, and scaled using the cerebellar gray matter mean value as a reference. Then the global neocortical SUVR value was extracted for each individual using a predefined neocortex mask (including all regions but the cerebellum, hippocampus, amygdala, and subcortical gray nuclei). This global neocortical SUVR was used to classify subjects as florbetapir positive or negative, using a threshold derived from an independent group of 41 young individuals from the IMAP project (16/25 females/males; age = 28·40 ± 6·06 years).[12,13] The positivity threshold was obtained from the 99·9th percentile of the global neocortical SUVR of these 41 healthy young controls, corresponding to a Florbetapir SUVR of 1·31. Individuals with values above this threshold were considered as amyloid-positive and those below this threshold as amyloid-negative.

iii) for statistical analyses

In addition to the intention-to-treat analysis of the primary outcome as described in the main manuscript, a sensitivity analysis to missing data was performed using a maximum bias analysis strategy, with missing data

© 2022 Chételat G et al. *JAMA Neurology.*

replaced by a failure (worst value of change observed all groups combined) in one group and by a success (best value of change observed all groups combined) in the other, and vice versa.

A *'minimum intervention'* analysis was also conducted. It included only randomized participants who attended at least 20% of their allocated intervention classes for the meditation or non-native language group or were in the no-intervention group, and who had primary endpoints available.

Finally, we conducted several post-hoc analyses: 1) additional adjustment for recruitment wave or baseline brain amyloid mean SUVR (quantitative value) in between-group comparisons for the primary outcome; 2) primary outcome analysis stratified by recruitment wave; and 3) subgroup analyses to explore the moderating effect of risk factors for Alzheimer's disease: APOE genotype (no e4 allele *vs.* at least one), brain amyloid status (see above in ii) for description of the method), familial history of dementia or presence of at least one of these risk factors. All the subgroup analyses were conducted by including an interaction term between the allocated group and each moderator considered, in distinct models. The mean differences between groups and their 95% confidence intervals were then computed from these models for each category of the moderators.

© 2022 Chételat G et al. *JAMA Neurology.*

Appendix 4: Details on the 15 subscales included in the global composite score of attention regulation, socio-emotional and self-knowledge capacities. Descriptive statistics for scales comprising the composite scores by group and visit. To derive the three subscore composites, we first ensured that higher scores on each scale indicated higher capacities, reverse-scoring them when appropriate. Second, we subtracted each scale score at each time point from the pooled mean at baseline. Third, we divided this difference score by the pooled standard deviation at baseline. Fourth, each composite score was computed by averaging the z-scores of the scales that were assigned to the respective composite, yielding three composite subscores with a baseline mean of 0 and a standard deviation smaller than one. The global composite score was derived by averaging the attention regulation, socio-emotional and self-knowledge composite subscores. Fifth, to ease longitudinal data interpretation, we re-standardised each composite score and subscores so that longitudinal changes in each score reflected changes in standard deviation units.

	**Meditation**				**Non-native language**				**Passive**			
	Pre		Post		Pre		Post		Pre		Post	
Composites and measures	n	Mean (SD)	n	Mean (SD)	n	Mean (SD)	n	Mean (SD)	n	Mean (SD)	n	Mean (SD)
**Attention regulation**[1]	44	-0·08 (0·97)	45	0·40 (0·81)	44	-0·02 (1·0)	44	0·08 (0·95)	46	0·09 (1·0)	45	-0·004 (0·97)
MAIA noticing	45	3·1 (1·2)	45	3·5 (0·9)	44	3·3 (1·1)	44	3·5 (1·0)	46	3·6 (1·1)	45	3·4 (1·1)
MAIA attention regulation	45	2·7 (1·0)	45	3·4 (0·8)	44	2·8 (0·9)	44	3·0 (0·7)	46	3·0 (0·9)	45	2·9 (0·9)
MAIA emotional awareness	45	3·5 (1·0)	45	3·8 (0·9)	44	3·5 (1·0)	44	3·6 (0·9)	46	3·4 (1·1)	45	3·3 (1·0)
MAIA self-regulation	45	3·1 (0·9)	45	3·7 (0·8)	44	3·2 (1·0)	44	3·4 (0·8)	46	3·0 (1·0)	45	3·0 (1·0)
MAIA body listening	44	2·3 (1·2)	44	3·2 (1·0)	44	2·7 (1·2)	44	2·7 (1·1)	46	2·4 (1·2)	44	2·5 (1·2)
FFMQ observing	45	9·4 (2·9)	45	9·4 (2·6)	44	8·8 (2·9)	44	8·9 (3·0)	46	10·3 (3·0)	45	9·9 (2·9)
FFMQ act with awareness	45	11·6 (2·1)	45	11·6 (2·2)	44	11·7 (2·2)	44	11·2 (2·3)	46	11·8 (2·3)	45	11·8 (2·2)
**Socio-emotional**[1]	45	0·10 (1·08)	45	0·14 (1·15)	44	-0·06 (0·96)	44	-0·33 (1·15)	46	-0·04 (0·97)	45	-0·20 (0·9)
Compassionate Love Scale	45	92·8 (22·1)	45	98·2 (18·8)	44	88·7 (21·9)	44	85·6 (22·8)	46	90·1 (19·6)	45	88·6 (20·1)
IRI empathic concern	45	19·4 (4·8)	45	19·4 (4·4)	44	20·3 (4·0)	44	19·1 (4·6)	46	19·8 (3·9)	45	18·6 (3·7)
IRI perspective taking	45	17·8 (3·6)	45	17·9 (3·9)	44	16·8 (3·8)	44	16·4 (2·9)	46	17·6 (3·1)	45	17·5 (2·8)
Prosocialness Scale	45	61·8 (7·6)	45	60·6 (9·4)	44	60·1 (7·4)	44	58·0 (10·8)	46	59·3 (9·5)	45	58·4 (7·8)
**Self-knowledge**[1]	45	-0·12 (1·10)	45	0·15 (1·15)	44	-0·12 (0·96)	44	-0·13 (1·06)	46	0·24 (0·91)	45	0·26 (0·94)
Drexel Defusion Scale	45	34·4 (5·7)	45	34·6 (6·4)	44	33·5 (5·7)	44	34·0 (7·2)	46	35·2 (5·4)	45	35·4 (5·7)
FFMQ non-judging	45	11·1 (2·7)	45	11·2 (2·5)	44	11·7 (1·9)	44	11·6 (2·4)	46	12·2 (2·3)	45	12·2 (1·8)
FFMQ non-reactivity	45	9·2 (2·4)	45	10·5 (2·5)	44	9·7 (2·0)	44	9·3 (2·1)	46	10·3 (2·2)	45	10 (2·3)
IRI personal distress[2]	45	18·4 (5·0)	45	18·9 (5·1)	44	16·9 (5·0)	44	17·2 (5·4)	46	18·1 (5·7)	45	18·8 (4·6)
**Global**[2]	44	-0·07 (1·12)	45	0·37 (1·18)	44	-0·10 (0·94)	44	-0·19 (1·11)	46	0·16 (0·94)	45	0·04 (0·90)

Note. SD = standard deviation; MAIA = Multidimensional Assessment of Interoceptive Awareness; FFMQ = Five Facet Mindfulness Questionnaire; IRI = Interpersonal Reactivity Index.
[1]These scores have been standardised using the baseline mean and standard deviation of the total sample.
[2]Here, higher scores indicate lower levels of distress.

© 2022 Chételat G et al. *JAMA Neurology.*

Appendix 5: Results of the moderator analyses. Subgroup analyses to explore the moderating effect of risk factors for Alzheimer's disease: APOE genotype (no ε4 allele *vs.* at least one), brain amyloid status, and familial history of dementia, or presence of at least one of these risk factors, for the anterior cingulate cortex (ACC) volume (**A**), ACC perfusion (**B**), insula volume (**C**), and insula perfusion (**D**).

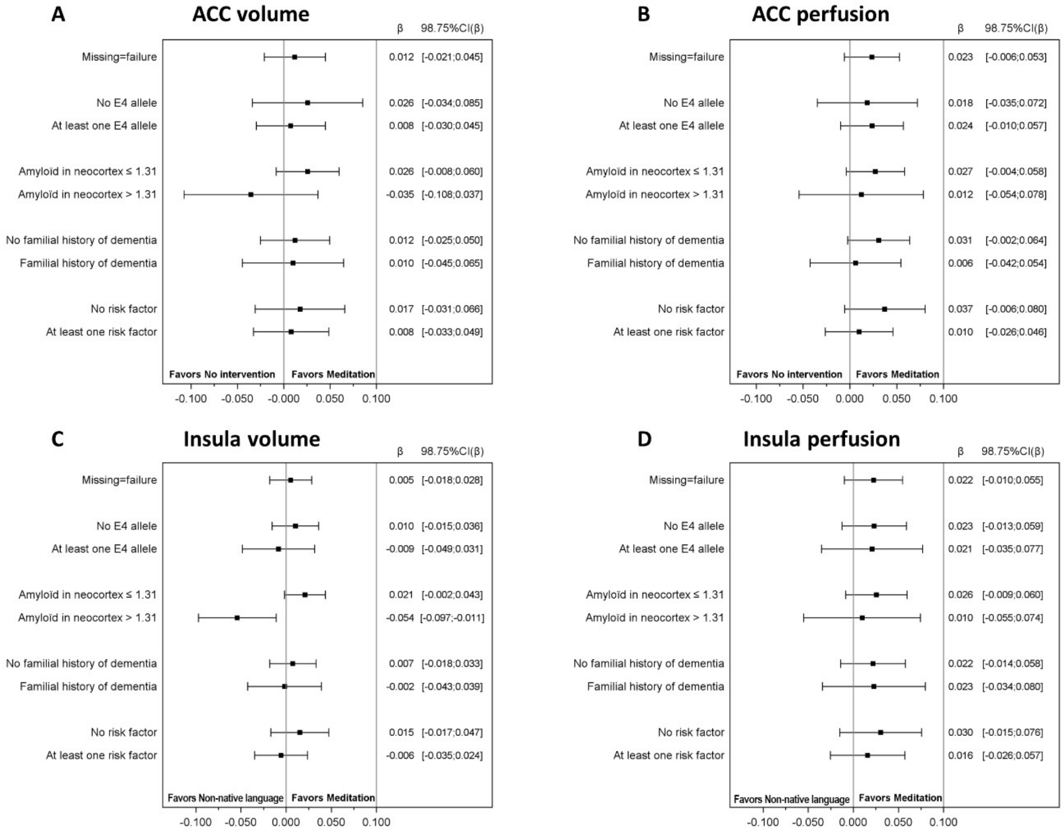

© 2022 Chételat G et al. *JAMA Neurology.*

Appendix 6: Baseline values of the composite scores by group.

	Meditation		Non-native language		No intervention	
	N	Mean (SD)	N	Mean (SD)	N	Mean (SD)
Attention regulation	44	-0.08 (0.97)	45	0.004 (1.00)	45	0.07 (1.05)
Socio-emotional	45	0.10 (1.08)	45	-0.07 (0.95)	45	-0.03 (0.98)
Self-knowledge	45	-0.12 (1.10)	45	-0.09 (0.97)	45	0.21 (0.91)
Global	44	-0.07 (1.12)	45	-0.08 (0.94)	45	0.14 (0.94)

© 2022 Chételat G et al. *JAMA Neurology.*

Appendix 7: Longitudinal changes from baseline (Pre) to 18 months (Post) in the composite scores by group. The figure displays observed standardized means and SEs (error bars = 1 SE) based on all available Age-Well data. The evolution of the observed scores in the passive control group is provided for descriptive purposes.

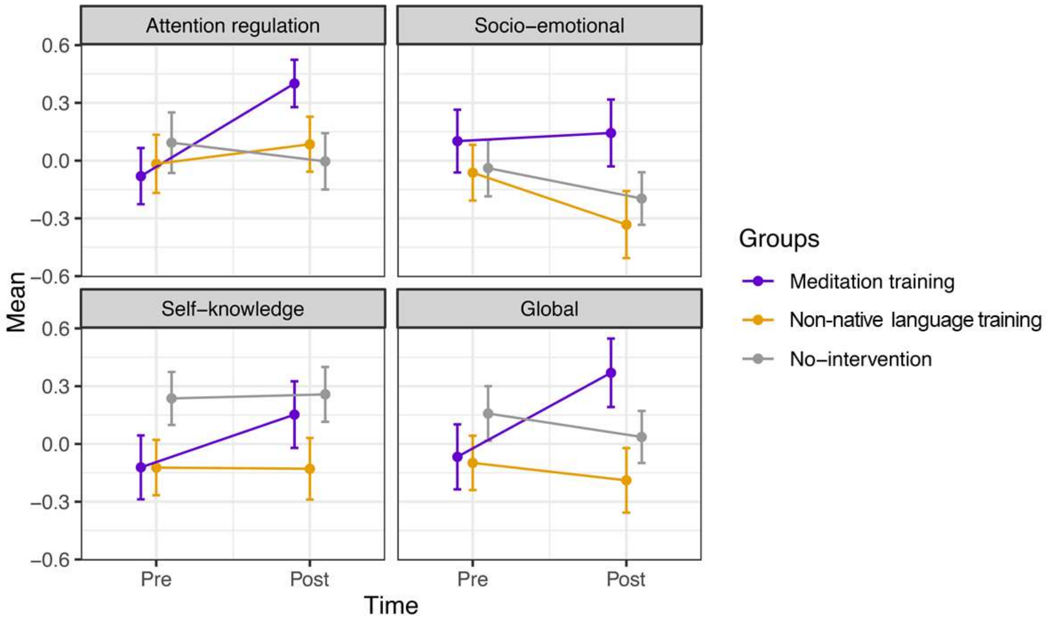

© 2022 Chételat G et al. *JAMA Neurology.*

eTable. Primary outcome descriptive results

	Meditation training (n=45)			Non-native language training (n=45)			No-intervention (n=46)			Mean (98.75%CI) difference between groups	
	N	Mean (SD)	Mean (SD) observed change	N	Mean (SD)	Mean (SD) observed change	N	Mean (SD)	Mean (SD) observed change	Adjusted*	p value†
ACC volume											
Baseline	45	3.006 (0.208)		45	2.939 (0.231)		46	2.988 (0.212)			
18 months	45	2.983 (0.212)	-0.023 (0.059)	45	2.920 (0.233)	-0.019 (0.061)	45	2.967 (0.202)	-0.032 (0.055)‡	0.012 (-0.021 to 0.045)‡	0.36‡
ACC perfusion											
Baseline	44	1.587 (0.099)		45	1.584 (0.090)		45	1.605 (0.095)			
18 months	45	1.573 (0.110)	-0.011 (0.051)	45	1.557 (0.086)	-0.027 (0.044)	45	1.578 (0.091)	-0.031 (0.051)‡	0.022 (-0.007 to 0.052) ‡	0.06‡
Insula volume											
Baseline	45	2.099 (0.110)		45	2.075 (0.155)		46	2.073 (0.128)			
18 months	45	2.079 (0.111)	-0.020 (0.036)	45	2.051 (0.157)	-0.024 (0.046)	45	2.055 (0.134)	-0.022 (0.037)¶	0.005 (-0.018 to 0.028)¶	0.58¶
Insula perfusion											
Baseline	44	1.700 (0.100)		45	1.699 (0.119)		45	1.724 (0.097)			
18 months	45	1.702 (0.104)	0.002 (0.054)	45	1.678 (0.136)	-0.021 (0.058)	45	1.692 (0.088)	-0.032 (0.063)¶	0.022 (-0.011 to 0.054)¶	0.09¶

* Adjusted on age, sex, education, baseline Mini Mental State Examination score and baseline value of the corresponding primary outcome. † Significance threshold set at 0.0125 (Bonferroni correction for multiple testing). ‡ planned comparison = Meditation *versus* No-intervention. ¶ planned comparison = Meditation *versus* Non-native language training. CI = confidence interval.

© 2022 Chételat G et al. *JAMA Neurology.*

References

1 André C, Rehel S, Kuhn E, *et al.* Association of Sleep-Disordered Breathing With Alzheimer Disease Biomarkers in Community-Dwelling Older Adults: A Secondary Analysis of a Randomized Clinical Trial. *JAMA Neurol* 2020; **77**: 716–24.

2 Ourry V, Gonneaud J, Landeau B, *et al.* Association of quality of life with structural, functional and molecular brain imaging in community-dwelling older adults. *Neuroimage* 2021; **231**: 117819.

3 Hsiao I-T, Huang C-C, Hsieh C-J, *et al.* Correlation of early-phase 18F-florbetapir (AV-45/Amyvid) PET images to FDG images: preliminary studies. *Eur J Nucl Med Mol Imaging* 2012; **39**: 613–20.

4 Lin K-J, Hsiao I-T, Hsu J-L, *et al.* Imaging characteristic of dual-phase (18)F-florbetapir (AV-45/Amyvid) PET for the concomitant detection of perfusion deficits and beta-amyloid deposition in Alzheimer's disease and mild cognitive impairment. *Eur J Nucl Med Mol Imaging* 2016; 43: 1304-14..

5 Gonneaud J, Arenaza-Urquijo EM, Mézenge F, *et al.* Increased florbetapir binding in the temporal neocortex from age 20 to 60 years. *Neurology* 2017; **89**: 2438–46.

6 Tzourio-Mazoyer N, Landeau B, Papathanassiou D, *et al.* Automated anatomical labeling of activations in SPM using a macroscopic anatomical parcellation of the MNI MRI single-subject brain. *Neuroimage* 2002; **15**: 273–89.

7 Rolls ET, Joliot M, Tzourio-Mazoyer N. Implementation of a new parcellation of the orbitofrontal cortex in the automated anatomical labeling atlas. *Neuroimage* 2015; **122**: 1–5.

8 Rolls ET, Huang C-C, Lin C-P, Feng J, Joliot M. Automated anatomical labelling atlas 3. *Neuroimage* 2020; **206**: 116189.

9 Fan L, Li H, Zhuo J, *et al.* The Human Brainnetome Atlas: A New Brain Atlas Based on Connectional Architecture. *Cereb Cortex* 2016; **26**: 3508–26.

10 Villain N, Fouquet M, Baron J-C, *et al.* Sequential relationships between gray matter and white matter atrophy and brain metabolic abnormalities in early Alzheimer's disease. *Brain* 2010; **133**: 3301–14.

11 Kuhn E, Moulinet I, Perrotin A, *et al.* Cross-sectional and longitudinal characterization of SCD patients recruited from the community versus from a memory clinic: subjective cognitive decline, psychoaffective factors, cognitive performances, and atrophy progression over time. *Alzheimers Res Ther* 2019; **11**: 61.

12 Perrotin A, La Joie R, de La Sayette V, *et al.* Subjective cognitive decline in cognitively normal elders from the community or from a memory clinic: Differential affective and imaging correlates. *Alzheimers Dement* 2017; **13**: 550–60.

13 Besson FL, La Joie R, Doeuvre L, *et al.* Cognitive and Brain Profiles Associated with Current Neuroimaging Biomarkers of Preclinical Alzheimer's Disease. *J Neurosci* 2015; **35**: 10402–11.

14 Braver TS, Barch DM, Gray JR, Molfese DL, Snyder A. Anterior cingulate cortex and response conflict: effects of frequency, inhibition and errors. *Cereb Cortex* 2001; **11**: 825–36.

15 Babiloni F, Gee J. The Power of Connecting Dots: Advanced Techniques to Evaluate Brain Functional Connectivity in Humans. *IEEE Trans Biomed Eng* 2016; **63**: 2447–9.

© 2022 Chételat G et al. *JAMA Neurology.*
