# Supplementary material for: Effect of an 18-Month Meditation Training on Regional Brain Volume and Perfusion in Older Adults: The Age-Well Randomized Clinical Trial
Source: JAMA Neurol. 2022 Oct 10;79(11):1165–74. doi: 10.1001/jamaneurol.2022.3185 (PMC9552046; doi:10.1001/jamaneurol.2022.3185)
Supplement: Supplement 3. — The Medit-Ageing Research Group nonauthor collaborators [file jamaneurol-e223185-s003.pdf]

Supplemental Online Content: Nonauthor Collaborators
*First name, last name, and suffix (if applicable) are required and will appear in PubMed.

## *Group Name(s): Medit-Ageing Research Group

| *First Name and Middle Initial(s) | *Last Name | *Suffix (eg, Jr, III) | Academic Degrees | Institution | Location (city, state/province, country) | Role or Contribution, eg, chair, principal investigator | Group (if more than 1 Group listed in the byline) and/or Subgroup (eg, Steering Committee) |
|---|---|---|---|---|---|---|---|
| Florence | ALLAIS | | BSc | INSERM | Bordeaux,France | data manager | NA |
| Claire | ANDRÉ | | PhD | INSERM | Caen, France | PhD student | NA |
| Eider M | ARENAZA URQUIJO | | PhD | INSERM | Caen, France | post-doctoral position | NA |
| Julien | ASSELINEAU | | MSc | INSERM | Bordeaux,France | statistician | NA |
| Sebastian | BAEZ LUGO | | MSc | UNIGE | Geneva, Switzerland | PhD student | NA |
| Martine | BATCHELOR | | | independent | Bordeaux,France | meditation teacher | NA |
| Axel | BEAUGONIN | | | independent | Caen/Paris, France | meditation teacher | NA |
| Alexandre | BEJANIN | | PhD | INSERM | Caen, France | post-doctoral position | NA |
| Pierre | CHAMPETIER | | MSc | UNICAEN | Caen, France | PhD student | NA |
| Gaël | CHÉTELAT | | PhD | INSERM | Caen, France | Coordinator, WP6 leader, research director | NA |
| Anne | CHOCAT | | MD | INSERM | Caen, France | investigating medical doctor | NA |
| Fabienne | COLLETTE | | PhD | ULG | Liege, Belgium | WP3 leader, research director | NA |
| Roxane | COUERON | | MSc | INSERM | Lyon, France | clinical research assistant | NA |
| Sophie | DAUTRICOURT | | MD PhD | UNICAEN | Caen/ Lyon, France | PhD student | NA |
| Robin | DE FLORES | | PhD | INSERM | Caen, France | post-doctoral position | NA |
| Vincent | DE LA SAYETTE | | MD PhD | CHU Caen | Caen, France | Principal Investigating medical doctor | NA |
| Pascal | DELAMILLIEURE | | MD PhD | CHU Caen | Caen, France | meditation teacher | NA |
| Marion | DELARUE | | MSc | INSERM | Caen, France | neuropsychologist | NA |
| Yacila | DEZA ARAUJO | | PhD | UNIGE | Geneva, Switzerland | post-doctoral position | NA |
| Francesca | FELISATTI | | MSc | INSERM | Caen, France | PhD student | NA |
| Eglantine | FERRAND DEVOUGE | | MD | INSERM | Caen, France | investigating medical doctor | NA |
| Eric | FRISON | | MD PhD | INSERM | Bordeaux,France | methodologist | NA |
| Antoine | GARNIER CRUSSARD | | MDMSc | CHU Lyon | Lyon, France | PhD student | NA |
| Francis | GHEYSEN | | MD | independent | Caen, France | meditation teacher | NA |
| Julie | GONNEAUD | | PhD | INSERM | Caen, France | WP2 leader, post-doctoral position | NA |
| Marc | HEIDMANN | | MSc | INSERM | Lyon, France | PhD student | NA |
| Thien (Titi) | HUONG TRAN (DOLMA) | | | INSERM | Paris, France | meditation teacher | NA |
| Frank | JESSEN | | MD PhD | UKK | Bonn, Germany | WP5 deputy, principal investigating medical doctor | NA |
| Olga | KLIMECKI | | PhD | UNIGE | Geneva, Switzerland | WP4 leader, researcher | NA |
| Pierre | KROLAK SALMON | | MD PhD | HCL | Lyon, France | principal investigating medical doctor | NA |
| Elizabeth | KUHN | | PhD | INSERM | Caen, France | PhD student | NA |
| Brigitte | LANDEAU | | MSc | INSERM | Caen, France | neuroimaging development engineer | NA |

*First name, last name, and suffix (if applicable) are required and will appear in PubMed.

| ***First Name and Middle Initial(s)** | ***Last Name** | ***Suffix (eg, Jr, III)** | Academic Degrees | Institution | Location (city, state/province, country) | Role or Contribution, eg, chair, principal investigator | Group (if more than 1 Group listed in the byline) and/or Subgroup (eg, Steering Committee) |
|---|---|---|---|---|---|---|---|
| Gwendoline | LE DU | | MSc | INSERM | Caen, France | technician | NA |
| Valérie | LEFRANC | | BA | INSERM | Caen, France | technician | NA |
| Antoine | LUTZ | | PhD | INSERM | Lyon, France | WP1 leader, research director | ExCom |
| Natalie | MARCHANT | | PhD | UCL | London, United Kingdom | WP5 leader, associate professor | ExCom |
| Florence | MEZENGE | | BA | INSERM | Caen, France | neuroimaging engineer assistant | NA |
| Jose Luis | MOLINUEVO | | MDPhD | IDIBAPS | Barcelona, Spain | medical doctor | NA |
| Inès | MOULINET | | PhD | INSERM | Caen, France | PhD student | NA |
| Valentin | OURRY | | PhD | INSERM | Caen, France | PhD student | NA |
| Cassandre | PALIX | | MSc | INSERM | Caen, France | PhD student | NA |
| Léo | PALY | | MSc | INSERM | Caen, France | neuropsychologist | NA |
| Géraldine | POISNEL | | PhD | INSERM | Caen, France | WP7 leader, research engineer | NA |
| Stefano | POLETTI | | MSc | INSERM | Lyon, France | psychologist | NA |
| Anne | QUILLARD | | MD | INSERM | Caen, France | investigating medical doctor | NA |
| Géraldine | RAUCHS | | PhD | INSERM | Caen, France | researcher | NA |
| Stéphane | REHEL | | PhD | INSERM | Caen, France | PhD student | NA |
| Florence | REQUIER | | MSc | ULG | Liege, Belgium | PhD student | NA |
| Eric | SALMON | | MD PhD | ULG | Liege, Belgium | executive medical director | NA |
| Raquel | SANCHEZ | | MD PhD | IDIBAPS | Barcelona, Spain | principal investigating medical doctor | NA |
| Corinne | SCHIMMER | | MSc | UNICAEN | Caen, France | English teacher | NA |
| Marco | SCHLOSSER | | MSc | UCL | London, United Kingdom | PhD student | NA |
| Christine | SCHWIMMER | | PhD | INSERM | Bordeaux, France | project manager | NA |
| Siya | SHERIF | | PhD | INSERM | Caen, France | research engineer | NA |
| Edelweiss | TOURON | | MSc | INSERM | Caen, France | PhD student | NA |
| Matthieu | VANHOUTTE | | PhD | INSERM | Caen, France | post-doctoral position | NA |
| Denis | VIVIEN | | PhD | INSERM | Caen, France | biomarker expert, research director | NA |
| Patrik | VUILLEUMIER | | MD | UNIGE | Geneva, Switzerland | WP4 deputy, research director | NA |
| Cédrick | WALLET | | MSc | INSERM | Bordeaux, France | data manager | NA |
| Caitlin | WARE | | MSc | INSERM | Caen, France | English teacher | NA |
| Miranka | WIRTH | | PhD | DZNE | Dresden, Germany | WP2 deputy, researcher | NA |
