# Supplementary material for: Effect of an 18-Month Meditation Training on Regional Brain Volume and Perfusion in Older Adults: The Age-Well Randomized Clinical Trial
Source: JAMA Neurol. 2022 Oct 10;79(11):1165–74. doi: 10.1001/jamaneurol.2022.3185 (PMC9552046; doi:10.1001/jamaneurol.2022.3185)
Supplement: Supplement 4. — Data sharing statement. [file jamaneurol-e223185-s004.pdf]

Data Sharing Statement

Chételat. Effect of an 18-Month Meditation Training on Regional Brain Volume and Perfusion in Older Adults. *JAMA Neurol*. Published October 10, 2022. doi:10.1001/jamaneurol.2022.3185

Data
Data available: Yes
Data types: Deidentified participant data
How to access data: The data underlying this report are made available on request following a formal data sharing agreement and approval by the consortium and executive committee (https://silversantestudy.eu/2020/09/25/data-sharing).
When available: With publication

Supporting Documents
Document types: None

Additional Information
Who can access the data: The Material can be mobilized, under the conditions and modalities defined in the Medit-Ageing Charter, by any research team belonging to an Academic. The Material may also be mobilized by non-academic third parties, under conditions, in particular financial, which will be established by separate agreement between Inserm and by the said third party. Data sharing policies described in the Medit-Ageing Charter are in compliance with our ethics approval and guidelines from our funding body.
Types of analyses: for carrying out a scientific research project relating to the scientific theme of mental health and well-being in older people
Mechanisms of data availability: on request following a formal data sharing agreement and approval by the consortium and executive committee (https://silversantestudy.eu/2020/09/25/data-sharing)
